# Whole genomes define concordance of matched primary, xenograft, and organoid models of pancreas cancer

**DOI:** 10.1371/journal.pcbi.1006596

**Published:** 2019-01-10

**Authors:** Deena M. A. Gendoo, Robert E. Denroche, Amy Zhang, Nikolina Radulovich, Gun Ho Jang, Mathieu Lemire, Sandra Fischer, Dianne Chadwick, Ilinca M. Lungu, Emin Ibrahimov, Ping-Jiang Cao, Lincoln D. Stein, Julie M. Wilson, John M. S. Bartlett, Ming-Sound Tsao, Neesha Dhani, David Hedley, Steven Gallinger, Benjamin Haibe-Kains

**Affiliations:** 1 Centre for Computational Biology, Institute of Cancer and Genomic Sciences, University of Birmingham, Birmingham, United Kingdom; 2 School of Science and Technology, Nottingham Trent University, Nottingham, United Kingdom; 3 PanCuRx Translational Research Initiative, Ontario Institute of Cancer Research (OICR), Toronto, Ontario, Canada; 4 Informatics and Bio-computing Program, Ontario Institute for Cancer Research, Toronto, Ontario, Canada; 5 Princess Margaret Living Biobank Core, University Health Network, Toronto, Ontario, Canada; 6 Department of Statistical Science, University of Toronto, Toronto, Ontario, Canada; 7 Department of Pathology, University Health Network, University of Toronto, Toronto, Ontario, Canada; 8 UHN Program in BioSpecimen Sciences, Department of Pathology, University Health Network, Toronto, Ontario, Canada; 9 Transformative Pathology, Ontario Institute for Cancer Research, Toronto, Ontario, Canada; 10 Division of Medical Oncology, Princess Margaret Cancer Centre, Toronto, Ontario, Canada; 11 Molecular Genetics Department, University of Toronto, Toronto, Ontario, Canada; 12 Lunenfeld-Tanenbaum Research Institute, Mount Sinai Hospital, Toronto, Ontario, Canada; 13 Hepatobiliary/Pancreatic Surgical Oncology Program, University Health Network, Toronto, Ontario, Canada; 14 Princess Margaret Cancer Centre, University Health Network, Toronto, Ontario, Canada; 15 Department of Medical Biophysics, University of Toronto, Toronto, Ontario, Canada; 16 Vector Institute, Toronto, Ontario, Canada; Weizmann Institute of Science, ISRAEL

## Abstract

Pancreatic ductal adenocarcinoma (PDAC) has the worst prognosis among solid malignancies and improved therapeutic strategies are needed to improve outcomes. Patient-derived xenografts (PDX) and patient-derived organoids (PDO) serve as promising tools to identify new drugs with therapeutic potential in PDAC. For these preclinical disease models to be effective, they should both recapitulate the molecular heterogeneity of PDAC and validate patient-specific therapeutic sensitivities. To date however, deep characterization of the molecular heterogeneity of PDAC PDX and PDO models and comparison with matched human tumour remains largely unaddressed at the whole genome level. We conducted a comprehensive assessment of the genetic landscape of 16 whole-genome pairs of tumours and matched PDX, from primary PDAC and liver metastasis, including a unique cohort of 5 ‘trios’ of matched primary tumour, PDX, and PDO. We developed a pipeline to score concordance between PDAC models and their paired human tumours for genomic events, including mutations, structural variations, and copy number variations. Tumour-model comparisons of mutations displayed single-gene concordance across major PDAC driver genes, but relatively poor agreement across the greater mutational load. Genome-wide and chromosome-centric analysis of structural variation (SV) events highlights previously unrecognized concordance across chromosomes that demonstrate clustered SV events. We found that polyploidy presented a major challenge when assessing copy number changes; however, ploidy-corrected copy number states suggest good agreement between donor-model pairs. Collectively, our investigations highlight that while PDXs and PDOs may serve as tractable and transplantable systems for probing the molecular properties of PDAC, these models may best serve *selective* analyses across different levels of genomic complexity.

## Author summary

PDAC is a highly lethal, therapy-resistant malignancy, whose etiology is poorly understood. PDAC PDX and PDO have emerged as important *in vitro* and *in vivo* preclinical platforms to study PDAC at the molecular level. However, current investigations of these models provide limited snapshots of donor-model comparisons. To date, comprehensive assessment of PDAC disease models using whole-genome sequencing (WGS) has not been performed. We conducted a comprehensive assessment of the genetic landscape of 16 whole-genome pairs of tumors and matched PDX, from primary PDAC and liver metastasis, including a unique cohort of 5 ‘trios’ of matched primary tumor, PDX, and PDO. Globally, our findings indicate that PDX and PDO successfully recapitulate primary and metastatic disease at the level of simple somatic mutations mainly in driver genes. Disease model fidelity is not fully retained when assessing structural variation (SV) events, based on heterogeneity we observed across donor-model pairs. Strikingly, we found that clustering of large SV events across particular chromosomes is retained when the tumors are implanted into their respective disease models. Polyploidy presents a major challenge when assessing copy number changes; however, ploidy-corrected copy number states suggest good agreement between donor-model pairs. Our work provides biological insights into PDAC disease heterogeneity and presents a tractable pipeline for future WGS research that compares donor samples with matched PDX and PDO.

## Introduction

Pancreatic ductal adenocarcinoma (PDAC) is a highly lethal, therapy-resistant malignancy, with a dismal overall 5-year survival rate that remains minimally unchanged over the past several decades [1, 2]. Multiple failed clinical trials suggest that new approaches are necessary towards understanding PDAC molecular etiology and personalizing treatment [3]. There is continued interest in *in vitro* and *in vivo* preclinical models that emulate the PDAC morphologic and genomic landscape, and which can ultimately serve as platforms to select and test candidate treatments.

An increasing number of experimental findings demonstrate that patient-derived organoids (PDO) and patient-derived xenografts (PDX) function as important preclinical platforms for investigations into the molecular landscape of cancer. Studies on cell lines and PDX have alluded to the agreement of tumour histo-architecture between disease models and primary human PDAC [4, 5]. Huang *et al*. demonstrated that PDAC PDO maintain differentiation status, recreate histo-architectural heterogeneity, and retain patient-specific physiological changes [6]. Recent studies emphasized the fidelity of PDAC disease models at the genomic level by focusing on mutational profiles from whole-exome sequencing (WES) data. Xie *et al* [7], characterized somatic SNVs (single-nucleotide variations) of paired primary tumours and metastases and PDX, focusing on the distribution of allelic frequencies and functional mutations affecting known cancer drivers or tumour suppressors. Witkiewicz et al. and Knudsen et al. [4, 8] compared cell lines and PDX models derived from the same tumour, demonstrating their utility in recapitulating patient-specific therapeutic sensitivities. Collectively, these studies provide valuable insight on the significance of PDAC models as ‘avatars’ for precision treatment, but their singular focus on mutational patterns and morphological changes fails to capture the full spectrum of complex genomic events that underlie PDAC heterogeneity.

Despite progress in sequencing efforts for PDAC, comprehensive assessment of PDAC disease models using whole-genome sequencing (WGS) has not been performed. Using WGS, the genomic complexity of resected PDAC tumours has been thoroughly described [2, 9–12]. WGS analysis of primary and metastatic tumours has also shed light on catastrophic mitotic phenomena, such as chromothripsis, that occur with high frequency in the disease [12]. WGS analysis of PDAC preclinical models would demonstrate how such models recapitulate complex genomic events, including structural variation (SV) and copy number variation (CNV) changes that play a significant role in PDAC tumourigenesis and drug response [13–15].

A large majority of PDAC disease model literature has focused on cell lines and PDX, while genomic characterization of PDO models remains unaddressed. This is despite growing findings that suggest that PDO, compared to PDX and cell lines, may present as models that can reconstitute niches most similar to PDAC [6, 16]. In particular, the 3-D architecture of organoids promotes interaction between pancreatic cells (including normal pancreatic cells, paraneoplastic cells, and neoplastic pancreatic cells) and provides improved conditions for polarization of these epithelial cells [17, 18]. Organoids have also been shown to exhibit ductal- and stage-specific characteristics, and recapitulate the full spectrum of PDAC tumourigenesis [16]. These promising findings pose an opportunity for probing such PDOs at the genomic level. Genomic analysis of PDOs remains a missing link to identify whether these models recapitulate patient tumours at the molecular level, a necessary step before widespread screening therapeutics.

In this study, we conducted a detailed assessment of the genetic landscape of a series of paired tumours and PDX from primary PDAC and liver metastases, including WGS data from a unique set of 5 matched ‘trios’ of primary tumour, PDX, and PDO. Globally, our findings indicate that PDX and PDO successfully recapitulate primary and metastatic disease at the level of simple somatic mutations mainly in driver genes, but not across the greater expanse of annotated mutations. Disease model fidelity is not readily retained when assessing structural variation (SV) events, based on heterogeneity we observed across donor-model pairs. However, our results indicate that clustering of large SV events across particular chromosomes of the tumours is retained in matching disease models. Our work demonstrates stable consistency of *ploidy-corrected* copy number states between donor-model pairs, but underscores a major concern regarding ploidy changes when tumours are implanted into their respective models. Our results provide new insights into the interplay and fidelity of different disease models toward recapitulating PDAC heterogeneity and genomics.

## Results

**Fig 1:**
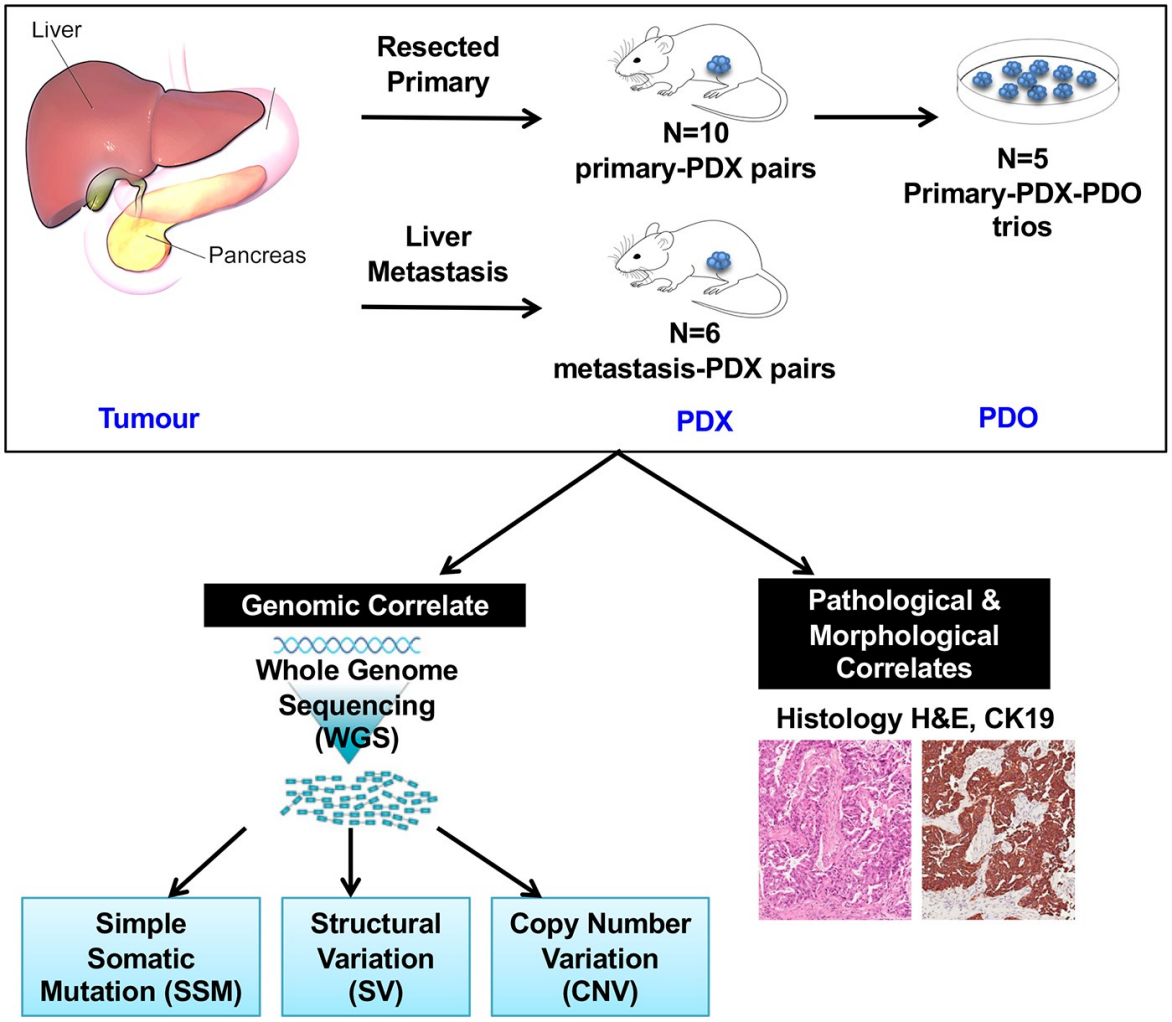
Schematic overview of samples and analysis. Disease models (PDX and PDO) were compared against matched human tumours in terms of morphological agreement, and genomic agreement. Assessment of genomic agreement was established by a top-down approach that determined genomic changes at varying levels of complexity, spanning single-based resolution (SSM) towards genome-wide comparisons (CNV).

We conducted comprehensive characterization of paired PDX and PDO from PDAC primary tumours and liver metastases [Fig 1, S1 Table, S1 Fig]. WGS was performed for 16 pairs of tumours with their matched disease models [S1 Table, S1 Fig]. These included a series of 10 resected tumours and 6 liver metastases [S1 Table]. Of the primary tumours, five samples also had a PDO derived from the PDX, comprising a unique cohort of matched tumour-PDX-PDO, hereby referred to as ‘trios’ throughout the paper. The PDOs were derived from the PDX tissue, as opposed to the primary patient material.

### Histology

**Fig 2:**
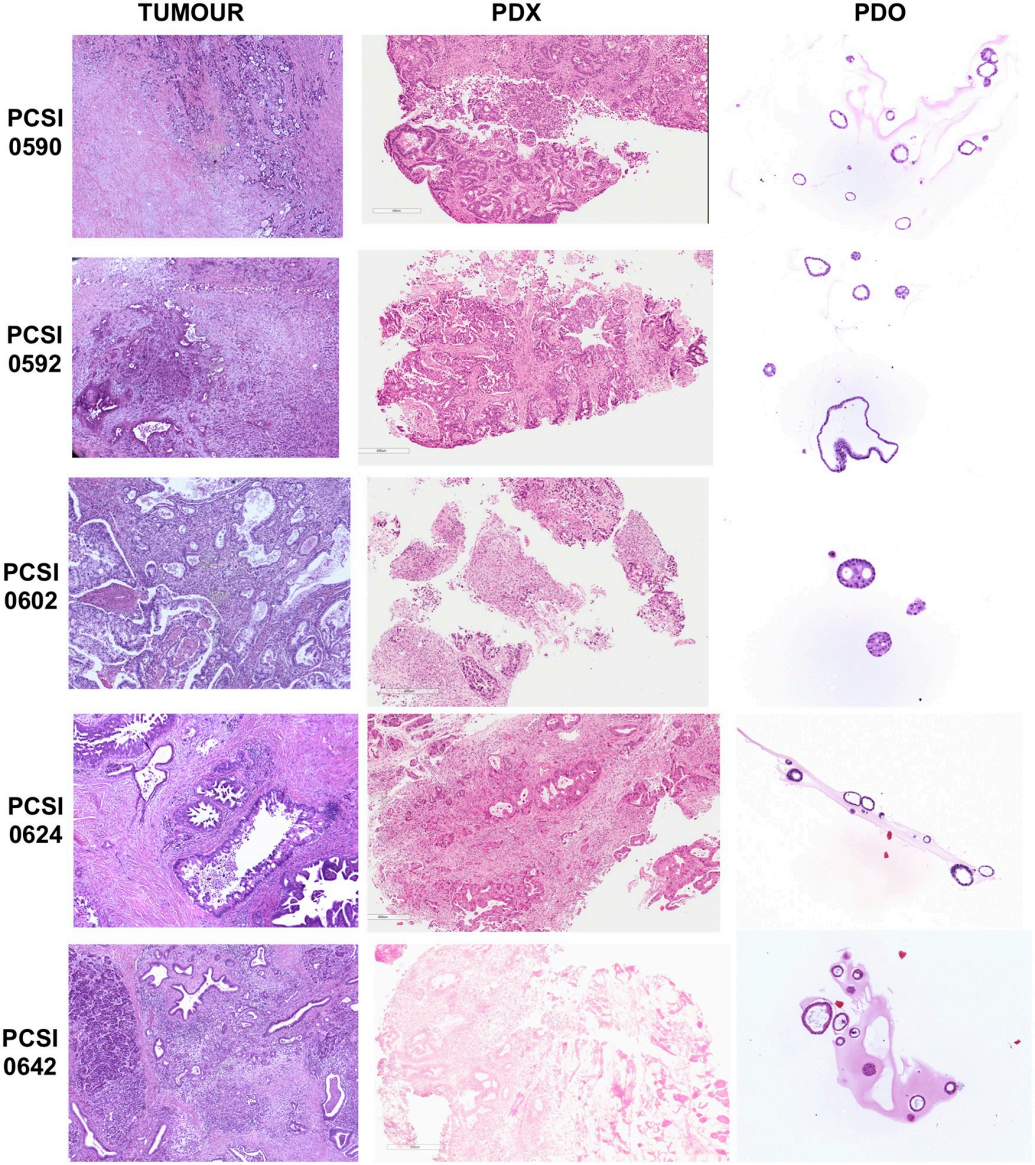
Representative H&E stained sections of primary tumour, matched PDX, and PDO in 5 trios. PDX images are shown at 5.6X zoom, with a scale of 400 uM. PDO images are shown at 10X zoom.

We assessed the conservation of tumour histo-architecture in the trios [Fig 2]. All samples had strong staining of cytokeratin 19 (CK19) [S2 Fig, S2 Table], confirming that all PDX and PDO samples consisted of human PDAC. All samples demonstrated a mainly tubular architecture with varying degrees of cellularity and stroma content [S2 Table]. PDOs were also observed to mimic the histo-architectural heterogeneity of their matched tumours, and were comprised of a hollow central lumen surrounded by one layer of polarized epithelia [Fig 2, S2 Fig].

### Genomic profile—SSM, SV, and CNV changes of PDAC driver genes

**Fig 3:**
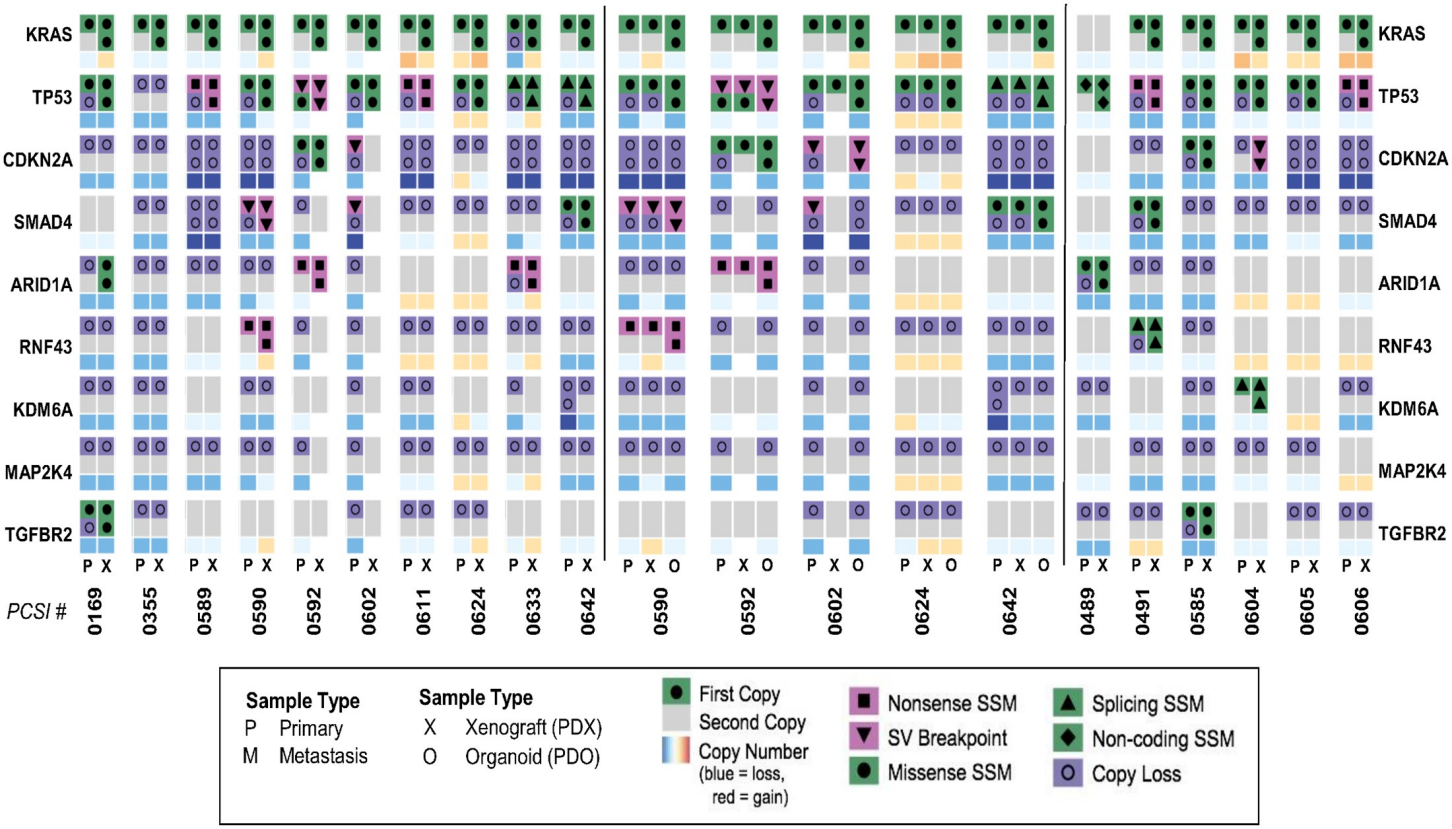
Comparative analysis of SSM, SV, and CNV across genes of PDAC tumourigenesis and disease therapy. The genomic profile of driver genes across primary-PDX pairs (left), metastasis-PDX pairs (left), and primary-PDX-PDO trios (middle) is shown.

We compared the genomic profiles of 9 PDAC driver genes, including oncogenes and tumour suppressor genes, in primaries and metastases, and in their matched disease models [Fig 3]. We annotated SSM, SV breakpoints, and copy number changes associated with these genes in tumour-PDX pairs, and in tumour-PDX-PDO trios.

Several structural arrangements observed in the resected tumours were recapitulated in the corresponding PDX, including deletion events and copy number loss (eg: MAP2K4 and TP53 across 80% of the primary-PDX pairs) [Fig 3]. Homozygous deletions of CKDN2A were observed in several primary-PDX pairs (ex: PCSI_0590, PCSI_0642) and recapitulated in their corresponding trios [Fig 3]. There were cases of copy number discordance between primary-PDX pairs for some genes (ex: RNF43 in PCSI_0592, PCSI_0602 and KDM6A in PCSI_0592, PCSI_0602, PCSI_0633) which also extended to the trios; in the case of the trios, copy loss was observed in the PDO, even though it has not been observed in the PDX from which it was derived [Fig 3].

Structural rearrangements and copy state changes demonstrated consistency across the majority of the metastasis-PDX pairs and the 9 genes assessed. Exceptional cases of discordance were mainly found in KRAS in PCSI_0585, and CDKN2A in PCSI_0604 [Fig 3].

### Simple somatic mutation

**Fig 4:**
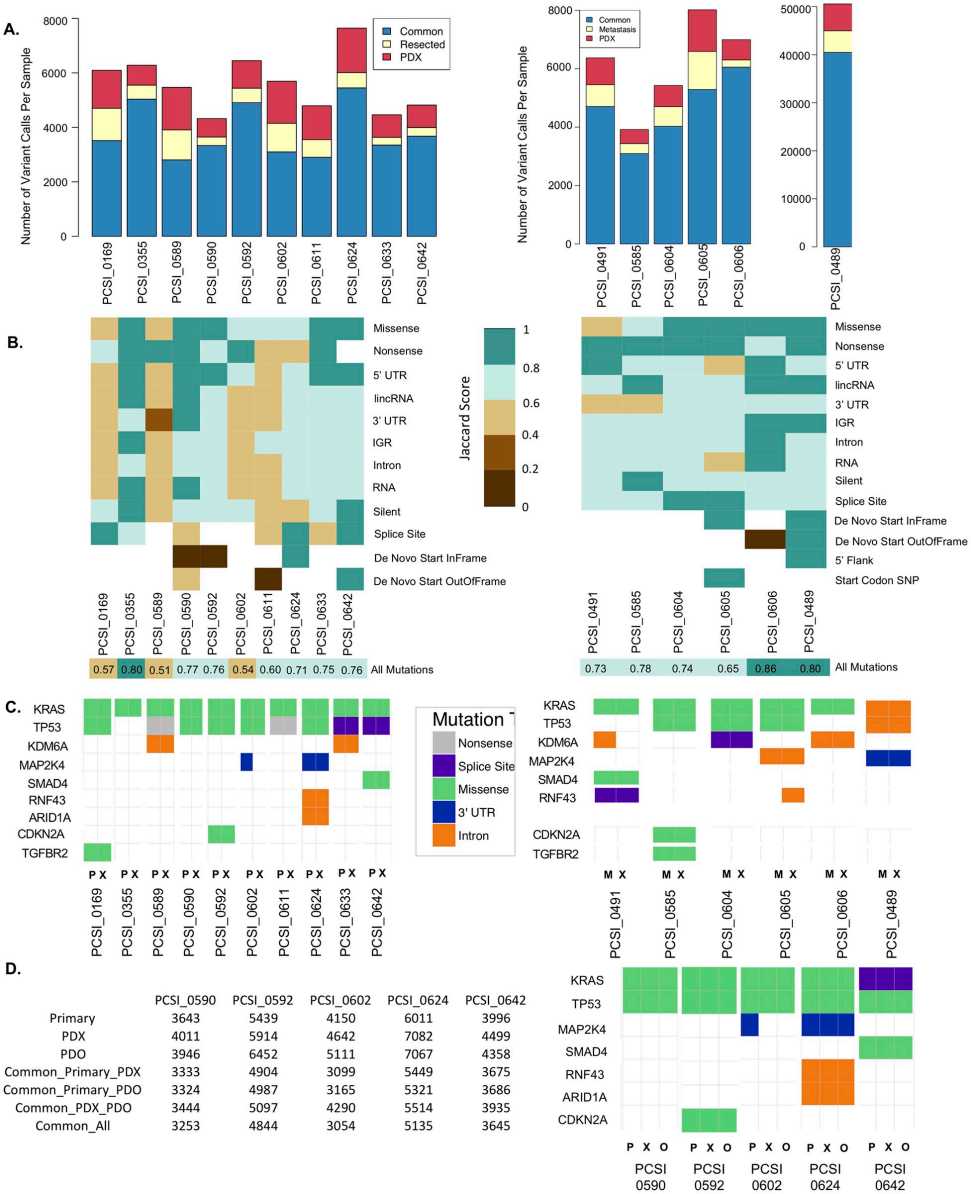
Comparative analysis of SSM across tumour-PDX pairs of resected primary (left) and liver metastasis (right). **(A)** Total number of variant calls across matched tumour-PDX pairs for 10 primary samples (left) and 6 metastasis samples (right). The total number of common mutations across a given pair is indicated, as well as variants that are specific to the tumour sample or matching PDX. **(B)** Heatmap representation of the Jaccard index for a given tumour-PDX pair, across all categories of functional and non-functional mutation types annotated in the primary samples (left) and metastasis samples (right). White cells indicate mutation types that are not available for a tumour-PDX pair. Overall concordance of a tumour-PDX pair across all mutations is indicated by the Jaccard index in the last row (“All Mutations”). **(C)** Conservation of mutation types across oncogene and tumour suppressor genes in the primary samples (left) and metastases (right). Samples are labeled as primary (P), xenograft (X), and metastasis (M). **(D)** Total number of SSM calls across primary-PDX-PDO trios. The top rows indicate the total number of mutations observed in each for the primary, PDX, and PDO samples. Common mutations across primary-PDX, primary-PDO, and PDX-PDO pairs also indicated. The total number of common mutations shared across all samples of the trio is delineated in the last row. Conservation of mutation types across oncogenes and tumour suppressor genes in the trios is also indicated (right). Samples are labeled as primary (P), xenograft (X), and organoid (O).

We conducted a comparative analysis of SSM events in tumour-PDX primaries, trios, and paired tumour-PDX metastases, to assess preservation of somatic variants between disease models and their source tumours [Fig 4A, S3(A)–S3(E) Table]. An average of 4,500 and 5,000 mutations were identified in the resected tumours and in their matched PDX, respectively [Fig 4A, S3(A) Table]. More than 50% of the mutations observed in the primary tumour were retained in the paired PDX [Fig 4A]. Considering all mutations, 70% of the pairs exhibited a Jaccard index ≥ 0.6 [Fig 4B, S3(D) Table], with PCSI_0355 scoring the highest (Jaccard index = 0.8). Jaccard scores of the remaining 3 pairs (PCSI_0169, PCSI_0589, PCSI_0602) ranged from 0.51 to 0.57. To assess conservation or diversity across functional and non-functional mutations, including synonymous and non-synonymous events (SNVs), we calculated the Jaccard index for each tumour-PDX pair for 12 mutation types observed in the samples [Fig 4B]. Eighty percent of the pairs exhibited an SNV Jaccard score ≥ 0.6 for functional mutations (missense, nonsense). Some individual pairs exhibited Jaccard scores ≥ 0.8 for missense mutations (PCSI 0355, 0590, 0592, 0633, 0642), and similarly for nonsense mutations (PCSI 0355, 0589, 0590, 0602, 0633). Pairwise-comparison of mutation categories also highlighted other mutation types, including mutations of lincRNA, for which the majority of pairs (>60% of the pairs) had a Jaccard score ≥ 0.6.

We conducted an in-depth analysis of mutation patterns for driver genes of PDAC tumourigenesis, including PDAC oncogenes (KRAS) and tumour suppressor genes (CDKN2A, TP53, SMAD4, TGFB2) [Fig 4C]. In cases of apparent discordance between tumour-PDX pairs for a given mutation, we conducted additional manual inspection of the variants to ensure that the failure to call the variant was not attributed to insufficient coverage or statistical threshold for variant calling. Despite discrepancies observed between tumour-PDX pairs across different mutation types, SNVs across the main driver genes remain conserved. KRAS and TP53 mutations were observed in 90% of the pairs, and harbored the same functional consequences (mostly missense mutations) in matched tumour-PDX samples [Fig 4C]. Detailed examination of the KRAS mutations calls in the primary tumour series revealed the presence of G12D, G12R, and G12V oncogenic mutations, with the majority of missense mutations belonging to G12D. The majority of TP53 mutations were missense mutations, but also included splice-site mutations in 2 pairs of the series [Fig 4C]. We assessed the frequency of reads carrying the variant alleles across tumour-PDX pairs. Comparable frequencies between tumours and matched PDX were also observed in larger sets of variants from genes encompassing oncogenes, tumour suppressor genes, and frequently mutated genes involved in PDAC tumourigenesis [S3(A) Fig].

Comparison of liver metastasis pairs [Fig 4, **right column]** recapitulated much of the somatic mutational landscape observed in the primary tumours. Across 5 liver metastasis samples, we observed averages of ~5,300 mutations per tumour, and ~5,500 mutations per PDX, with a range of 66–86% overlap of mutation calls in metastasis-PDX pairs [Fig 4A, S3(B) Table]. One sample (PCSI_0489) had a strikingly high mutation load, with an average of ~45,500 mutations in both the metastasis sample and the matched PDX, due to DNA mismatch-repair (MMR) deficiency. This sample had a germline frameshift MLH1 deletion, loss of heterozygosity of MLH1 on the p-arm of chromosome 3 and elevated C>T transitions, which corresponds with the diagnosis of Lynch syndrome in the patient [19]. All of the metastasis-PDX pairs, including the MMR deficient case, demonstrated a Jaccard index ≥ 0.6 across all mutations considered [Fig 4B, S3(E) Table], with PCSI_0606 scoring the highest (Jaccard index = 0.86). All of the pairs exhibited an SNV Jaccard score ≥ 0.6 for functional mutations (missense, nonsense), with the exception of the MMR case [Fig 4B]. Individually, 4/6 pairs had Jaccard scores ≥ 0.8 for missense mutations, and 5/6 pairs had Jaccard scores ≥ 0.8 for nonsense mutations [Fig 4B]. Missense mutations for KRAS and TP53 were conserved in the majority of the metastasis-PDX pairs; for the MMR case only non-functional variants for those genes were observed [Fig 4C]. Frequencies of reads carrying the variant allele were also comparable between liver metastasis samples and their matched PDX [S3(B) Fig].

Matched PDO samples demonstrated the same mutation pattern in oncogenic driver and tumour suppressor genes as that of their matched PDX and tumour [Fig 4D, S3(C) Table]. Pairwise comparisons across the tumours and the models highlighted overall consistency of read frequencies carrying the variant alleles in tumour-PDX, tumour-PDO, and PDX-PDO samples for each of the trios [S3(C) Fig].

### Structural variation

**Fig 5:**
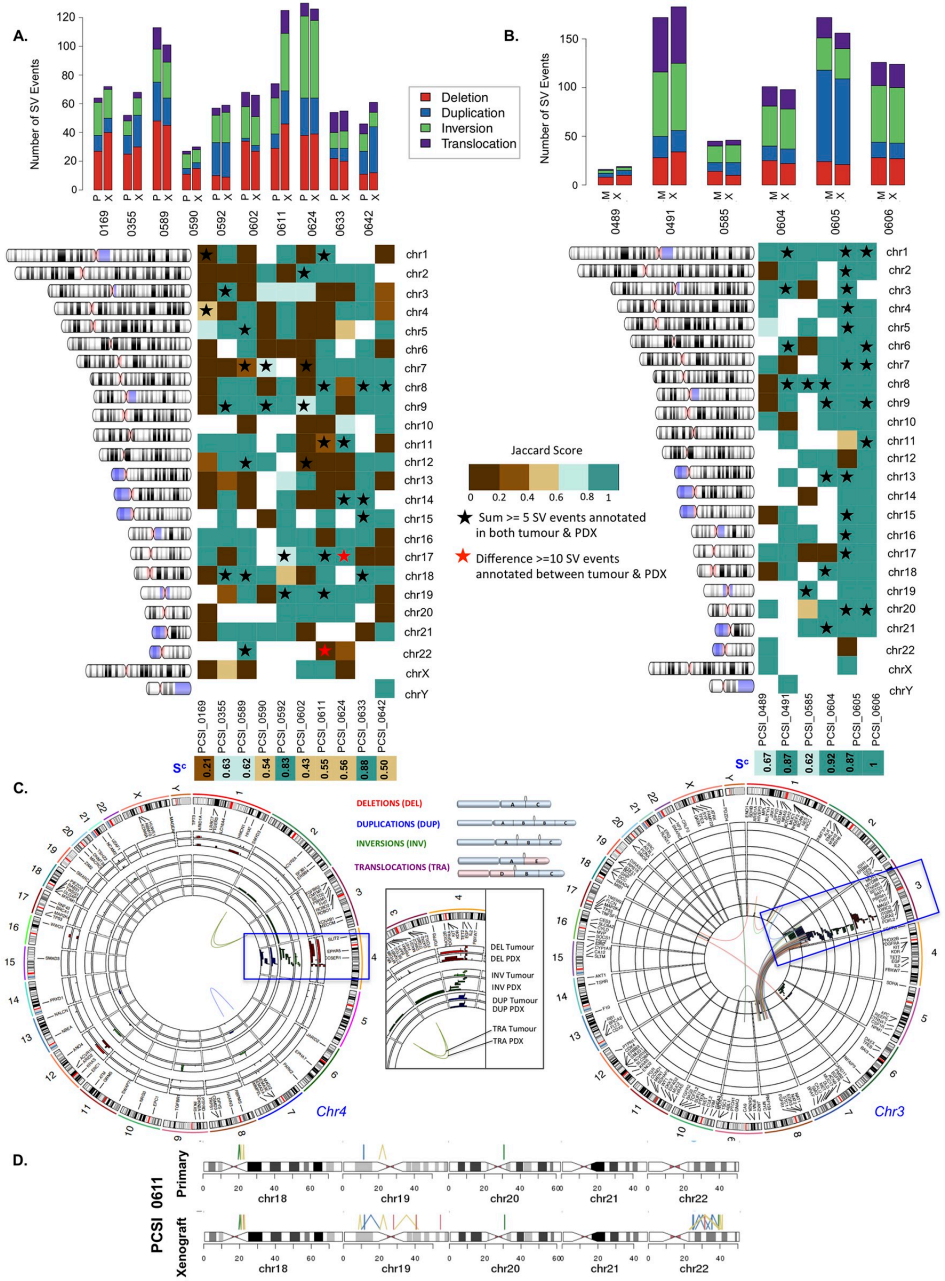
Comparative analysis of structural variation (SV) across tumour-PDX pairs of the primary and metastasis cohorts. **(A)** Distribution of SV events (deletion, duplication, inversion, translocation) in each sample across 10 primary-PDX pairs (left) and 6 metastasis-PDX pairs (right). **(B)** Chromosome-specific Jaccard indices across 10 primary-PDX pairs (left) and 6 metastasis-PDX pairs (right). Samples are labeled by their PCSI identifier. Chromosomes with an observed large number of rearrangements (≥5 events) in both the tumour and matched PDX are indicated (black stars). Chromosomes with a large difference of SV events between a tumour-PDX pair (≥10 SV events difference) are highlighted (red stars). The overall concordance (S^c^) score for a tumour-PDX pair across all chromosomes in indicated (bottom row). **(C)** Genome-wide SV events observed in tumour-PDX pairs in PCSI_0169 (resected primary, left) and PCSI_0491 (liver metastasis, right). Each type of SV event is color-coded with a similar color between tumours and matching PDX. For each SV type, tumours are annotated on the outer rings of the circos plot and the matching PDX on the inner rings. Chromosomes exhibiting clustered SV events (potential chromothripsis) are highlighted in the blue boxes. **(D)** Comparison of SV events across chr18-chr22 for the PCSI_0611 primary tumour and its matching PDX. There is an apparent chromothripsis event on chr22 of the PDX but not the primary sample.

We used our WGS data to assess structural variation (chromosomal rearrangements). Analysis of SVs in the resected primary-PDX pairs [Fig 5, **left column]** revealed that the majority of variants were intra-chromosomal, including deletions (DEL), inversions (INV), and duplications (DUP) [Fig 5A, S4(A) Table]. Inter-chromosomal translocations (TRA) were less prevalent [Fig 5A, S4(A) Table]. The total numbers of SV events observed were similar between primary-PDX pairs with the exception of PCSI_0611, where the number of events in the PDX were almost double that observed in the primary tumour [Fig 5A].

We compared the distribution of structural variation events across each chromosome, for every primary-PDX pair [Fig 5B, S5(A) Table]. For each pair assessed, we observed a disparity of SV events across the majority of the chromosomes (Jaccard index ≤ 0.4) [Fig 5B, S6(A) Table]. We computed an overall concordance (S^c^; see Methods) score for each primary-PDX pair, to quantify concordance across all chromosomes that harbor SV events [Fig 5B]. Only 40% of the pairs had an overall concordance S^c^ ≥ 0.6, including highly-scoring PCSI_0633 (S^c^ = 0.88) and PCSI_0592 (S^c^ = 0.83). PCSI_0169 was the least concordant across the genome (S^c^ = 0.21) [Fig 5B]. Upon further investigation, we found that the PDX has more than twice as many indels as the primary tumour, and is enriched for deletions, which suggests that mutations may be accumulating due to deficiency in a DNA repair pathway.

Jaccard scores (scores ≥ 0.8) were observed for many chromosomes with elevated numbers of SV events (defined as ≥5 SV events in both the primary and its matching PDX) [Fig 5B, S5(A) and S6(A) Tables]. This concordance extended to chromosomes that displayed clusters of structural variants in the primary-PDX pair. Elevated counts of clustered chromosomal rearrangements in these chromosomes, compared to the rest of the genome, is suggestive of chromothripsis [Fig 5C, S5(A) Table]. In the majority of the primary-PDX pairs, we identified particular chromosomes with clustered SV events, including PCSI_0169 (chr 4), PCSI_0589 (chr 7), PCSI_0592 (chr 17, chr 18), PCSI_0611 (chr1), PCSI_0624 (chr17), and PCSI_0642 (chr 8) [Fig 5B and 5C, S4(A) Fig, S5(A) Table]. We also identified chromosomes that were strongly discordant, owing to a large difference in the number of SV events between the primary and matched PDX. Most notable of these was the q arm of chromosome 22 in PCSI_0611, for which a cluster of SV events was observed in the PDX but not in the primary sample [Fig 5D, S5(A) and S6(A) Tables].

We computed both genome-wide and chromosome-centric scores across different categories of SV events, to identify cases of agreement or disparity in the distribution of SV breakpoints [S5 Fig, S7(A) Table]. For low-scoring samples (S^c^ ˂ 0.6), there was demonstrable variability between tumour-PDX pairs across deletions, inversions, duplications, and translocations [S5(A) Fig]. We subsequently focused on chromosomes demonstrating chromothripsis to identify whether rearrangements of SV events in these chromosomes are typically different [S5(B) Fig, S7(B) Table]. Clustering of SV events in the tumours were typically different from the matched PDX, with Jaccard scores ranging between 0–1 across all four SV categories assessed. PCSI_0611 (chr1) and PCSI_0642 (chr8) demonstrated greater consistency of SV breakpoints across the tumour-PDX pairs, such that Jaccard scores almost equated to 1 for all intra-chromosomal variants (deletions, duplications, inversions) [S5(B) Fig, S7(B) Table].

We conducted a comparative analysis of SV events in paired metastasis and matched PDX samples [Fig 5, **right column]** to identify distinct chromosomal instability profiles observed in primary tumours that could also extend to liver metastasis pairs. Metastasis-PDX pairs exhibited variable counts of SV events, ranging between 16–172 events in the samples [Fig 5B, S4(B) Table]. Not surprisingly, the MMR deficient case (PCSI_0489) had the fewest number of SV events in the pairs [Fig 5B, S4(B) Table], and scored moderately (S^c^ = 0.6). In contrast to the primary-PDX pairs however, the majority of the pairs (4/6) demonstrated greater agreement across the genome (S^c^ > 0.8 for PCSI_0491, PCSI_0604, PCSI_0605, and PCSI_0606). This agreement was also reflected when assessing different categories of SV events across the genome [S5(C) Fig, S7(C) Table].

Chromosome-centric analyses of metastasis-PDX pairs indicated consistency of SV events across the majority of the chromosomes (Jaccard index ≥ 0.6) [Fig 5B, S5(B) and S6(B) Tables]. Jaccard scores ≥ 0.8 were observed for chromosomes with elevated counts of SV events in both the metastasis and matched PDX [Fig 5B, S5(B) and S6(B) Tables]. We identified clustering of SV events in specific chromosomes for the metastasis-PDX pairs, with the exception of the MMR deficient case [Fig 5C, S4(B) Fig]. These chromosomes include PCSI_0491 (chr 3), PCSI_0604 (chr 13), PCSI_0605 (chr1, chr2), and PCSI_0606 (chr1, chr9, chr11) [Fig 5C, S4(B) Fig, S5(B) and S6(B) Tables]. Analysis of rearrangement of SV events, for these chromosomes, indicates that the clustering of SV events in the metastasis and matched PDX are typically similar, or almost identical, for the majority of the cases, with Jaccard scores ~1 for SV categories in 5 of the 7 chromosomes assessed [S5(C) and S5(D) Fig, S7(C) and S7(D) Table]. The most variable clustering of SV events was observed in PCSI_0605 (chr1) and PCSI_0606 (chr 11) [S5(D) Fig, S7(D) Table].

**Fig 6:**
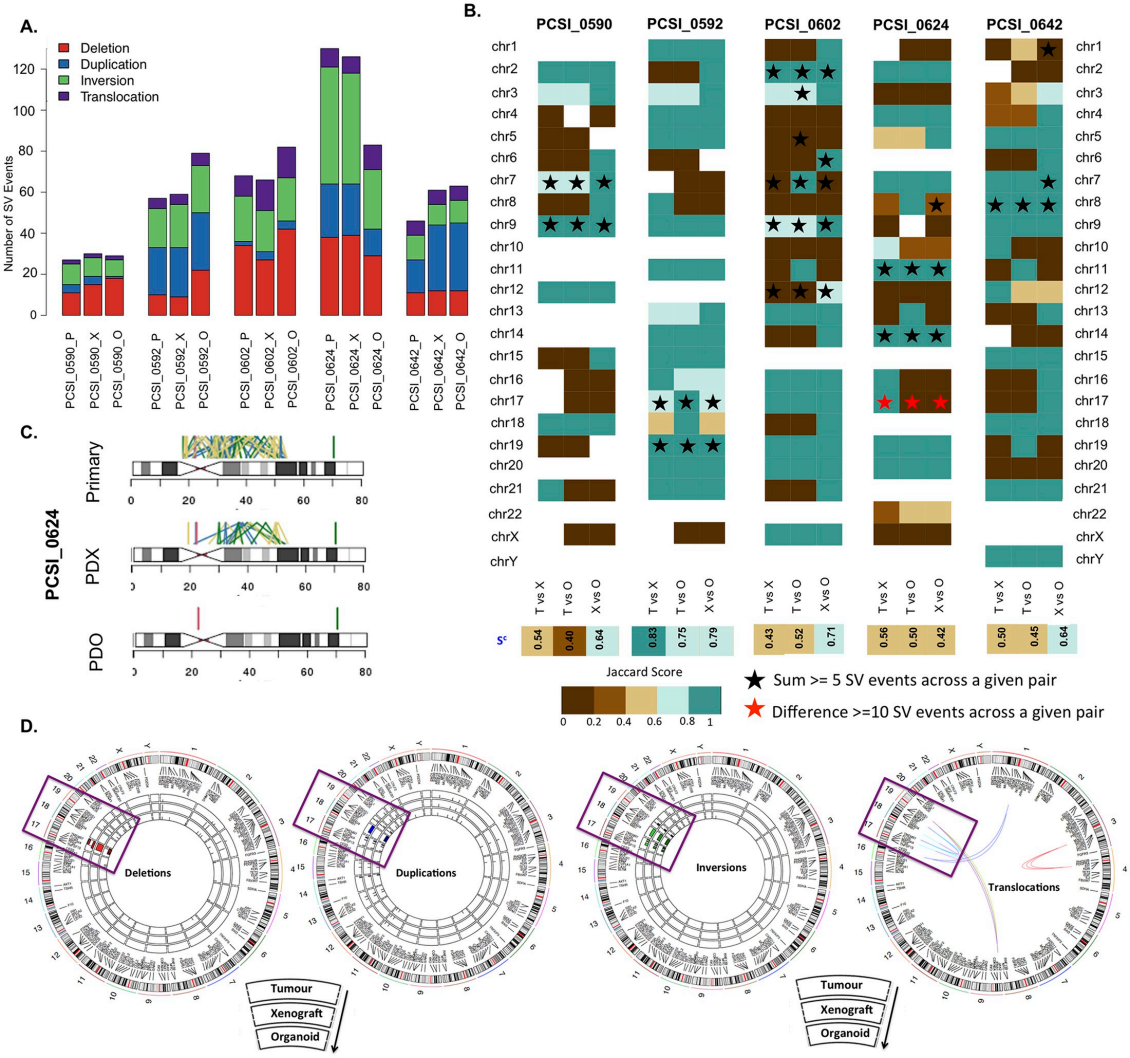
Comparative analysis of structural variation (SV) across tumour-PDX-PDO trios. **(A)** Distribution of SV events (deletion, duplication, inversion, translocation) in 5 trio samples. The primary (P), PDX (X), and PDO (O) sample for each trio is indicated. **B)** Chromosome-specific Jaccard indices in 5 primary-PDX-PDO trios. Each trio is split into 3 pairs representing primary-PDX (T vs X), primary-PDO (T vs O) and PDX-PDO (X vs O) comparisons. Chromosomes with an observed large number of rearrangements (≥5 events) in each pair are indicated (black stars). Chromosomes with a large difference of SV events between a tumour-PDX pair (≥10 SV events difference) are highlighted (red stars). The overall concordance (S^c^) score for each pair across all chromosomes in indicated (bottom row). **(C)** Comparison of SV events across chromosome 17 of PCSI_0624. This chromosome was discordant between primary-PDX, primary-PDO, and PDX-PDO pairs. **(D)** Distribution of SV events across the primary tumour, matched PDX, and matched PDO samples of the PCSI_0592 trio. Each type of SV (deletion, inversion, duplication, and translocation) is represented as one circos plot, with 3 rings indicating tumour (outer), PDX (middle), and PDO (inner). Chromosomes demonstrating chromothripsis are highlighted in boxes.

Analysis of the primary-PDX-PDO trios highlighted the extent to which structural variation of primary-PDX pairs were captured in organoid models [Fig 6, S4(C) and S5(C) Tables, S6(C) Fig]. The distribution of intra-chromosomal and inter-chromosomal events in the PDO samples shares a similar pattern to that of their matched tumour and PDX [Fig 6A, S4(C) Table]. By splitting the trio into matched pairs (primary-PDX, primary-PDO, and PDX-PDO), we assessed concordance in the primary tumours and each of the disease models (tumour-PDX, tumour-PDO pairs), and the interrelationship between the disease models themselves (PDX-PDO pairs) [Fig 6B, S5(C) and S6(C) Tables]. PCSI_0592 was the highest scoring trio, across tumour-pdx, tumour-PDO, and PDX-PDO comparisons [Fig 6B]. Tumour-PDX and tumour-PDO comparisons indicated lower agreement across the genome (S^c^ <0.6), and across many chromosomes (Jaccard scores ≤0.4) for the remaining 4 trios [Fig 6B, S6(C) Table]. Interestingly, S^c^ scores of PDX-PDO comparisons scored higher than tumour-PDX and tumour-PDO comparisons for these pairs [Fig 6B]. Despite inter-model variability observed at both genome-wide and chromosome-centric levels, there is however a stronger agreement of SV events in the trios, for chromosomes with highly clustered SV events. This concordance (S^c^ ≥ 0.8) was observed across all tumour-PDX, tumour-PDO, and PDX-PDO pairs for several chromosomes, including PCSI_0592 (chr19), PCSI_0624 (chr11, chr14), PCSI_0642 (chr8) [Fig 6B and 6D, S6(C) Table].

Comparison of tumour-PDX, tumour-PDO, and PDX-PDO pairs highlighted particular cases that were discordant at both chromosome and genome-wide levels. We observed the most discordance in all specimens of the trio for chromosome 17 of PCSI_0624. Further investigation into this chromosome revealed a very high count of SV events in the primary tumour, with reduced events in the matching PDX, and almost no events in the PDO [Fig 6C, S5(C) Table]. Comparison of the trios genome-wide (using overall S^c^ scores) also demonstrates that PCSI_0590 and PCSI _0624 were the lowest scoring among the trios; all of the pairs of those trios demonstrated overall concordance S^c^ < 0.6.

### Copy number variation

**Fig 7:**
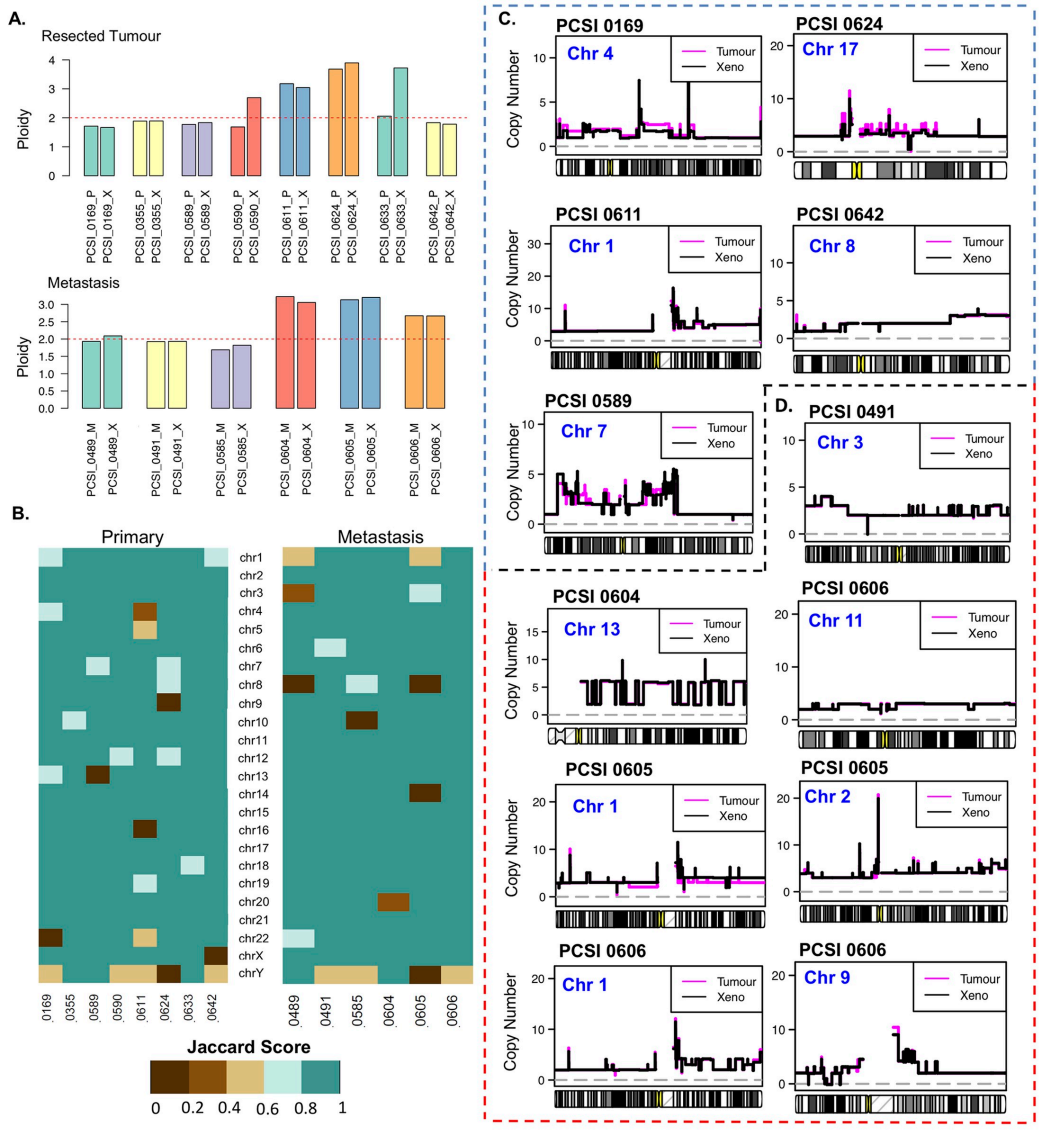
Comparative analysis of copy number state across tumour-PDX pairs of the resected primary cohort. **(A)** Ploidy across tumour-PDX pairs for primary tumours (top) and liver metastasis (bottom). **(B)** Chromosome-specific Jaccard indices across 10 primary-PDX pairs (left) and 6 metastasis-PDX pairs (right). **(C)** Copy number state (uncorrected) of primary tumour (magenta) and matching PDX (black) in primary-PDX pairs, highlighted for chromosomes for which chromothripsis had been observed. **(D)** Copy number state (uncorrected) of liver metastases (magenta) and matching PDX (black) in metastasis-PDX pairs, highlighted for chromosomes for which chromothripsis had been observed.

**Fig 8:**
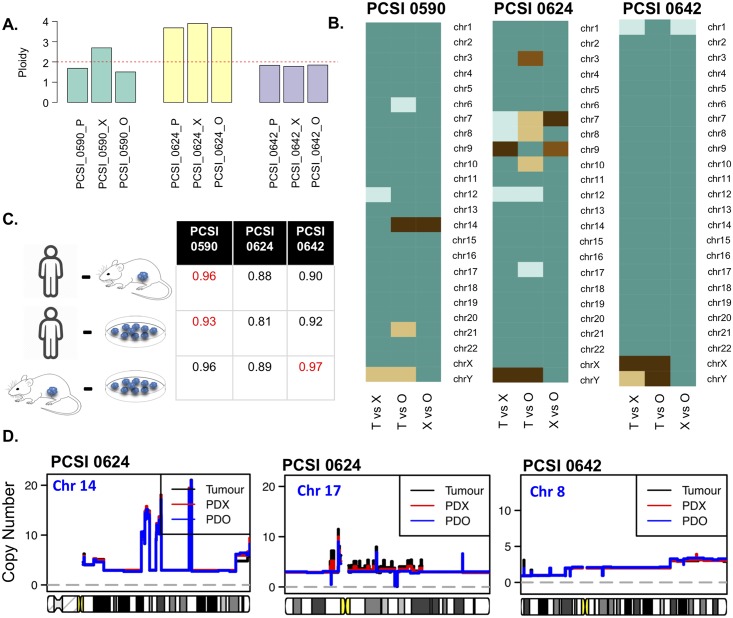
Comparative analysis of copy number state across tumour-PDX-PDO trios of the resected primary cohort. **(A)** Ploidy across primary-PDX-PDO trios. **(B)** Chromosome-specific Jaccard indices across 3 primary-PDX-PDO trios (PCSI_0590, PCSI_0624, and PCSI_0642). Each trio is split into 3 pairs representing primary-PDX (T vs X), primary-PDO (T vs O) and PDX-PDO (X vs O) comparisons. **(C)** Genome-wide concordance score for primary-PDX, primary-PDO, and PDX-PDO pairs for 3 trios. The highest scoring sample for across tumour-PDX, tumour-PDO, and PDX-PDO pairs is highlighted in red. **(D)** Copy number state (uncorrected) of primary tumour (black), matching PDX (red), and matching PDO (blue), highlighted for chromosomes for which chromothripsis had been observed.

Genome-wide copy number state was assessed for primary-PDX pairs, trios, and metastasis-PDX pairs [Figs 7 and 8, S8 and S9 Tables]. Evaluation of ploidy could not be determined unambiguously for two of the samples (PCSI_0592 and PCSI_0602) and they were excluded from the copy number analysis. Accordingly, 8/10 primary-PDX pairs were further evaluated for ploidy and copy number state [S8(A) Table, S7 Fig]. Both tumours and their matched PDX exhibited comparable ploidy in the majority of the pairs [Fig 7A, S8(A) Table]. Two pairs (PCSI_0590 and PCSI_0633) demonstrated a doubling in ploidy in the PDX, compared to the matching primary tumour [Fig 7A, S8(A) Table, S8 Fig].

Jaccard indices were computed to quantify overall similarity between the ploidy-adjusted copy number states of primary tumours and matching PDX, across all bases of the genome, and for individual chromosomes. Using ploidy-normalized scores, all tumour-PDX pairs demonstrated concordance ≥ 0.8 across the entire genome [Fig 7B, S9(A) Table]. Chromosome-centric analyses indicated discordance for individual chromosomes, with some somatic chromosomes scoring ≤ 0.4 in the tumour-PDX pairs [Fig 7B, S9(A) Table]. Notably, chromosomal concordance ≥ 0.8 was observed for all chromosomes that were previously annotated with highly clustered SV events [Fig 7B]. Comparison of the raw (uncorrected) copy number values identifies cases of concordance that support the chromosomal score calculations (ex: PCSI_0611 chr 1, PCSI_0642 chr 8), but also highlights discrepancies in several of these chromosomes (ex: PCSI_0169 chr 4, PCSI_0624 chr 17) whose effect may have been reduced in the ploidy-adjusted copy number states [Fig 7B and 7C, S8 Fig].

Assessment of copy number variation in the liver metastasis set demonstrated comparable ploidy states across the 6 metastasis-PDX pairs, and none of those pairs had to be excluded from the analysis [Fig 7A, S8(B) Table, S7 Fig]. Using ploidy-normalized scores, all metastasis-PDX pairs demonstrated concordance ≥ 0.8 across the entire genome [Fig 7B, S9(B) Table, S8 Fig]. The MMR deficient sample (PCSI_0489) did not exhibit major disruptions in copy number across the genome [Fig 7A, S8 Fig]. Chromosome-centric analyses identified some cases of discordance for individual chromosomes, with some somatic chromosomes scoring ≤ 0.4 in the metastasis-PDX pairs [Fig 7B, S9(B) Table]. Chromosomal concordance ≥ 0.8 was observed for all chromosomes that were previously annotated with highly clustered SV events, with the exception of PCSI_0605 chr1 [Fig 7B]. This agreement of ploidy-corrected copy number states between metastasis and PDX is observed even when comparing raw (uncorrected) copy number measurements [Fig 7D, S8 Fig].

Copy number state was compared within tumour-PDX, tumour-PDO, and PDX-PDO matched pairs for trios [Fig 8, S8(C) and S9(C) Tables, S9 and S10 Figs]. As PCSI_0592 and PCSI_0602 were excluded due to ploidy estimation problems, only 3/5 trios were available for further analysis [Fig 8A, S8(C) Table, S9 Fig]. PDO samples exhibited similar ploidy to their matched primary tumour and PDX [Fig 8A, S8(C) Table]. There was an overall consistency of ploidy between tumours, PDX, and PDO trios, with the exception of PCSI_0590, which exhibited disparate ploidy in the PDX as previously described [Fig 8A, S9 Fig]. Pairwise-comparisons (tumour-PDX, tumour-PDO, and PDX-PDO) of ploidy-adjusted values highlighted strong concordance of copy number (concordance rate ≥ 0.8) in all the pairs at a genome-wide level, and almost no instances of disparity for somatic chromosomes, even for chromosomes that had been identified as cases of chromothripsis [Fig 8B and 8C, S9(C) Table]. Assessment of raw (unadjusted) copy number measurements, for chromosomes with chromothripsis reveals that PDO copy number changes are generally in agreement with the primary tumour and PDX, with the exception of PCSI_0624 (chr17) [Fig 8D, S10 Fig].

### Clonality across tumours and disease models

We tested for clonality of primary tumours, metastases, and matching models using PyClone [S11 Fig]. PyClone was tested only on SNVs that were called in all of the samples derived from a patient (i.e., all SNVs found in tumours and PDX in the pairs, and all SNVs found in tumours and PDX and PDO in the trios). Based on our sample size and lack of technical replicates, we cannot conclude that metastases are more clonal that the primary tumours based on the current analysis. On average, there are between 2–4 SNV cluster lines attributed to each patient sample (and matched models), whether for primary tumours or metastatic ones. Overall, for many of the resected PDAC cases and liver metastases, clonality of SNVs are preserved across the different samples belonging to a patient; SNV cluster lines are approximately horizontal as the cellular prevalence of the SNVs remain similar across the samples (for example: PCSI_0169, PCSI_0355, PCSI_0611, PCSI_0624 in PDAC, and PCSI_0605 and PCSI_0606 in metastases) [S11 Fig]. Notable exceptions include PCSI_0589 and PCSI_0602 in the resected primary patients [S11(A) Fig], where the cellular prevalence of some SNV clusters is higher in the PDX and PDO, compared to the primary sample tumour. PCSI_0489 of the liver metastases [S11(B) Fig] both had the largest number of clusters and the greatest variation in cellular prevalence across several clusters, corresponding to our observations of mismatch repair (MMR) for that sample.

## Discussion

Critical evaluation of disease models is gaining importance across several cancer types that utilize these surrogates as preclinical tools for exploring tumourigenesis and drug testing. Previous investigations of PDAC provide a limited snapshot of donor-model comparisons, in terms of morphological, pathological, and mutational correlates [S12 Fig]. Our histologic analysis demonstrated that PDXs and PDOs retain the main characteristics of the tumour samples from which they were derived, in agreement with previous work [4, 6, 8]. It can be argued however, that sampling of multiple sites from donors and disease models can identify a spectrum of histologic and morphologic patterns that would render tumours and their matching models as dissimilar. Accordingly, donor-model comparisons of particularly heterogeneous tumours, including PDAC, will benefit from the advent of next-generation sequencing technologies that can encapsulate all facets of the disease at the molecular level. We present, to our knowledge, the first quantitative assessment of whole-genome comparisons between human PDAC (both primary and metastasis) and matched model systems. We evaluated single base-pair genomic events (SSM), larger chromosomal changes across several bases or chromosomes (SV), and genome-wide changes (CNV) in tumour-PDX pairs and tumour-PDX-PDO trios.

In our PDAC cohort of paired tumour-PDX-PDO trios, the organoid samples are derived from the PDX, not the original patient donors. Given this experimental setup, the overall expectation is that the PDO would demonstrate greater fidelity with the PDXs. An assessment of structural variation changes across the trios demonstrated stronger concordance between PDX-PDO pairs than patient-PDX or patient-PDO pairs (ex: PCSI_0590, PCSI_0602, PCSI_0642). Our analysis of copy number variation across 3 trios also indicated that PDX-PDO pairs scored marginally higher in terms of overall concordance, with fewer copy number changes in individual chromosomes. Collectively, these findings suggest that true PDO representation of the donor tumour would benefit from direct growth from the patient tumour itself.

At the single-base and gene-centric level, our findings emphasize that PDAC disease models capture many of the mutational patterns and driving events involved in PDAC tumourigenesis, up to an extent. About ~1/3 of the observed SNVs are not shared by the tumour of origin and its matching PDX, rendering an intermediate scoring of tumour-PDX agreement across the majority of the PDAC pairs, and a rather low concordance in 30% of the models. However, there is greater concordance between tumours (primary or metastases) and matched PDX for functional mutation types (missense and nonsense mutations). A more detailed evaluation also demonstrated retention of genetic features for 9 driver genes involved in PDAC tumourigenesis. As such, our study confirms and extends prior findings that the genetic mutations of key driver genes are maintained between tumour-PDX pairs [4, 8, 20], and we now demonstrate that this fidelity extends to PDOs as well. Given the complex molecular landscape of PDAC [21], consistency of genomic changes in oncogenes and tumour suppressor genes provides further overall support for use of PDXs and PDOs in precision medicine. PDOs are particularly attractive, as they can be readily established from small patient biopsies [16]. However, our findings also posit a greater variability between tumours and disease models in terms of overall mutation load. The effects of this variability would need to be addressed further in future investigations. There remain unexplored categories of mutations (for example, mutations in lincRNA) that may warrant further exploration, to determine the effect of these mutations on genome-wide changes in PDAC.

Investigations into copy number variation emphasize that ploidy changes are a substantial factor that must be considered when conducting tumour-model investigations. Two of our 16 samples had undergone severe ploidy changes that resulted in their exclusion from the analysis. While many of the remaining tumour-PDX pairs and trios demonstrated comparable ploidy, our analysis also captured conspicuous cases of whole genome polyploidization events that gave rise to tetraploid genomes in matched disease models (notably across PCSI_0590 and PCSI_0633). Accordingly, measuring consistency had to be conducted on ploidy-scaled copy number events. With these corrected measurements, we were able to conclude that there is agreement of copy number events at genome-wide scales (overall concordance score) and chromosome-centric levels. However, we are cognizant that these normalized measures potentially mask discrepancies that are more readily apparent when comparing raw (uncorrected) copy number measurements. This is of import when assessing copy number changes in chromosomes that exhibit large-scale, complex genetic events, such as chromothripsis.

Deviation in ploidy underscores potential changes that arise when transferring portions of primary tumours to other disease model mediums, particularly across multiple passages. In our work, WGS profiling of PDX and PDOs was undertaken following third passage (P3) engraftment of tumours into mice. While the lack of profiling of earlier PDX passages prevents us from drawing conclusions about copy number aberrations (CNAs) in PDAC across multiple passages, one limitation includes the selection of subclones when different portions of the tissue are grown in future passages within PDXs and PDOs. Subclones may gain survival advantage growth in the disease models, and subsequently, tumour engraftment in PDXs may be confounded by underlying biological mechanisms that promote adaptation and growth of these tumours in a new environment [22]. Indeed, recent findings by Ben-David *et al*, based on observations from breast and hematopoietic cancers, suggest that clonal evolution of PDX occurs through directional selection of pre-existing clones [23]. Interestingly, their study emphasized quick genomic divergence and rapid CNA dynamics across the first few *in vivo* PDX passages, such that CNAs acquired through PDX passaging differ substantially from that in their parental tumours [23]. These revelations may explain the polyploidization events that we have observed in our PDAC PDXs, and the difficulty in attributing ploidy to some samples that were eventually excluded from the study (PCSI_0592 and PCSI_0602). We have attempted to examine the clonality of the primary tumours, metastases, and matching models with our current data. In many cases, clonality of SNVs are preserved across the different models belonging to a given patient. However, we observed differences in clonality and cellular prevalence for some clusters of SNVs, particularly for samples with ploidy changes (ex: PCSI_0602, PCSI_0633).

Assessment of structural variation (SV) events sheds light on biological phenomena that have not been previously described in PDAC disease models. Genome-wide scoring of SV concordance (S^c^ score) showed poor agreement between tumours and PDX, which promoted further investigation into chromosome-specific scoring methods to efficiently depict this heterogeneity. At this deeper level, many chromosomes still scored poorly in tumour-PDX comparisons. Strikingly however, we identified cases of genomic consistency across chromosomes with clustered SV events that suggest chromothripsis. In that context, chromosomes with chromothripsis in the primary tumour exhibited similar behavior in matching PDX and matching PDO. For these particular chromosomes, in resected patients, the actual rearrangements of the SV clusters appear to be quite different between tumour and PDX. However, these rearrangements are almost identical when comparing metastases-PDX pairs. Despite evidence of overall structural heterogeneity between tumours and their matched models, these findings argue that major structural changes that occur in a tumour sample can be largely ‘transmitted’ to matched PDXs and PDOs. There remain, however, other anomalous cases where clustered SV events in specific chromosomes within tumour samples have been ‘lost in translation’ in matched PDX and PDO (the most notable case is PCSI_0624, chromosome 17). Equally difficult to rationalize are cases where clustering of SV events have arisen in the matched PDX, but not in the source tumour (PCSI_0611, chromosome 22). While these events may once again be explained as a result of subclonal events or tumour selection, our limited sample size hinders investigations as to whether those events could be recurrent events in a larger PDAC series.

Our study presents a comparative analysis of patient tumours and disease models for both primary PDAC and metastasis. While our samples do not allow direct comparisons of matched primary and metastatic tumours, it is possible to draw conclusions about the overall genomic profile of the metastatic samples (and their matched PDX) in comparison with the primary series (and their matched PDX). Across all types of genomic events studied (SSM, SV, CNV), we observe that metastases demonstrate higher concordance with their matched PDX compared with primary-PDX pairs. All of the pairs demonstrate higher concordance levels (Jaccard index ≥ 0.6) for both SSM and CNV. SV heterogeneity across these pairs is less pronounced than those of primary tumours, producing improved concordance scores for SV events. Concordance at the SV level is also higher in metastases than primary tumours when considering chromosome-centric analyses, or focusing on categories of SV events (that potentially may have functional implications). The rearrangement of SV events for chromosomes with chromothripsis is almost identical between metastases-PDX pairs, and there are fewer discrepancies in copy number states. One possible explanation for this behavior may reflect upon the tumour microenvironment of metastatic samples compared to primary tumours, as metastatic samples represent more stable, aggressive, and proliferative derivatives of the primary tumour. Interestingly, our cohort of metastasis-PDX pairs also includes a sample demonstrating DNA mismatch-repair (MMR) deficiency (PCSI_0489) due to a germline MLH1 mutation. Alterations in MMR genes lead to microsatellite instability (MSI), a genotype found infrequently in PDAC [11, 24, 25]. Our analysis of the MMR sample highlights a high mutation load, few SV events across chromosomes, and a stable DNA copy number across the genome; all of these observations are reflective of the genomic instability expected in tumour samples exhibiting MMR deficiency. Observing the same behavior in the matched PDX is reassuring, as it emphasizes that the PDX model succeeds in recapitulating much of the genomic behavior of these rare and striking PDAC cases.

Our approach presents quantifiable parameters of model fidelity; assessing molecular similarity between tumour-model pairs is necessary towards utilization of these models in future preclinical testing. For example, our analysis highlighted a discordant tumour-PDX pair (PCSI_0169) that was consistently difficult to decipher across both SSM and SV comparisons. One possible explanation for this discordance may be a mutational process that is only present in the PDX, due to a clone from the primary tumour growing as the main clone in the PDX. Further investigations would be needed to explore this phenomenon; however, this is a clear case where the PDX may not a good surrogate for preclinical testing of the donor tumour. Deciphering the extent of discordance for that PDX sample would not have been possible without scoring donor-model comparisons across genome-wide, single-base, and chromosome-centric scales.

This study has several potential limitations. Our limited sample size hampers investigations into subclonal events within individual samples. Due to a lack of sufficient technical replicates for each disease model studied, it is difficult to measure within-line variability in the models. We have assessed potential clonality changes between tumours and their matched models for each patient, and present findings that suggest stable clonality in tumour-model comparisons, with notable exceptions. These however, are preliminary findings, as our limited sample size precludes explanations as to the prevalence of different clones or their statistical significance. Our findings demonstrate that larger-scale investigations to assess transcriptional and clonal evolution across these disease models will be warranted in the future, similar to previous analyses highlighting genomic evolution across *in-vitro* models [26]. Currently, our sample counts are also too few to support comprehensive identification of recurrent genomic patterns across a larger cohort of PDXs and PDOs, including, for example, recurrently affected genes. Future investigations will be necessary to estimate the number of technical replicates needed per PDX line that would be sufficient to capture variability (or stability) in a given model. This information will certainly be important when planning PDX screens for pre-clinical testing.

Genomic comparisons between primary tissues and disease models are necessary for future studies that focus on gene-drug associations across model systems. While we have highlighted in this manuscript examples of common and discrepant genomic aberrations between tumour-model pairs, the phenotypic relevance of these aberrations remains to be investigated. Previous work on PDAC disease models, using whole-exome sequencing, demonstrated that drug sensitivities remained stable across multiple passages of PDAC cell lines, suggesting a conservation of dominant genetic drivers and related therapeutic sensitivities [8]. However, other findings highlight that successful tumour engraftment of PDXs is associated with adverse clinico-pathological features and worse recurrence-free and overall survival; this variability in PDX growth suggests limited potential for systematic use of PDX tumours for real-time chemo-sensitivity testing [22]. From the WGS data presented here, it is not yet possible to conclude whether concordance in genomic aberrations would imply fidelity in drug response between tumour-PDX or PDX-PDO comparisons; this remains to be determined by comparing patient outcome and drug testing in future investigations, and comparing resultant gene expression patterns across tumour-model pairs.

In summary, we have conducted a detailed molecular dissection of WGS data to quantify concordance of genomic events between model systems and matched human PDAC. We present the first extensive genomic evaluation of consistency and in-consistency across pre-clinical models, for different levels of genomic complexity (SNV, SV, CNV). Broadly, our findings shed light on consistency between disease models for mutations in PDAC driver genes, but highlight relatively poor agreement between tumours and their matching models across the greater spectrum of mutational load. Disease model fidelity is not retained well when assessing structural variation events, but there are striking cases of clustering of SV events across particular chromosomes that are retained when tumours are implanted into their respective disease models. Copy number comparisons suggest good agreement across tumour-model comparisons, conditional of reducing potential changes in ploidy. Across all of these platforms, our systematic comparison of tumours, PDX, and PDO also highlights several genetic aberrations that are sample-specific, and which may not be shared across donor samples and matched models. Collectively, our investigations highlight that while PDXs and PDOs may serve as tractable and transplantable systems for probing the molecular properties of PDAC, these models may best serve selective analyses across different levels of genomic complexity. We expect that our analytic pipeline may serve as a framework for future WGS research that compares donor samples and matched PDX and PDO.

## Materials and methods

A schematic overview of the biospecimens and analytic design is presented in Fig 1 and S1 Fig.

### Model system derivation

PDX were established by subcutaneous implantation of fresh surgically resected primary tumour tissue into immunodeficient mice [27]. All animal manipulations were approved by the University Health Network Animal Welfare Committee.

PDO models were generated by the Princess Margaret Living Biobank core facility using previously described protocols [16]. Briefly, fresh PDX tissue was cut into small pieces and dissociated to single cells or small clumps of cells using Liberase^™^ TH (Sigma Aldrich, Ontario, Canada). Dissociated cells were collected and embedded in growth factor-reduced Matrigel (Corning, New York, USA), which is overlaid with growth medium [16]

### Histopathology

Snap frozen tumour tissue 5mm^3^ or larger were obtained for each case and stored at -80°C. Each tissue was serially cryosectioned (10um thickness) at -20°C, fixed with 100% ethanol for up to 30 min and mounted onto PEN membrane 1.0 slides (Carl Zeiss MicroImaging, GmbH, Munich, Germany). All but one section was stained to visualize structures using a cresyl violet protocol that stains Nissl granules purple. Sections were rinsed in deionized water and stained in 1% cresyl violet solution (1% w/v in 50% ethanol, 50% deionized water) for 1 minute, dipped in 70% ethanol and dehydrated by dipping in absolute ethanol. Slides were left to dry for approximately 15 minutes before being stored at -80°C in air tight, aluminum foil wrapped slide boxes. The last section of each series was mounted onto an uncharged slide and stained using a modified haematoxylin and eosin (H&E) protocol. Sections were rinsed in deionized water, stained in Mayer’s haematoxylin (10 minutes), rinsed with deionized water, blued in Scott’s tap water, rinsed in deionized water, stained in aqueous eosin Y (1 minute), dipped in 3 changes of absolute ethanol, dehydrated in three changes of xylene (2 minutes each), prior to coverslipping. Additional details on the histochemistry and laser capture microdissection of xenograft samples have been previously described [11].

### Laser-capture microdissection (LCM)

LCM was performed on the PDX as previously described [11]. Briefly, cresyl violet stained slides were brought to room temperature. Tumour cells from each cresyl violet section were microdissected using the PALM LMPC device (Carl Zeiss MicroImaging, GmbH, Munich, Germany). Tissue was collected in AdhesiveCap tubes (Carl Zeiss MicroImaging, GmbH, Munich, Germany) and stored at -80°C prior to extraction.

LCM of fresh frozen tissue samples from PDAC was performed on a Leica LMD 7000 instrument. Frozen tissue for tumour samples was maintained in vapor-phase liquid nitrogen and embedded in OCT cutting medium and sectioned in a cryotome into 8-μm thick sections. These sections were then mounted on PEN membrane slides (Leica) and lightly stained with hematoxylin to distinguish tumour epithelium from stroma. A pathologist (SF) marked tumour sections and LCM was performed on the same day according to manufacturer’s protocol on the Leica LMD7000 system. Microdissected tumour cells were collected by gravity into the caps of sterile, RNAse-free microcentrifuge tubes. Approximately 150,000–200,000 tumour cells were collected for each DNA extraction and stored at –80°C in Arcturus PicoPure Extraction Buffer.

### Whole genome sequencing

Paired-end cluster generation and sequencing was carried out using the Illumina HiSeq 2500 platform, on DNA isolated from fresh frozen tissue following LCM. Whole genome sequencing (WGS) of tumours, PDX, and PDO was performed with a minimum depth of ~30X per sample. Xenome (version 1.0.1) [28] was used to identify and filter mouse content. Non-mouse DNA reads from primaries, PDX and PDO were aligned to the human reference genome hg19 using the Burrows-Wheeler Aligner (BWA, version 0.6.2) [29] with default parameters. Picard (version 1.90) (http://broadinstitute.github.io/picard) was used to sort, merge, and mark duplicates from multiple lanes of the same sample, followed by the Genome Analysis Toolkit (GATK, version 1.3.16) [30, 31] to improve alignment accuracy.

### Simple somatic mutations (SSM)

Germline single nucleotide variants (SNV) were called using the Genome Analysis Tool Kit (GATK, version 1.3.16), using best practice guidelines made available by the Broad Institute. Briefly, data were locally realigned around indels and the base quality values were recalibrated prior to variant calling using the Unified Genotyper. This was followed by filtering using the VariantFiltration module, and subsequent classification of germline variants as those mutations which have a QUAL score greater than 50 in the normal sample. Both tumour and matched normal samples were processed simultaneously.

Strelka (version 1.0.7) [32] and MuTect (version 1.14) [33] were used to call somatic SNVs, with default parameters. Indels were also identified using Strelka. SNVs were selected based on the intersection of ‘Tier 1 SNVs’ from Strelka and ‘PASS’ filter variants from MuTect. Potential false positives caused by unfiltered mouse DNA were filtered using a blacklist of SNVs and INDELS generated by aligning model mouse DNA to hg19. Germline and somatic SSM were annotated using dbSNP 142 [34], COSMIC (version 54) [35], and ANNOVAR (version 2013-06-21) [36] to predict coding consequences of SNVs and indels. Functional consequences of mutations were predicted using Oncotator (version 1.5.3.0) [37].

Parsing of VCF files containing the filtered calls was conducted using the *vcfR* (version 1.4.0) [38] and *VariantAnnotation* (version 1.18.7) [39] packages in R. Measurements of read depth of the variant and reference alleles were extracted to plot the frequency of reads carrying the variant allele. Assessment of mutation patterns for PDAC driver genes across all samples was performed by parsing the output generated by Oncotator using the *GenVisR* package (version 1.0.4) [40].

Concordance of mutation calls between a tumour and its matching PDX was calculated using the Jaccard Index. For each mutation category annotated by Oncotator, the Jaccard Index for that mutation type (J_М_) was calculated as follows:

where

$${\text{J}}_{М}={\text{C}}_{М}/\left({\text{T}}_{М}+{\text{P}}_{М}-{\text{C}}_{М}\right)$$

М = Oncotator mutation type (ex: lincRNA, missense mutation)

T_М_ = Number of variant calls in the tumour sample annotated with mutation м

P_М_ = Number of variant calls in the PDX annotated with mutation м

C_М_ = Number of identical variant calls in both the tumour and PDX (i.e., share the same chromosomal position and base pair substitution), annotated with mutation м

### Structural variation

Structural variations (SV) were called using CREST (version alpha) [41] and DELLY (version 0.5.5) [42] with default parameters, and high-confidence SVs subsequently filtered. For high-confidence SV calls observed in at least one sample of a tumour-PDX pair, we manually reviewed whether the same variant was observed in the matched sample with lower frequency, and added those ‘rescued’ variants to the filtered list. The union of the filtered calls and the rescued calls was used for all downstream analysis.

Genome-wide structural changes across all the four categories of structural variation (DEL = deletion, INV = inversion, DUP = duplication, and TRA = translocation) in tumour-PDX pairs were rendered using the *RCircos* library (version 1.2.0) [43]. Quantification of structural variation events in all PDAC and liver metastasis tumour-PDX pairs was calculated using functions from the *RCircos* (version 1.2.0) [43], *GenomicRanges* (version 1.24.3) [44], *rtracklayer* (version 1.32.2) [45] and *PharmacoGx* (version 1.1.6) [46] packages in R.

Assessment of concordance and discordance among SV events was conducted for each chromosome individually across tumour-PDX pairs. We first tabulated the number of SV events observed across tumour, PDX, and PDO, to determine the overall distribution of SV events across tumours and their matched disease models. We identified chromosomes with ≥ 5 SV events in both tumours and their matching PDX. To count SV events per chromosome, intra-chromosomal events (deletions, inversions, duplications) were assigned a score of ‘1’ for their respective chromosomes, while a score of 0.5 was assigned to each of the chromosomes involved in a translocation event. Instances of discordance (where one sample of a pair had ≥ 10 SV events different from the other sample) were also noted.

Chromosome-specific concordance of structural variation events for a tumour and its matching PDX was quantified using the Jaccard Index, taking into account the genomic size (number of the genomic bases) of the SV events. For deletion (DEL), Inversion (INV), and Duplication (DUP) events, the genomic size of the event was calculated from the annotated breakpoints. For translocation (TRA) events, the total number of bases affected was set to 1. Jaccard indices of a tumour-PDX pair were then calculated individually for each chromosome (J_Ѵ_), across all SV events as follows:

$${\text{J}}_{Ѵ}={\text{C}}_{Ѵ}/({\text{T}}_{Ѵ}+{\text{P}}_{Ѵ}-{\text{C}}_{Ѵ})$$

_Ѵ_ = chromosome (autosomes in addition to chrX and chrY)

T_Ѵ_ = Sum of genomic bases from SV calls identified in the tumour in chromosome _Ѵ_

P_Ѵ_ = Sum of genomic bases from SV calls identified in the PDX in chromosome _Ѵ_

C_Ѵ_ = Sum of genomic bases from SV calls that are identical in both the tumour and the PDX in chromosome _Ѵ_. It is assumed that every reported SV is a separate event with a well-defined start and end breakpoint. Identical SV calls were determined by comparing chromosomal breakpoint positions in both the tumour and the PDX, and selecting SV events sharing the same start and end breakpoint positions.

We also generated an overall concordance (S^c^) score to summarize the agreement between tumour-PDX pairs. The score determines the ratio of ‘positive’ chromosomes across a tumour-PDX pair that have a Jaccard index ≥ 0.6. The score is calculated as follows:

$${\text{S}}^{\text{c}}=\text{P}/\left(\text{P}+\text{N}\right)$$

where

P = number of chromosomes with J_Ѵ_ ≥ 0.6

N = number of chromosomes with J_Ѵ_ ˂ 0.6

Concordance between tumour-PDX pairs was additionally calculated based on the type of SV event, at both genome-wide and chromosome-specific levels. SV events were divided into 4 categories: deletion (DEL), inversion (INV), duplication (DUP), and translocation (TRA) events. Concordance of SV events, per category, was determined as follows:

$${\text{J}}_{К}={\text{C}}_{К}/({\text{T}}_{К}+{\text{P}}_{К}-{\text{C}}_{К})$$

К = category of SV event (DUP, DEL, INV, TRA)

T_К_ = Sum of genomic bases in the tumour sample, across all SV calls annotated as category К

P_К_ = Sum of genomic bases in the PDX sample, across all SV calls annotated as category К

C_К_ = Sum of genomic bases for SV events that are identical in the tumour and the PDX, across all SV calls annotated as category К

Jaccard scores by SV category were calculated genome-wide (across all chromosomes), and calculated individually for each chromosome (autosomes in addition to chrX and chrY).

In the case of trios (tumour-PDX-PDO), SV concordance was quantified by splitting the trio into pairwise calculations of tumour-PDX, tumour-PDO, and PDX-PDO, and then calculating J_Ѵ_, J_К_, and S^c^, as described previously.

### Copy number variation

Copy number segments were obtained using CELLULOID (version 0.11.2) to estimate gene copy number and tumour ploidy from WGS [12]. Unless otherwise specified, copy number segments and parameters were extracted for the first solution (solution1) of the CELLULOID proposed solutions, for each sample.

Concordance of copy number state was calculated by first identifying overlapping genomic loci in tumour-PDX pairs using bedtools (version 2.24.0) [47]. To consider the copy number state relative to ploidy, the *imean* scores generated by CELLULOID (which represents average integer copy-number) for the genomic loci were rescaled by the ploidy of the respective samples. Genomic loci in tumour-PDX pairs were considered identical if they shared a copy number state with a difference ≤ 0.25. Genome-wide concordance scores (G) were calculated across all bases of a tumour-PDX pair as follows:

G=I/(T+X–C)

T = Number of genomic bases in the tumour with a defined copy number (imean-rescaled value)

X = Number of genomic basis in the PDX with a defined copy number (imean-rescaled value)

C = Number of genomic basis in either the tumour or PDX with a defined copy number (imean-rescaled value)

I = Subset of C, where the absolute value of the difference between the imean score in the tumour and PDX is ≤ 0.25

Chromosome-specific concordance scores (G _Ѵ_) were also calculated individually for each chromosome as follows:

$${\text{G}}_{Ѵ}={\text{I}}_{Ѵ}/({\text{T}}_{Ѵ}+{\text{X}}_{Ѵ}-{\text{C}}_{Ѵ})$$

where

_Ѵ_ = chromosome (autosomes in addition to chrX and chrY)

T = Number of genomic bases in the tumour with a defined copy number (imean-rescaled value) in chromosome _Ѵ_

X = Number of genomic basis in the PDX with a defined copy number (imean-rescaled value) in chromosome _Ѵ_

C = Number of genomic basis in either the tumour or PDX with a defined copy number (imean-rescaled value) in chromosome _Ѵ_

I = Subset of C, in chromosome _Ѵ_, where the absolute value of the difference between the imean score in the tumour and PDX is ≤ 0.25

In the case of trios (tumour-PDX-PDO), CN concordance was quantified by splitting the trio into pairwise calculations of tumour-PDX, tumour-PDO, and PDX-PDO, and calculating G and G _Ѵ_ scores, as described previously. Plots of overlapping copy number states were drawn using the *copynumber* package (version 1.12.0) [48].

### Ranking concordance or discordance between models

Quantitative comparison of SSM, SV, and CNV events between tumours and their corresponding PDX and PDO samples were developed by calculating Jaccard indices (J_М_, J_Ѵ_, J_К_) or concordance scores (S^c^, G, G _Ѵ_), as described previously. These scores follow a scale from 0 to 1. A score of 0 indicates complete *discordance* between a tumour and its matching model, and signifies that none of the genomic aberrations identified in the tumour could be identified in the matching model. A score of 1 indicates complete *concordance*, such that all genomic aberrations identified in the tumour are successfully recapitulated in the matching disease model.

### Clonality across disease models

The clonality of tumours, metastases, and disease models was determined using PyClone [49] on SNVs that were called in all of the samples derived from a given patient (tumour and matching PDX and PDO, as appropriate). SNVs were first filtered to exclude SNVs marked as intronic or intergenic. In PyClone, SNVs are clustered based on their common clonality and colored by cluster. Points are drawn at the cellular prevalence of the SNV in each sample (i.e., the proportion of tumour cells carrying the mutations, irrespective of the number of copies of the mutant allele).

### Data access

The datasets analyzed during the current study are available in the European Genome-phenome Archive (EGA), accession code EGAS00001002597. Comparison of SSM, SV, and CNV events between tumours and their corresponding PDX and PDO sample was conducted using R (version 3.3.1) [50]. All software dependencies are available on Bioconductor (BioC) or the Comprehensive Repository R Archive Network (CRAN), and have been listed throughout the methods as applicable. The code and associated tutorial describing how to run the analysis pipeline are publicly available on Github (github.com/DGendoo/PDACDiseaseModels), and processed data used by these scripts are provided as a zipped folder named ‘PDAC_WGS_Processed_Data’ under https://figshare.com/articles/PDAC_WGS_Processed_Data/5552767.

## Supporting information

S1 Fig Schematic overview of study analysis. (PDF)

S2 Fig Immunohistochemistry staining (H&E, CK19) for PDX and PDO samples of the trios. (PDF)

S3 Fig Frequency of reads carrying the variant allele for tumour-PDX pairs and tumour-PDX-PDO trios. Oncogenes, tumour suppressors, and genes involved pathways of PDAC tumourigenesis are plotted. **(A)** Frequency of reads carrying the variant allele for primary-PDX pairs. **(B)** Frequency of reads carrying the variant allele for metastasis-PDX pairs. **(C)** Frequency of reads carrying the variant allele for the trios. The trio is split into primary-PDX (top), primary-PDO, and PDX-PDO (bottom) pairs.
(PDF)

S4 Fig Circos plots of SV events across the genomes of each tumour-PDX pair. Each type of SV event is color-coded with a similar color between tumours and matching PDX. For each SV type, tumours are annotated on the outer rings of the circos plot and the matching PDX on the inner rings. SV events are colored as follows: deletions (red), inversions (green), and duplications (blue). Translocation events between chromosomes are also depicted (center). **(A)** Circos plots of SV events across the genomes of 10 primary-PDX pairs **(B)** Circos plots of SV events across the genomes of 6 metastasis-PDX pairs.
(PDF)

S5 Fig Jaccard similarity across tumour-PDX pairs, based on the category of the structural variation (SV) event. **(A)** Genome-wide jaccard scores, by SV category, for primary resected tumours and matched PDX. **(B)** Chromosome-specific jaccard scores, per SV category, for chromosomes exhibiting clustered SV events in primary resected tumours and matched PDX **(C)** Genome-wide jaccard scores, by SV category, for metastases and matched PDX **(D)** Chromosome-specific jaccard scores, per SV category, for chromosomes exhibiting clustered SV events in metastases and matched PDX.
(PDF)

S6 Fig Circos plots of SV events across the genomes of each primary-PDX-PDO trio (n = 5 samples). Each type of SV (deletion, inversion, duplication, and translocation) is represented as one circos plot, with 3 rings indicating tumour (outer), PDX (middle), and PDO (inner). SV events are colored as follows: deletions (red), inversions (green), and duplications (blue). Translocation events between chromosomes are also depicted (center).
(PDF)

S7 Fig Copy number profile across 8 matched primary-PDX pairs and 6 matched metastasis-PDX pairs, as rendered by CELLULOID. (PDF)

S8 Fig Copy number across 8 primary-PDX pairs and 6 metastasis-PDX pairs. For each pair, the copy number state of the primary tumour (magenta) and matching PDX (black) is plotted across all chromosomes. A detailed panel also shows the copy number of the tumour and PDX across each chromosome. Values presented are raw (uncorrected) copy number values.
(PDF)

S9 Fig Copy number profile across 3 primary-PDX-PDO trios, as rendered by CELLULOID. Complete CELLULOID solutions (solutions 1–5) are also provided for each of the samples which have been excluded from the analysis (PCSI_0592 and PCSI_0602).
(PDF)

S10 Fig Copy number across 3 primary-PDX-PDO trios. For each trio, the copy number of the tumour (black), matched PDX (red), and matching PDO (blue) is plotted across the genome. A detailed panel also shows the copy number of the tumour and PDX across each chromosome. Values presented are raw (uncorrected) copy number values.
(PDF)

S11 Fig Clonality across disease models using PyClone. PyClone output for **(A)** 10 resected primary samples and **(B)** 6 liver metastasis samples. Each graph represents one sample, with matching PDX and PDO (where applicable) labelled. SNVs are clustered based on their common clonality, and colored by cluster. Points are drawn at the cellular prevalence of the SNV in each sample.
(PDF)

S12 Fig Review of the current literature comparing PDAC tumours and disease models. (PDF)

S1 Table Meta-data of the samples studied. (XLS)

S2 Table IHC (H&E and CK19) observations pertaining to PCSI samples. (XLS)

S3 Table Total number of simple somatic mutation (SSM) calls across the cohorts. SSM calls across **(A)** primary tumours and matched PDX, **(B)** metastatic tumours and matched PDX, and **(C)** primary-PDX-PDO trios. Jaccard index for each tumour-PDX pair for mutation types are also shown for **(D)** primary tumours and matched PDX and **(E)** metastatic tumours and matched PDX.
(XLSX)

S4 Table Total number of structural variants (SV) calls across the cohorts. SV across **(A)** primary tumours and matched PDX, **(B)** metastatic tumours and matched PDX, and **(C)** primary-PDX-PDO trios. For each sample, the total number of deletions (DEL), duplications (DUP), inversions (INV), and translocations (TRA) is indicated.
(XLSX)

S5 Table Total number of structural variation events (SV) observed in each chromosome. Total events are indicated across **(A)** primary tumours and matched PDX, **(B)** metastatic tumours and matched PDX, and **(C)** primary-PDX-PDO trios. Deletions, duplications, and inversion events that occur in a chromosome were assigned a value of 1 prior to their summation, and translocation events assigned a value of 0.5.
(XLSX)

S6 Table Chromosome-specific jaccard scores for structural variation (SV) events. The indices are indicated across **(A)** primary tumours and matched PDX, **(B)** metastatic tumours and matched PDX, and **(C)** primary-PDX-PDO trios.
(XLSX)

S7 Table Jaccard score for across different categories of structural variation (SV) events. **(A)** Genome-wide jaccard scores, by SV category, for primary resected tumours and matched PDX. **(B)** Chromosome-specific jaccard scores, per SV category, for primary-PDX pairs **(C)** Genome-wide jaccard scores, by SV category, for metastases and matched PDX. **(D)** Chromosome-specific jaccard scores, per SV category, for metastasis-PDX pairs.
(XLSX)

S8 Table Celluloid metrics for each of the samples. Metrics are shown across **(A)** primary tumours and matched PDX, **(B)** metastatic tumours and matched PDX, and **(C)** primary-PDX-PDO trios. For each sample, the percentage of normal content (N), percentages of tumour content (T1), and the ploidy of the sample is indicated.
(XLSX)

S9 Table Chromosome-specific and genome-wide concordance score for copy number. Concordance is computed across **(A)** 8 primary-PDX pairs from resected tumour, **(B)** 6 metastasis-PDX pairs, and **(C)** 3 primary-PDX-PDO trios from resected tumour.
(XLSX)
