# Supplementary figures and images for: Whole genomes define concordance of matched primary, xenograft, and organoid models of pancreas cancer
Source: PLoS Comput Biol. 2019 Jan 10;15(1):e1006596. doi: 10.1371/journal.pcbi.1006596 (PMC6328084; doi:10.1371/journal.pcbi.1006596)

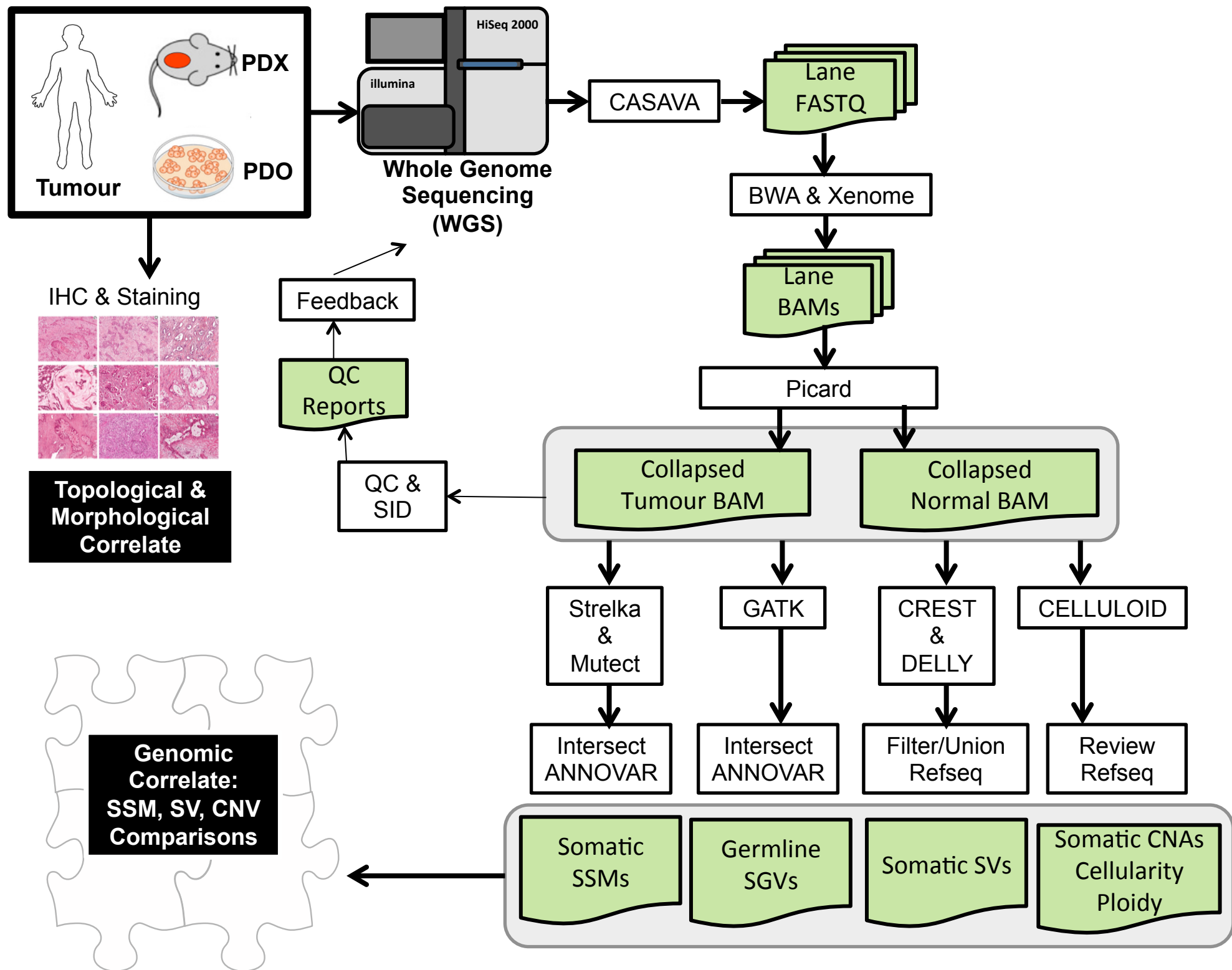

Supplement: S1 Fig — (PDF) [file pcbi.1006596.s001.pdf]

**H&E**

**CK19**

PCSI  
0590  
PDX

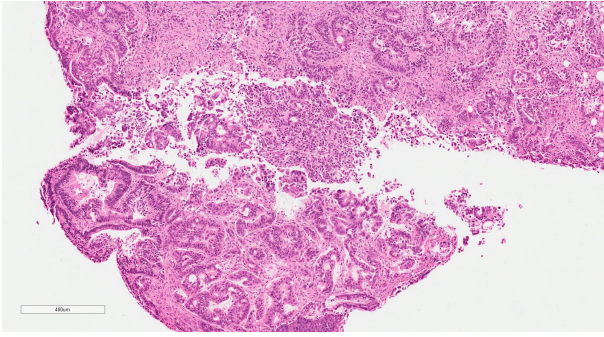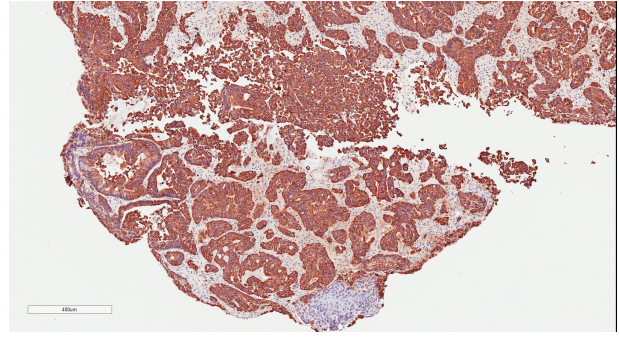

PCSI  
0590  
PDO

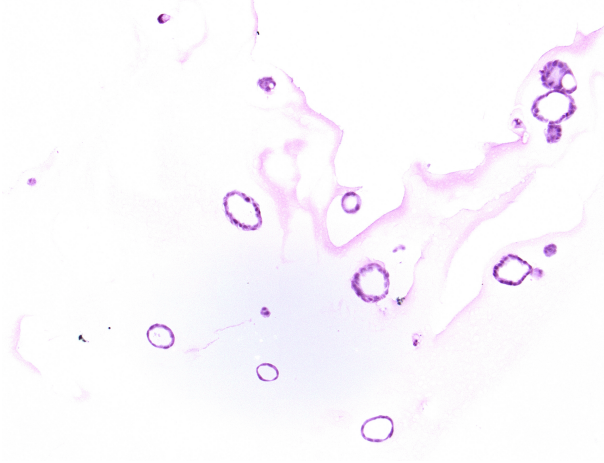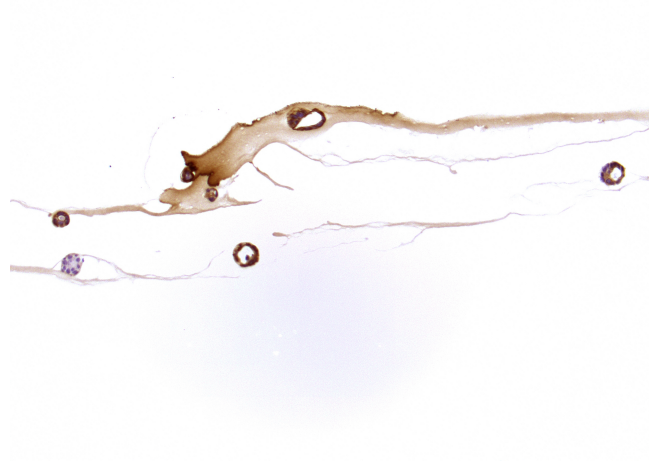

PCSI  
0592  
PDX

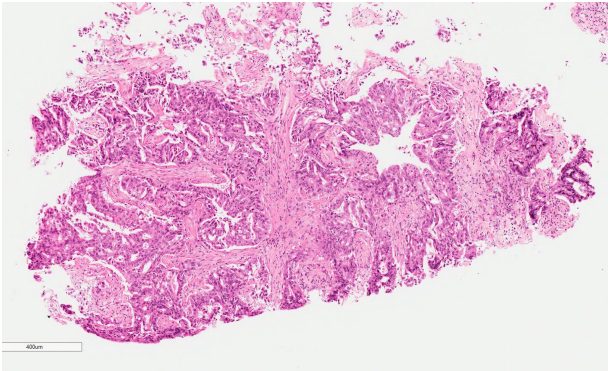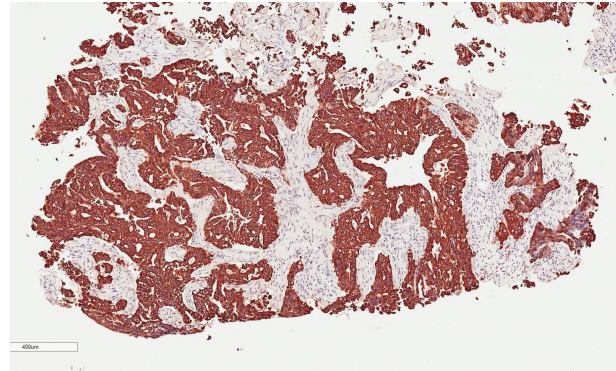

PCSI  
0592  
PDO

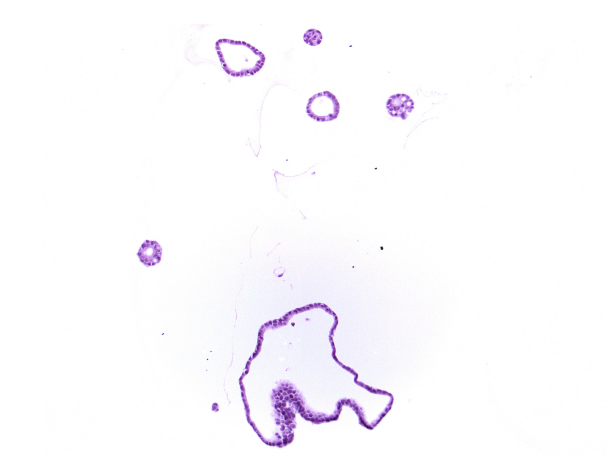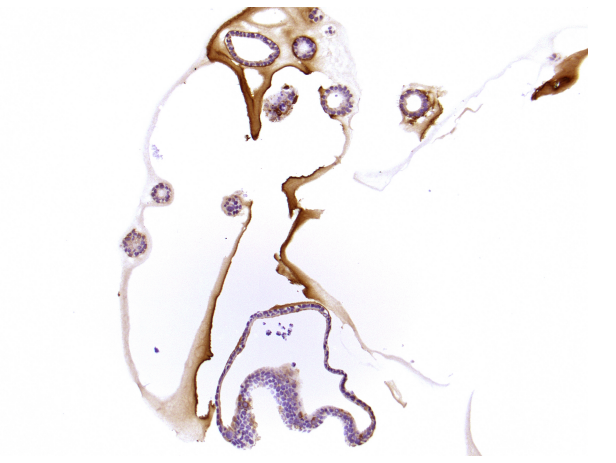

**H&E**

**CK19**

PCSI  
0602  
PDX

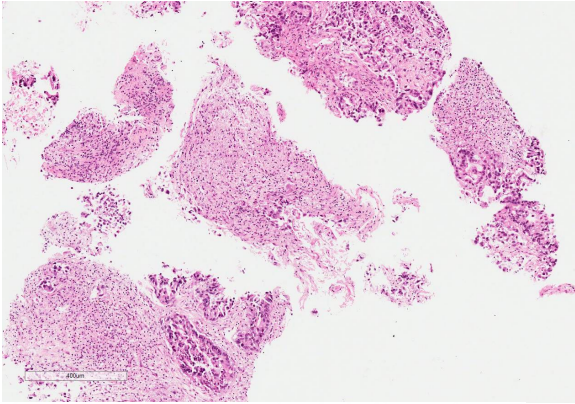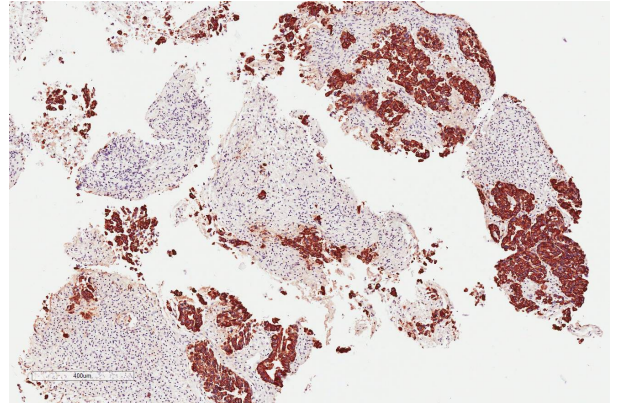

PCSI  
0602  
PDO

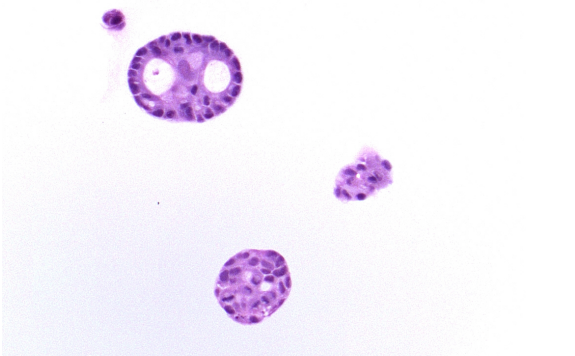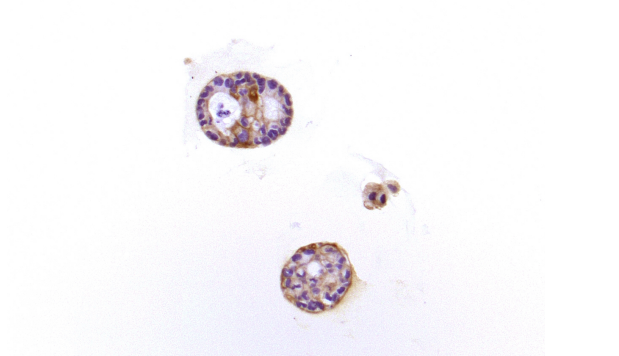

PCSI  
0624  
PDX

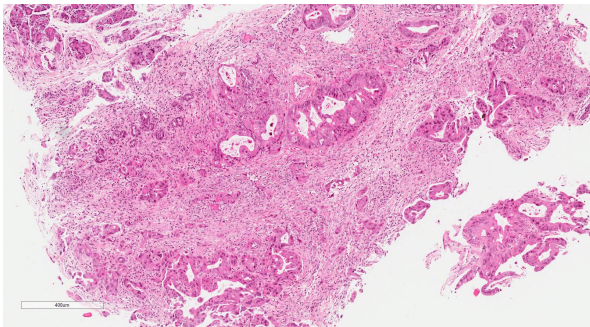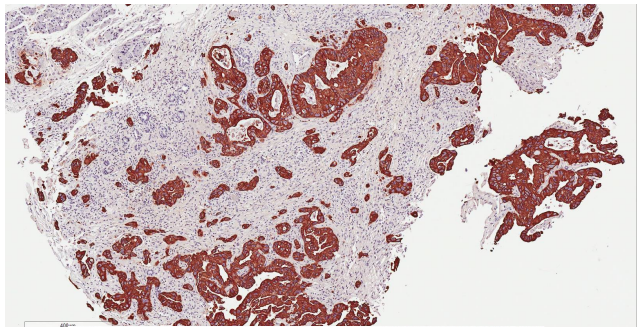

PCSI  
0624  
PDO

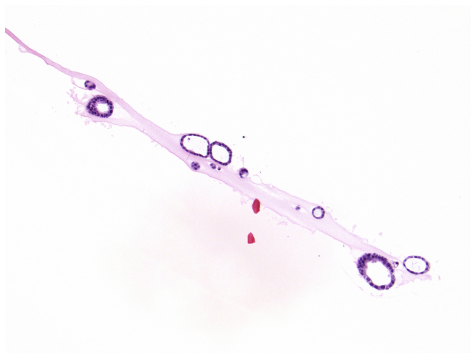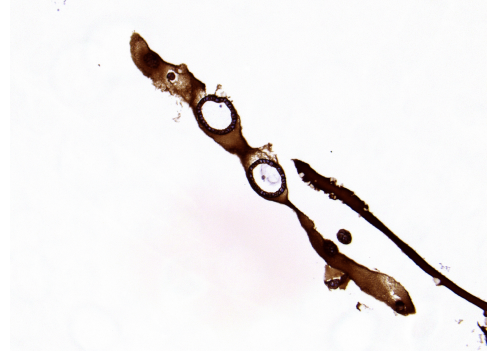

PCSI  
0642  
PDO

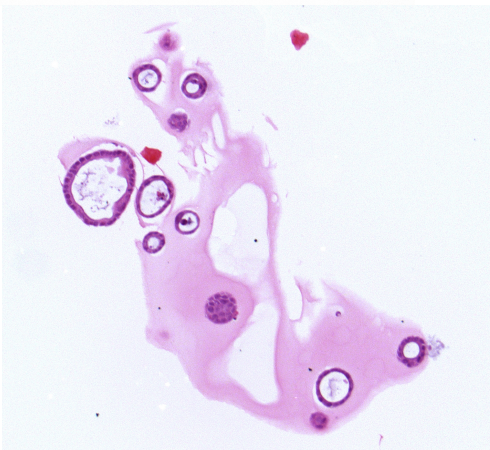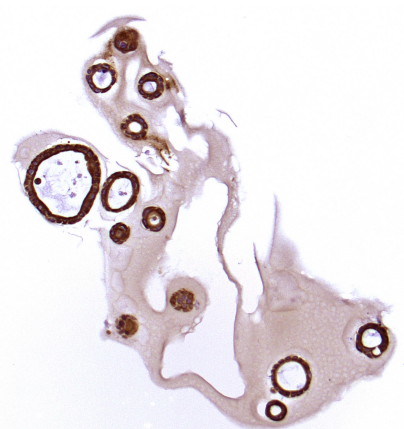

Supplement: S2 Fig — (PDF) [file pcbi.1006596.s002.pdf]

PCSI\_0169

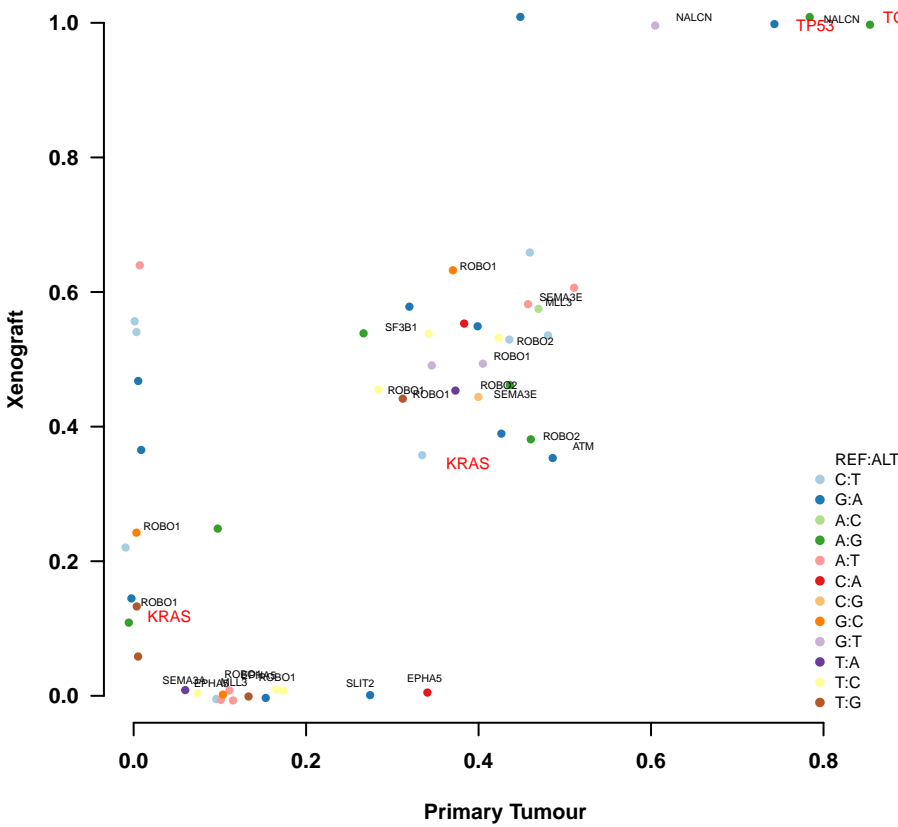

PCSI\_0355

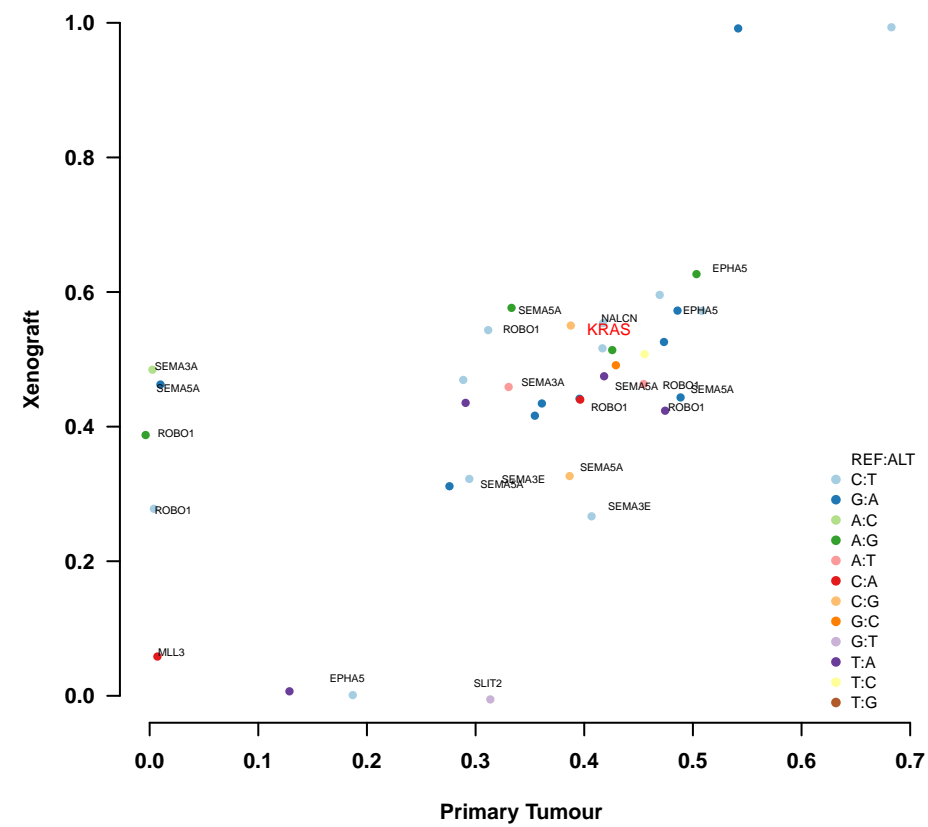

PCSI\_0589

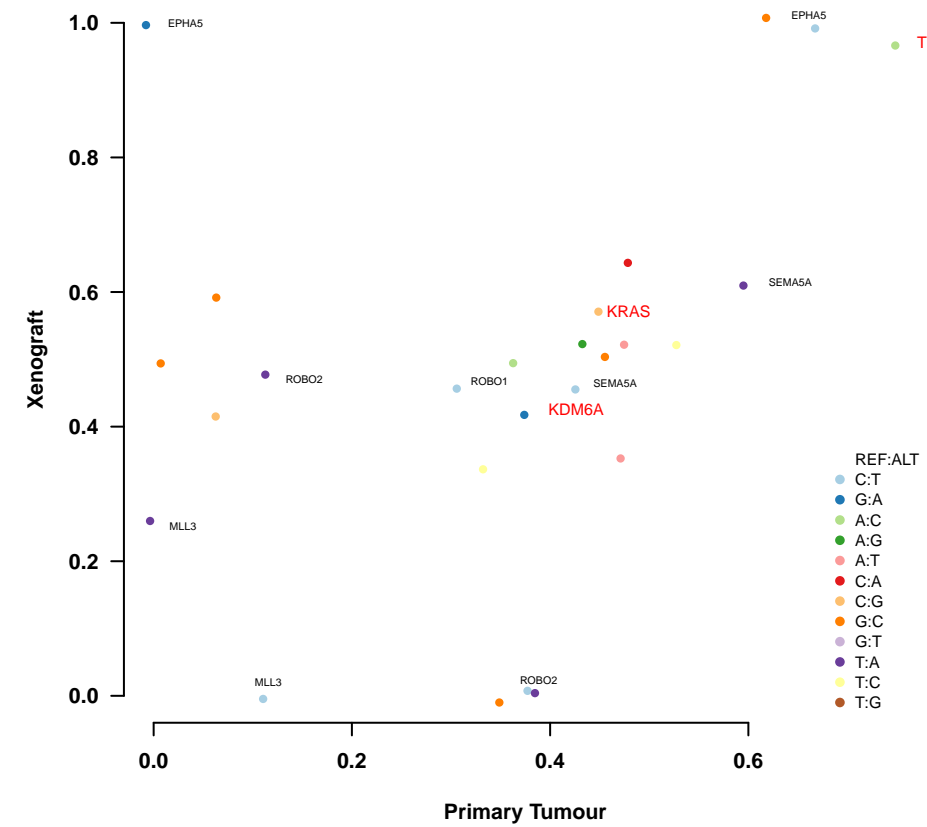

PCSI\_0590

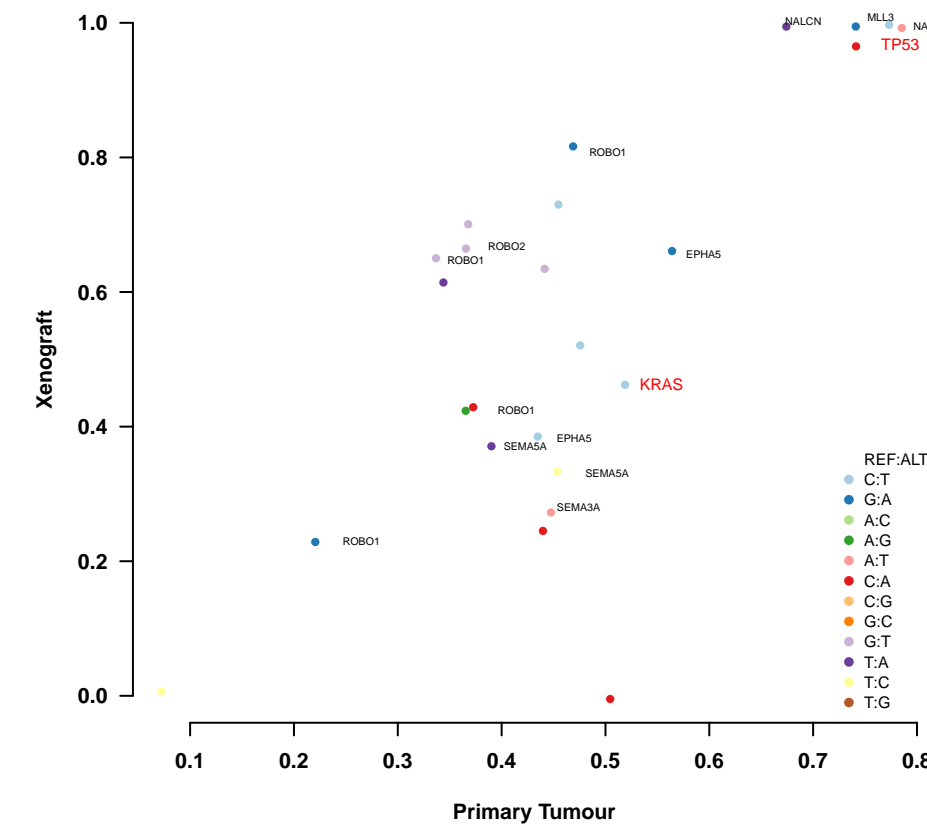

PCSI\_0592

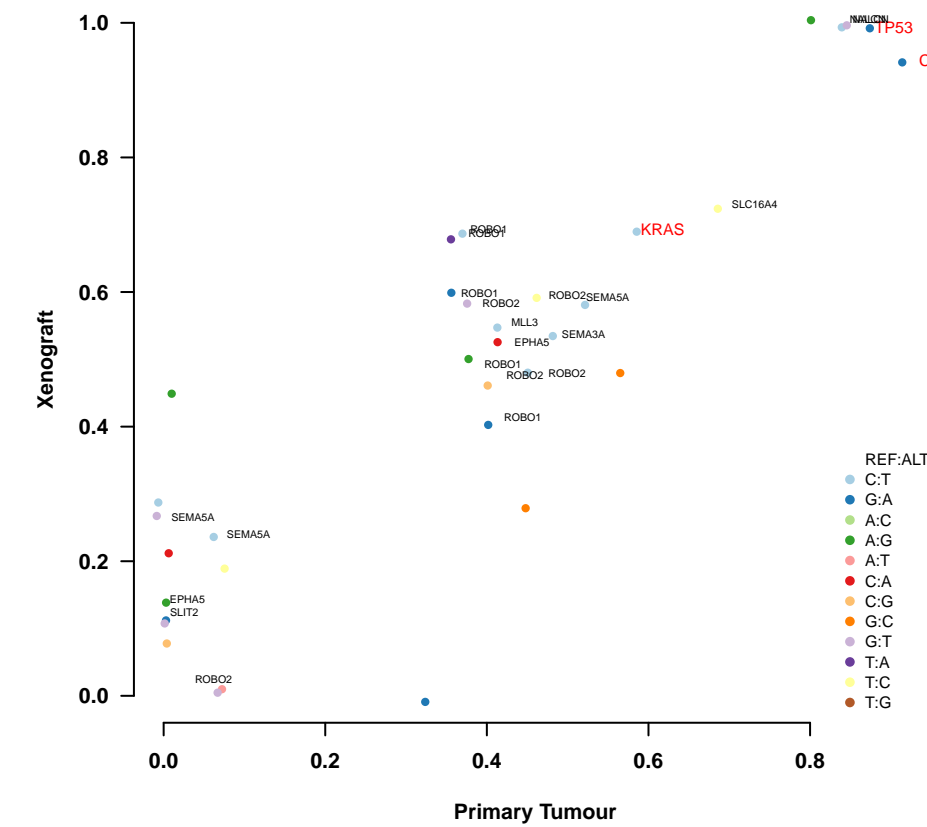

PCSI\_0602

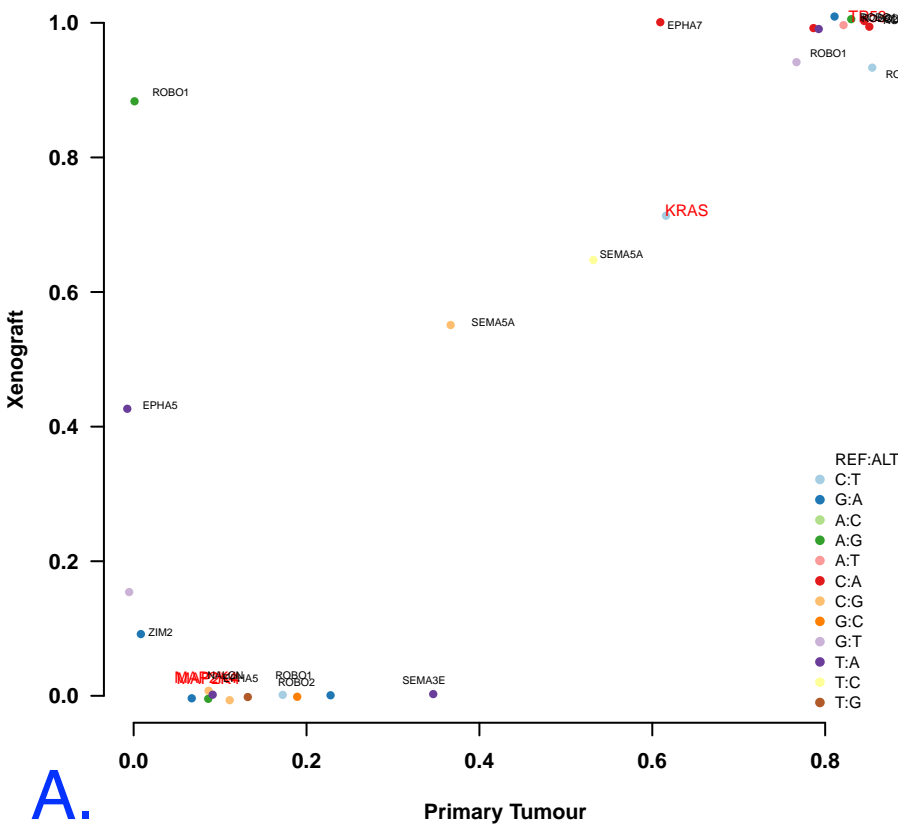

PCSI\_0611

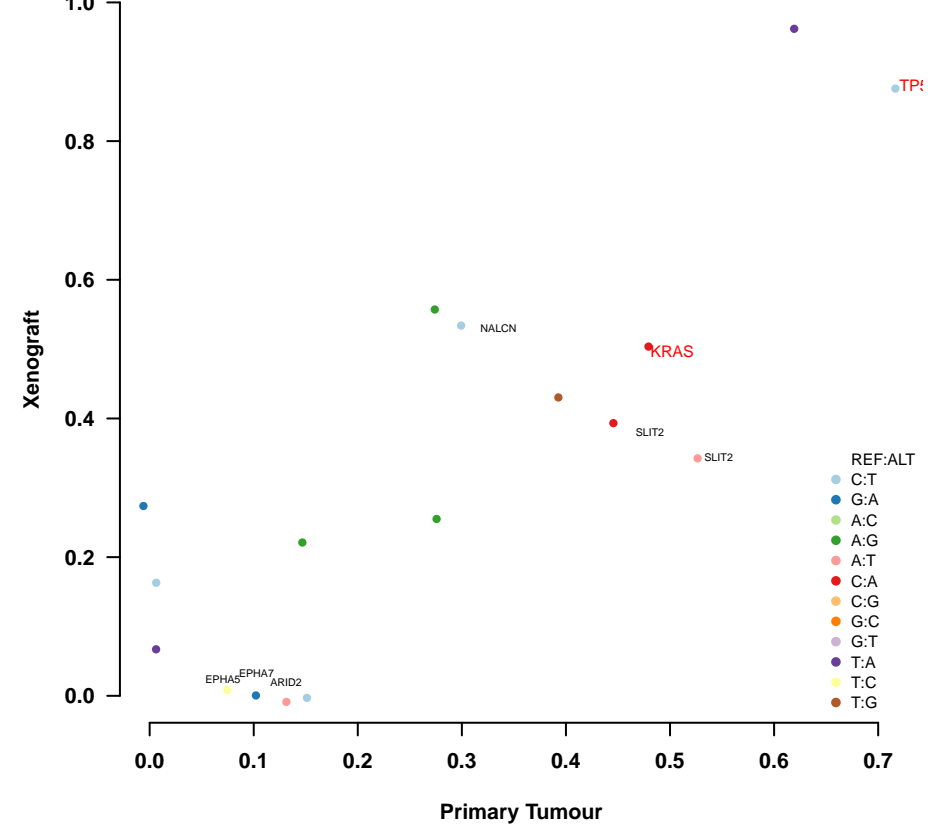

PCSI\_0624

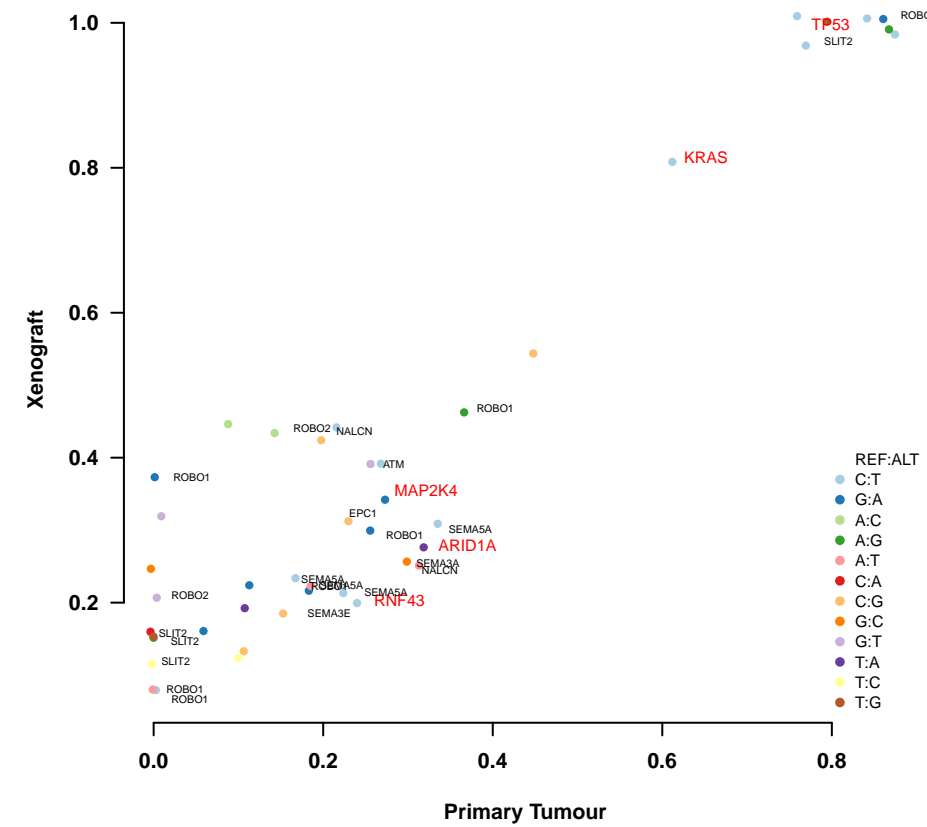

PCSI\_0633

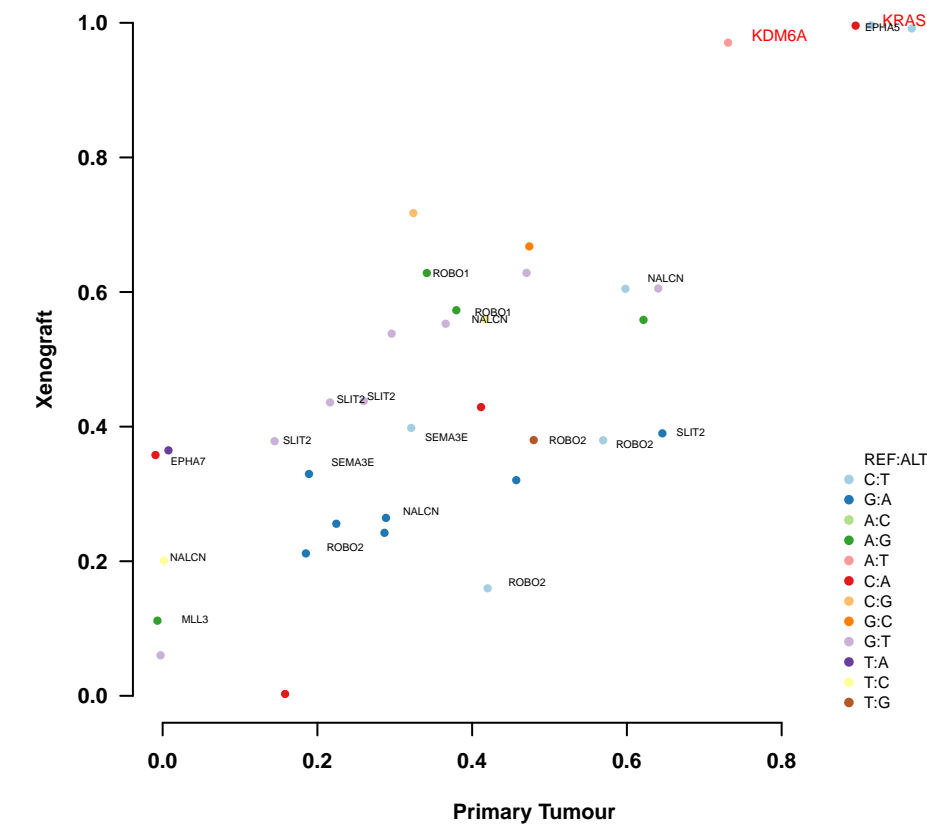

PCSI\_0642

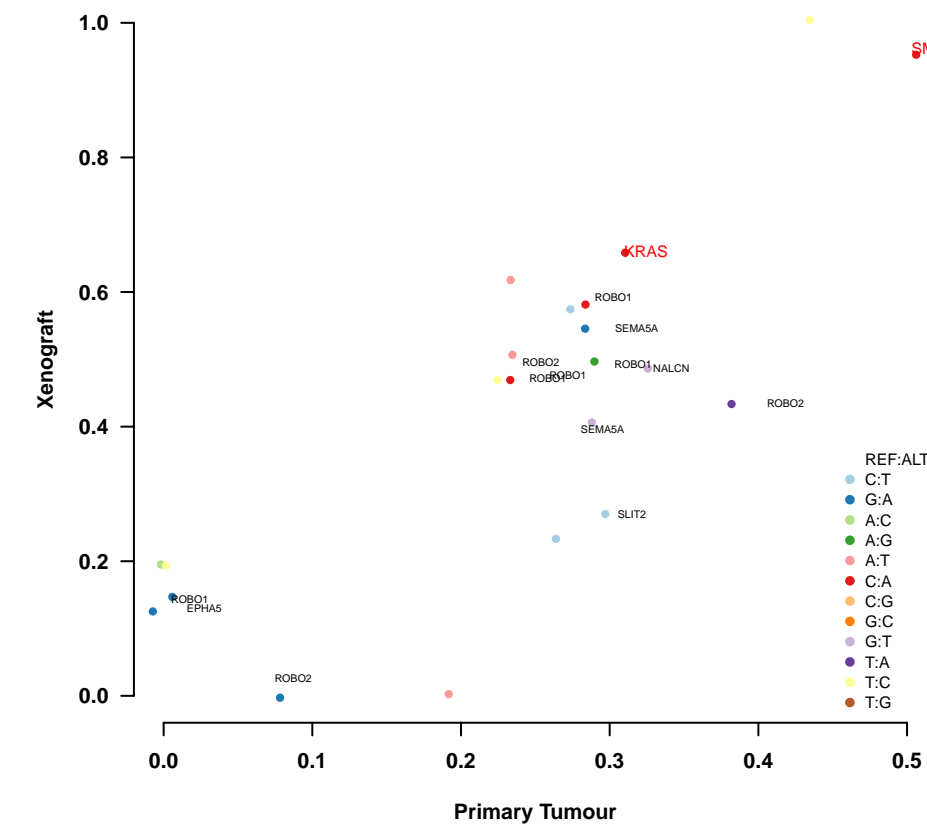

PCSI\_0489

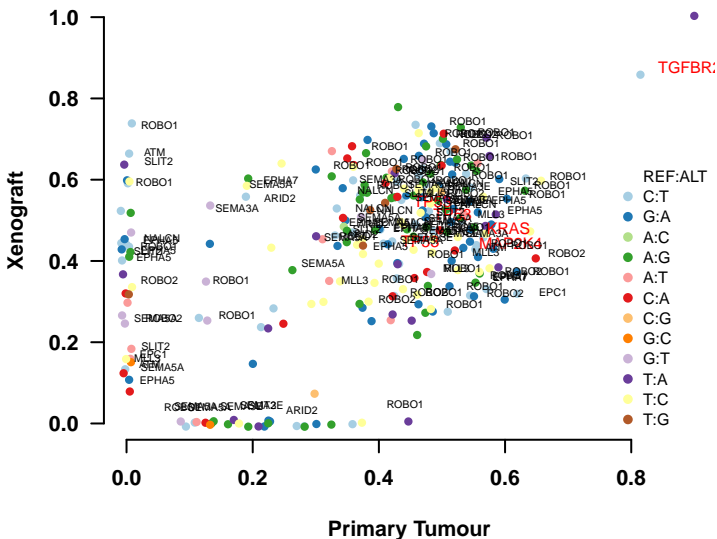

PCSI\_0491

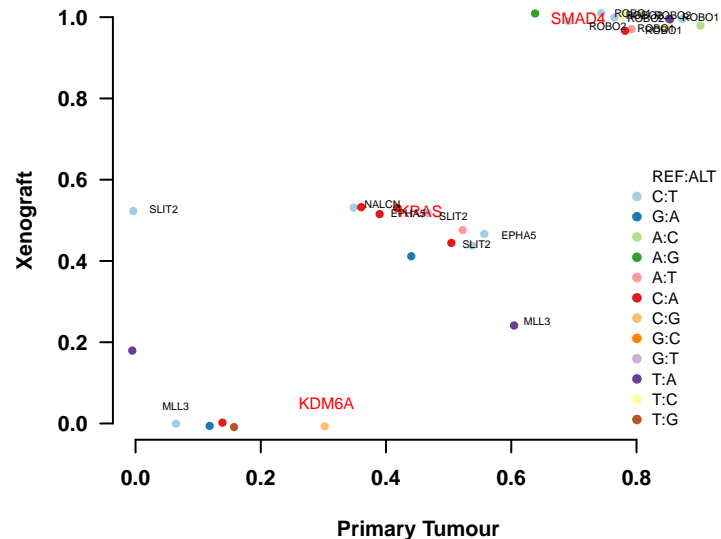

PCSI\_0585

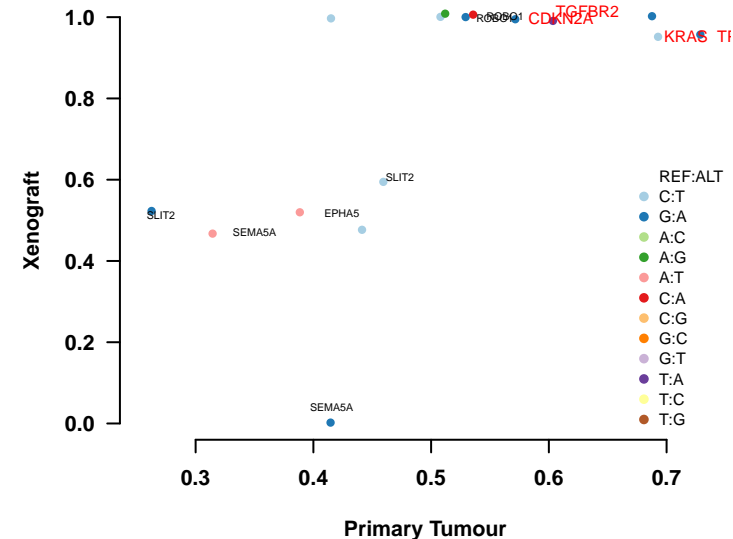

PCSI\_0604

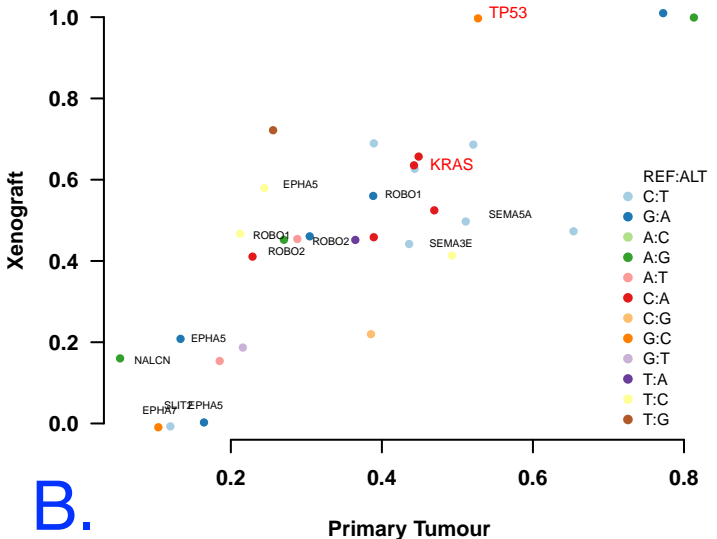

PCSI\_0605

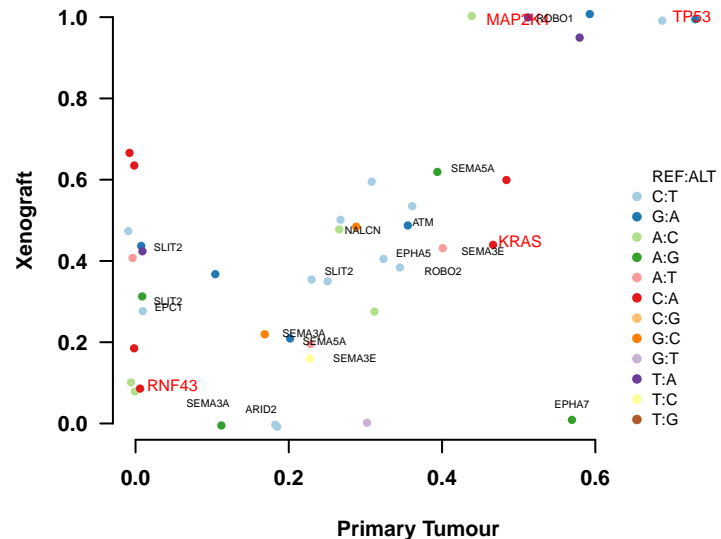

PCSI\_0606

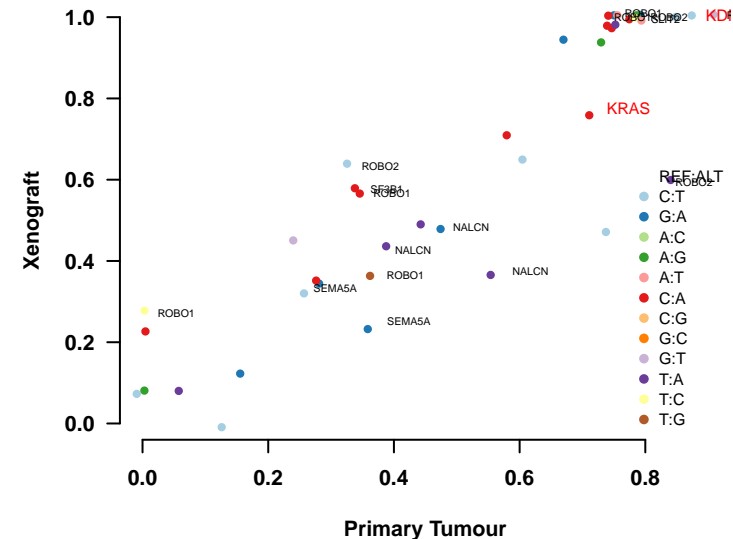

B.

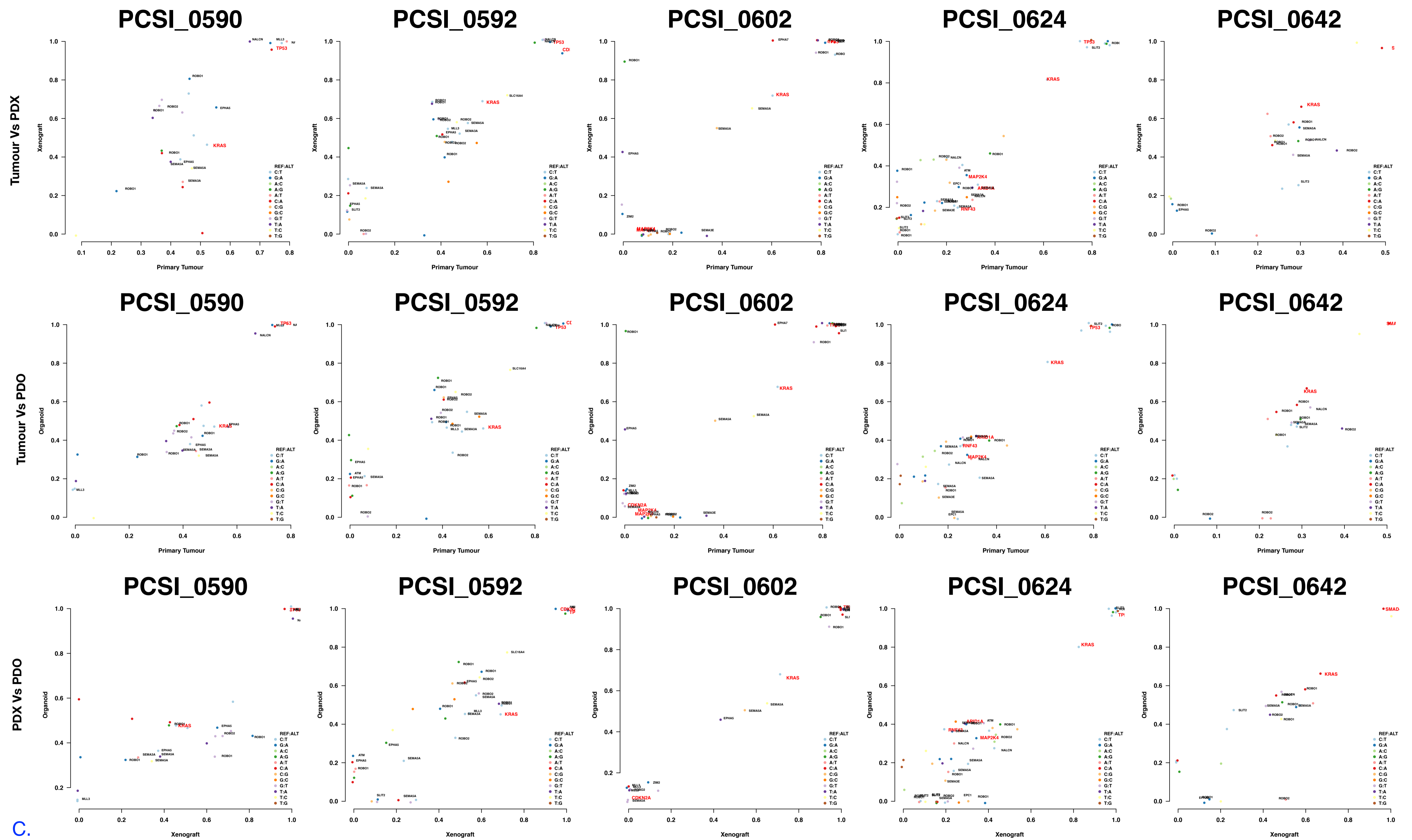

Supplement: S3 Fig — Oncogenes, tumour suppressors, and genes involved pathways of PDAC tumourigenesis are plotted. (A) Frequency of reads carrying the variant allele for primary-PDX pairs. (B) Frequency of reads carrying the variant allele for metastasis-PDX pairs. (C) Frequency of reads carrying the variant allele for the trios. The trio is split into primary-PDX (top), primary-PDO, and PDX-PDO (bottom) pairs. (PDF) [file pcbi.1006596.s003.pdf]

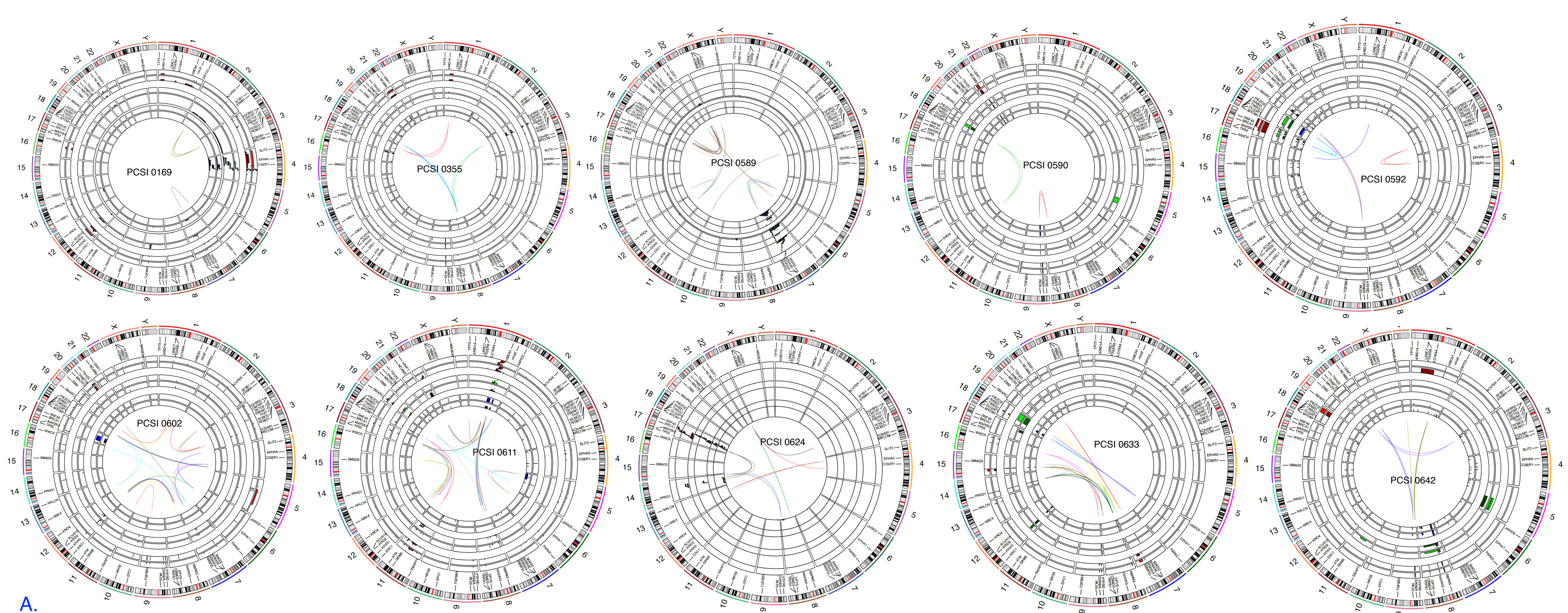

A.

PCSI 0489

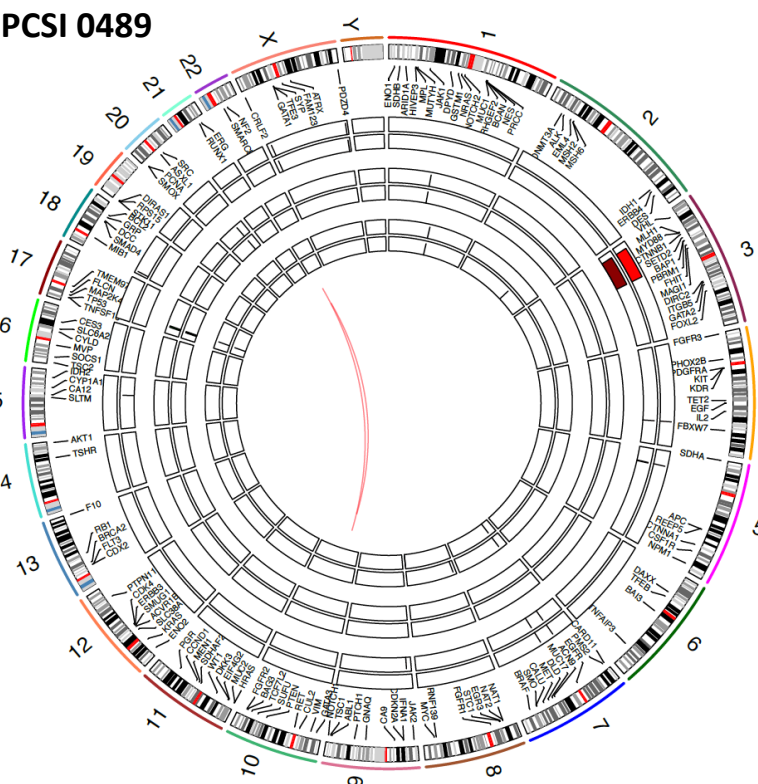

PCSI 0491

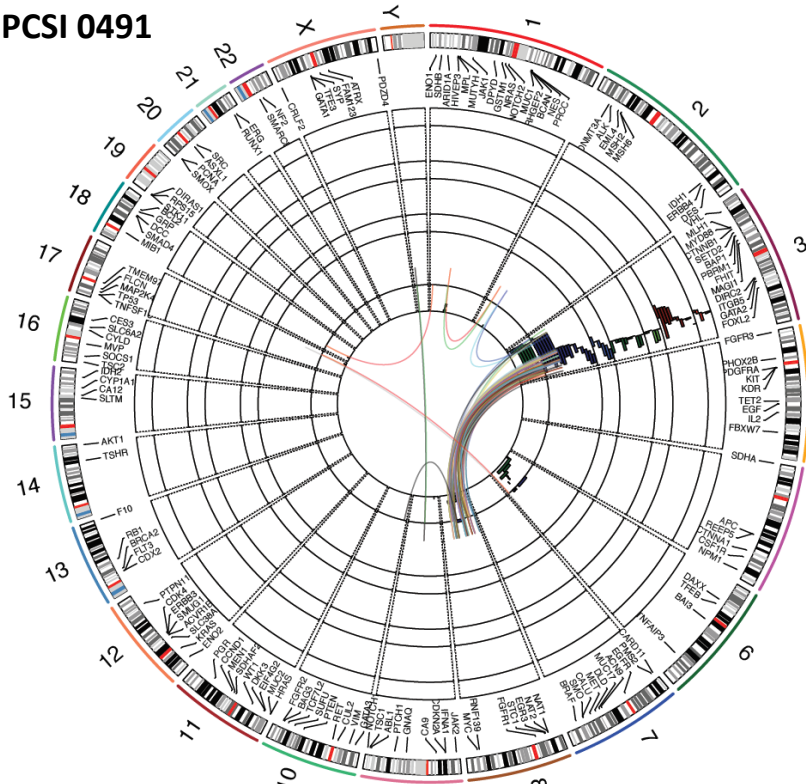

PCSI 0585

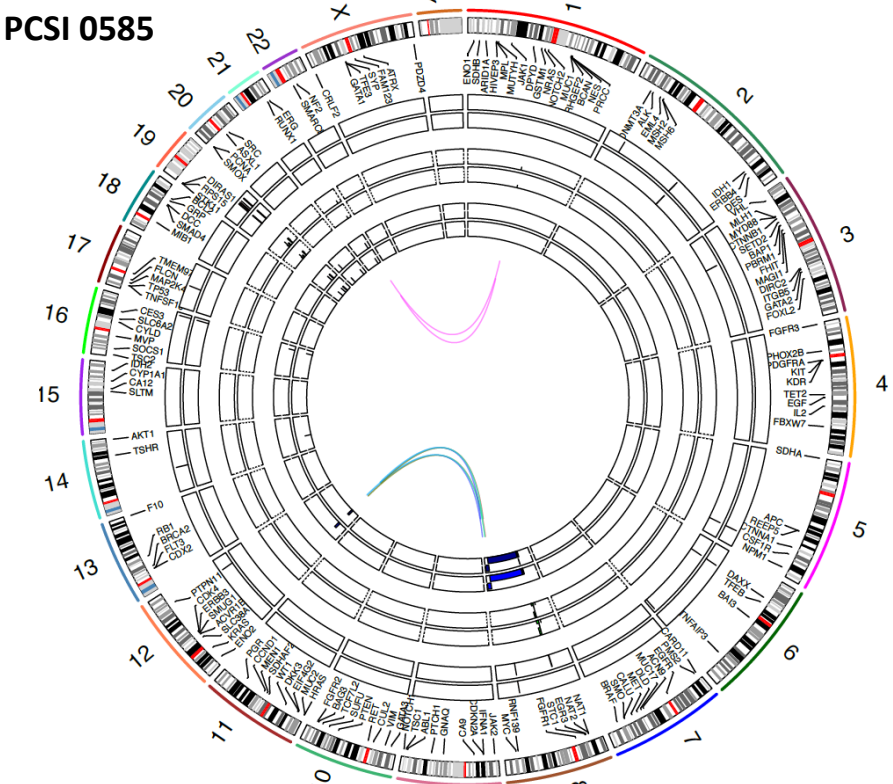

PCSI 0604

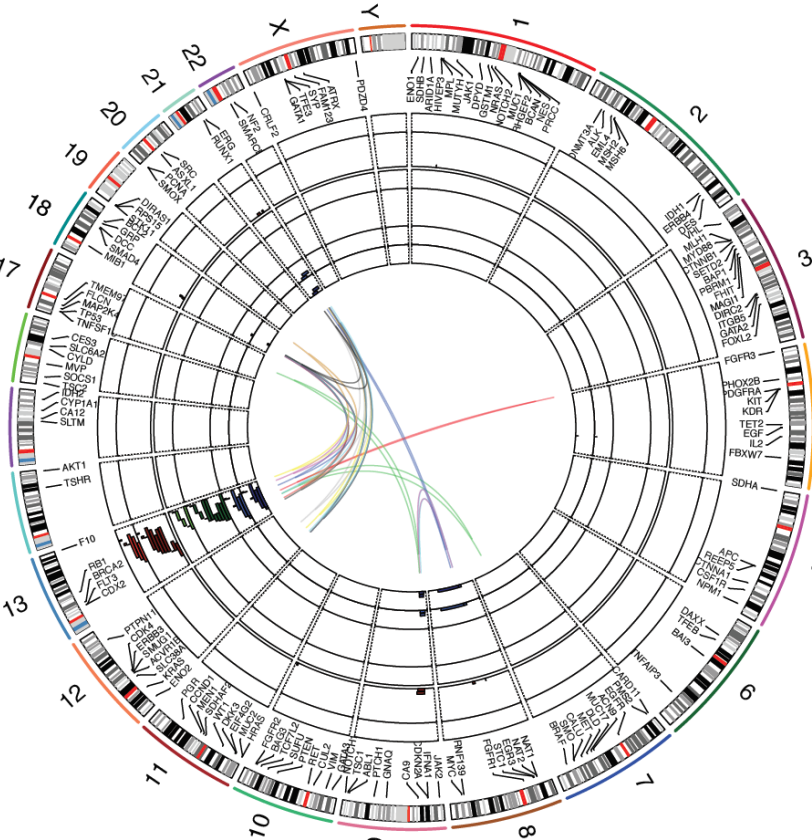

PCSI 0605

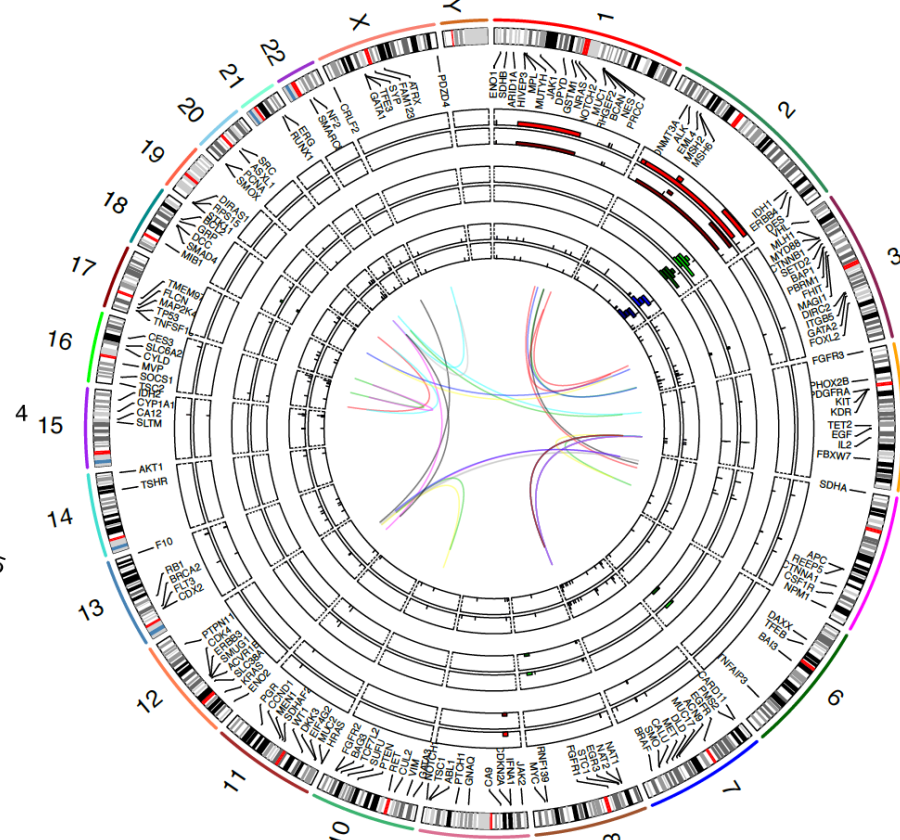

PCSI 0606

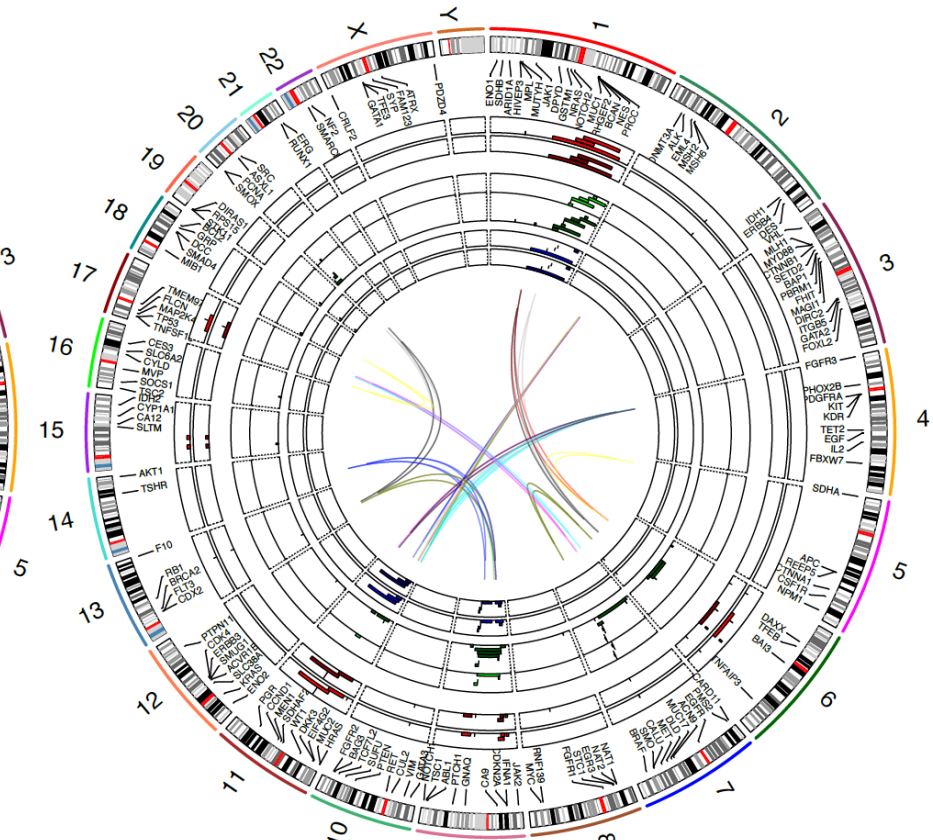

Supplement: S4 Fig — Each type of SV event is color-coded with a similar color between tumours and matching PDX. For each SV type, tumours are annotated on the outer rings of the circos plot and the matching PDX on the inner rings. SV events are colored as follows: deletions (red), inversions (green), and duplications (blue). Translocation events between chromosomes are also depicted (center). (A) Circos plots of SV events across the genomes of 10 primary-PDX pairs (B) Circos plots of SV events across the genomes of 6 metastasis-PDX pairs. (PDF) [file pcbi.1006596.s004.pdf]

PRIMARY PDAC

A.

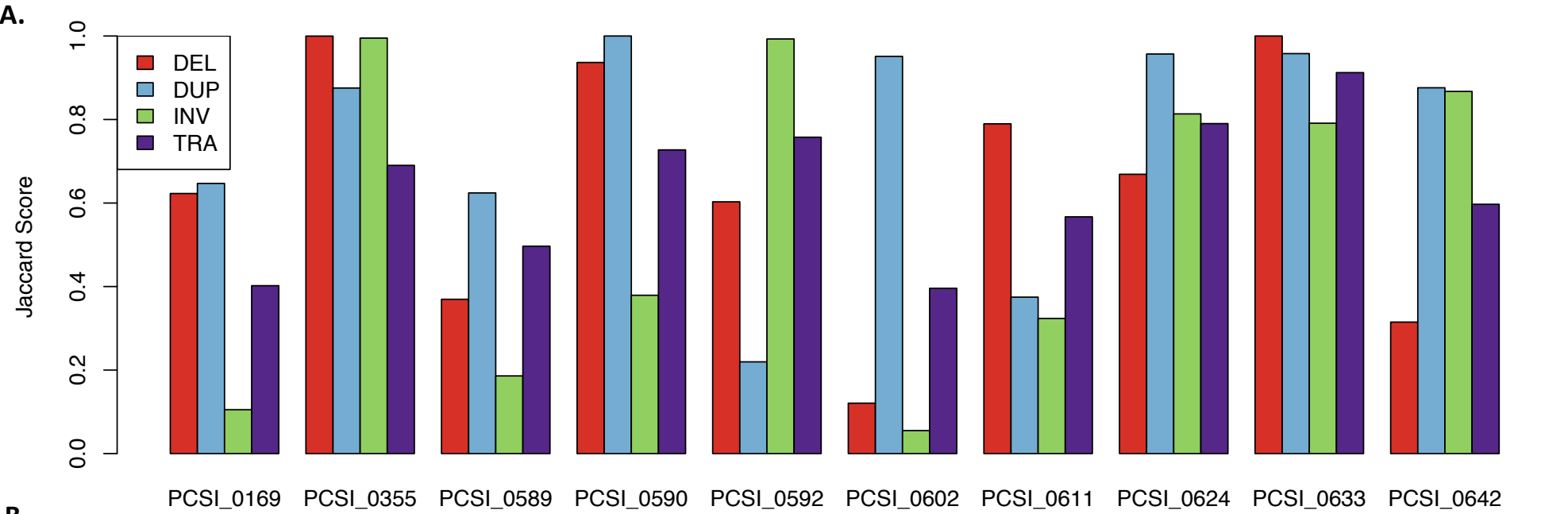

B.

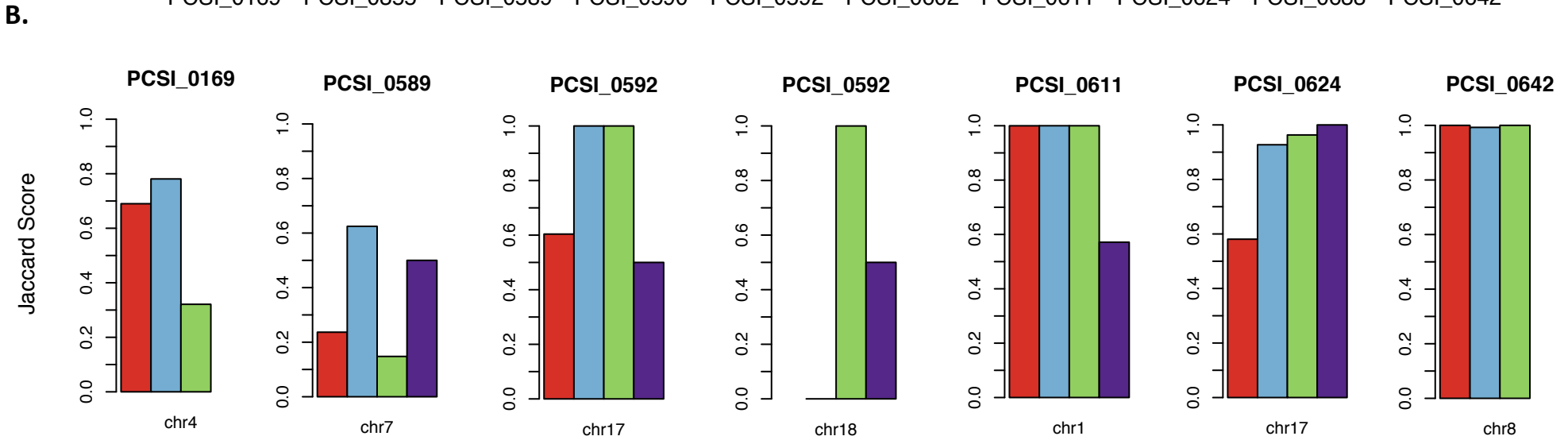

LIVER METASTASIS

C.

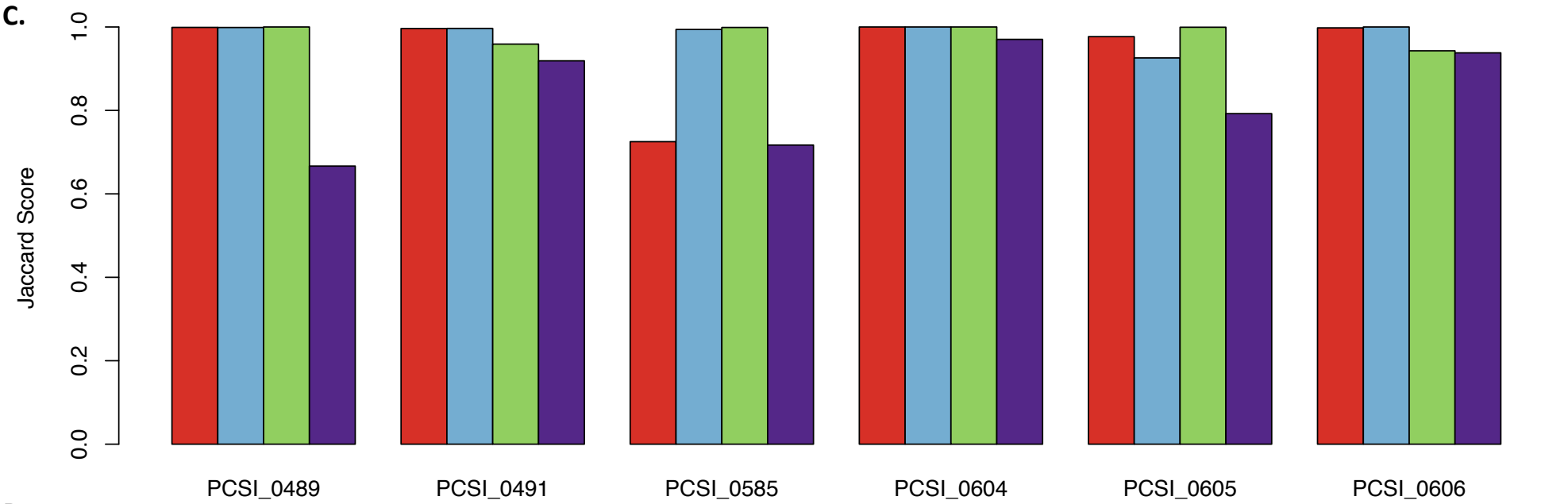

D.

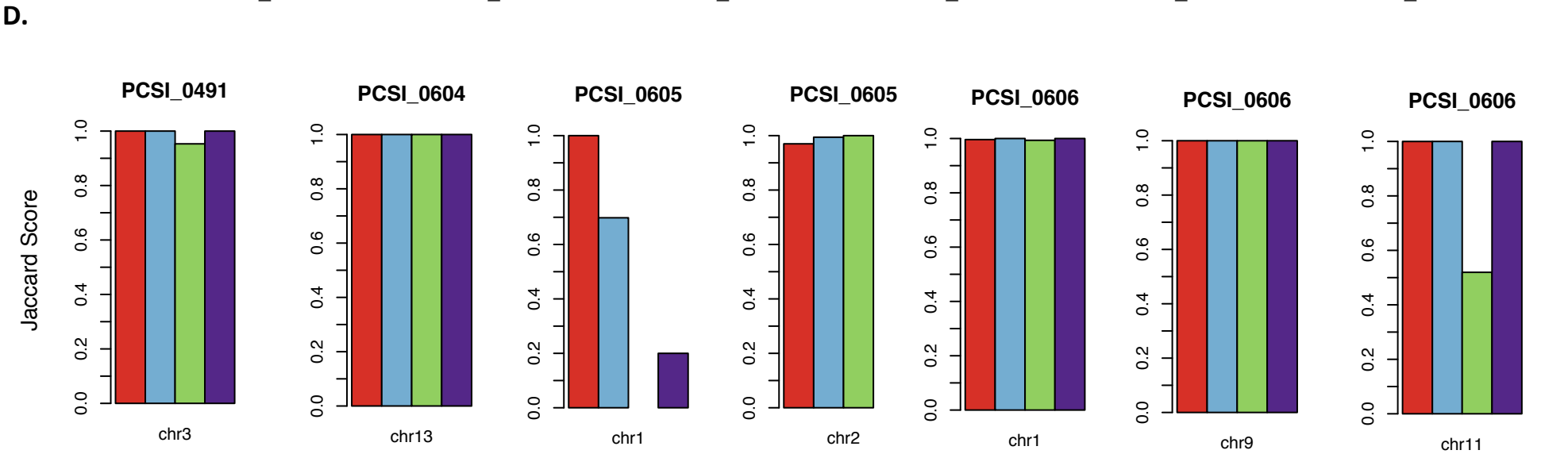

Supplement: S5 Fig — (A) Genome-wide jaccard scores, by SV category, for primary resected tumours and matched PDX. (B) Chromosome-specific jaccard scores, per SV category, for chromosomes exhibiting clustered SV events in primary resected tumours and matched PDX (C) Genome-wide jaccard scores, by SV category, for metastases and matched PDX (D) Chromosome-specific jaccard scores, per SV category, for chromosomes exhibiting clustered SV events in metastases and matched PDX. (PDF) [file pcbi.1006596.s005.pdf]

PCSI 0590

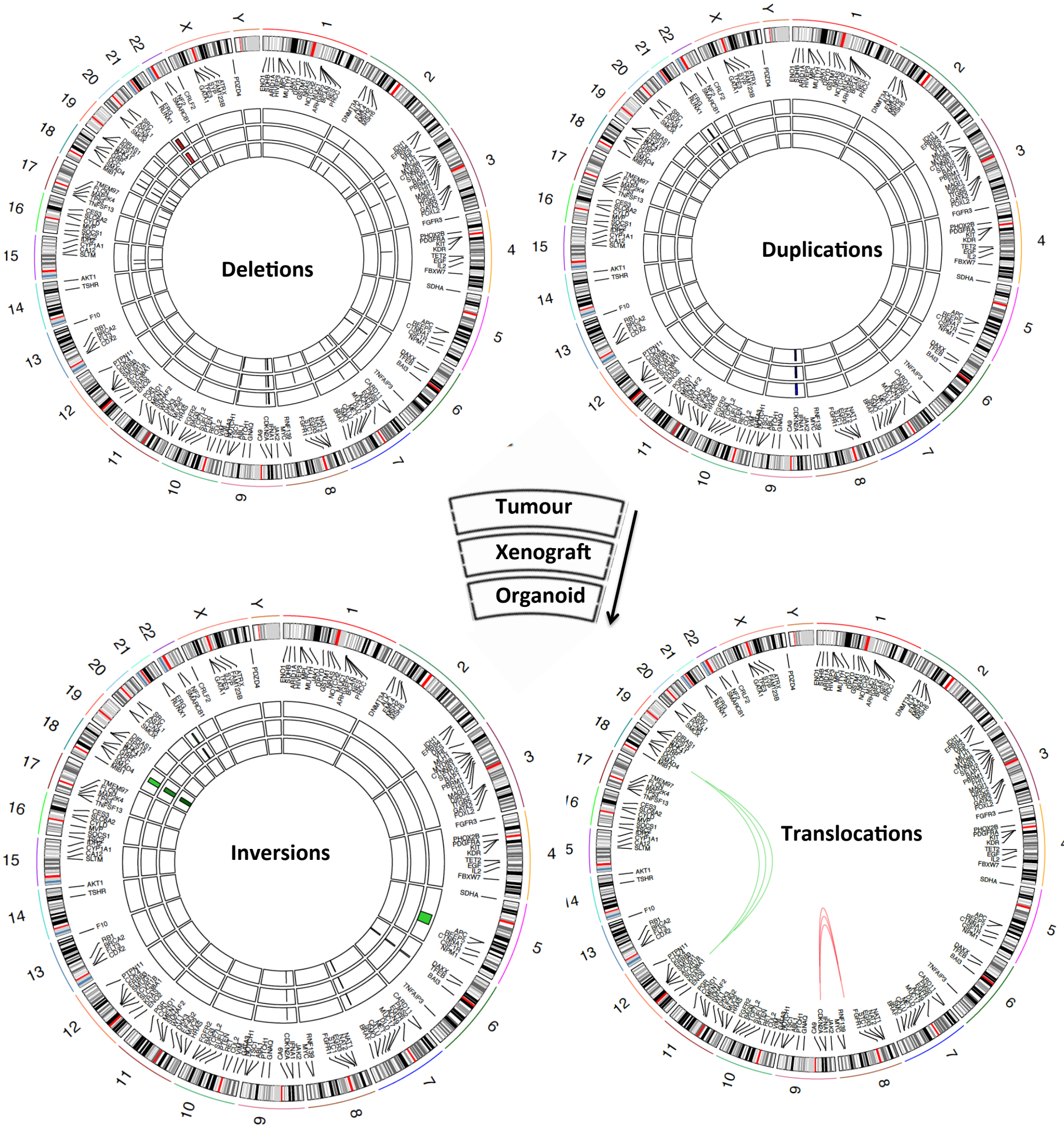

PCSI 0592

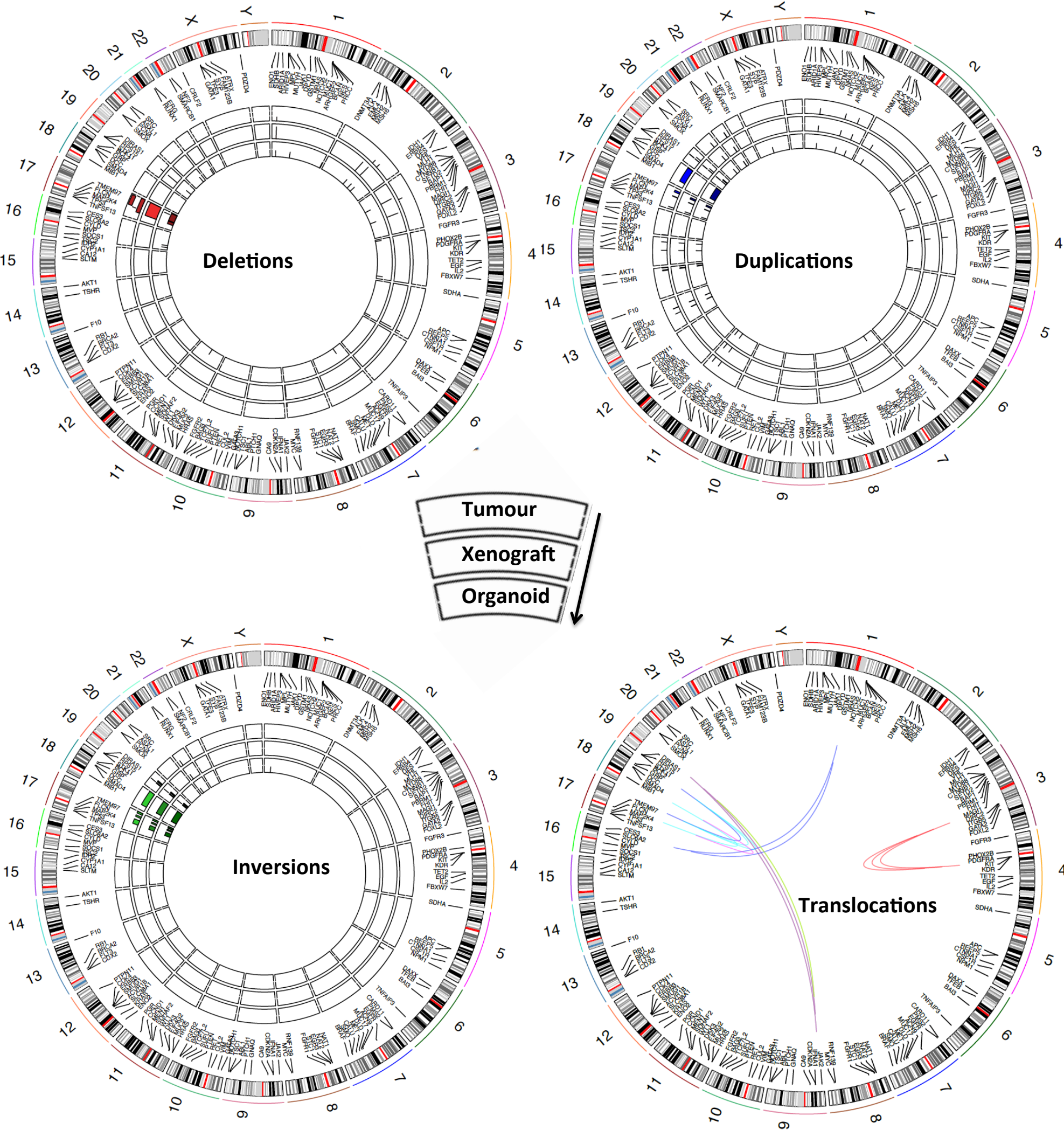

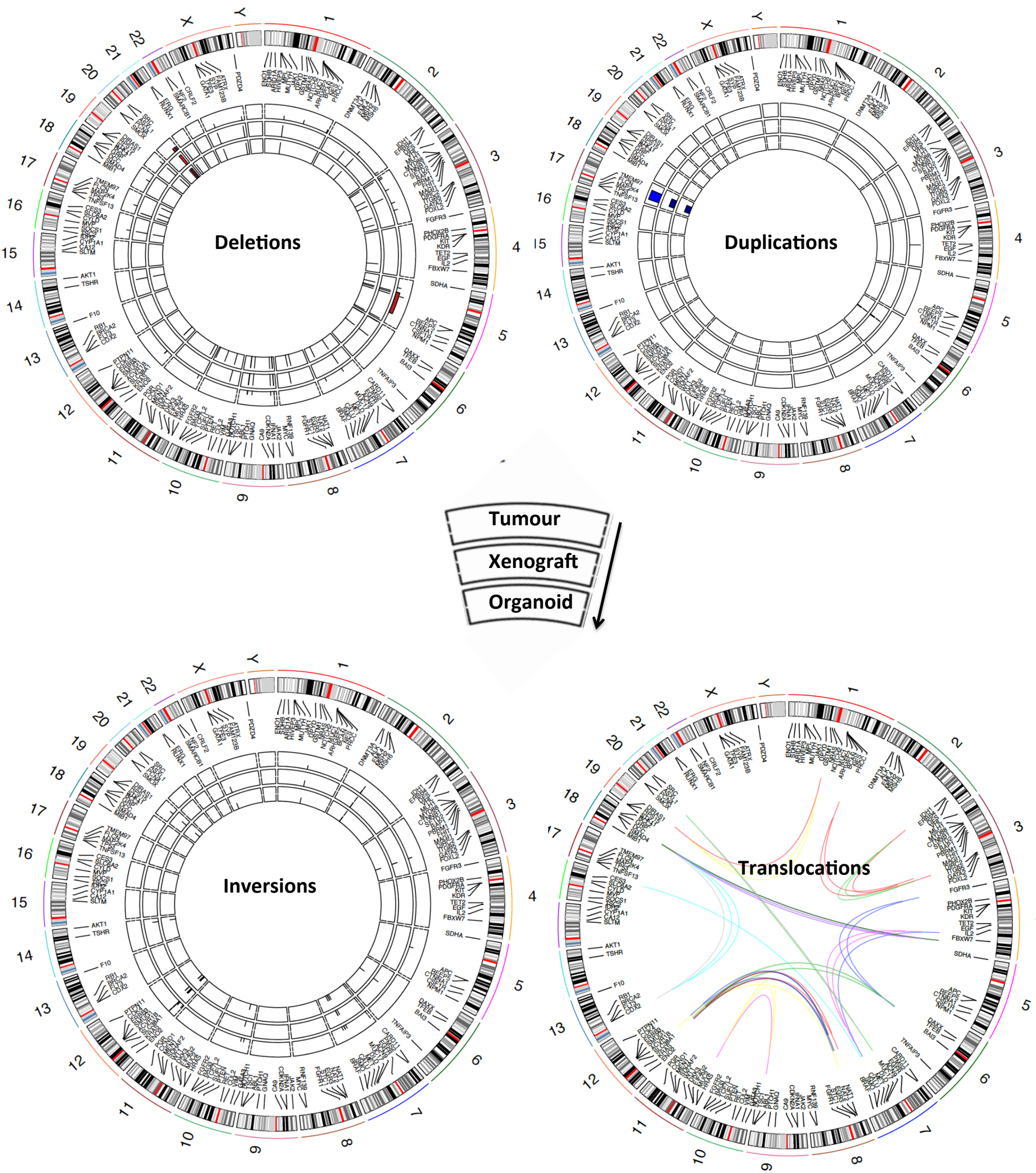

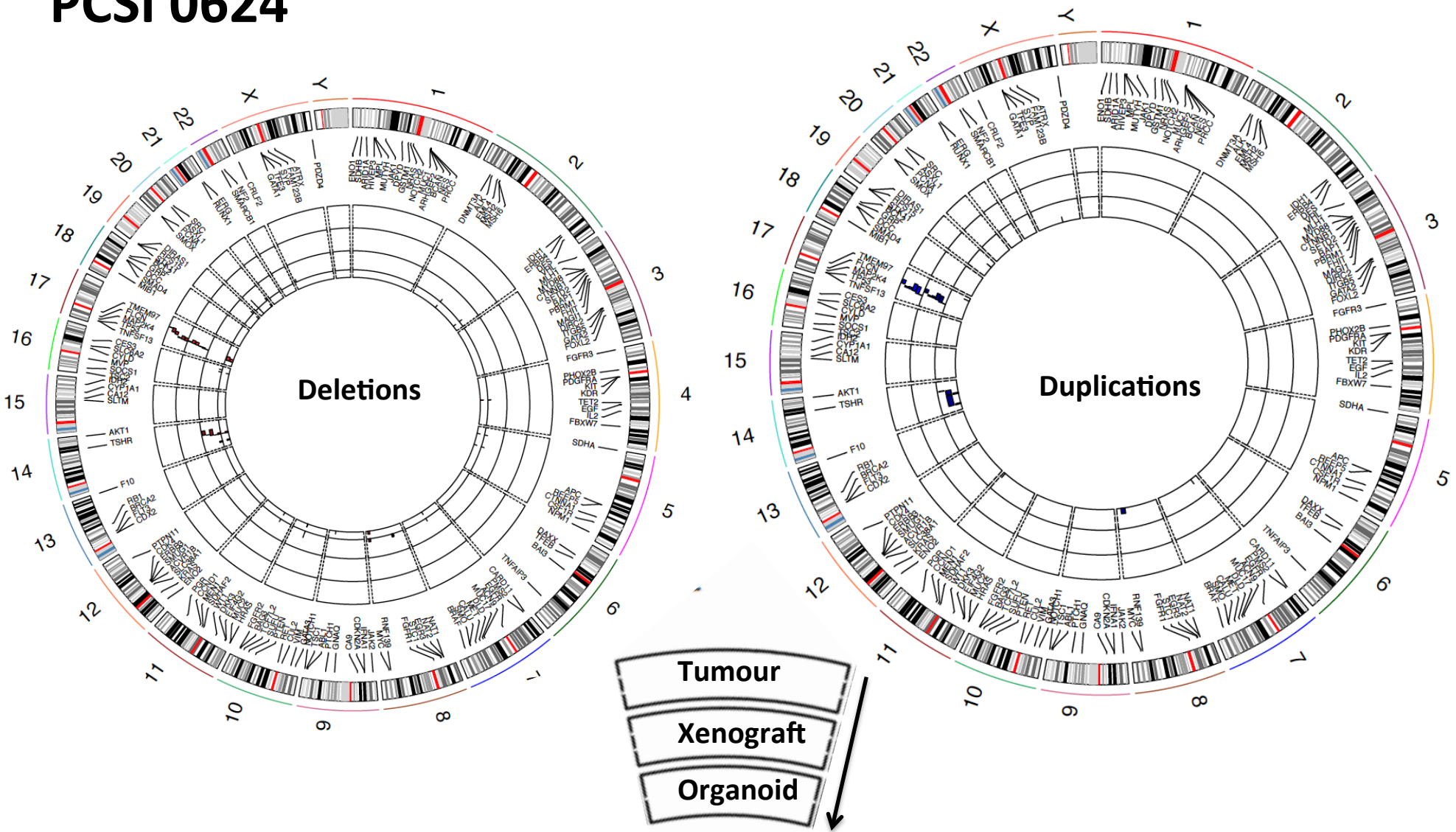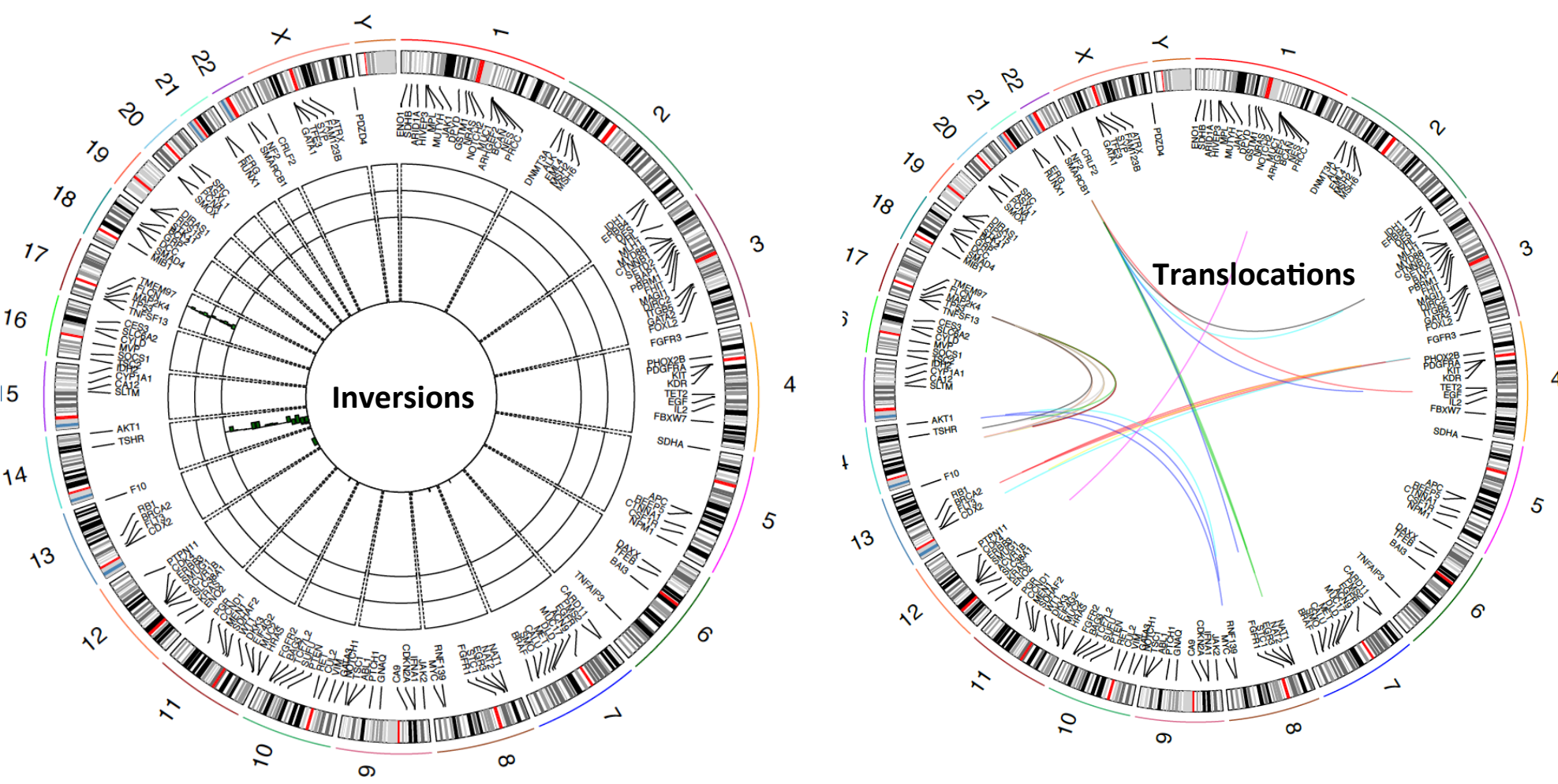

PCSI 0642

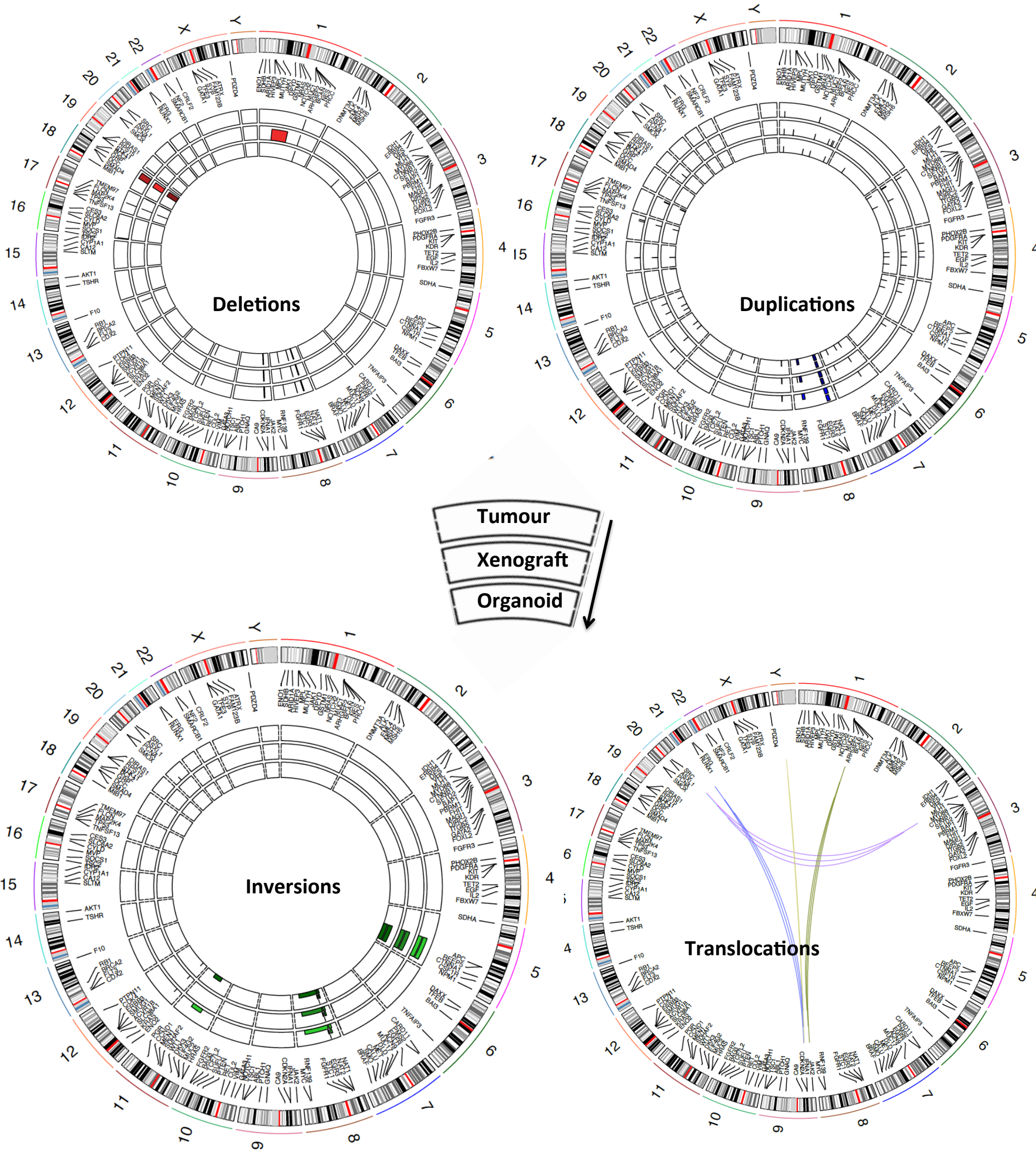

Supplement: S6 Fig — Each type of SV (deletion, inversion, duplication, and translocation) is represented as one circos plot, with 3 rings indicating tumour (outer), PDX (middle), and PDO (inner). SV events are colored as follows: deletions (red), inversions (green), and duplications (blue). Translocation events between chromosomes are also depicted (center). (PDF) [file pcbi.1006596.s006.pdf]

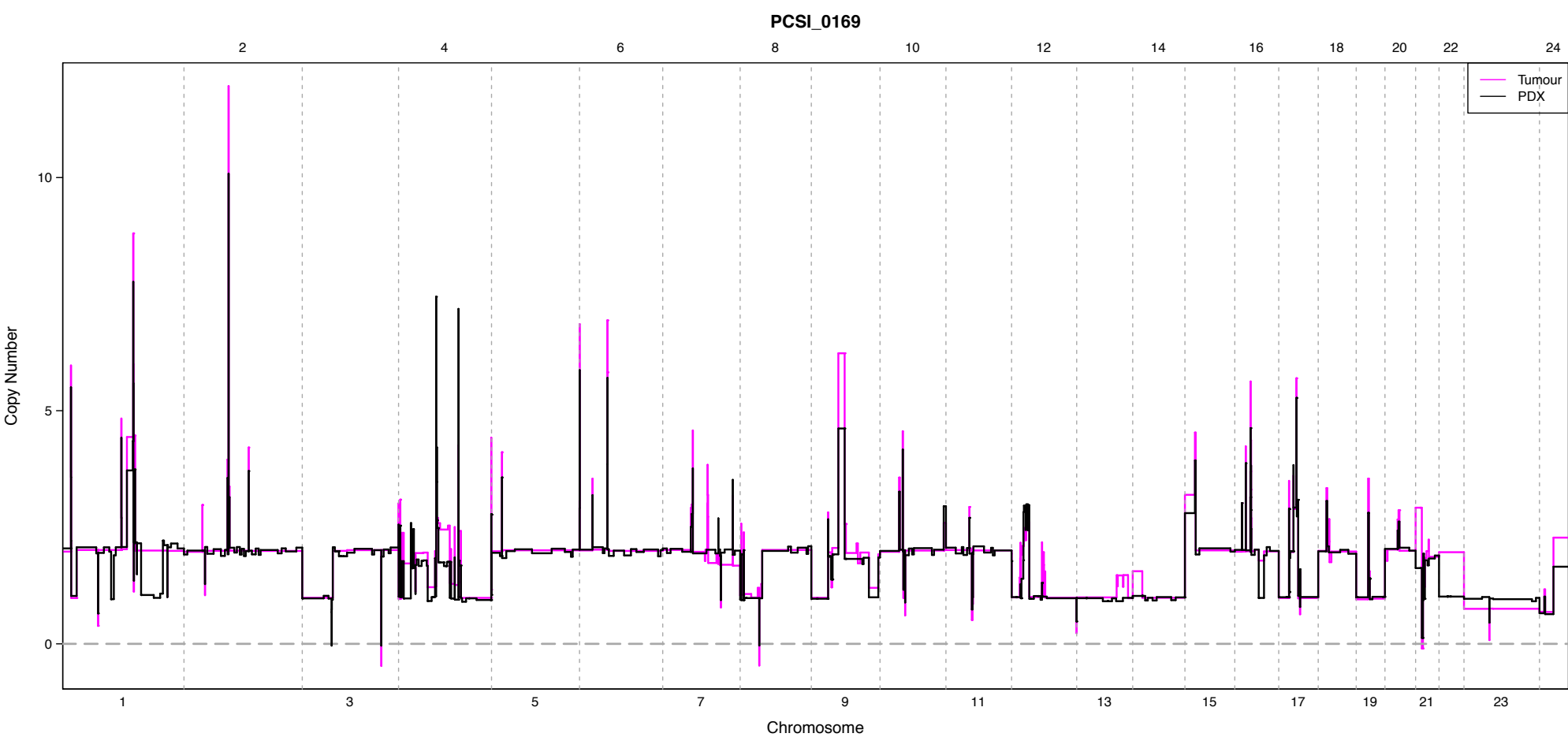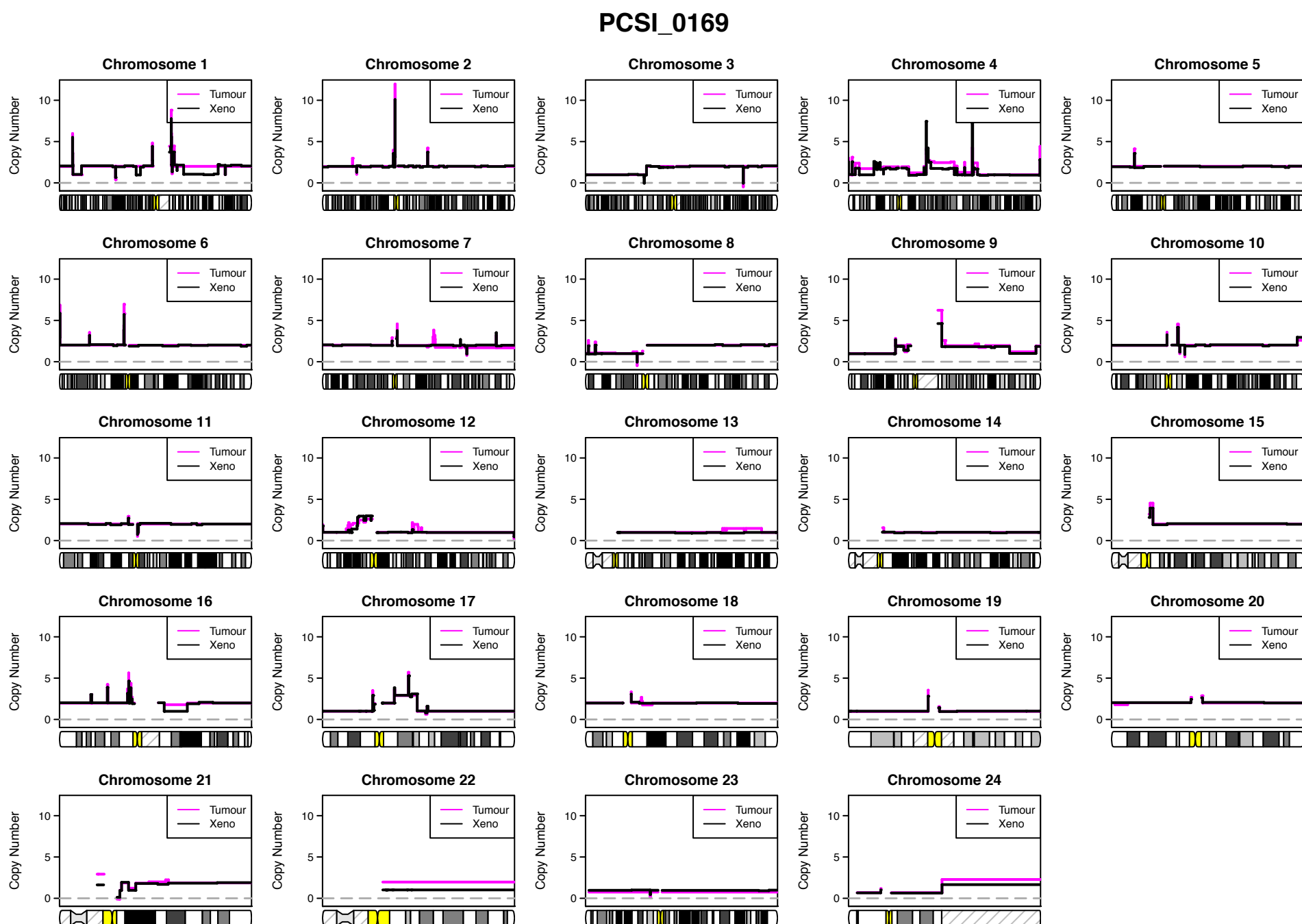

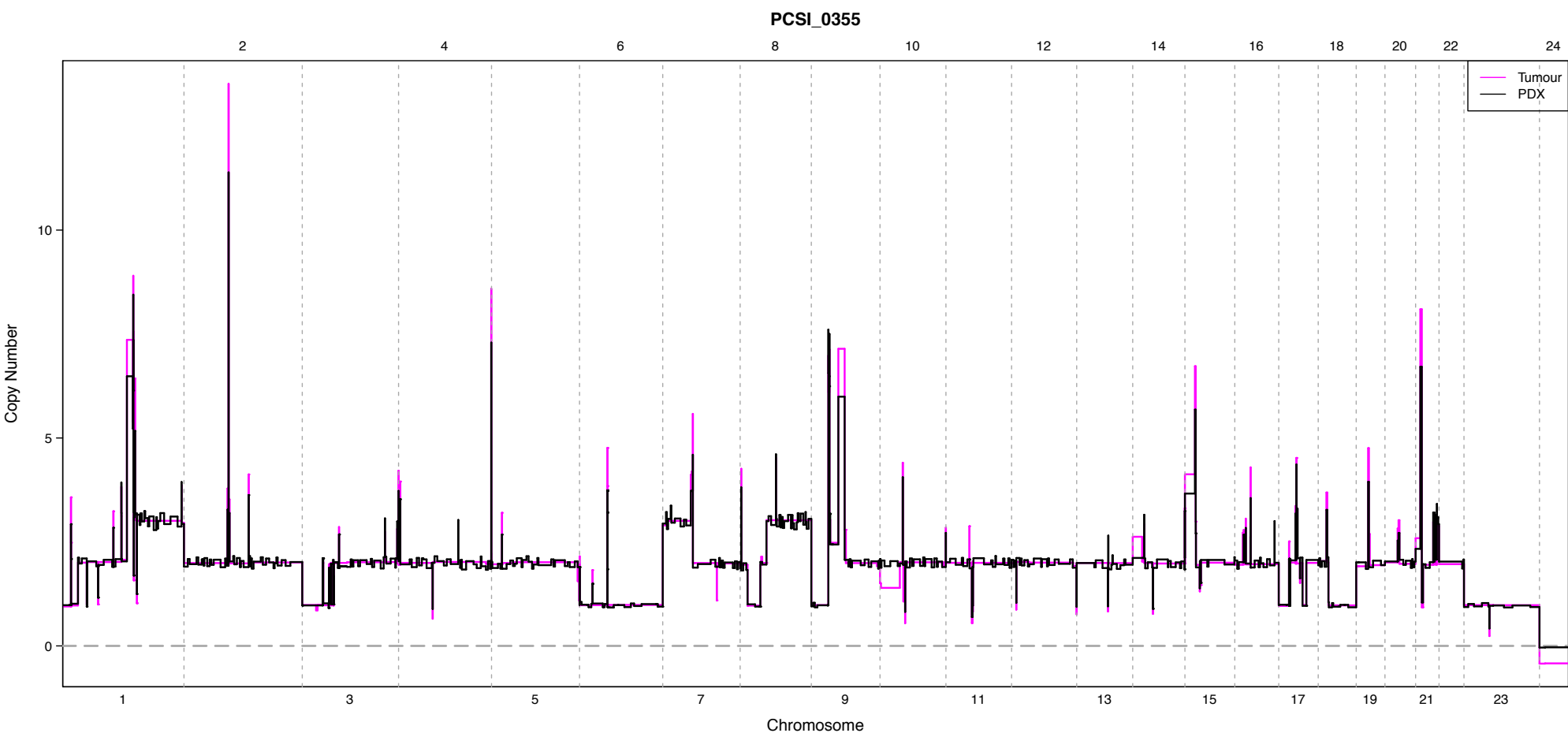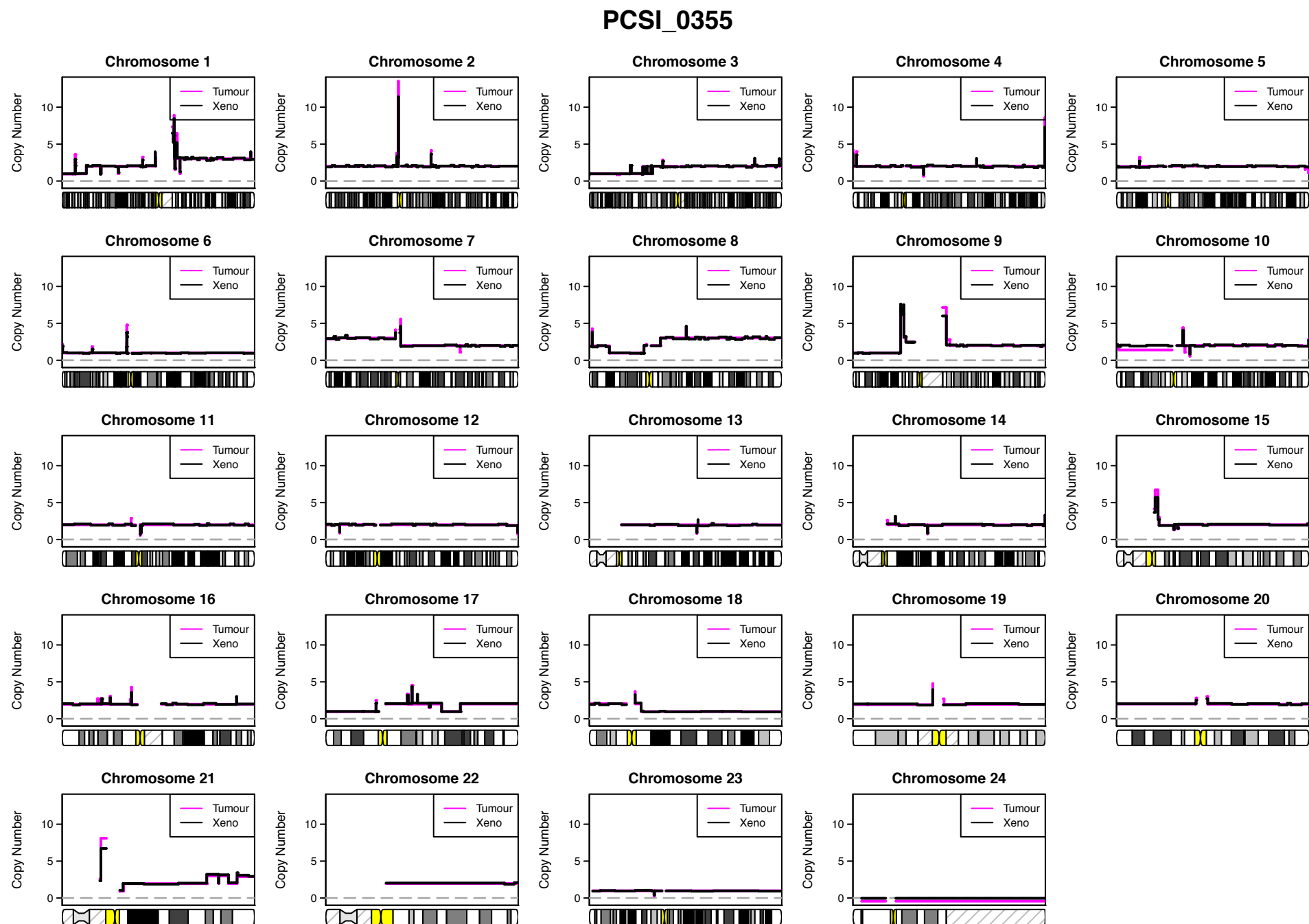

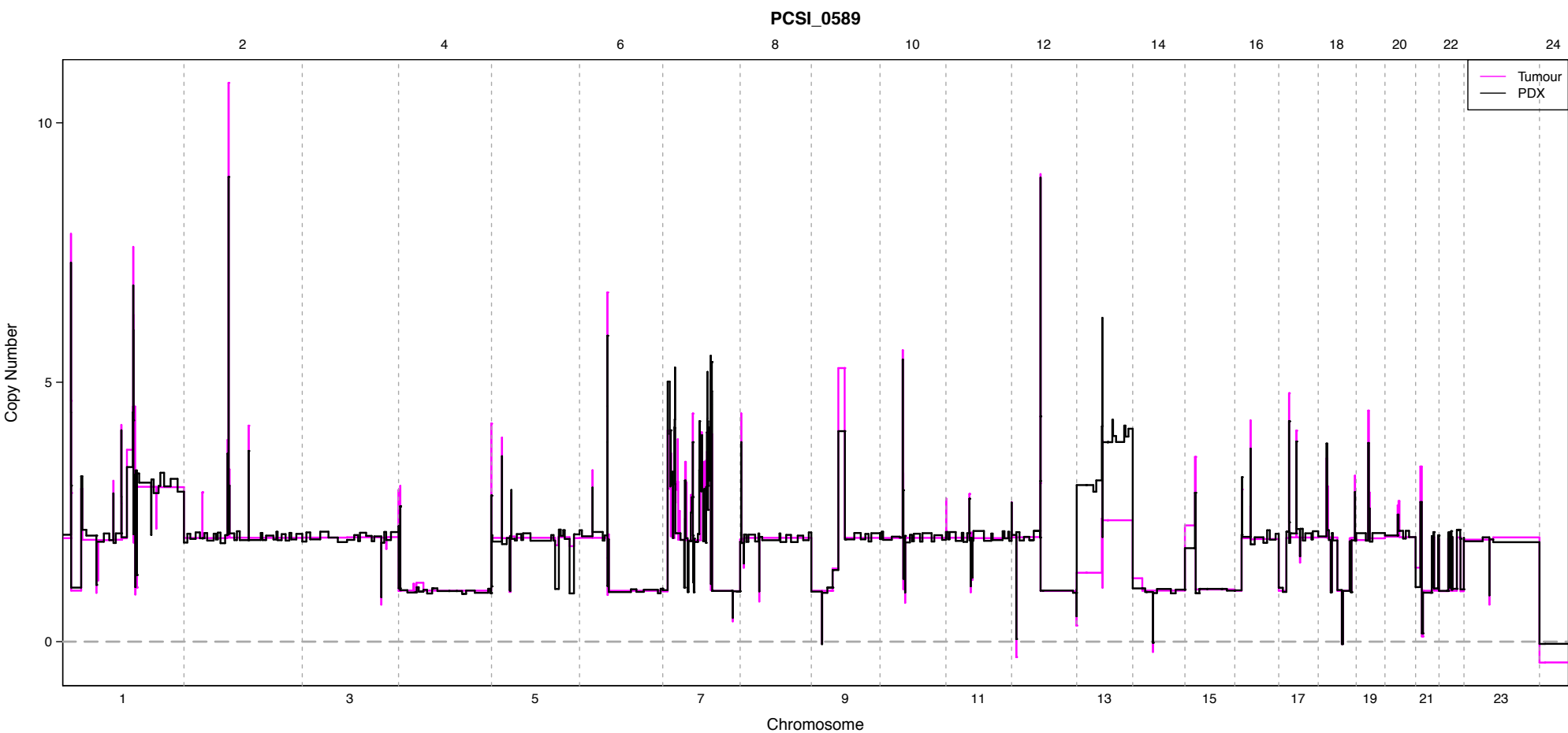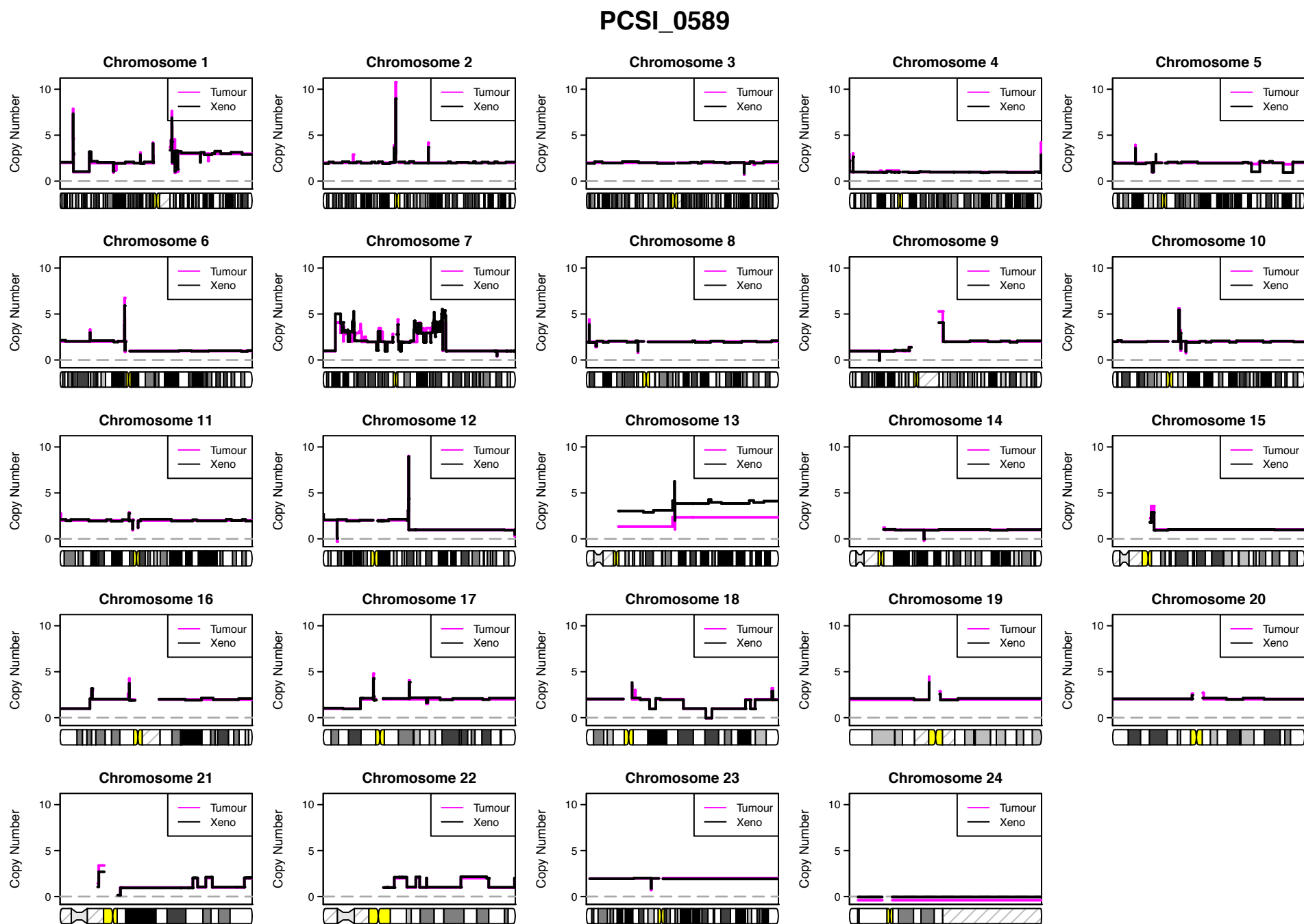

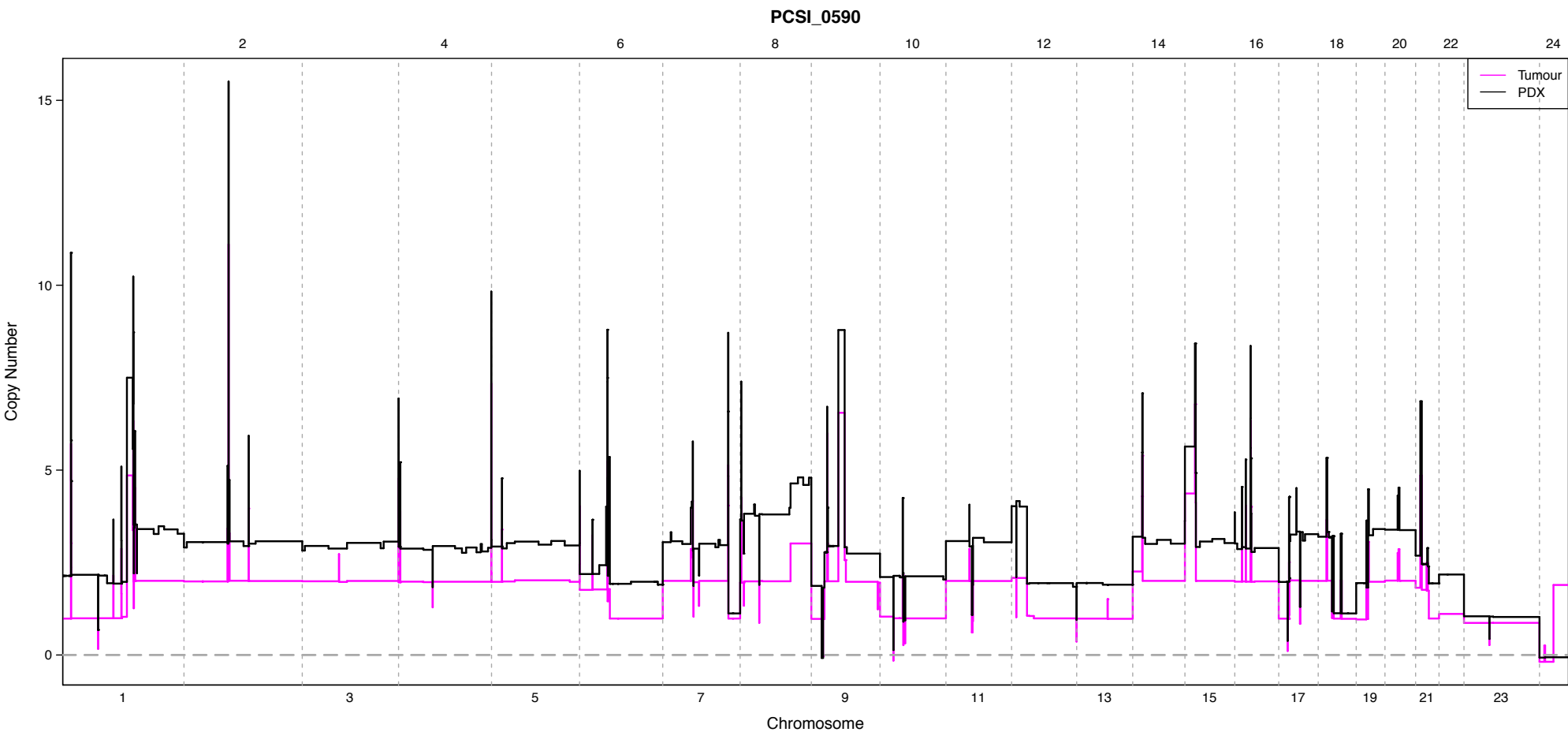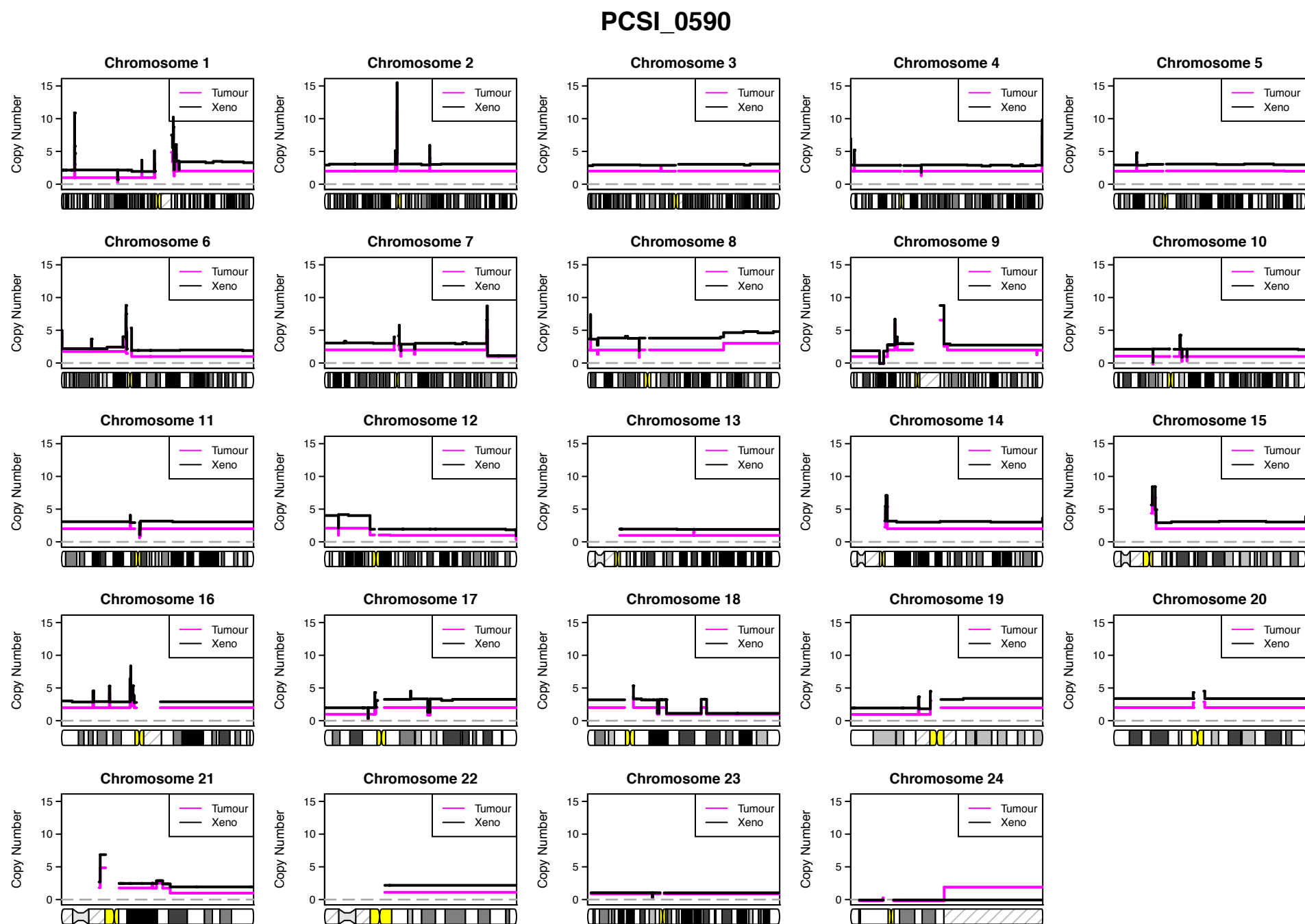

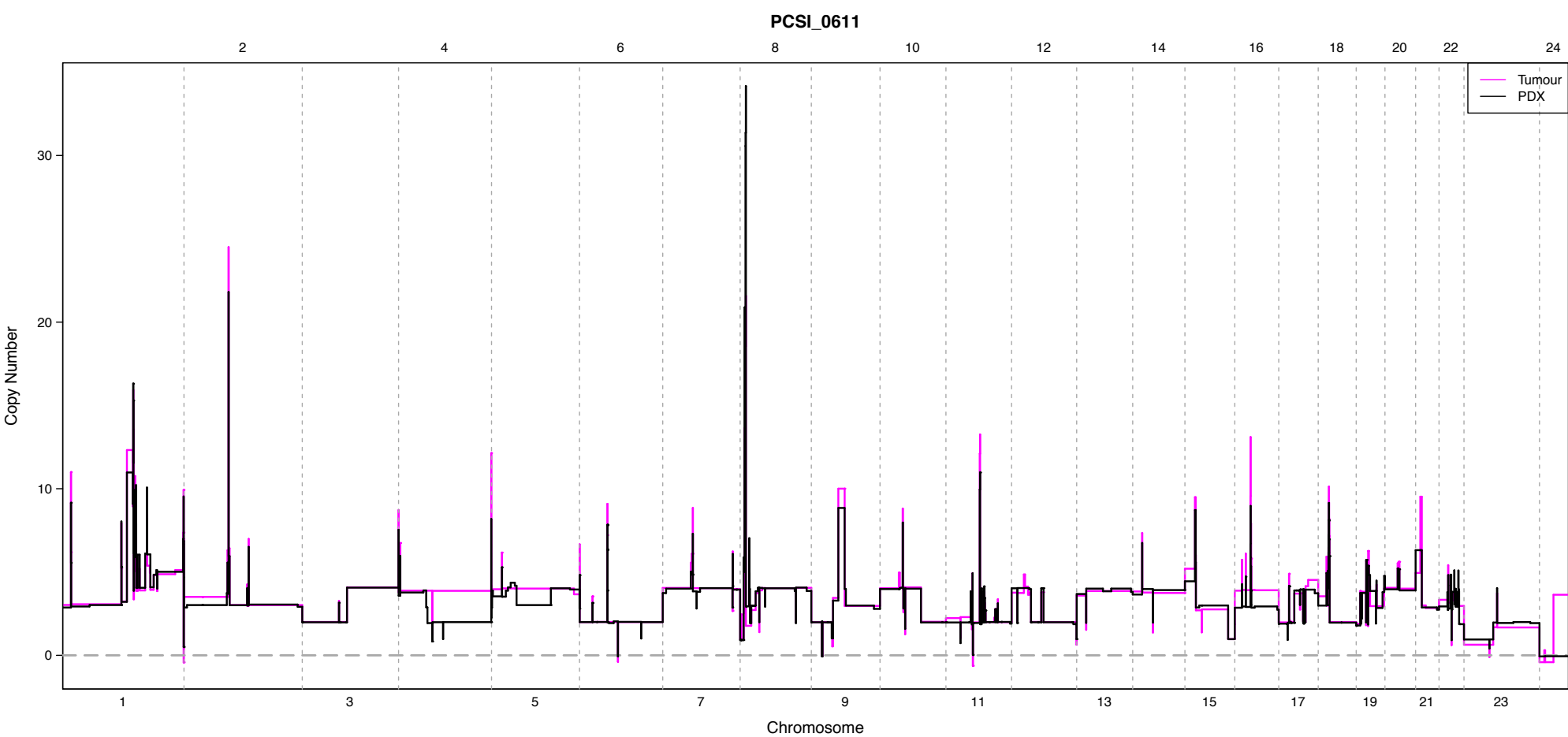

**PCSI\_0611**

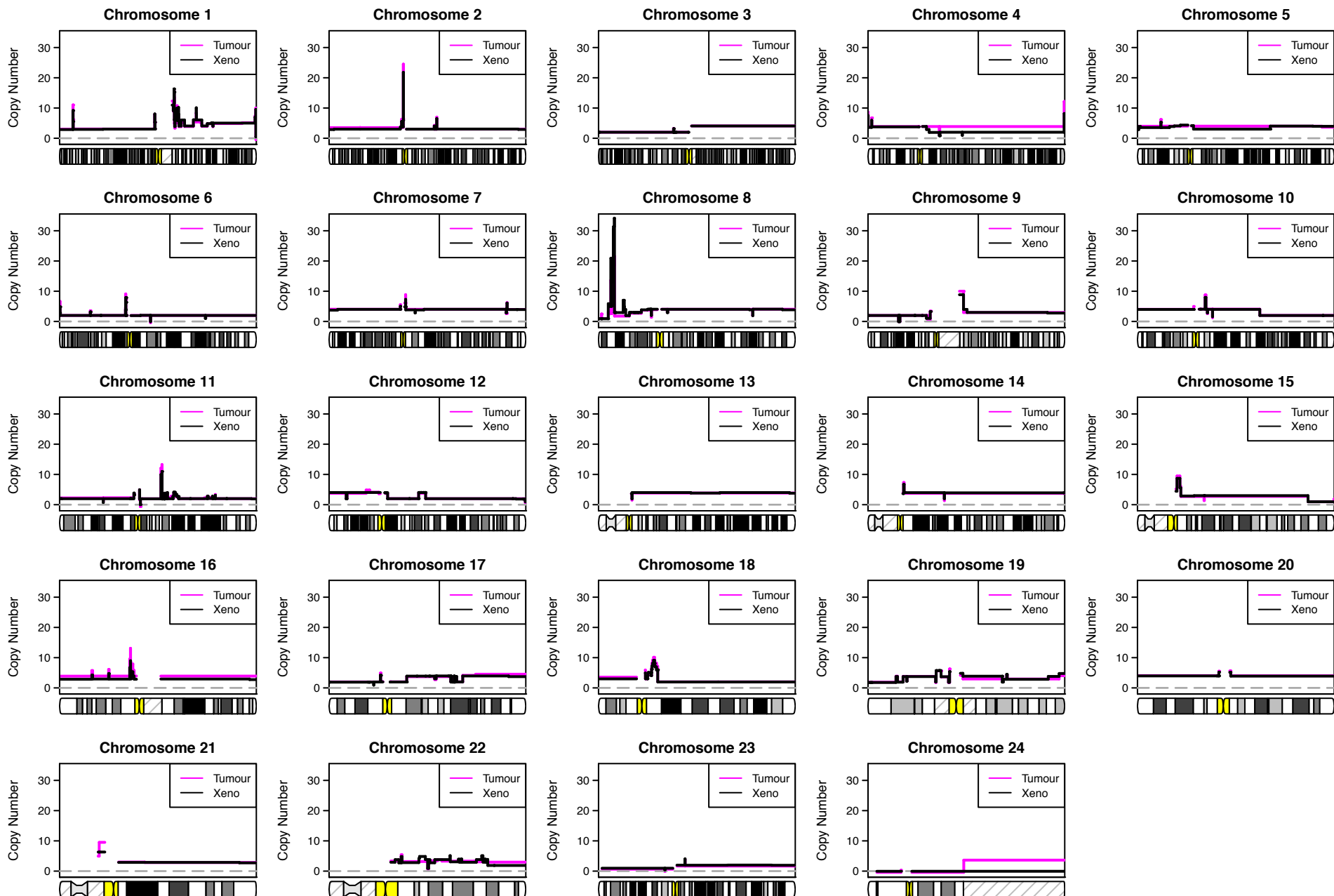

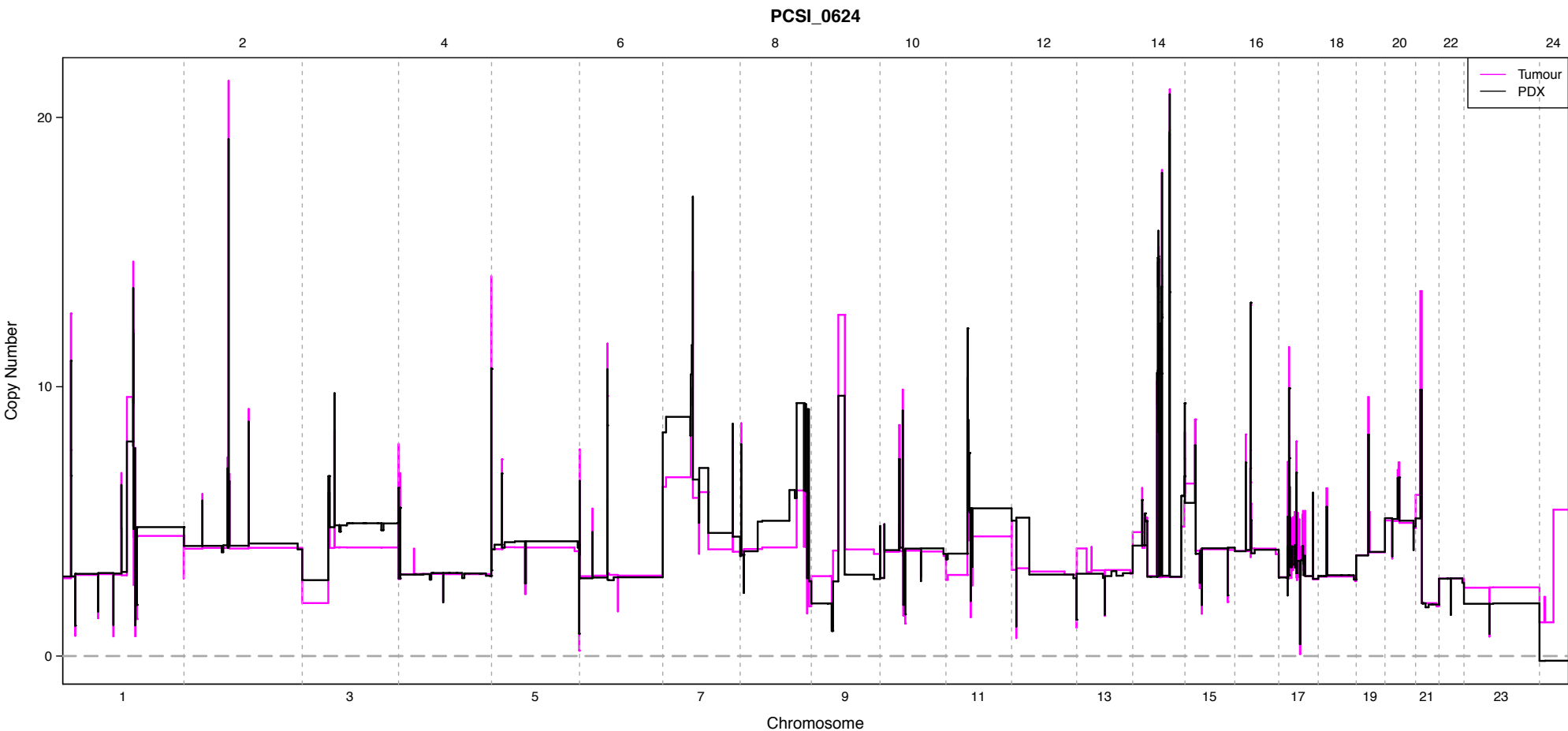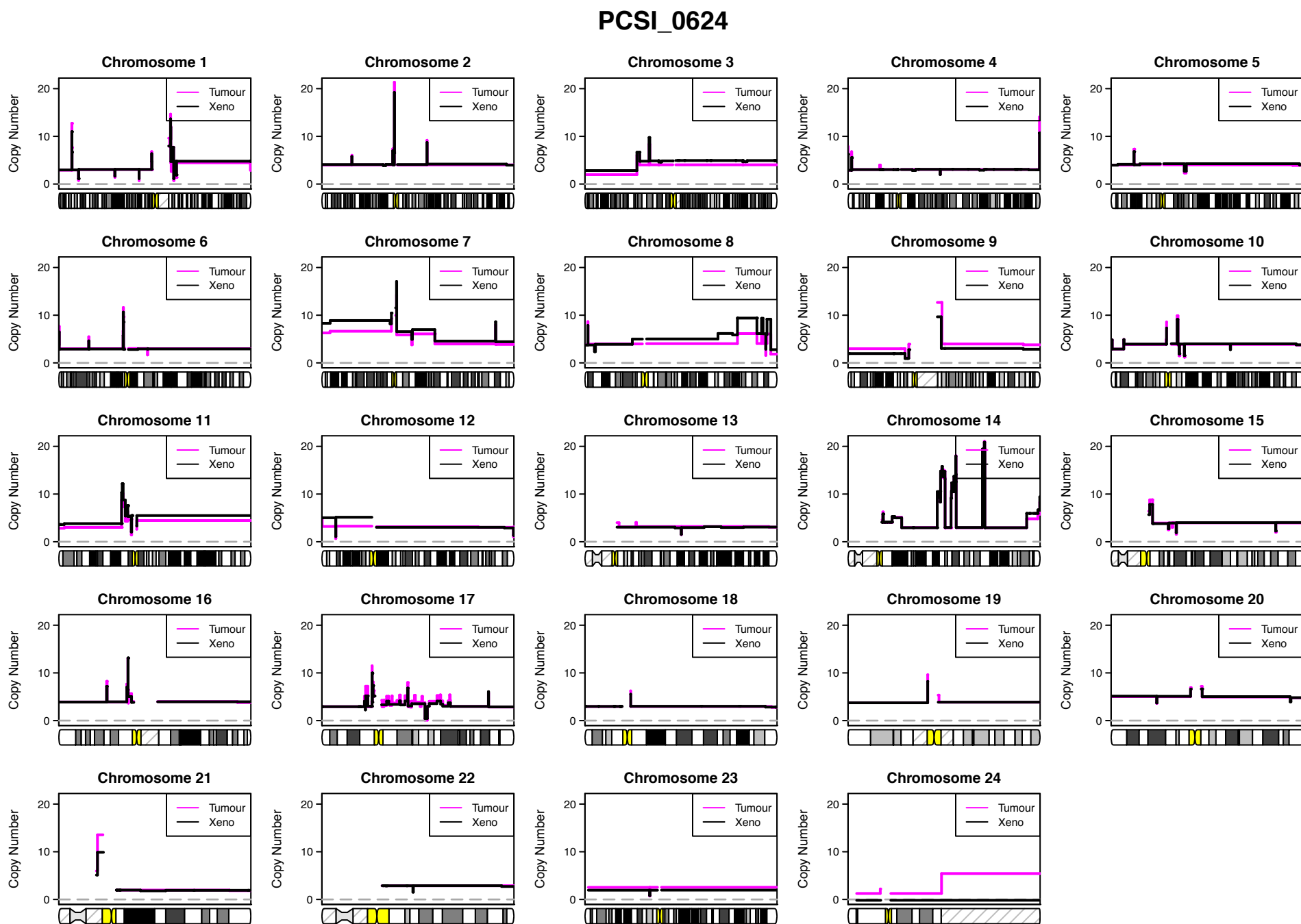

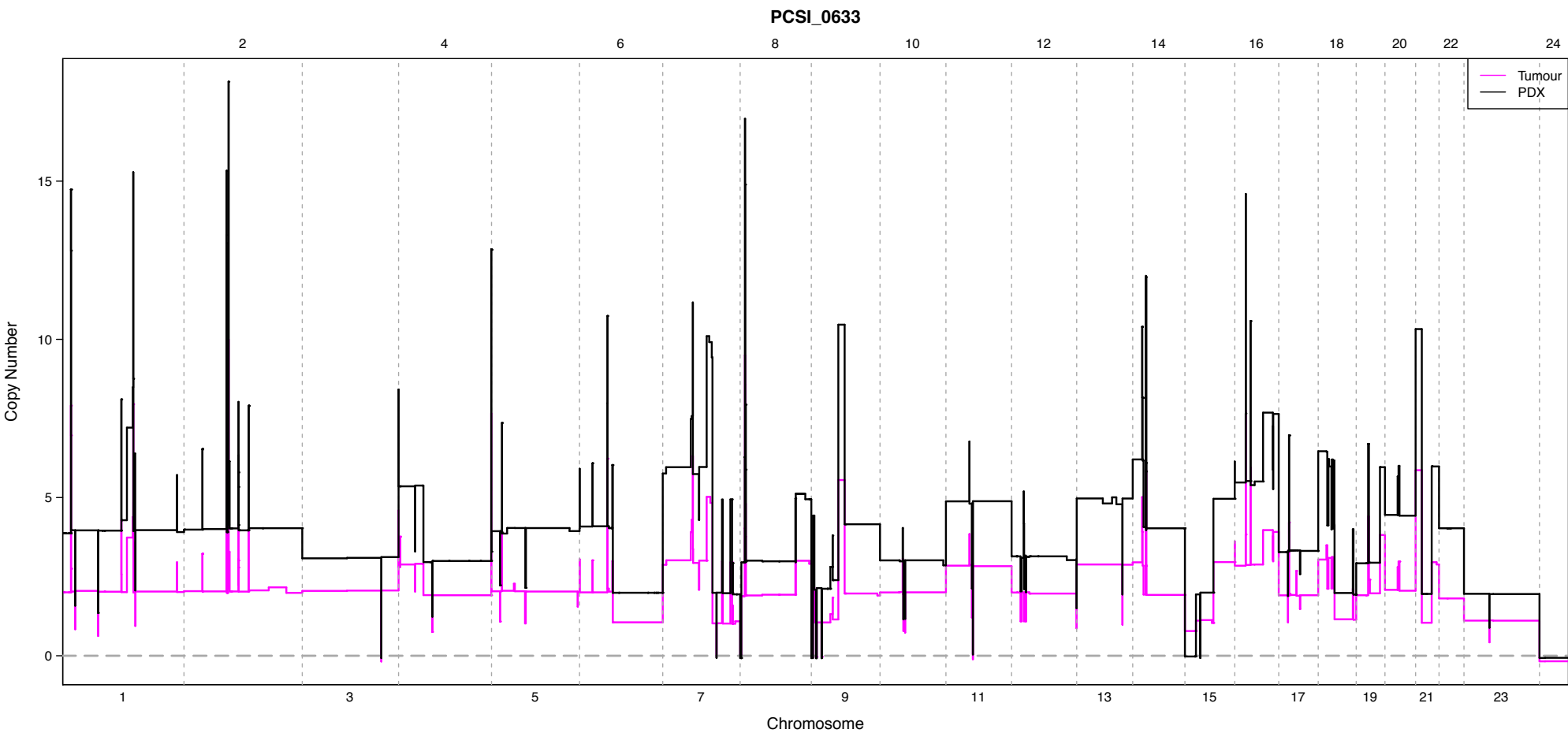

**PCSI\_0633**

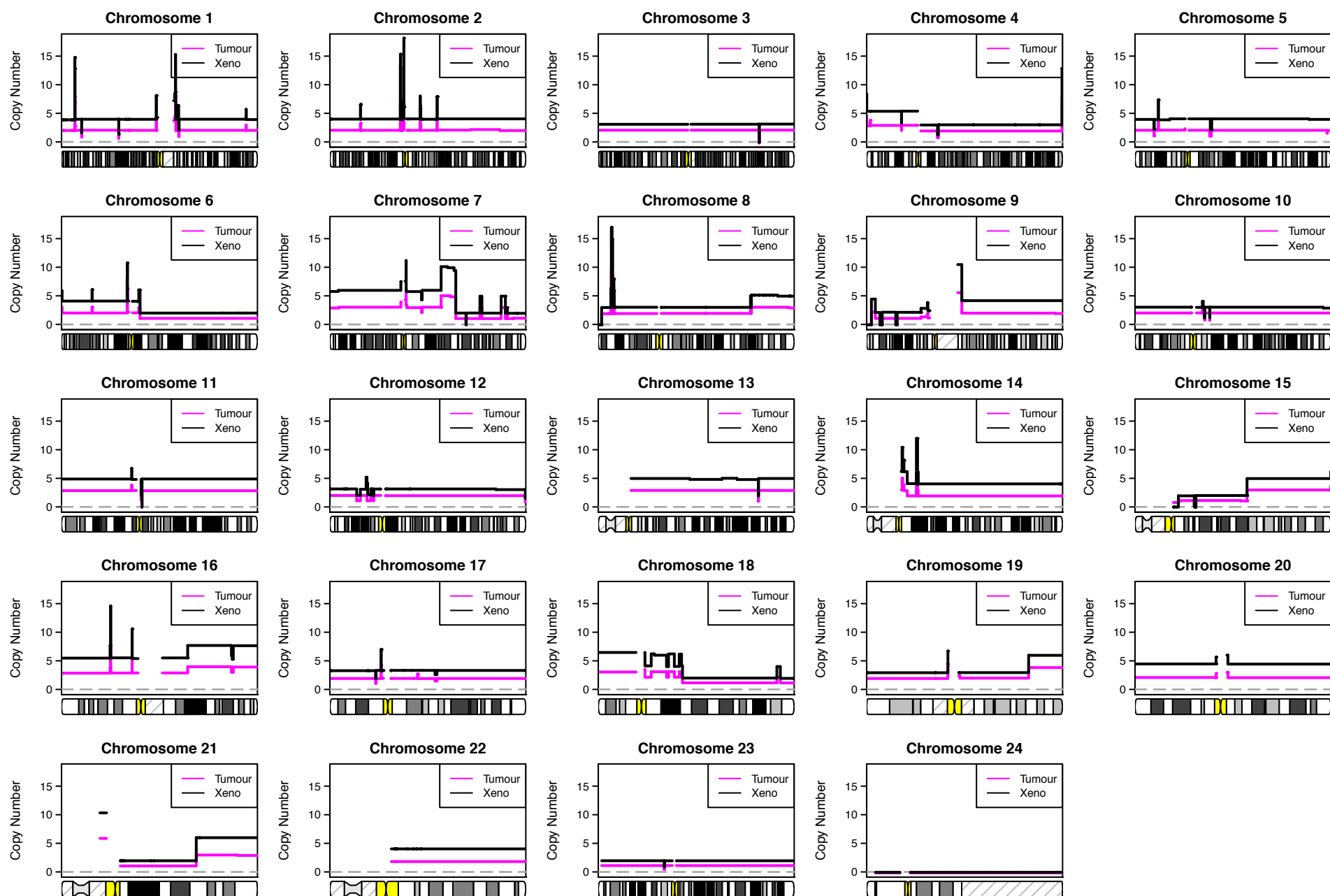

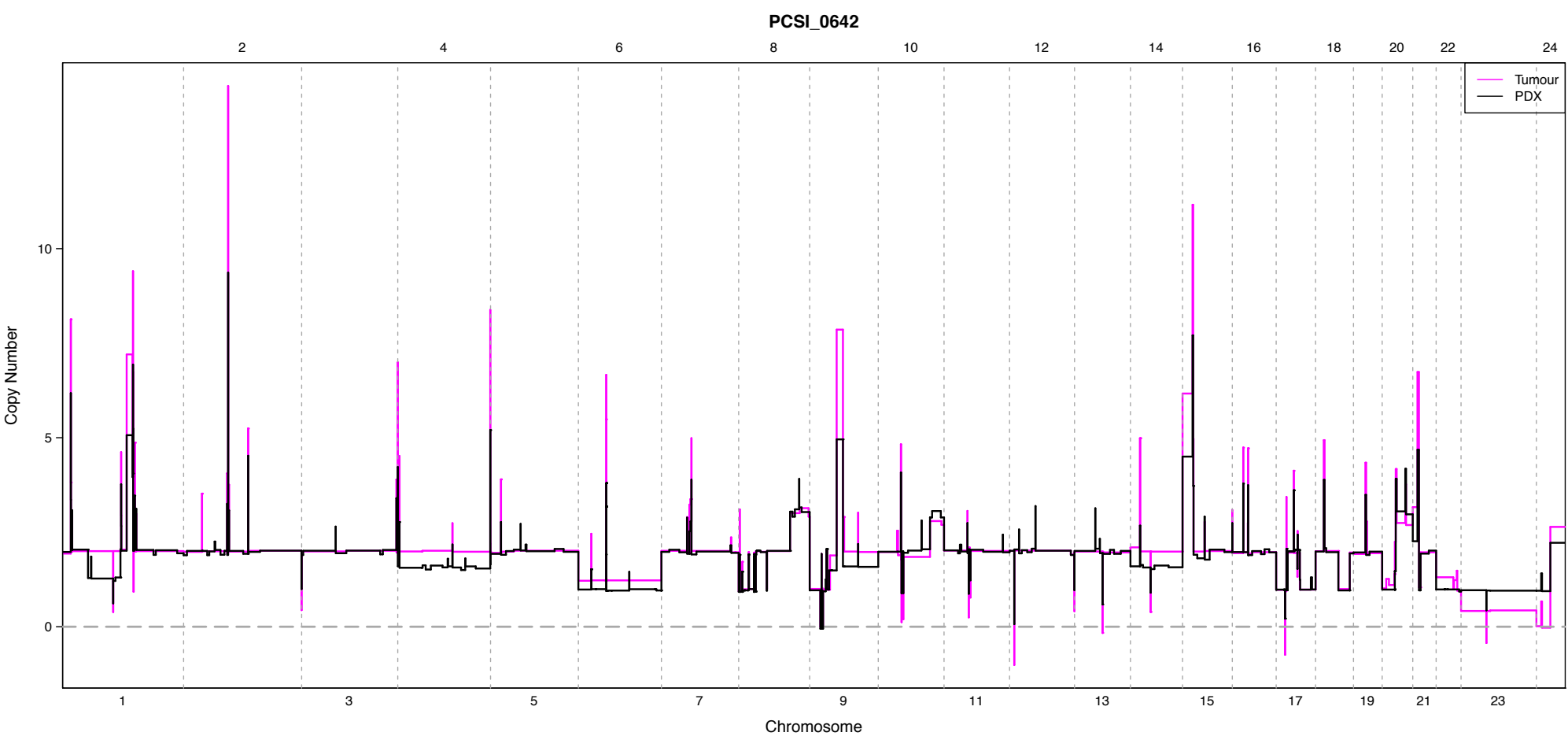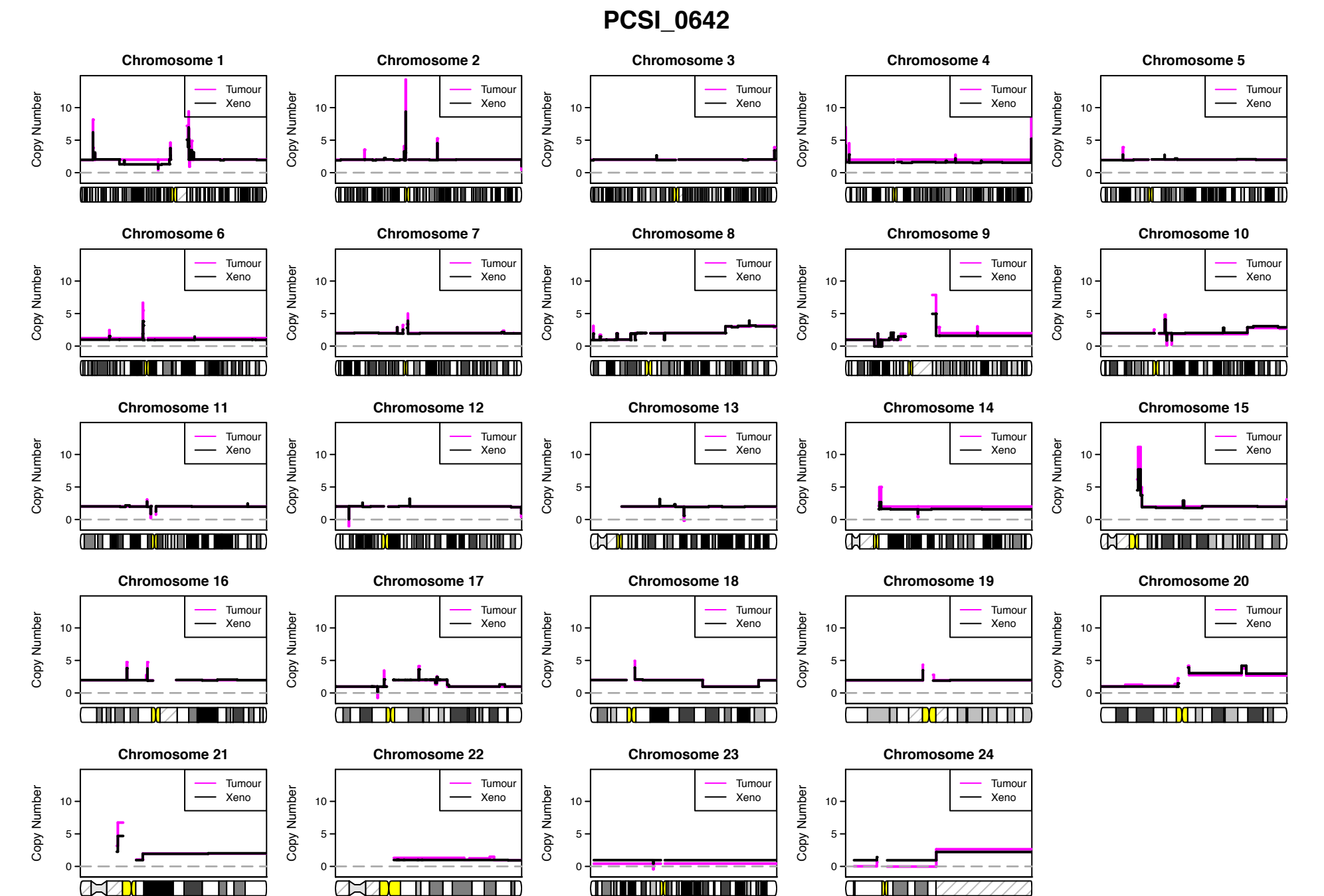

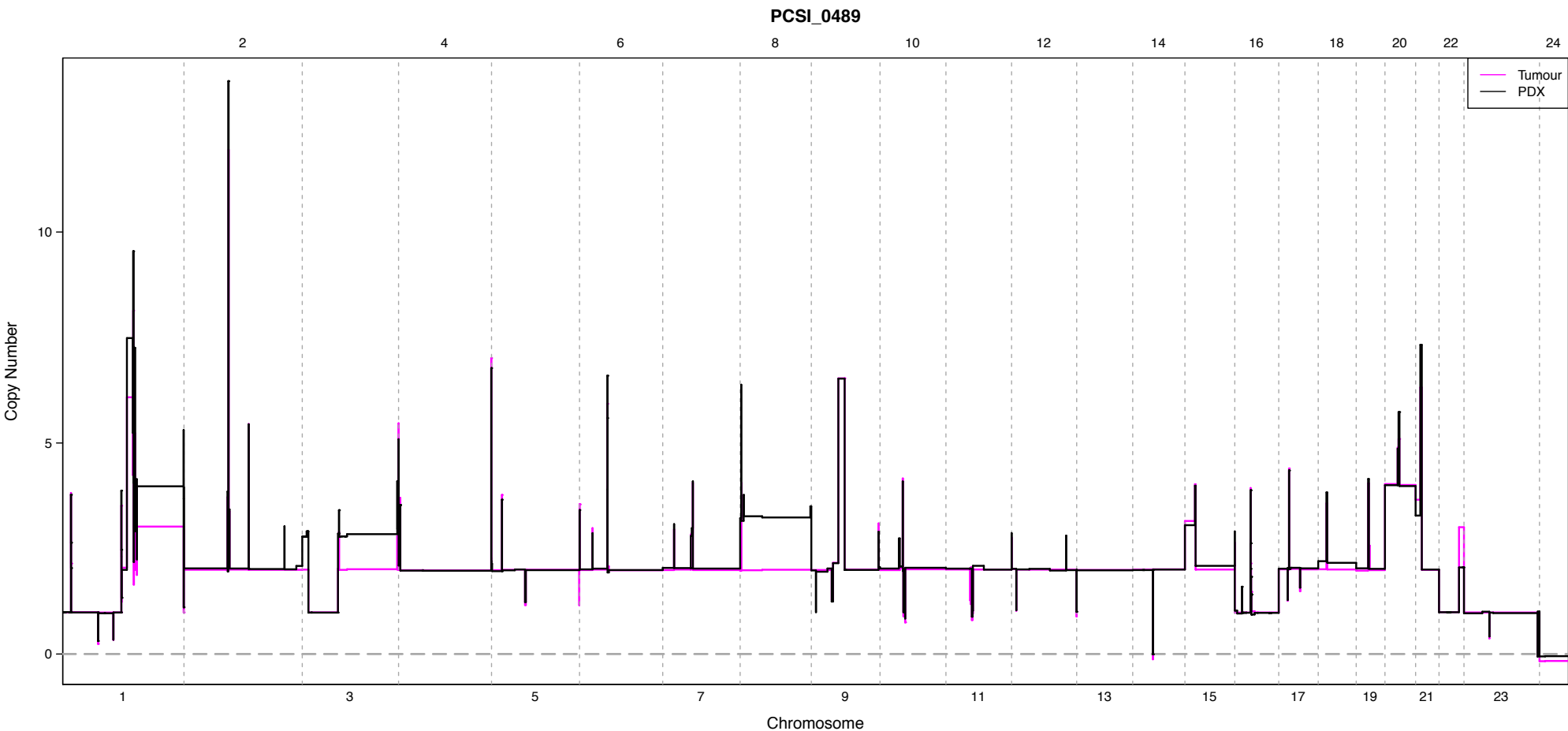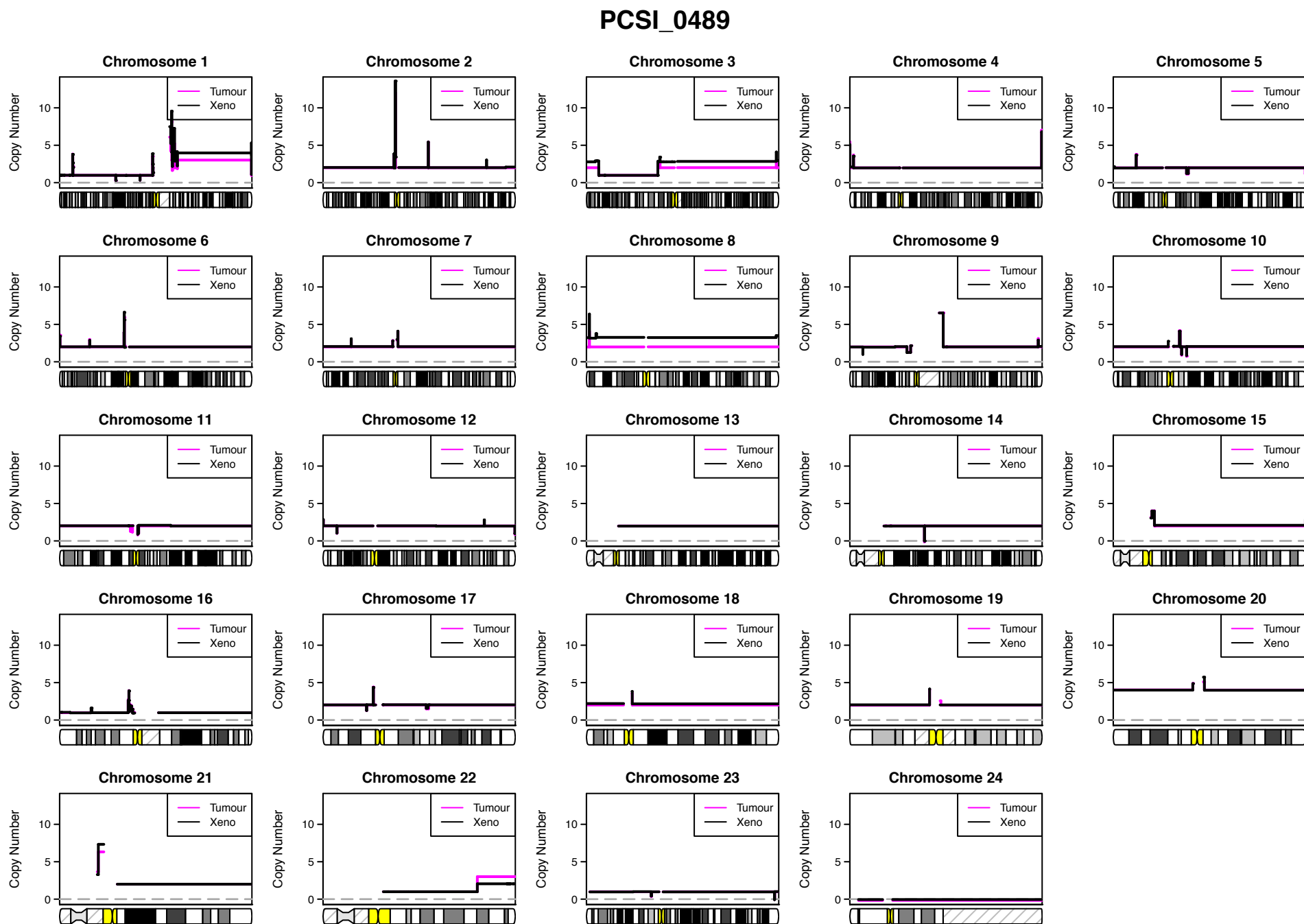

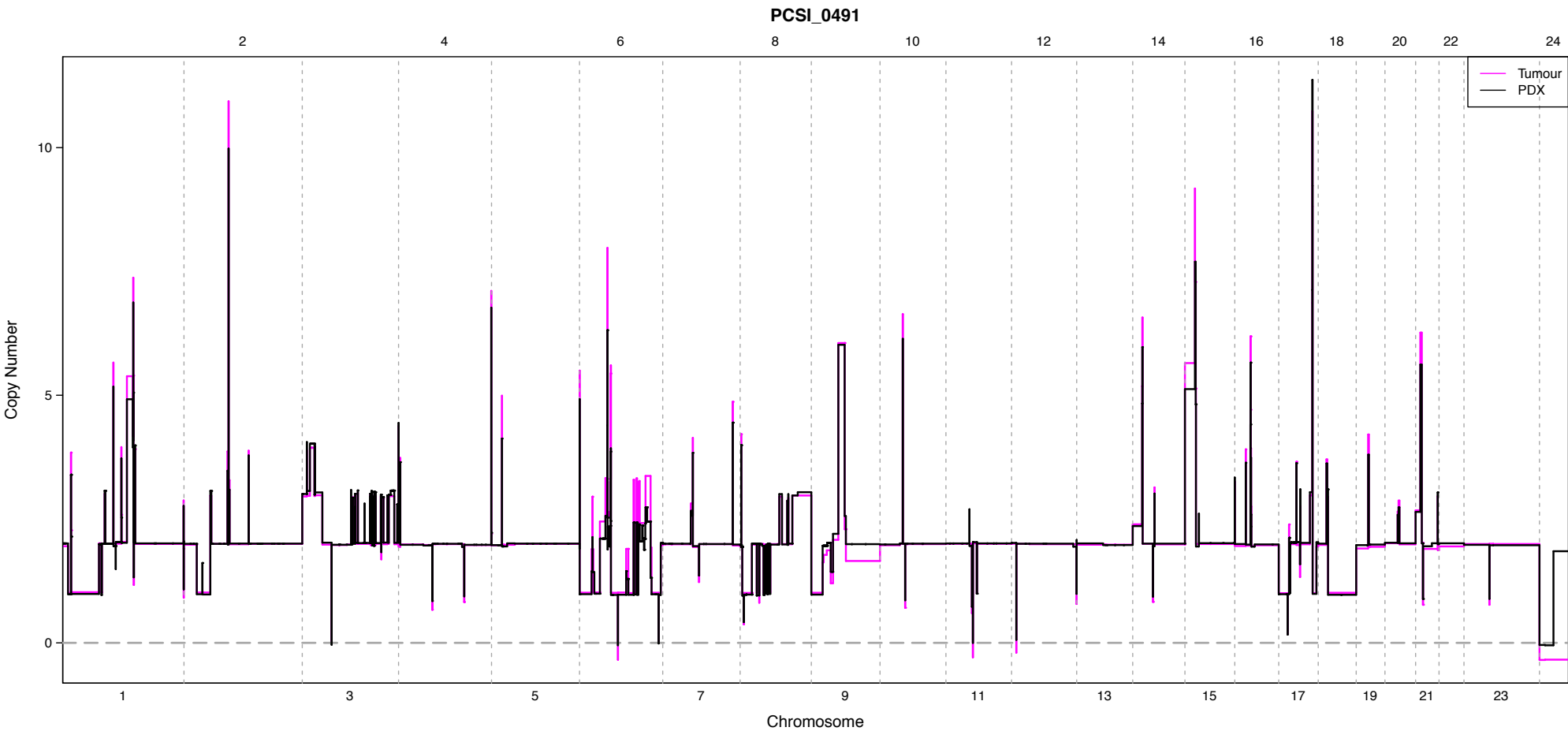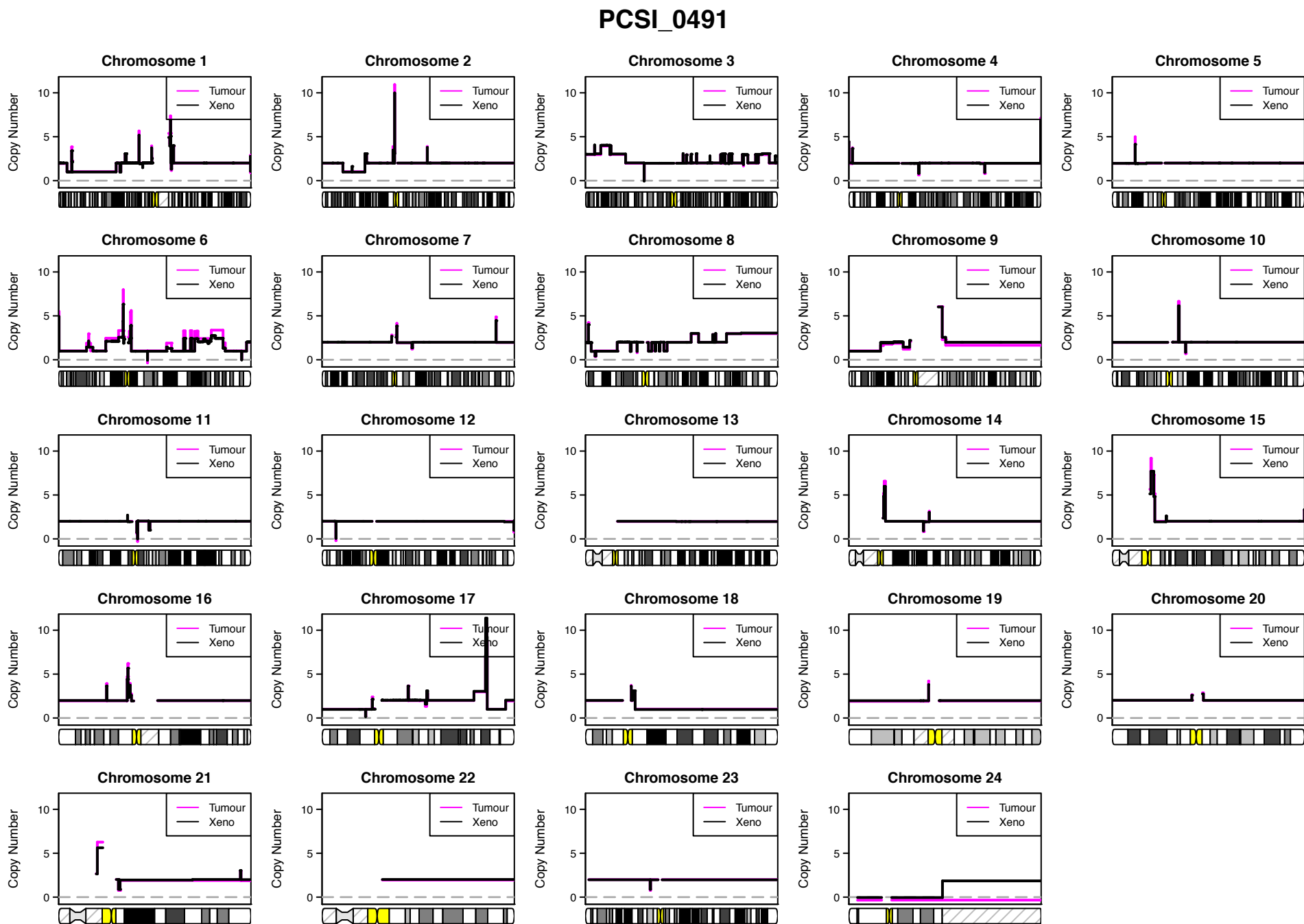

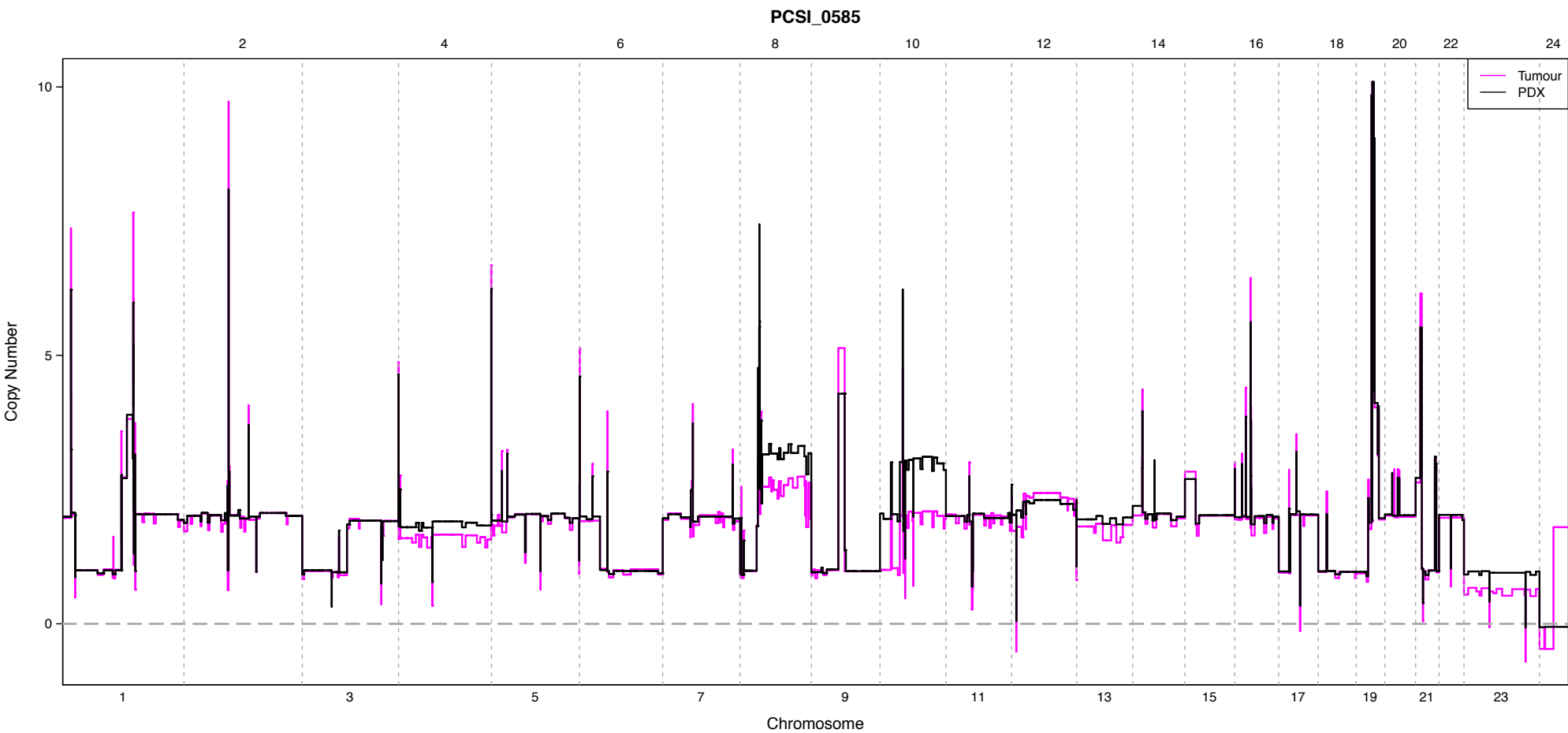

**PCSI\_0585**

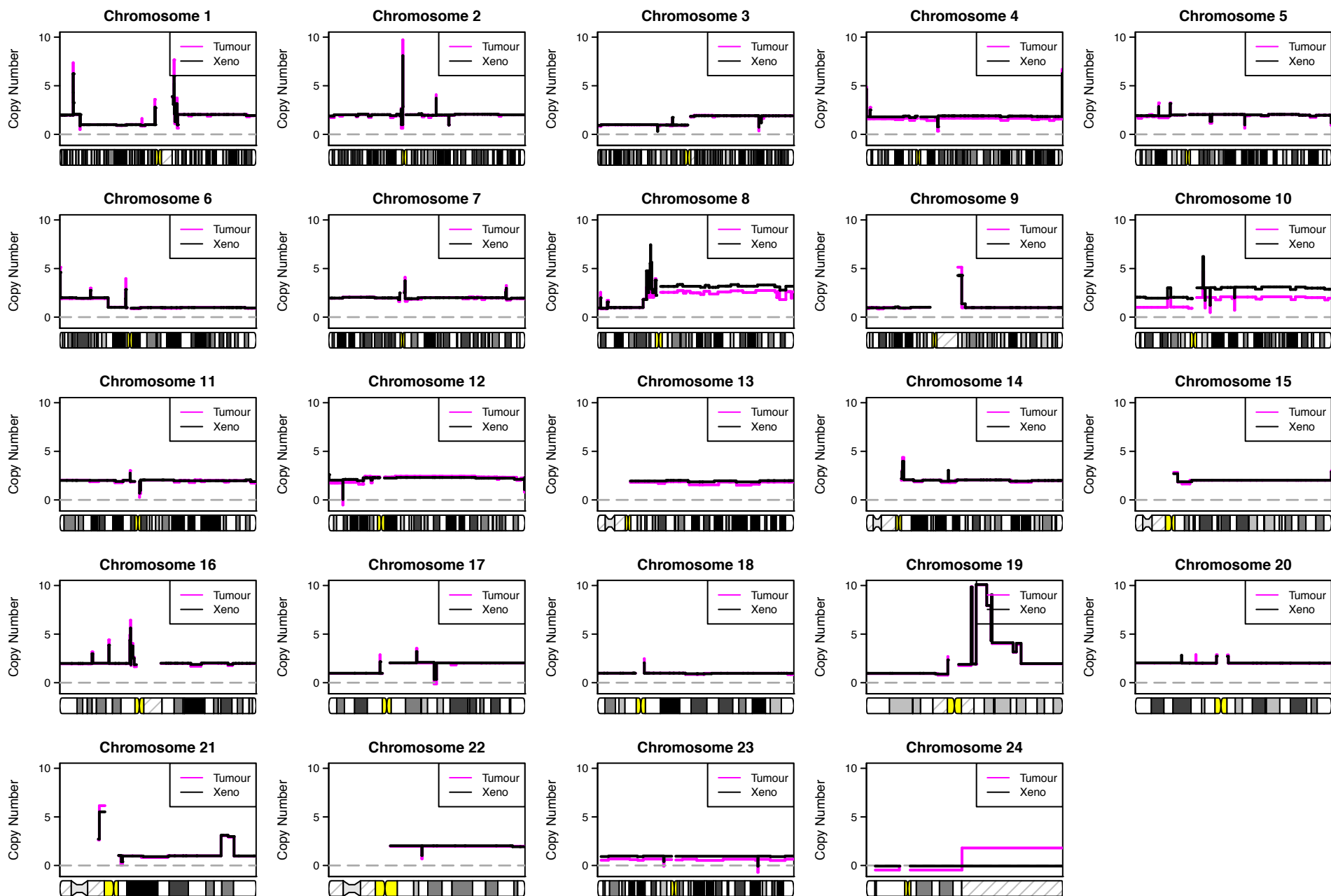

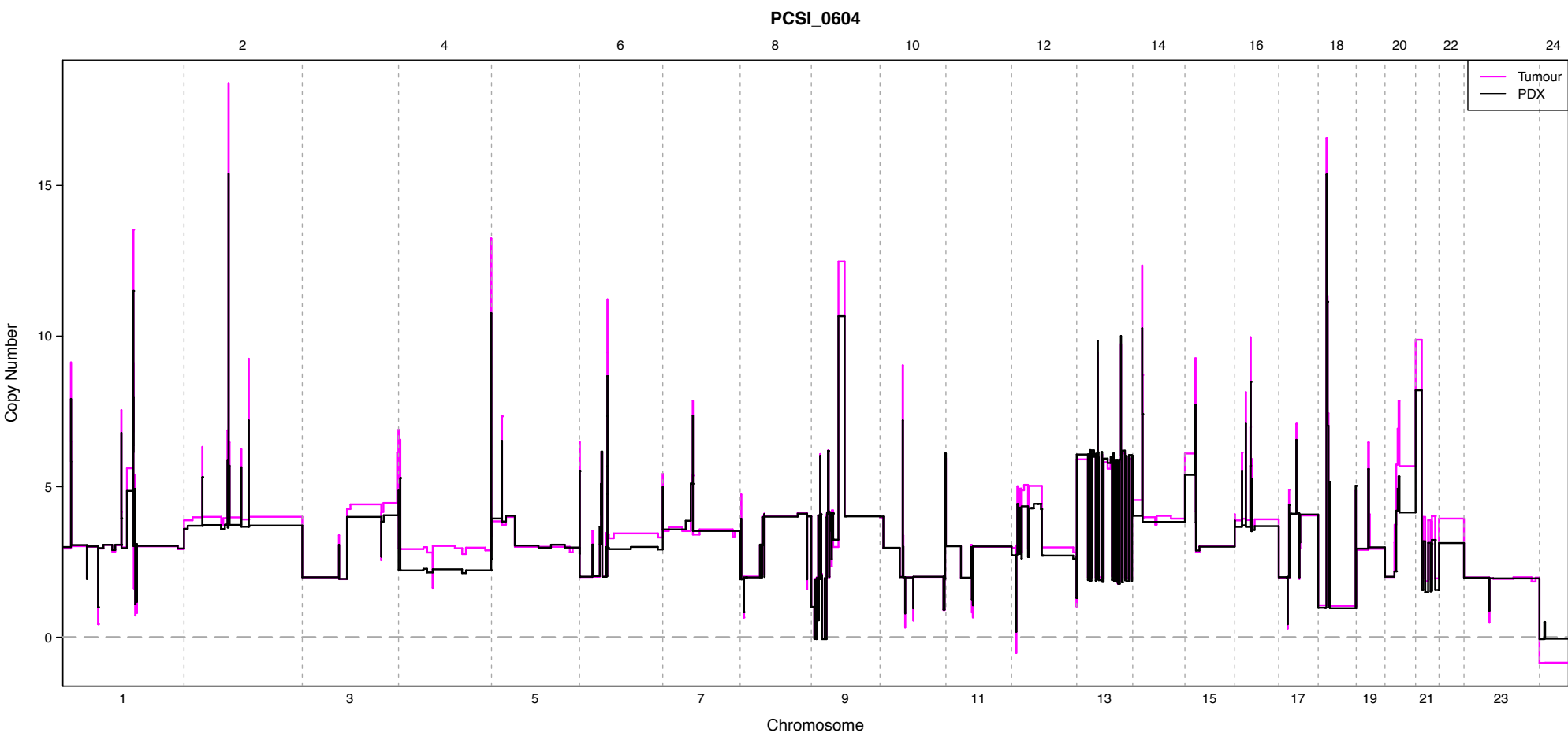

**PCSI\_0604**

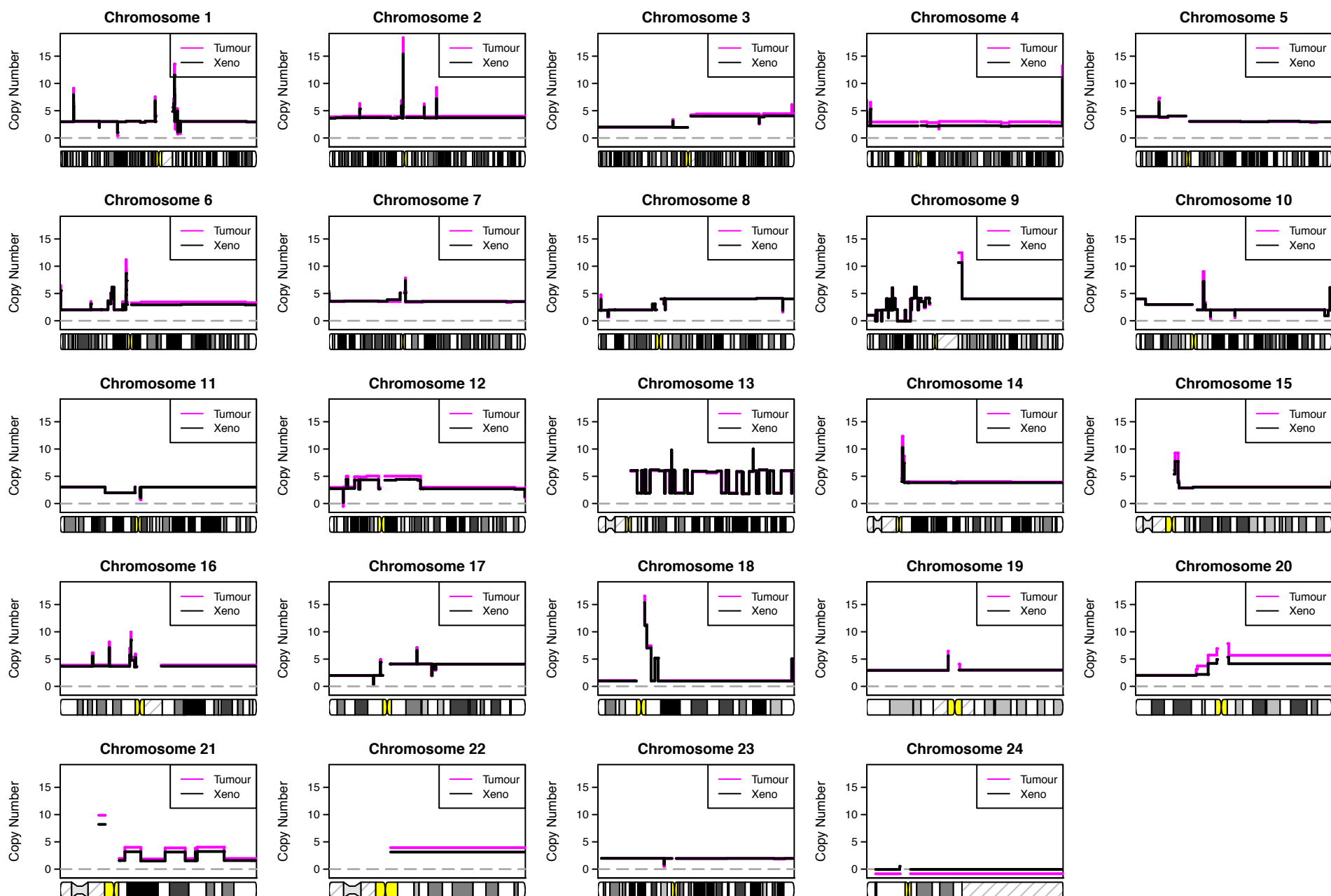

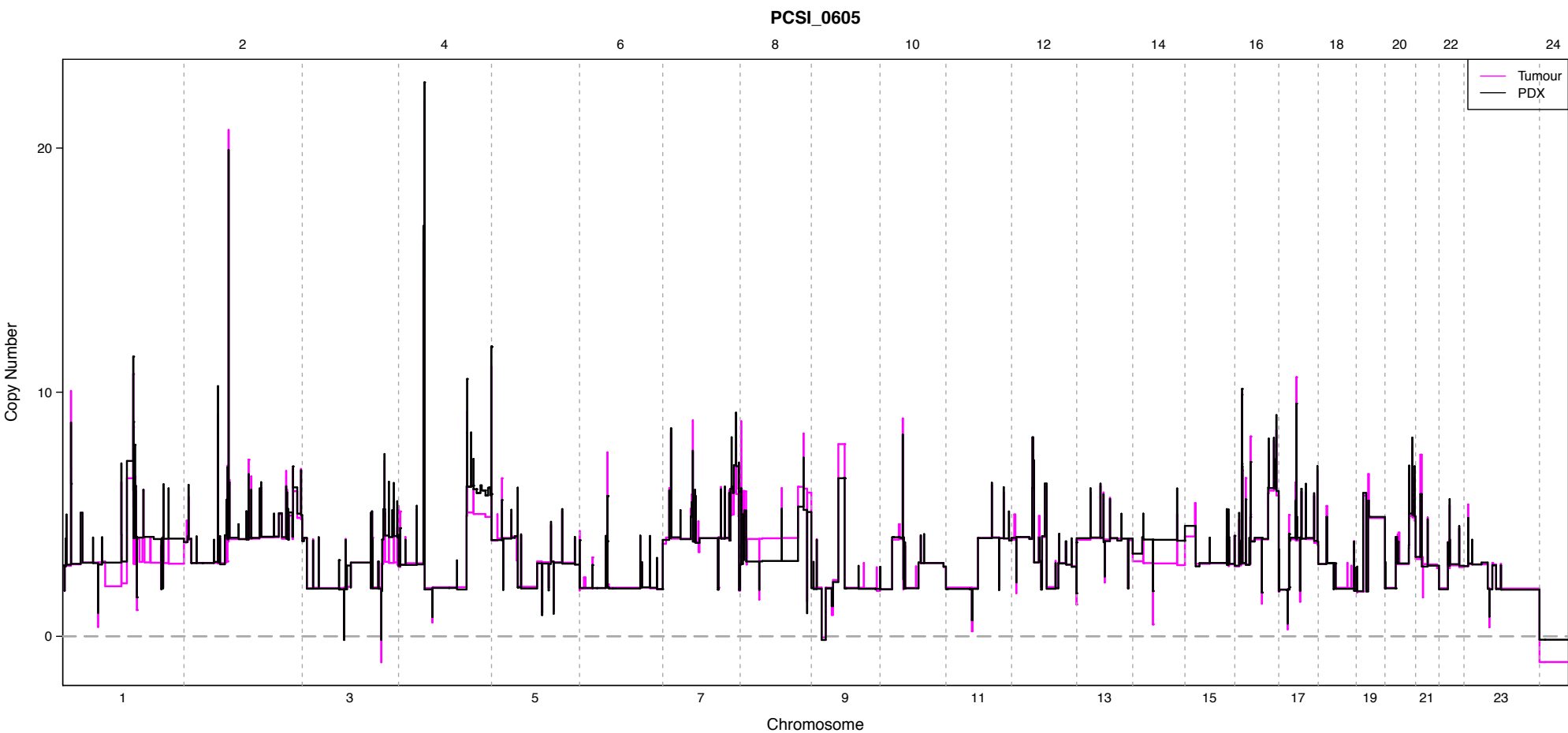

PCSI\_0605

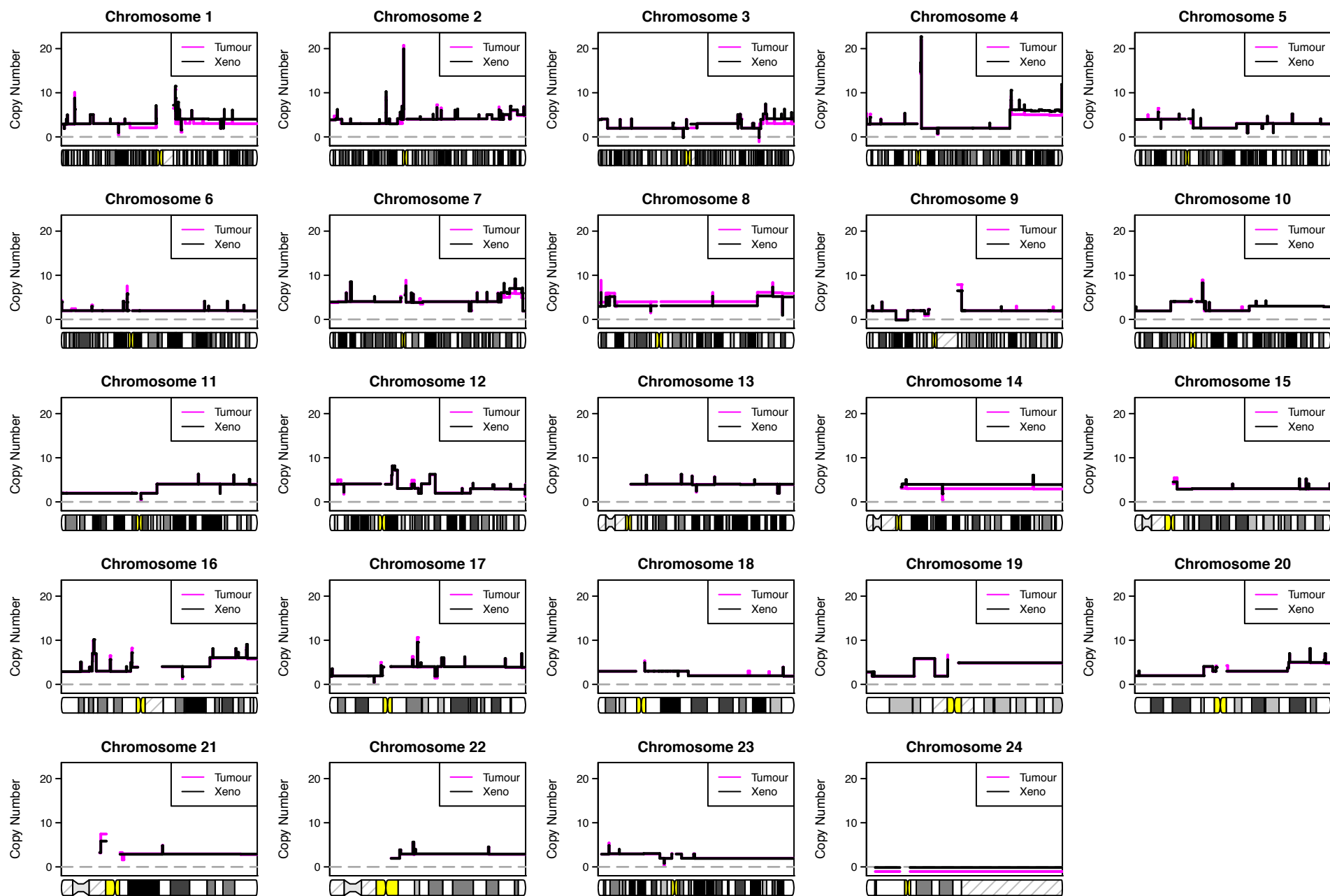

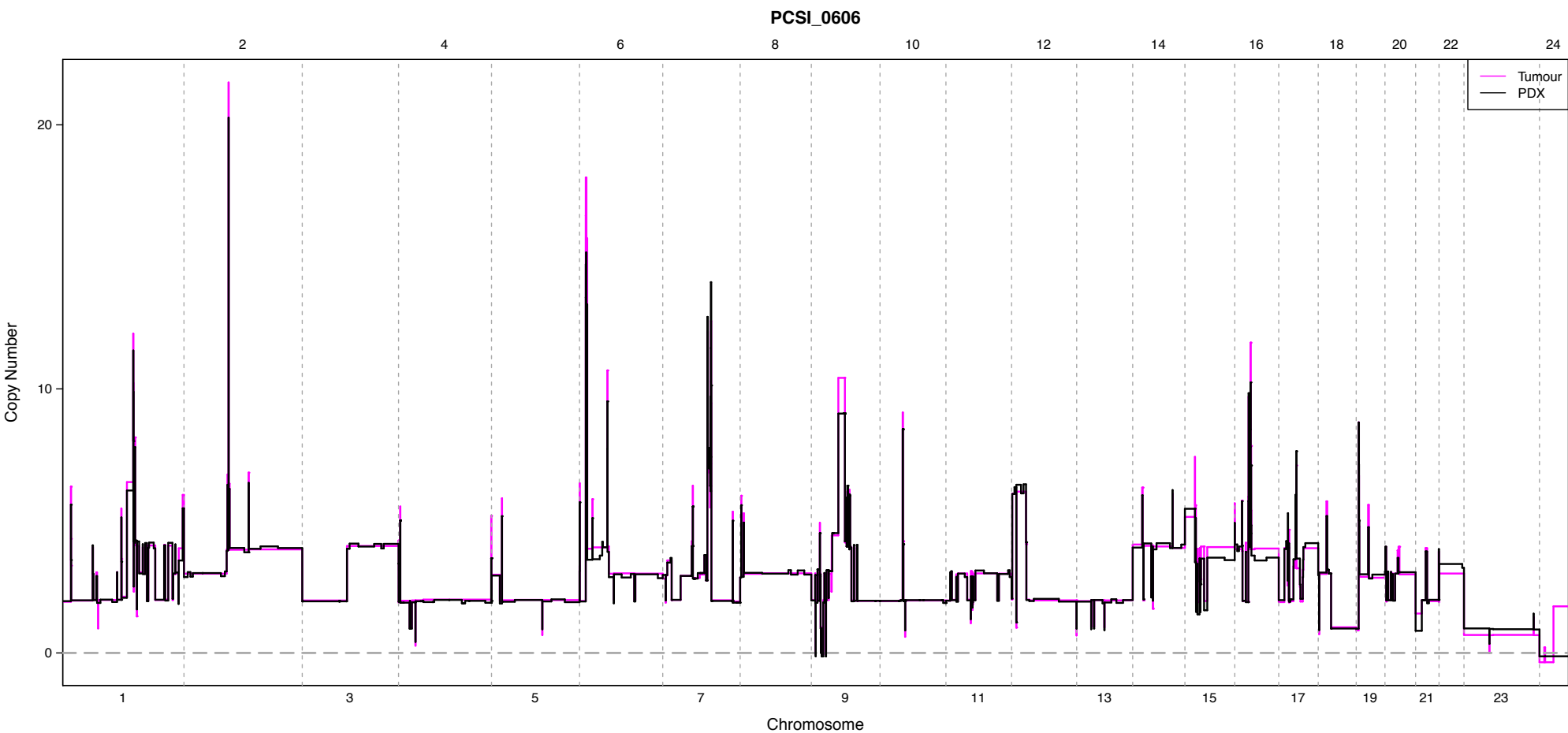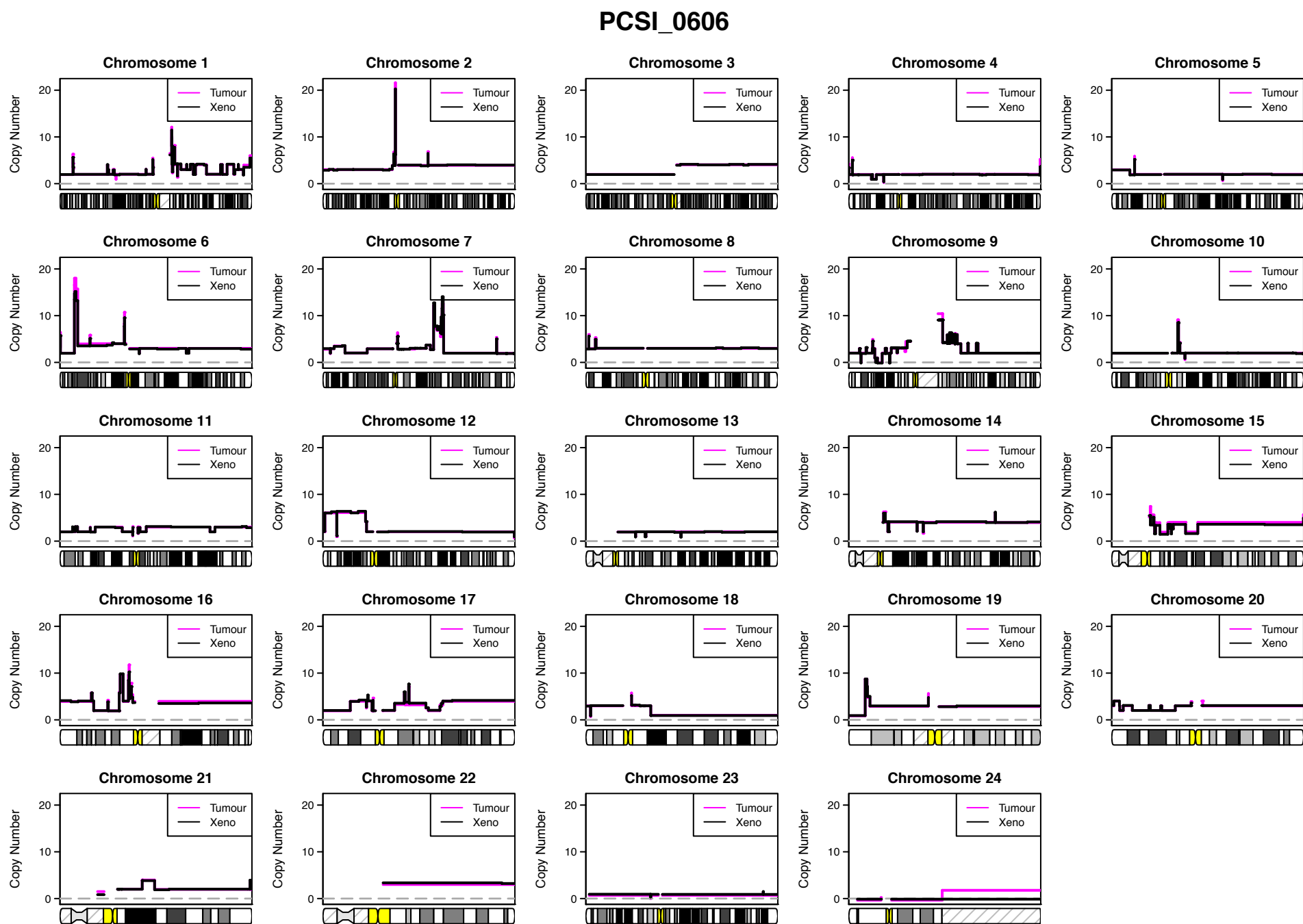

Supplement: S8 Fig — For each pair, the copy number state of the primary tumour (magenta) and matching PDX (black) is plotted across all chromosomes. A detailed panel also shows the copy number of the tumour and PDX across each chromosome. Values presented are raw (uncorrected) copy number values. (PDF) [file pcbi.1006596.s008.pdf]

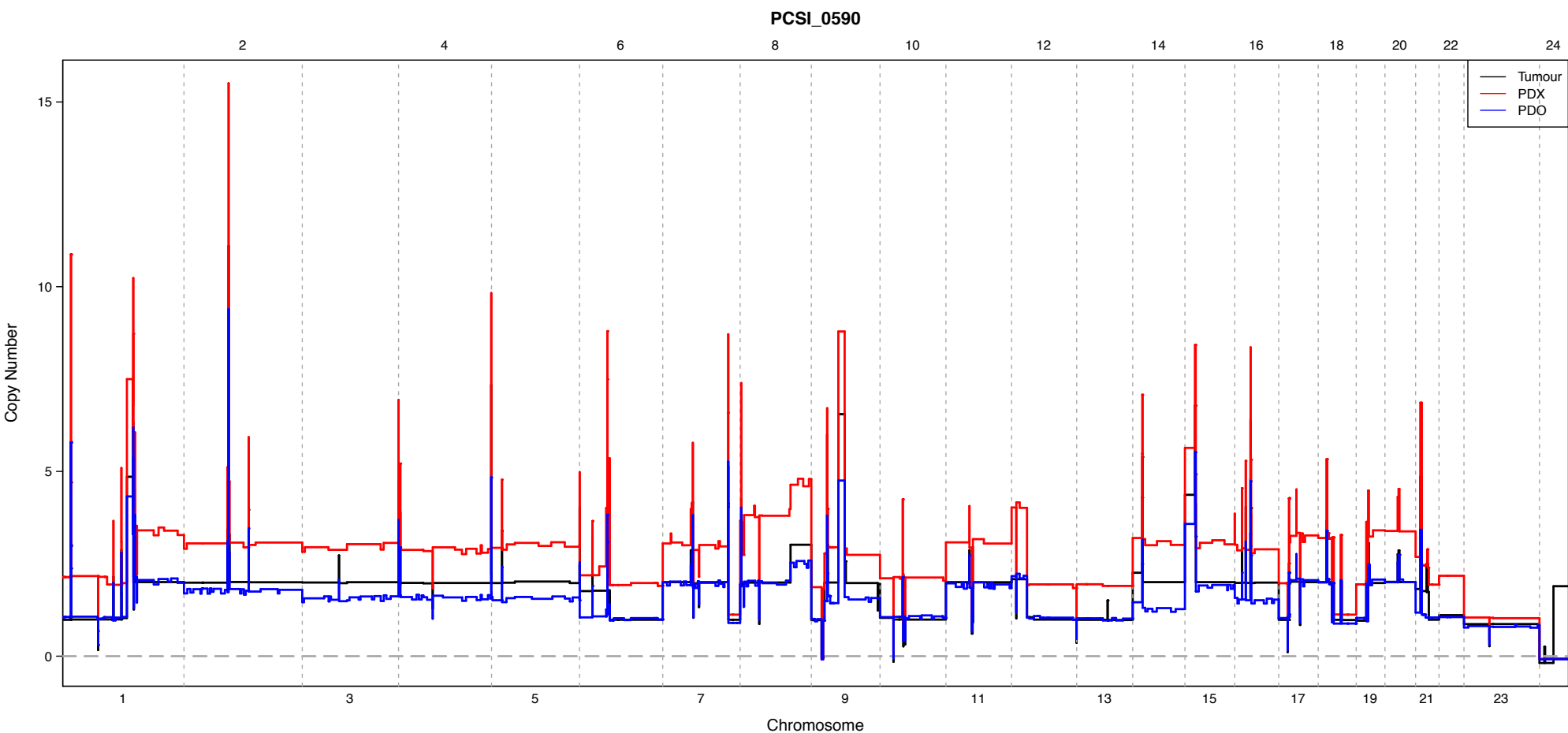

**PCSI\_0590**

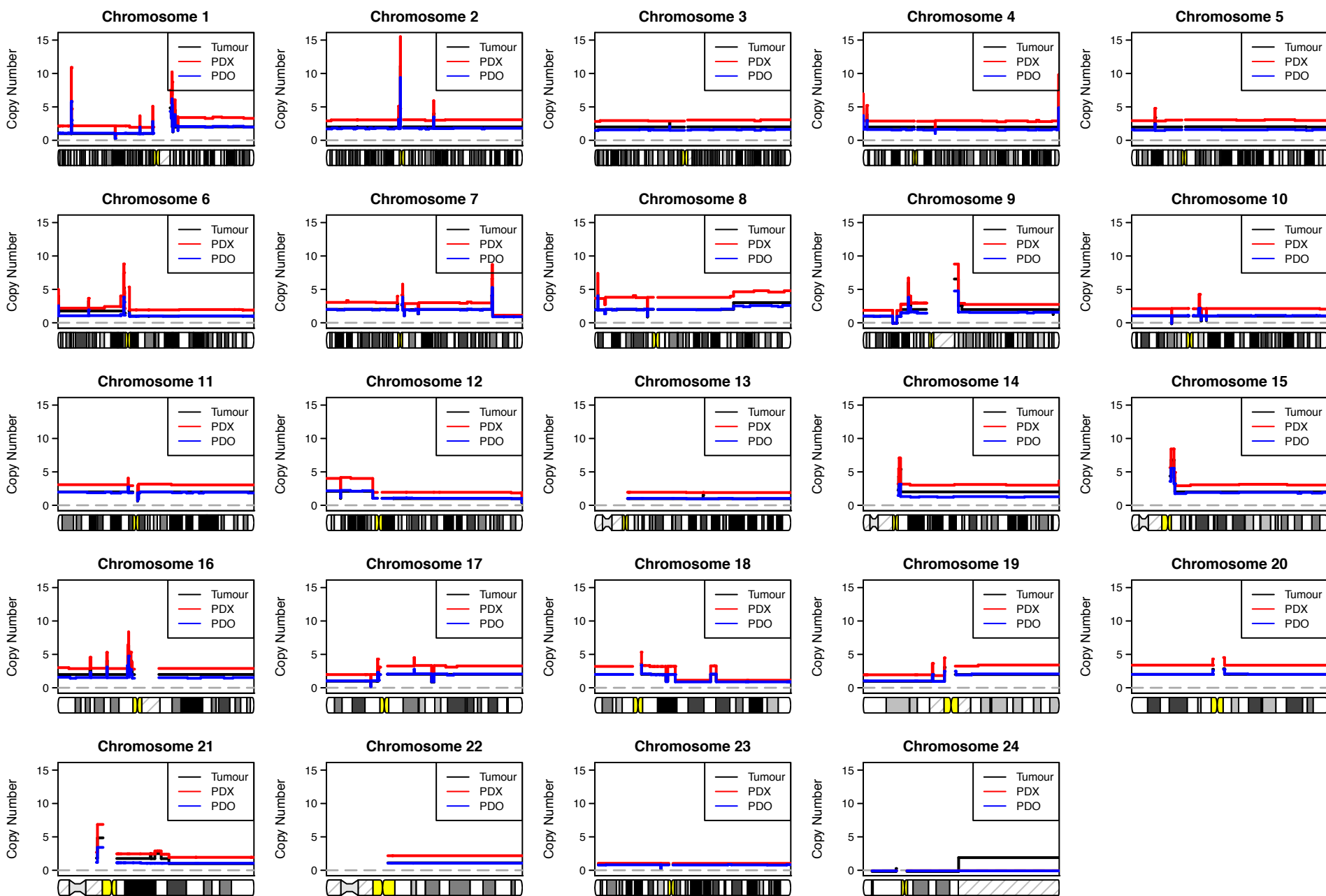

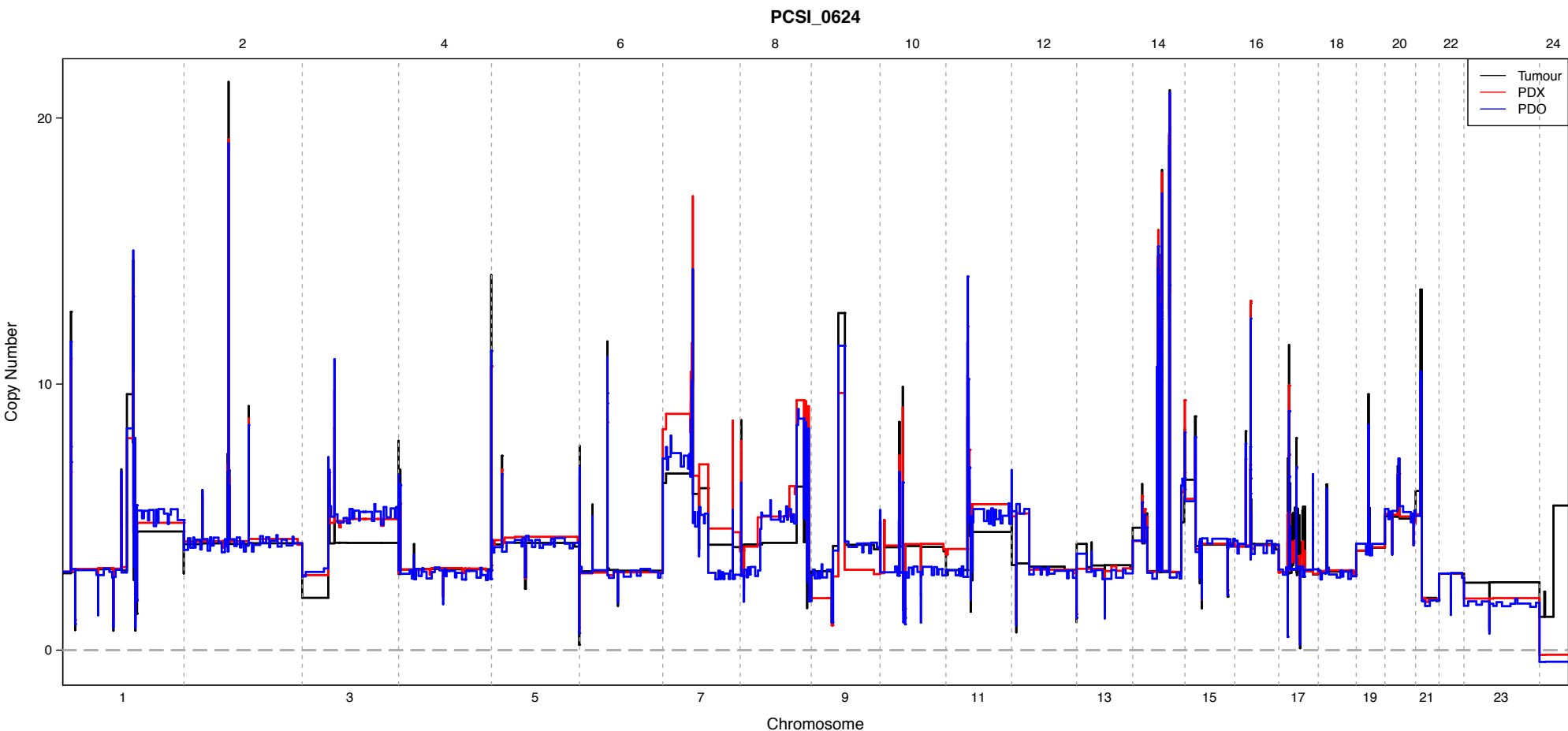

**PCSI\_0624**

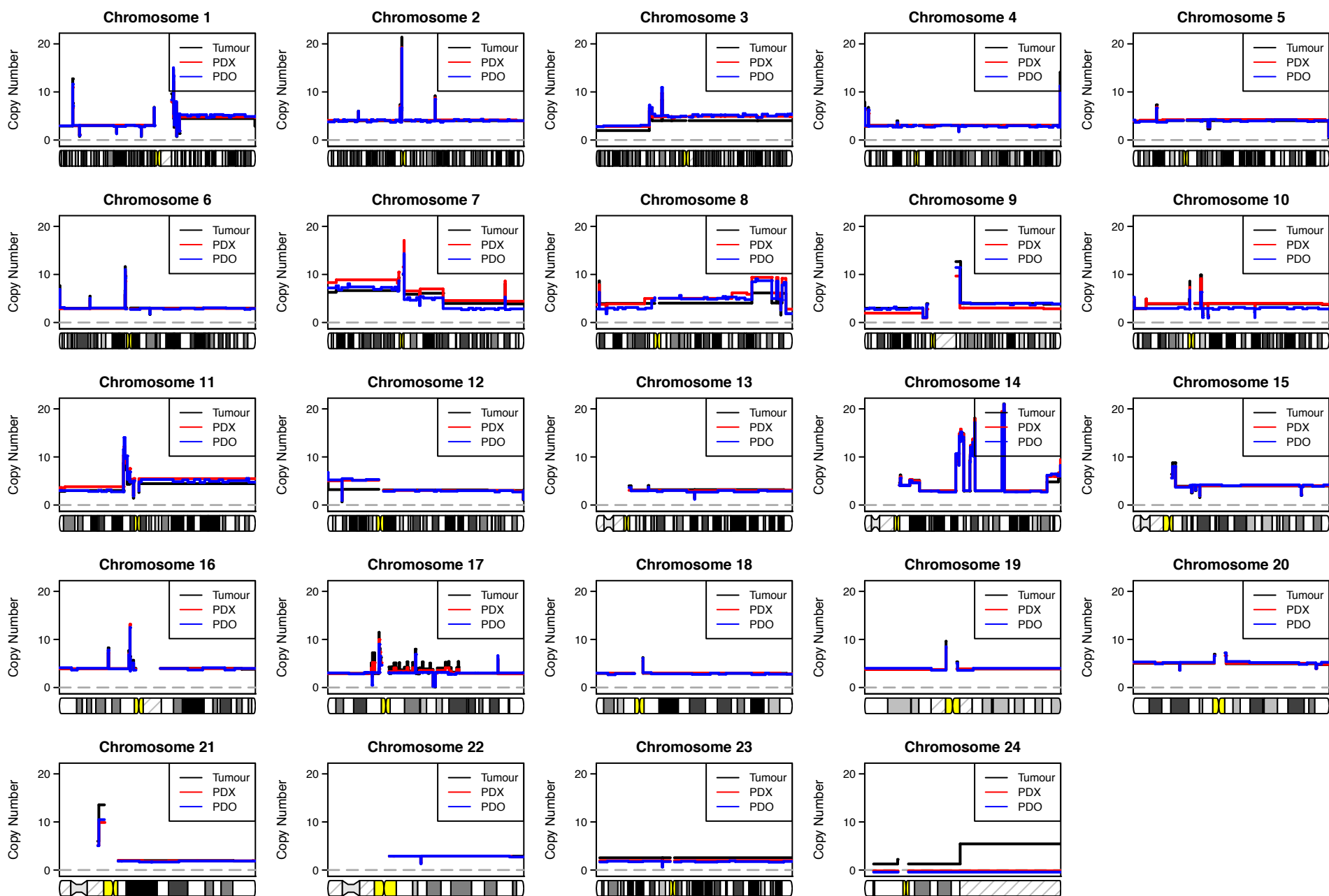

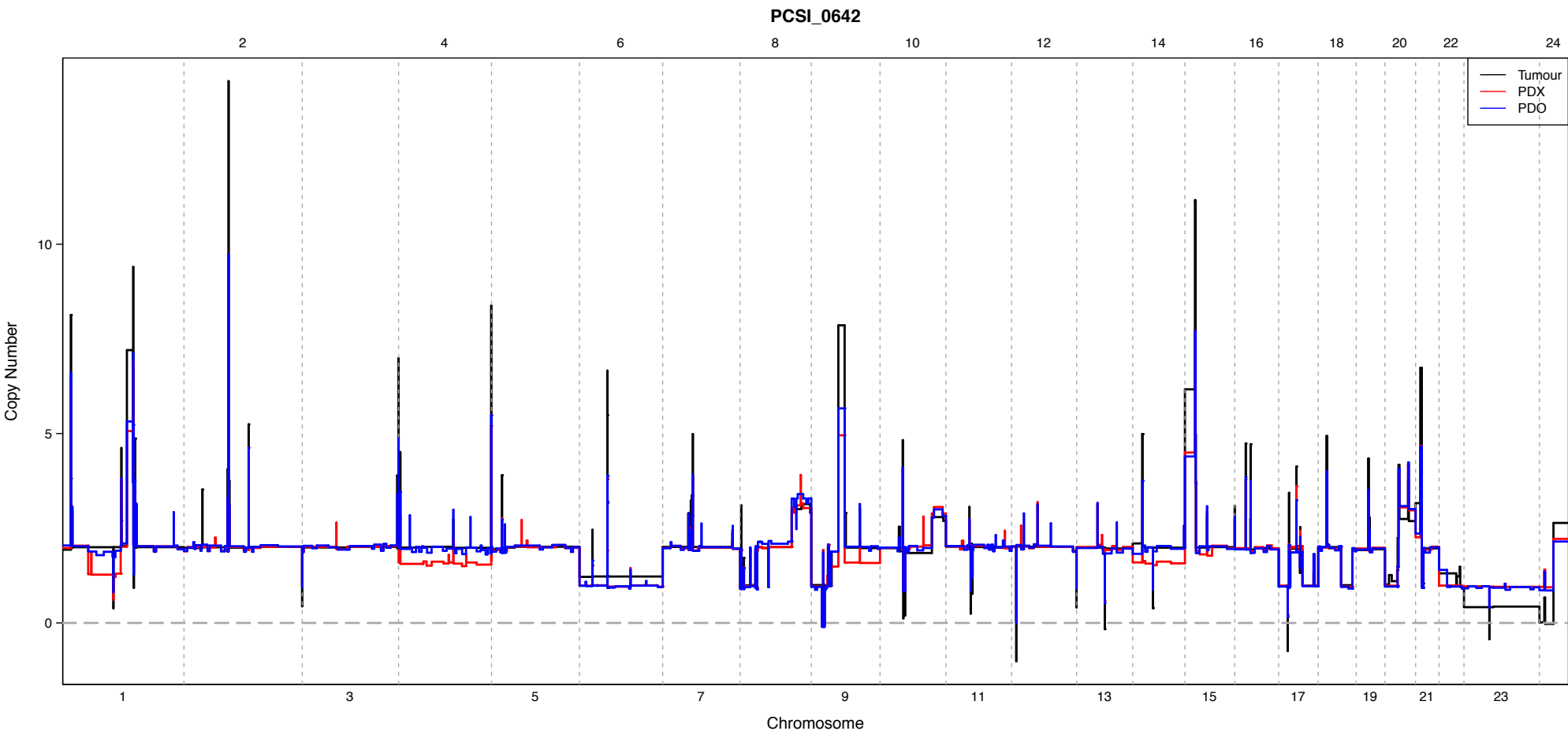

**PCSI\_0642**

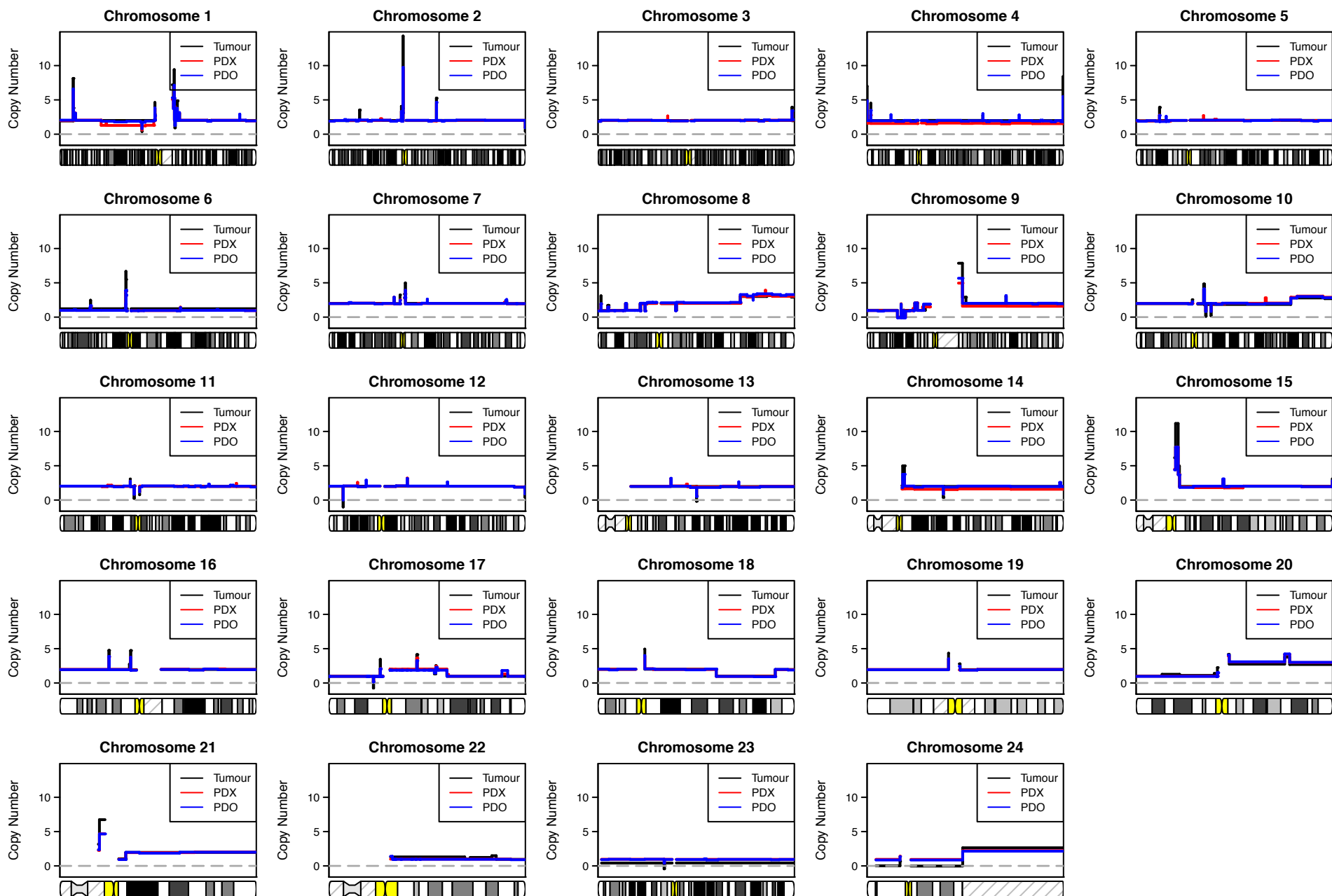

Supplement: S10 Fig — For each trio, the copy number of the tumour (black), matched PDX (red), and matching PDO (blue) is plotted across the genome. A detailed panel also shows the copy number of the tumour and PDX across each chromosome. Values presented are raw (uncorrected) copy number values. (PDF) [file pcbi.1006596.s010.pdf]

A. RESECTED PDAC PATIENTS

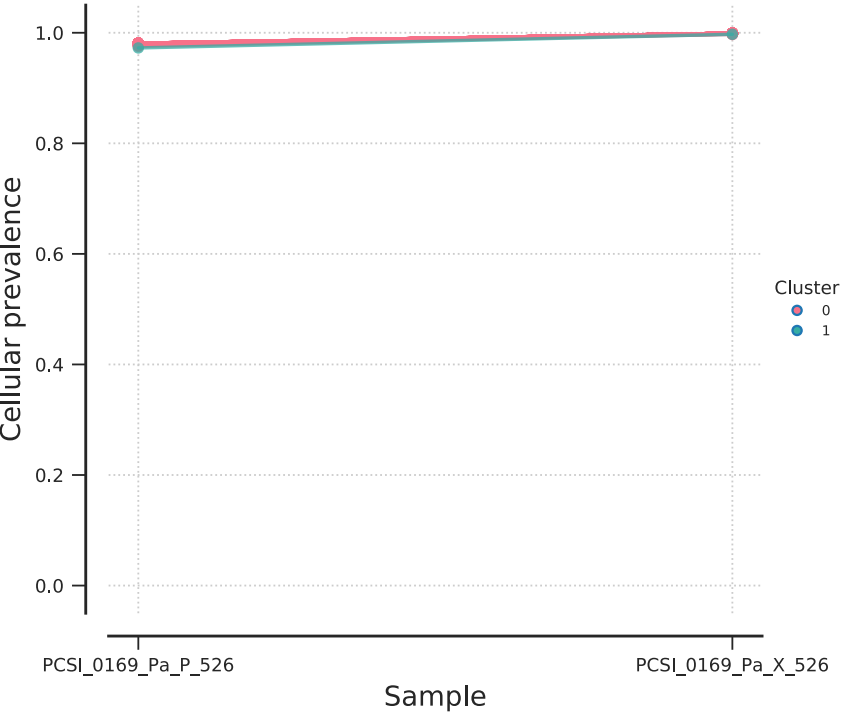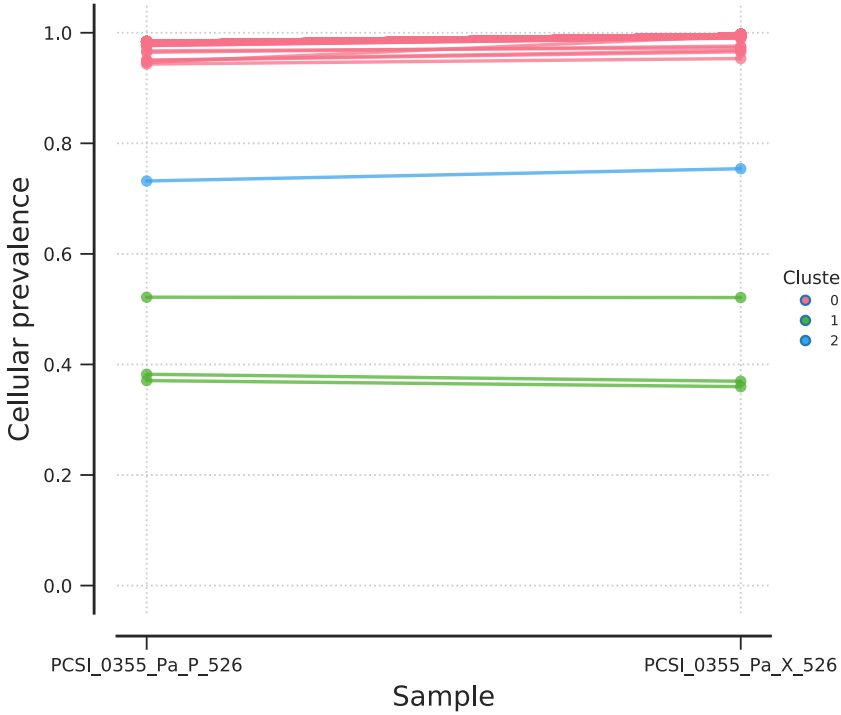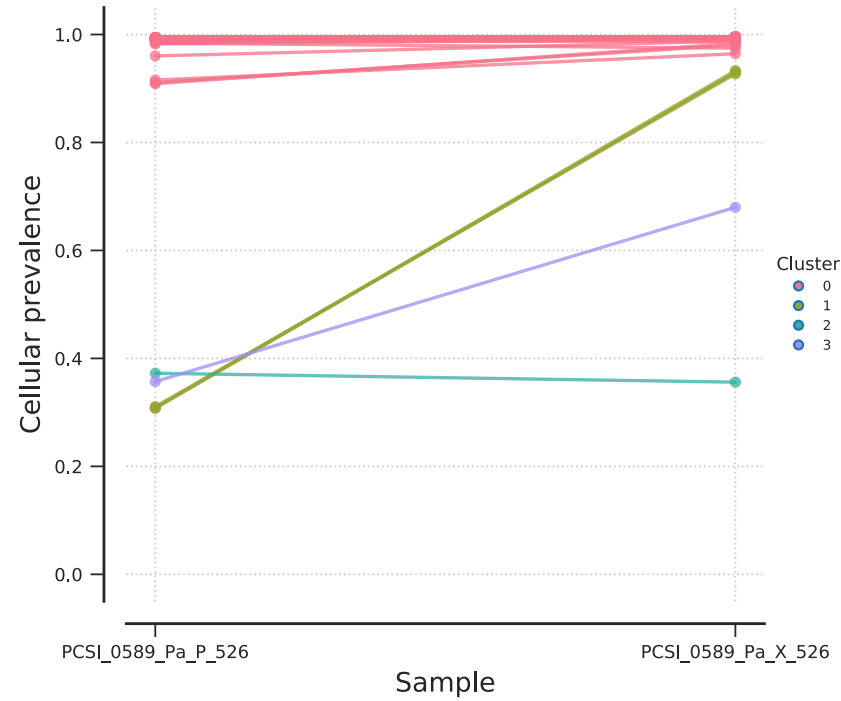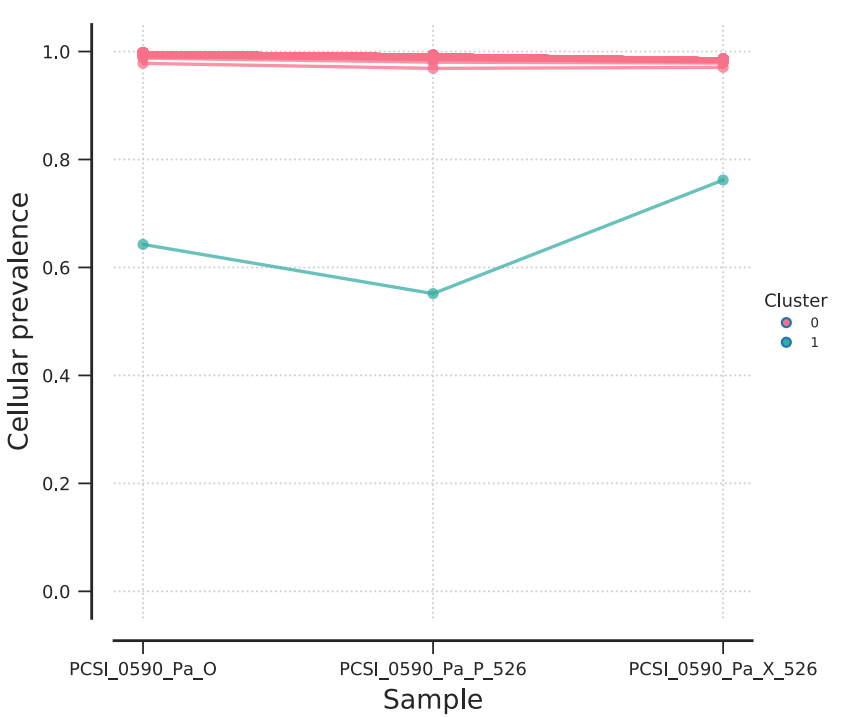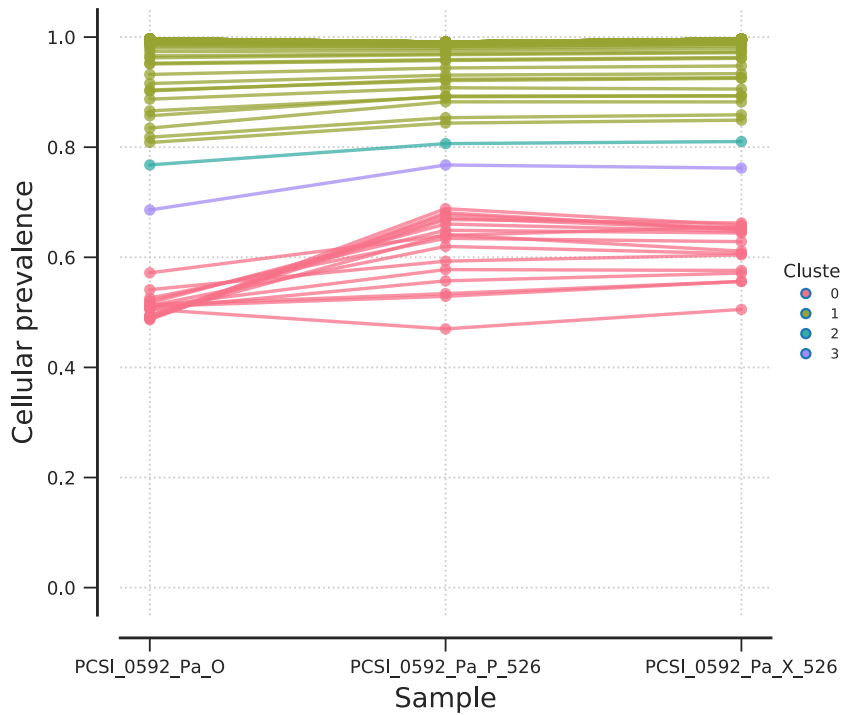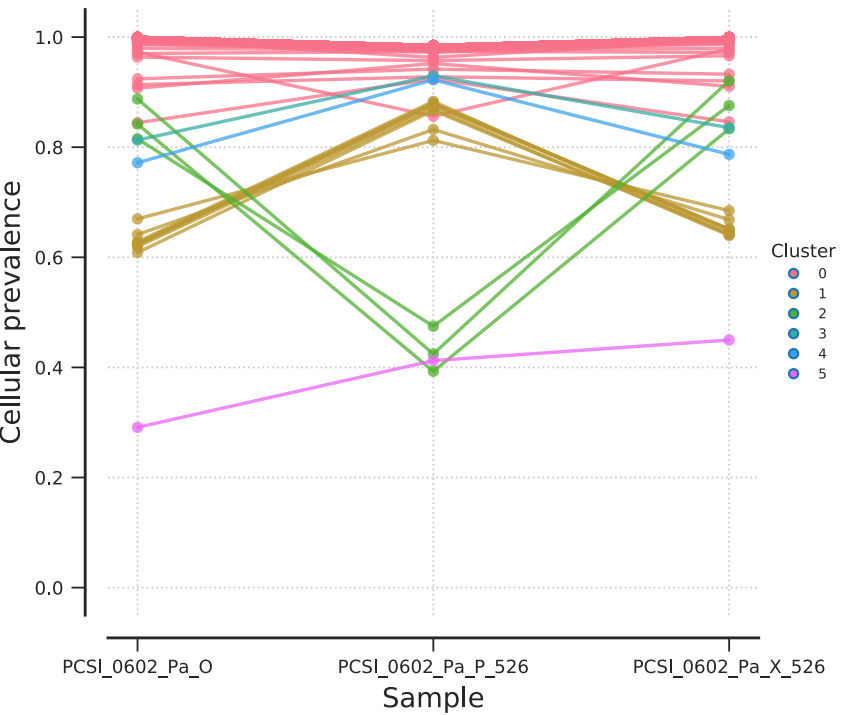

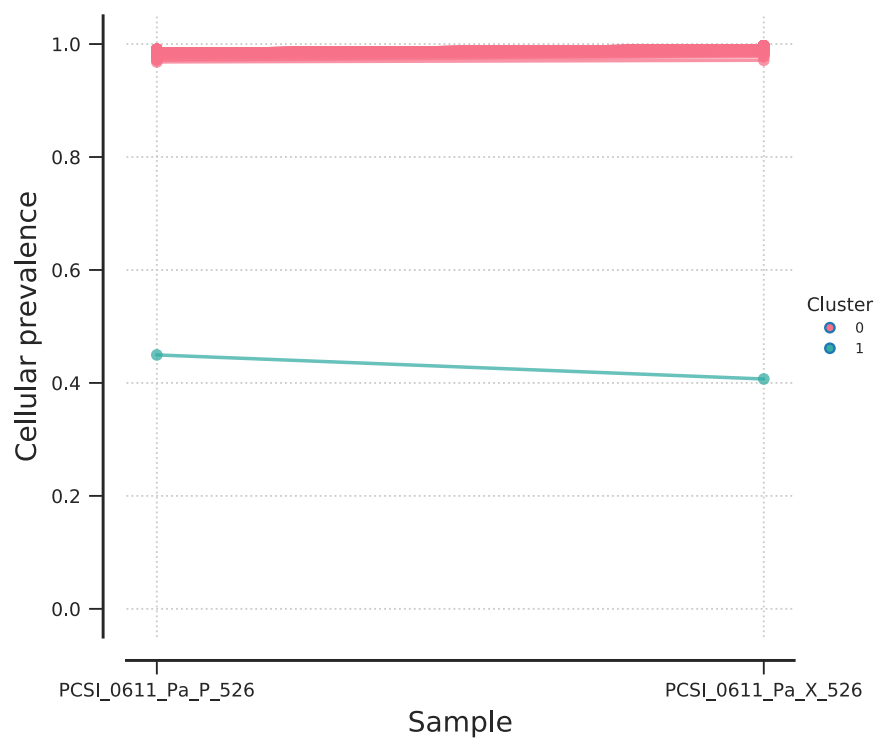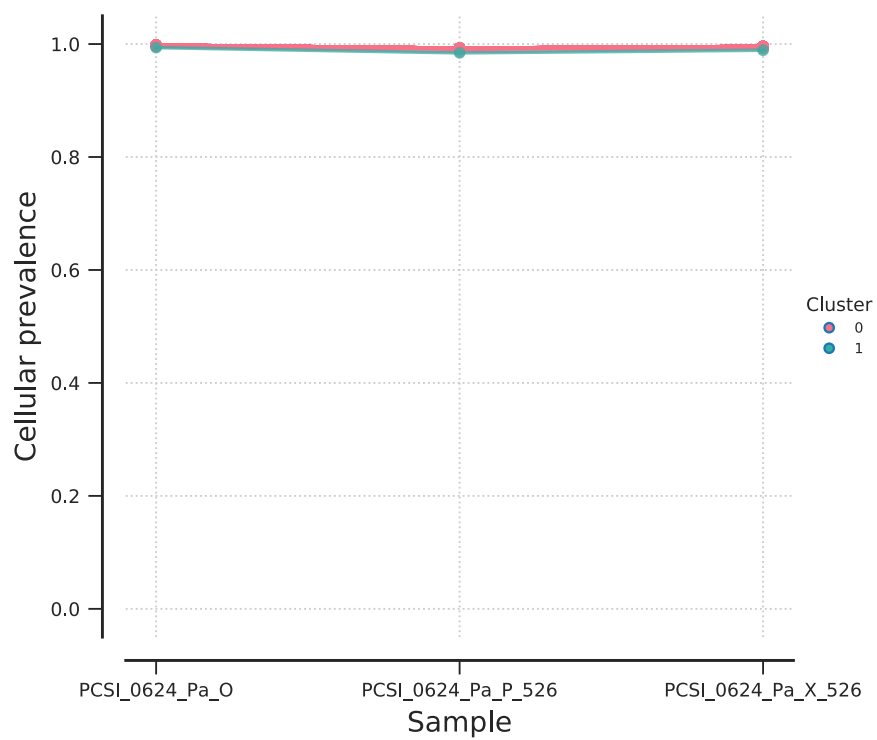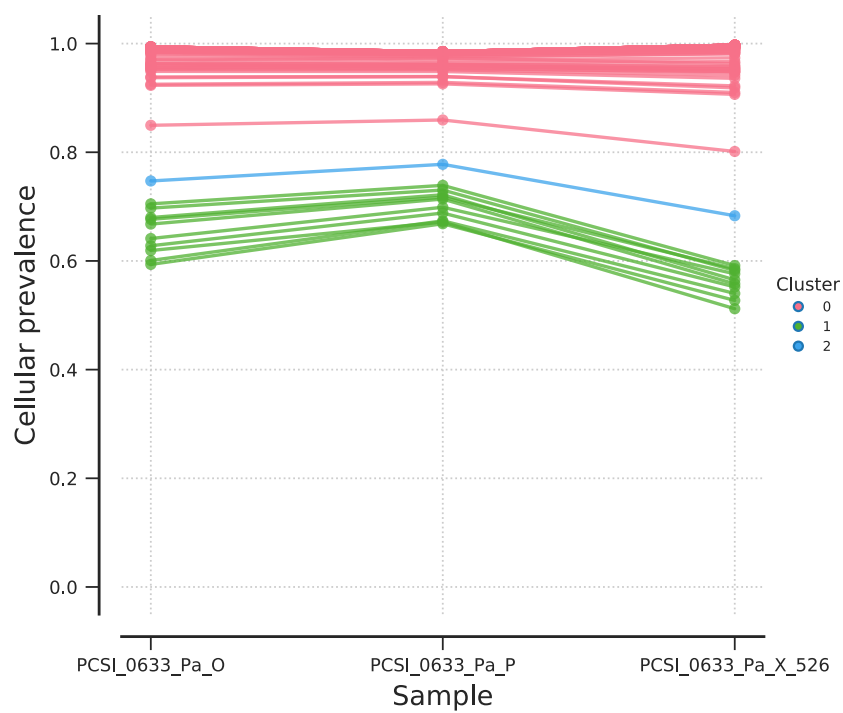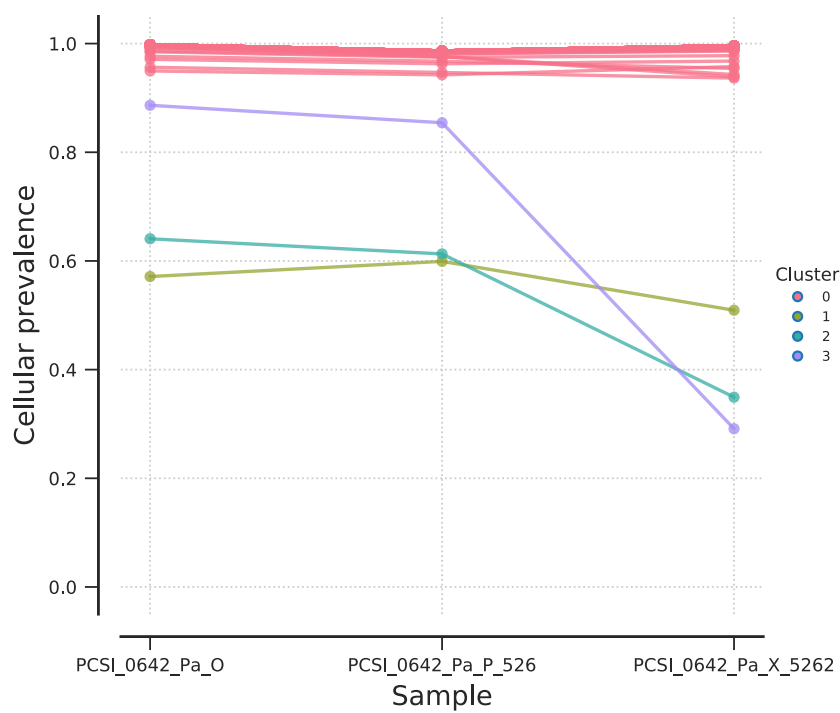

B. LIVER METASTASIS PATIENTS

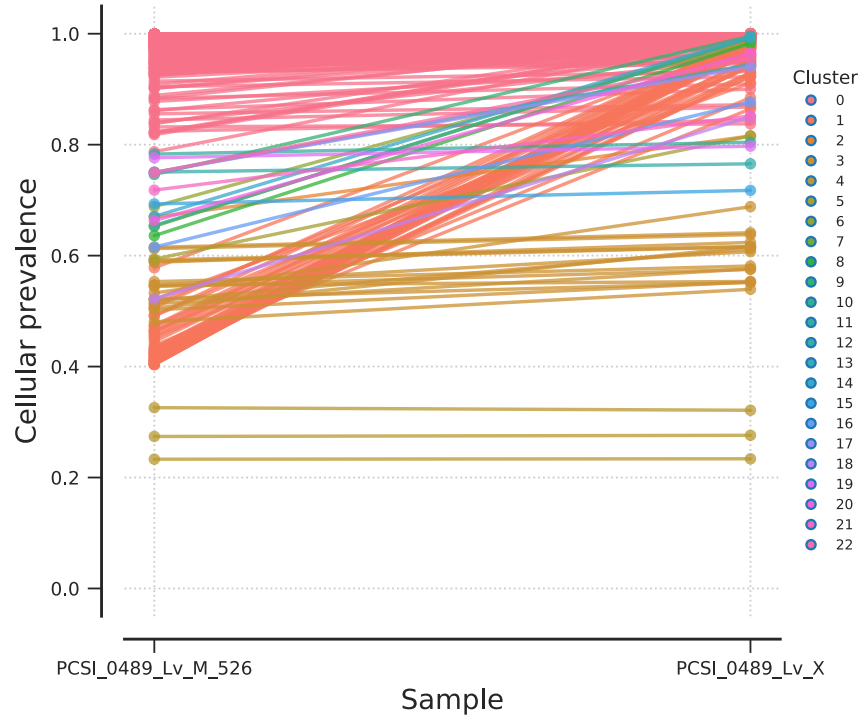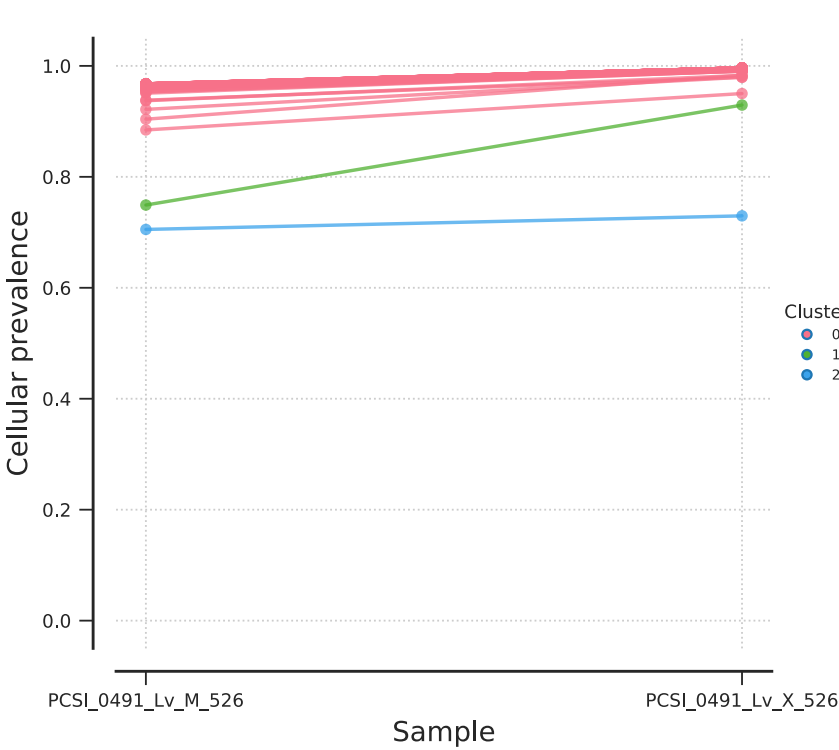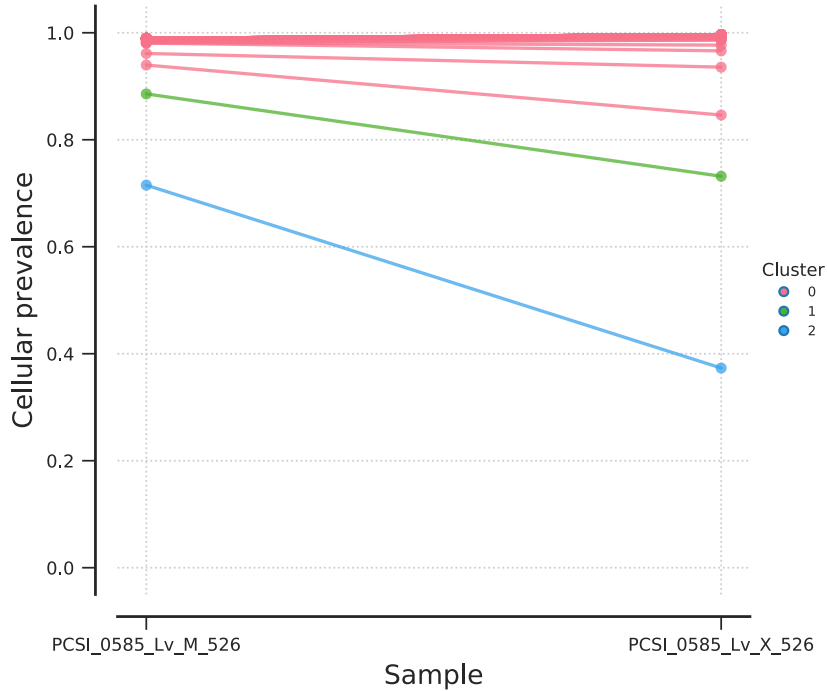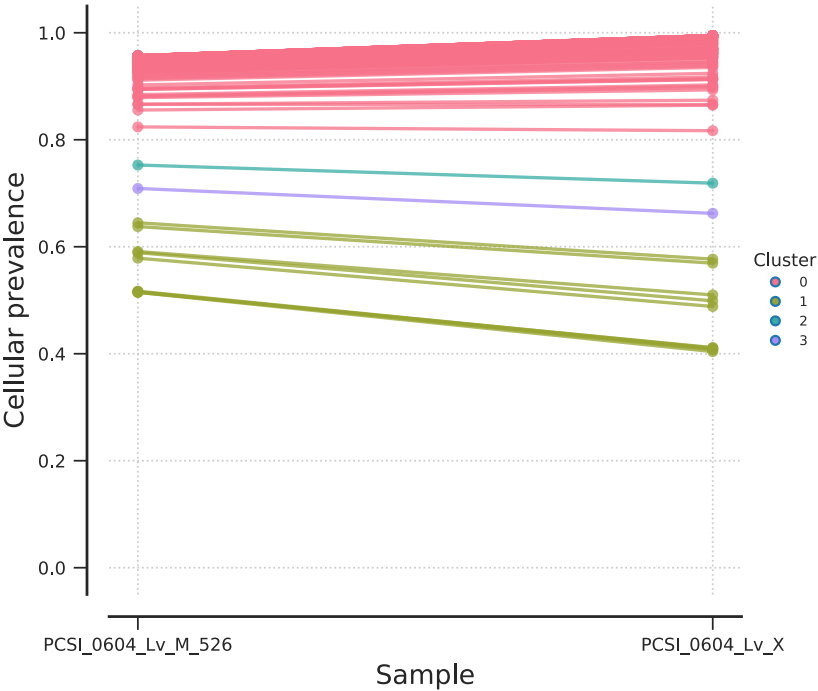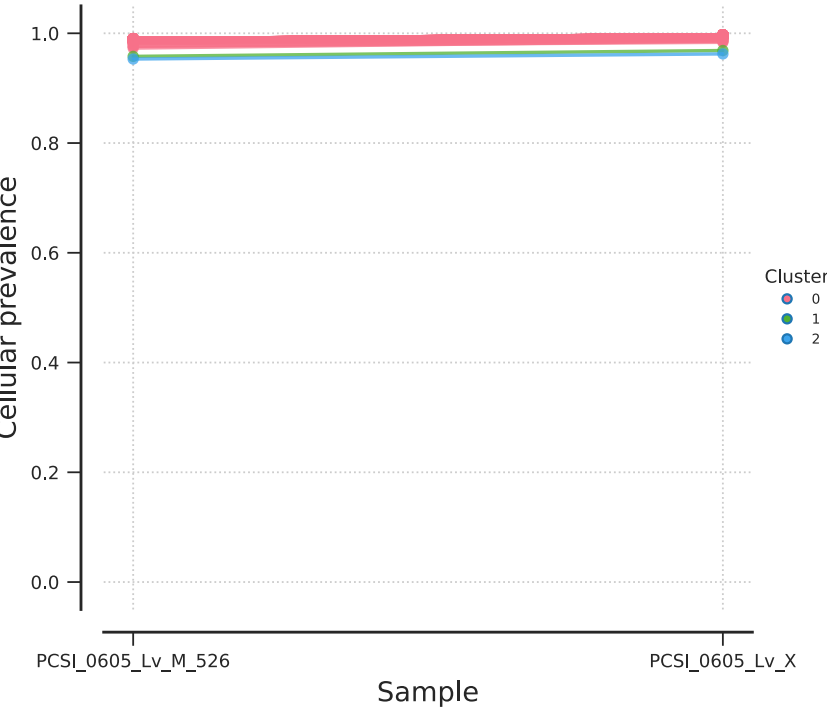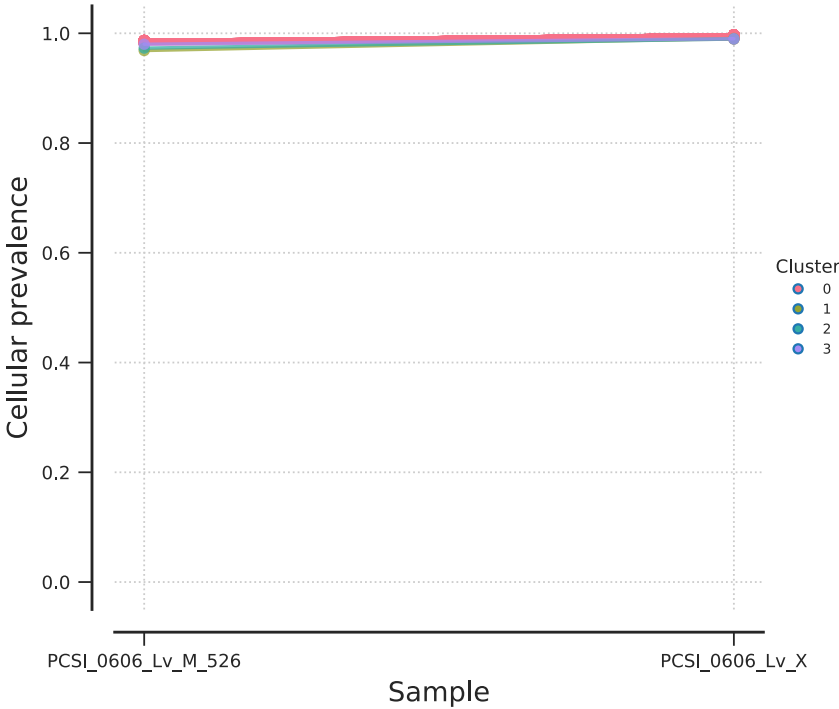

Supplement: S11 Fig — PyClone output for (A) 10 resected primary samples and (B) 6 liver metastasis samples. Each graph represents one sample, with matching PDX and PDO (where applicable) labelled. SNVs are clustered based on their common clonality, and colored by cluster. Points are drawn at the cellular prevalence of the SNV in each sample. (PDF) [file pcbi.1006596.s011.pdf]

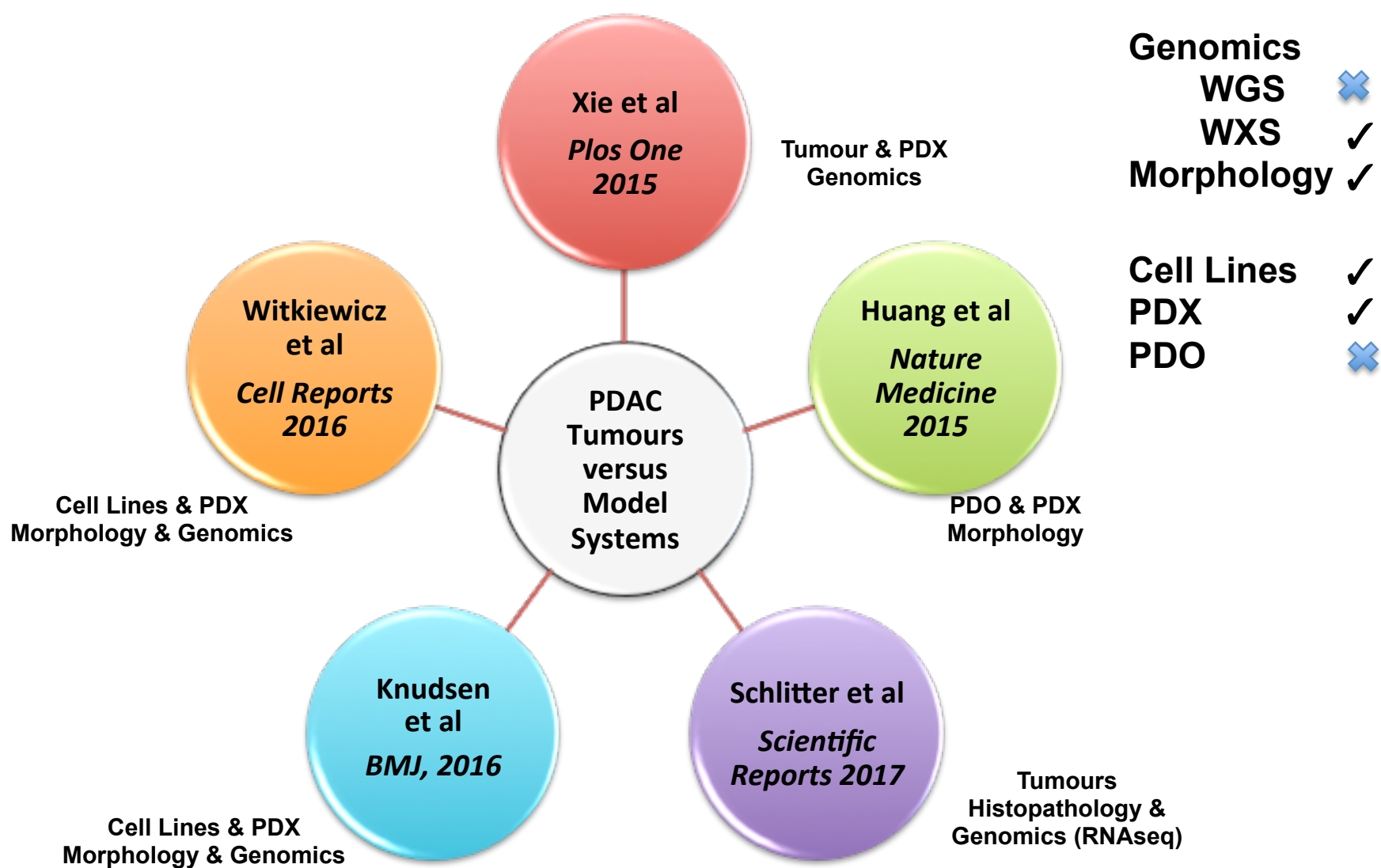

Supplement: S12 Fig — (PDF) [file pcbi.1006596.s012.pdf]
