# Supplementary material for: Whole genomes define concordance of matched primary, xenograft, and organoid models of pancreas cancer
Source: PLoS Comput Biol. 2019 Jan 10;15(1):e1006596. doi: 10.1371/journal.pcbi.1006596 (PMC6328084; doi:10.1371/journal.pcbi.1006596)

PCSI 0169 - Tumour

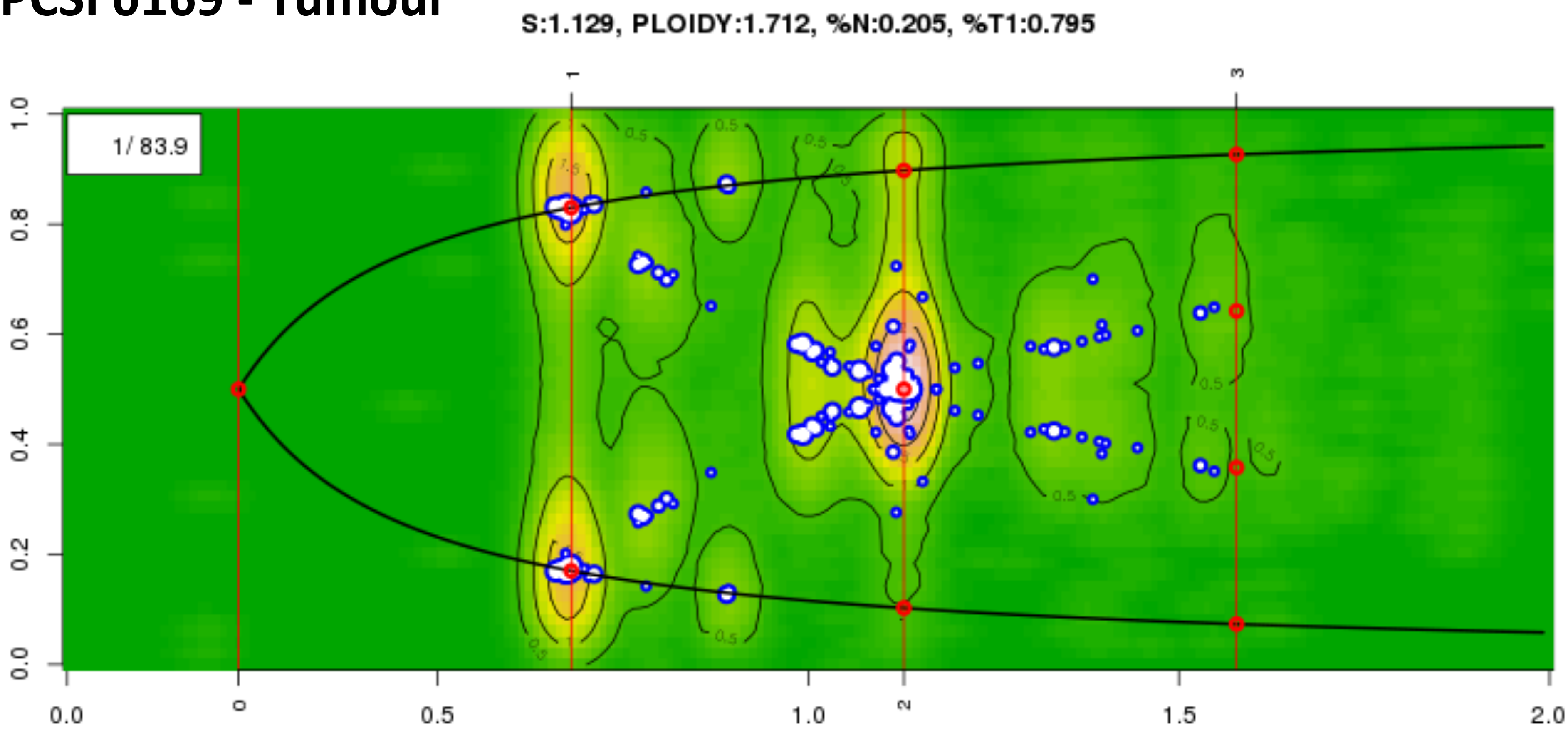

PCSI 0169 - Xenograft

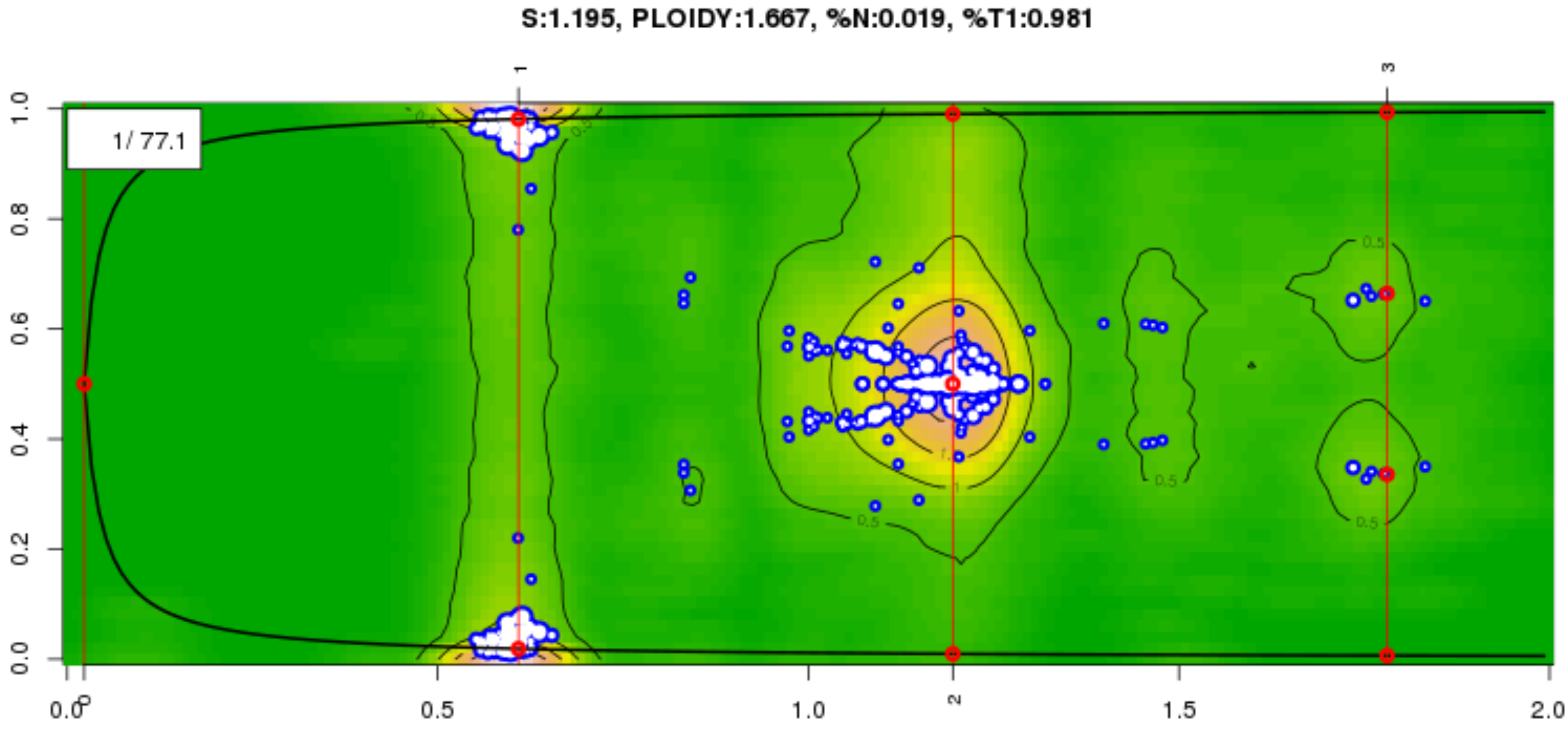

PCSI 0355 - Tumour

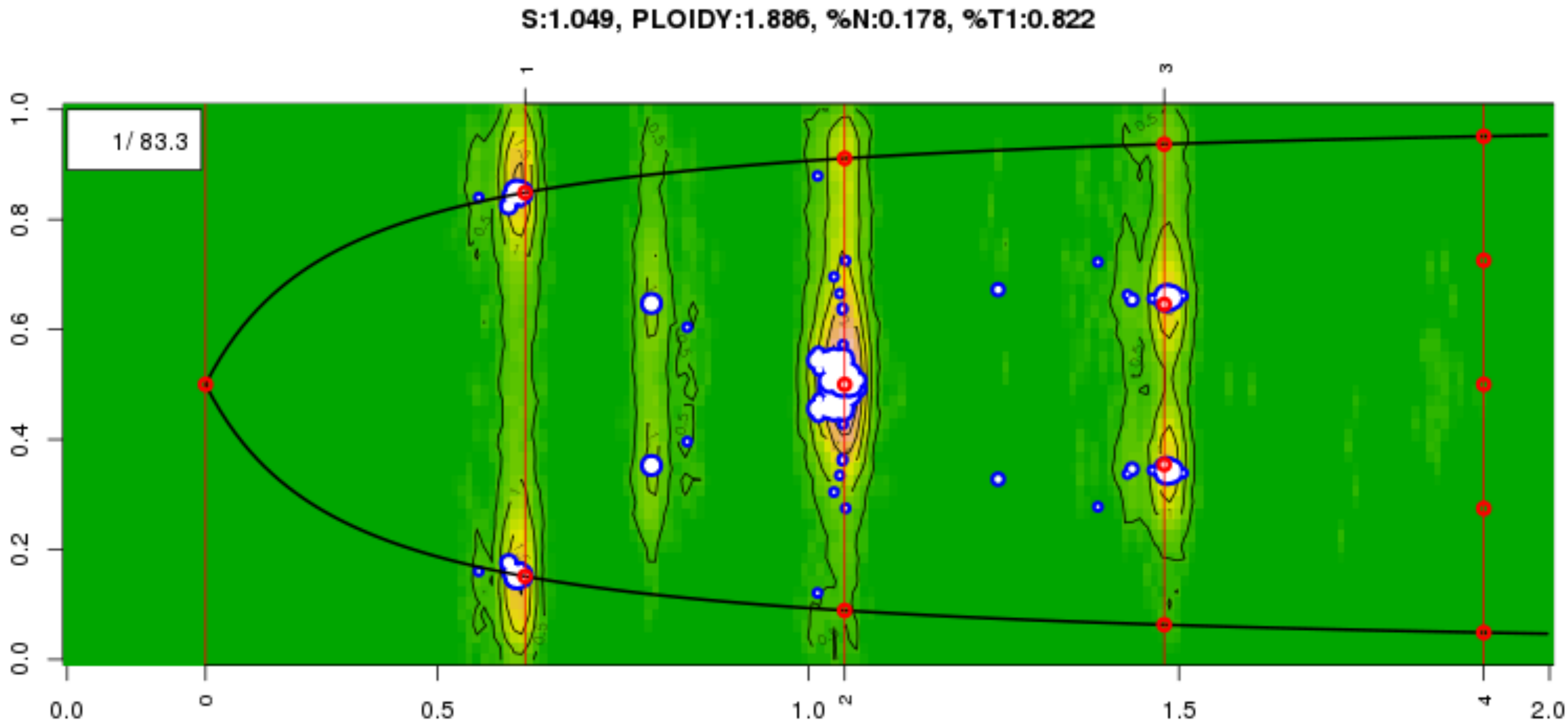

PCSI 0355 - Xenograft

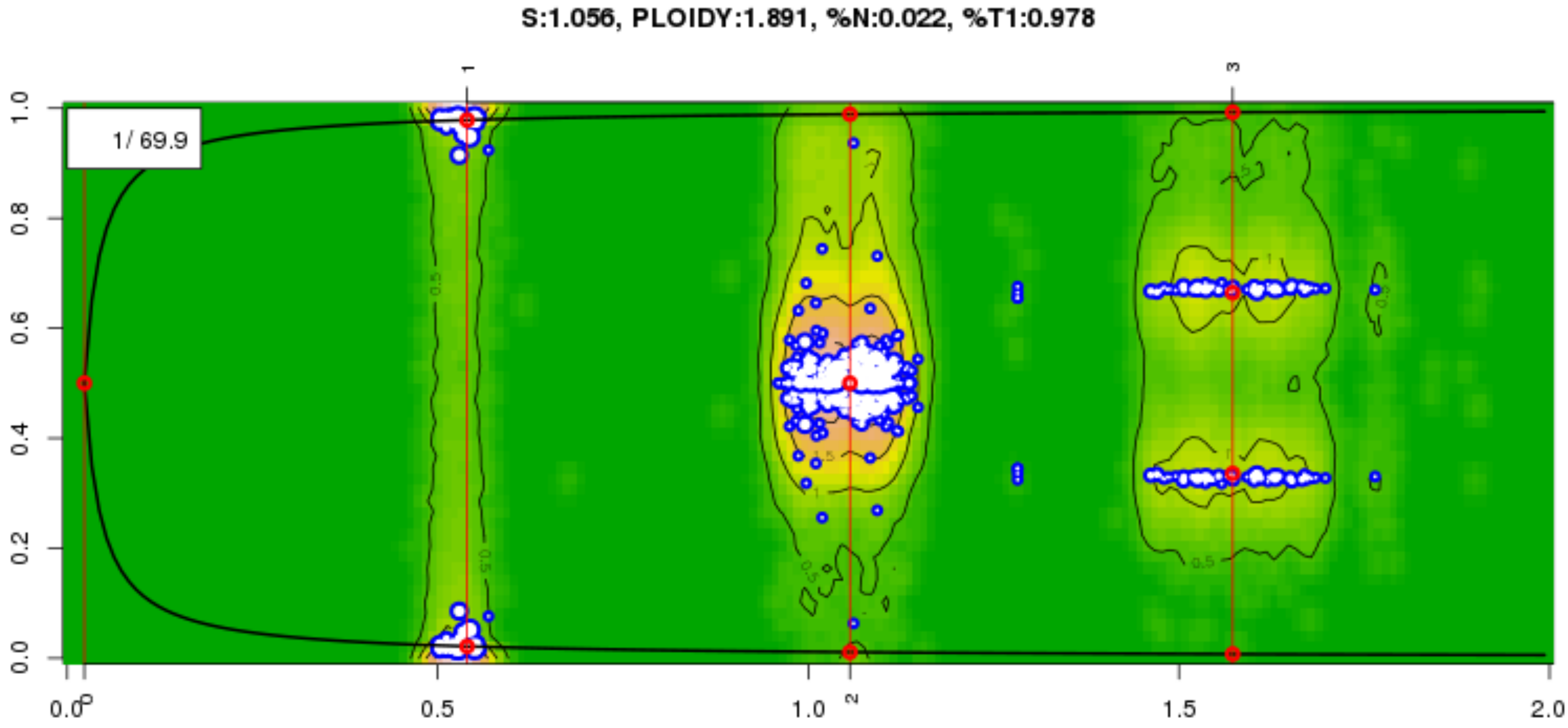

PCSI 0589 - Tumour

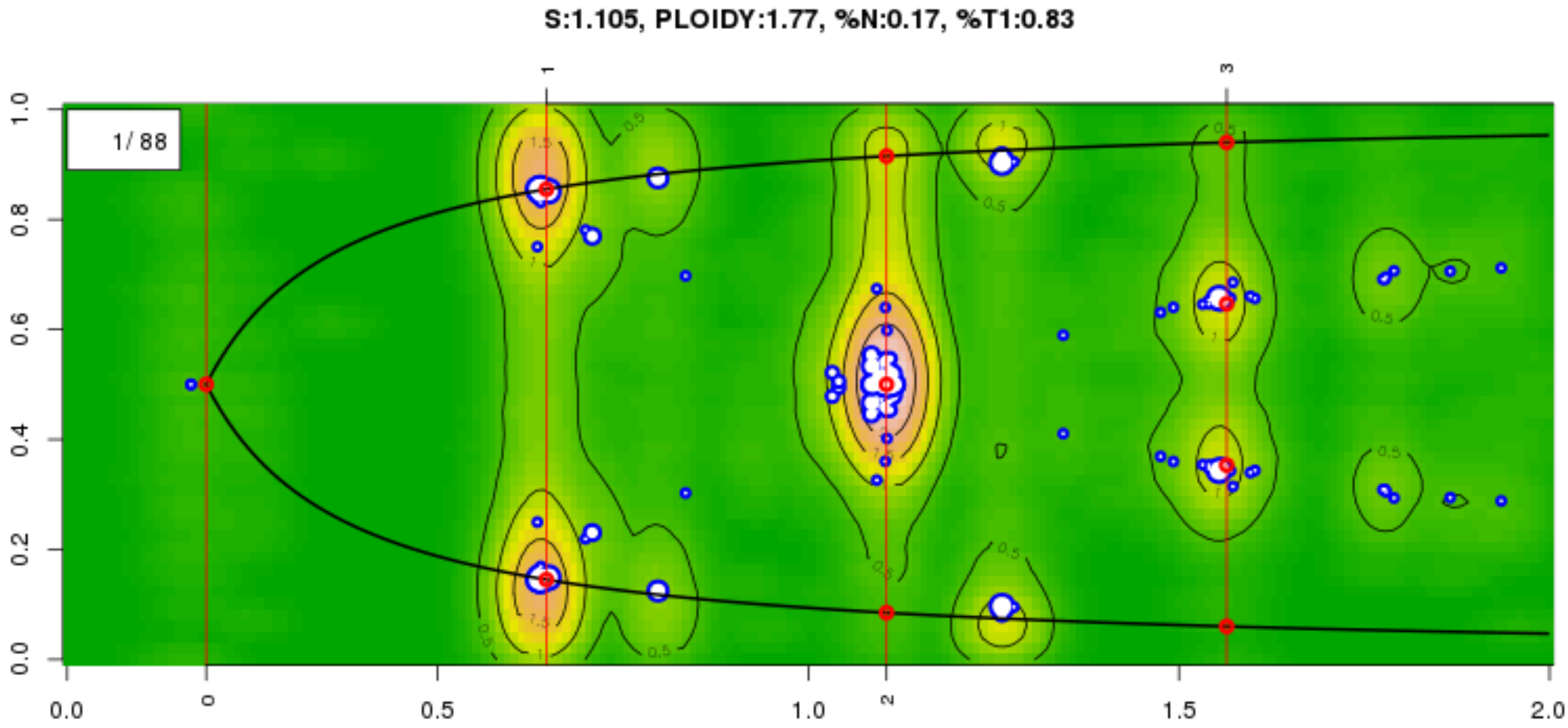

PCSI 0589 - Xenograft

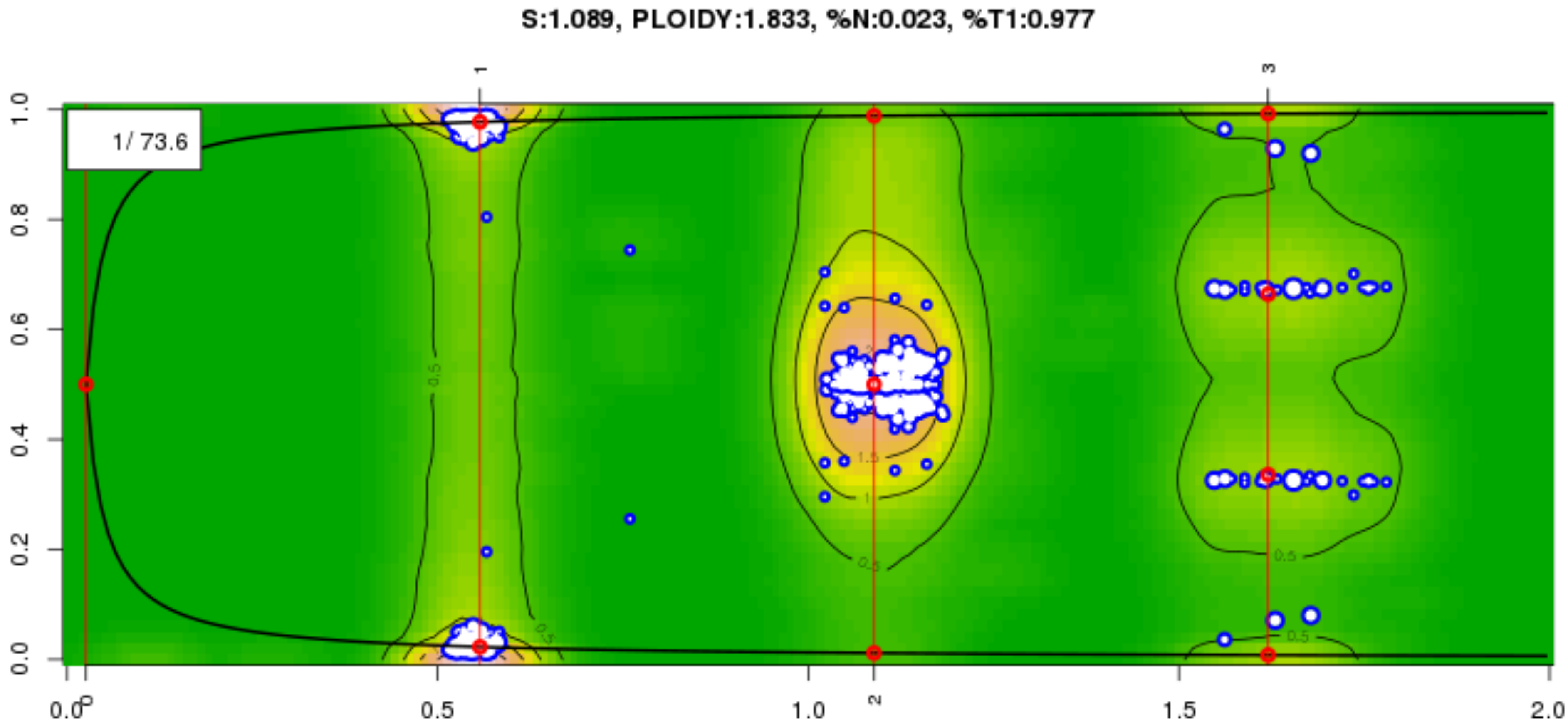

PCSI 0590 - Tumour

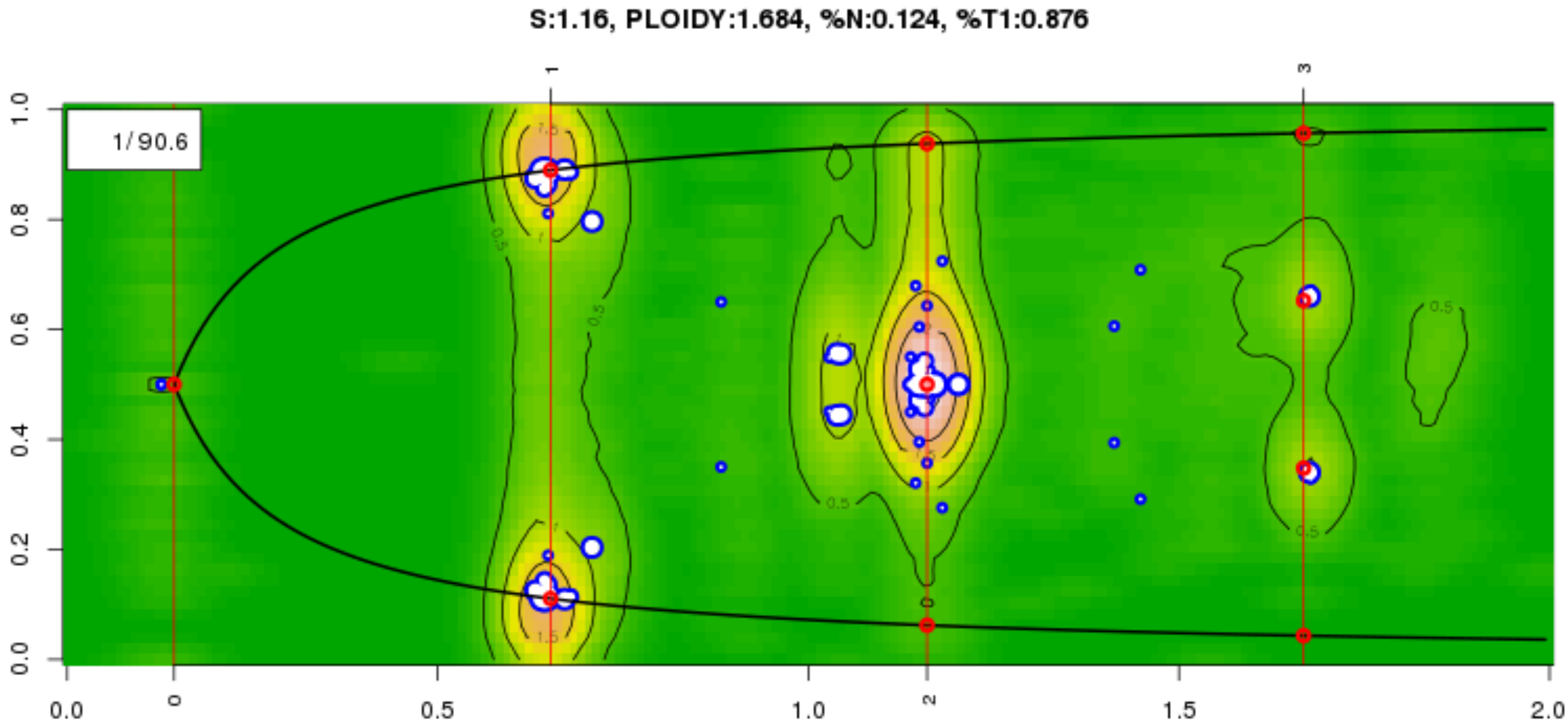

PCSI 0590 - Xenograft

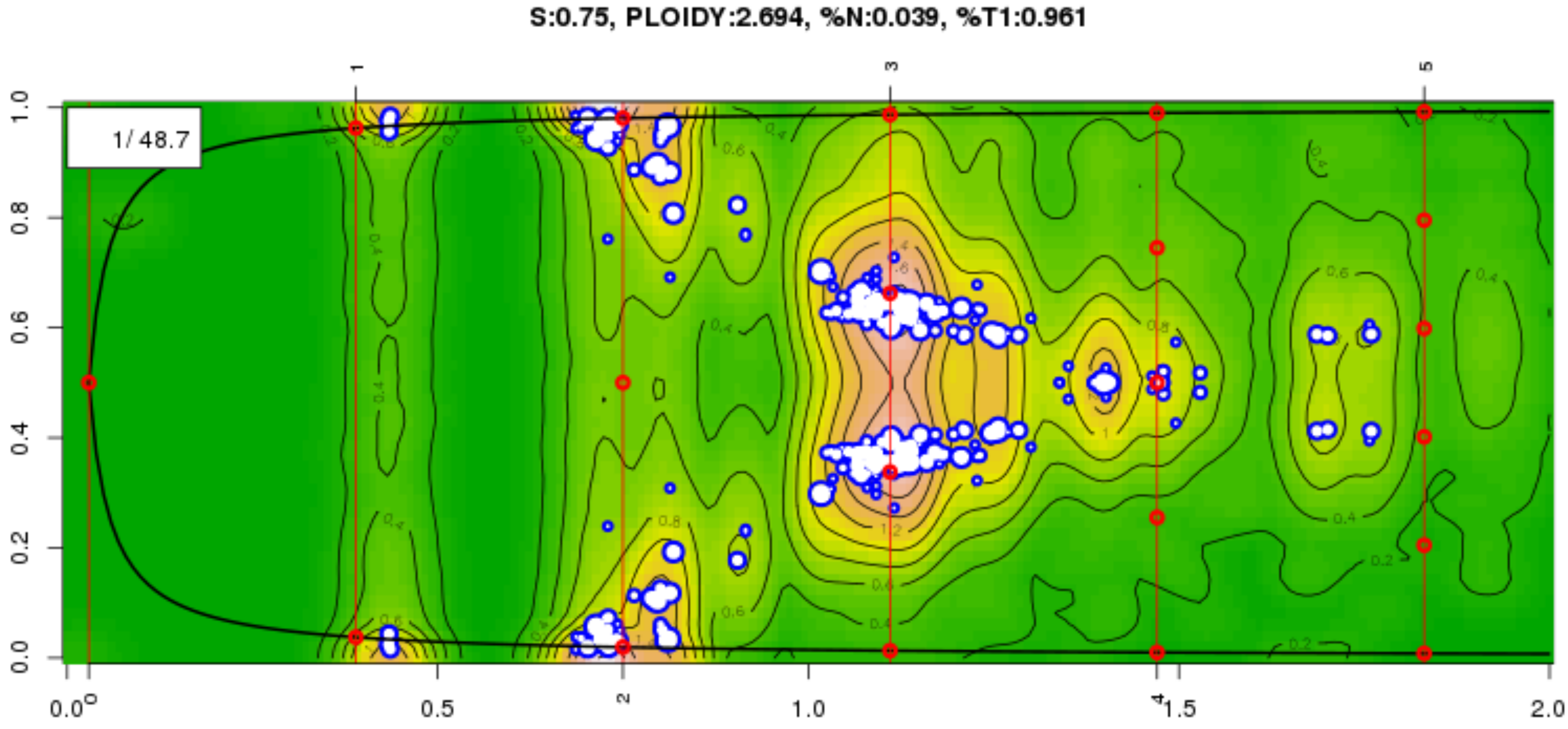

PCSI 0611 - Tumour

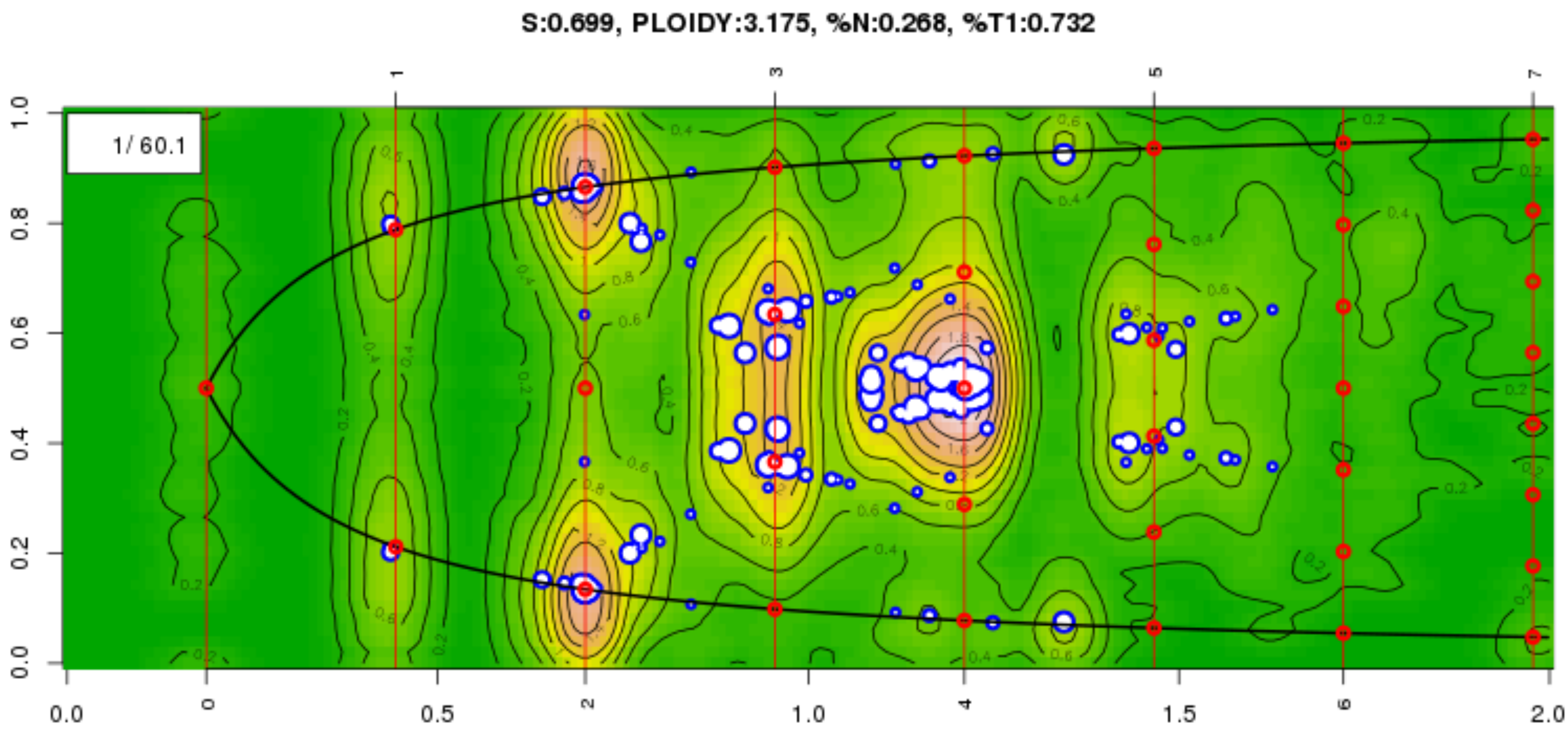

PCSI 0611 - Xenograft

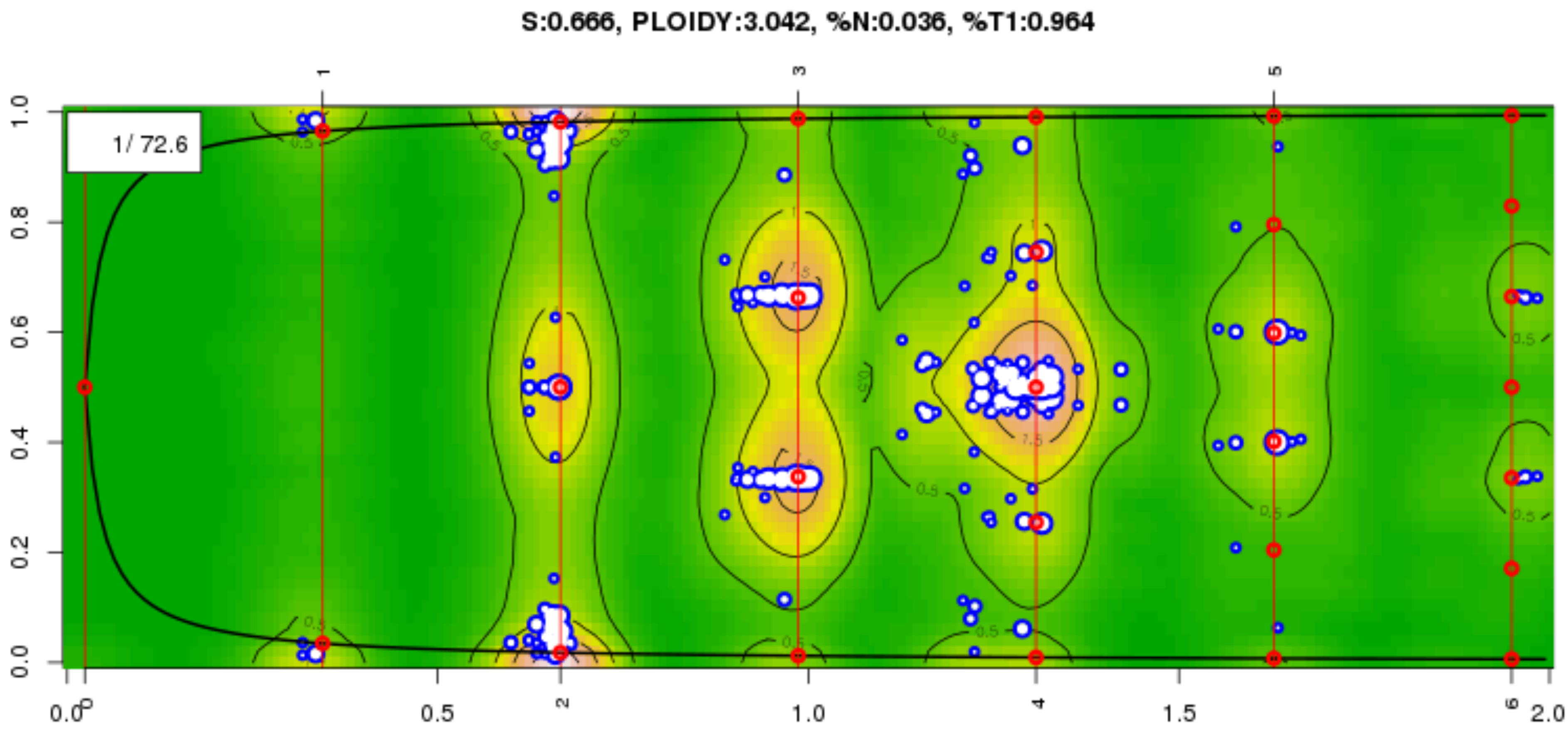

PCSI 0624 - Tumour

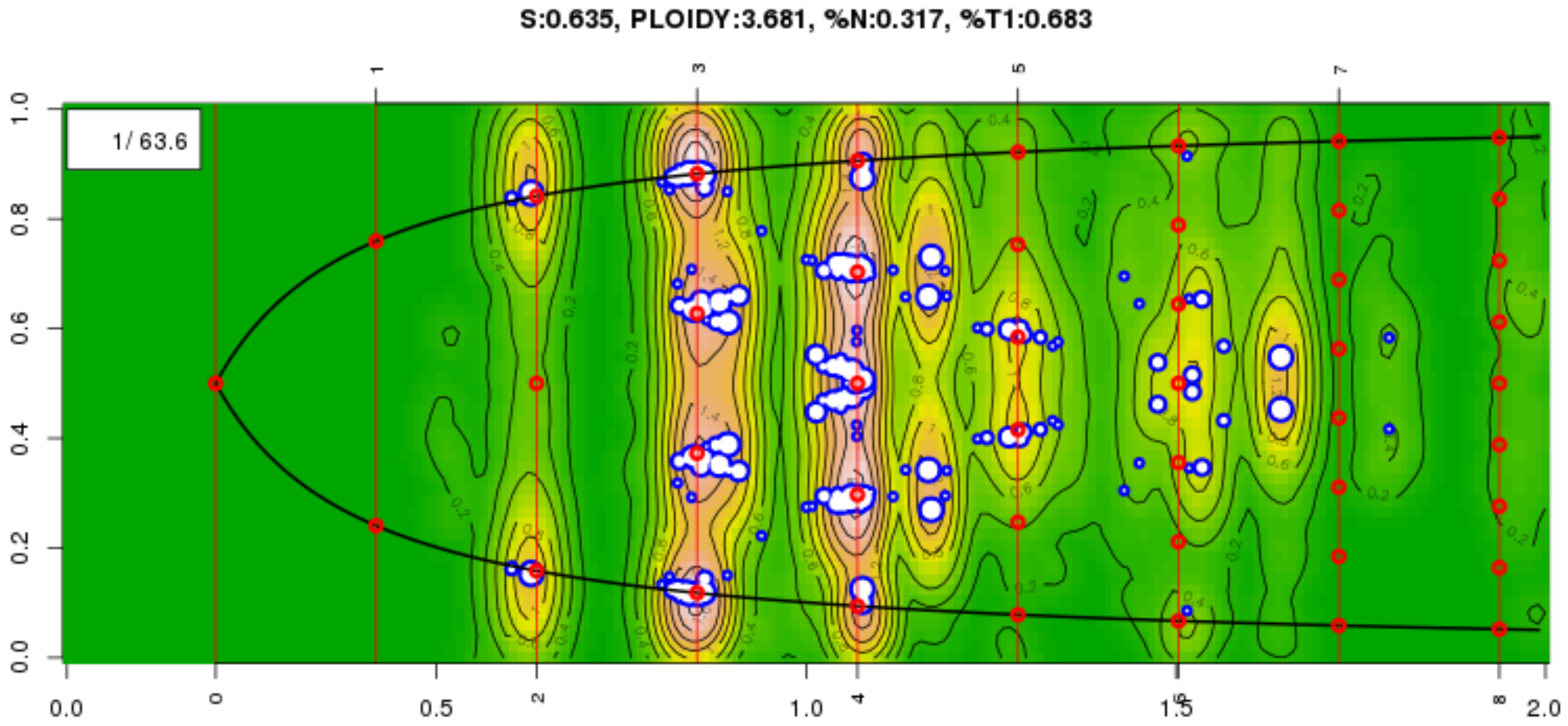

PCSI 0624 - Xenograft

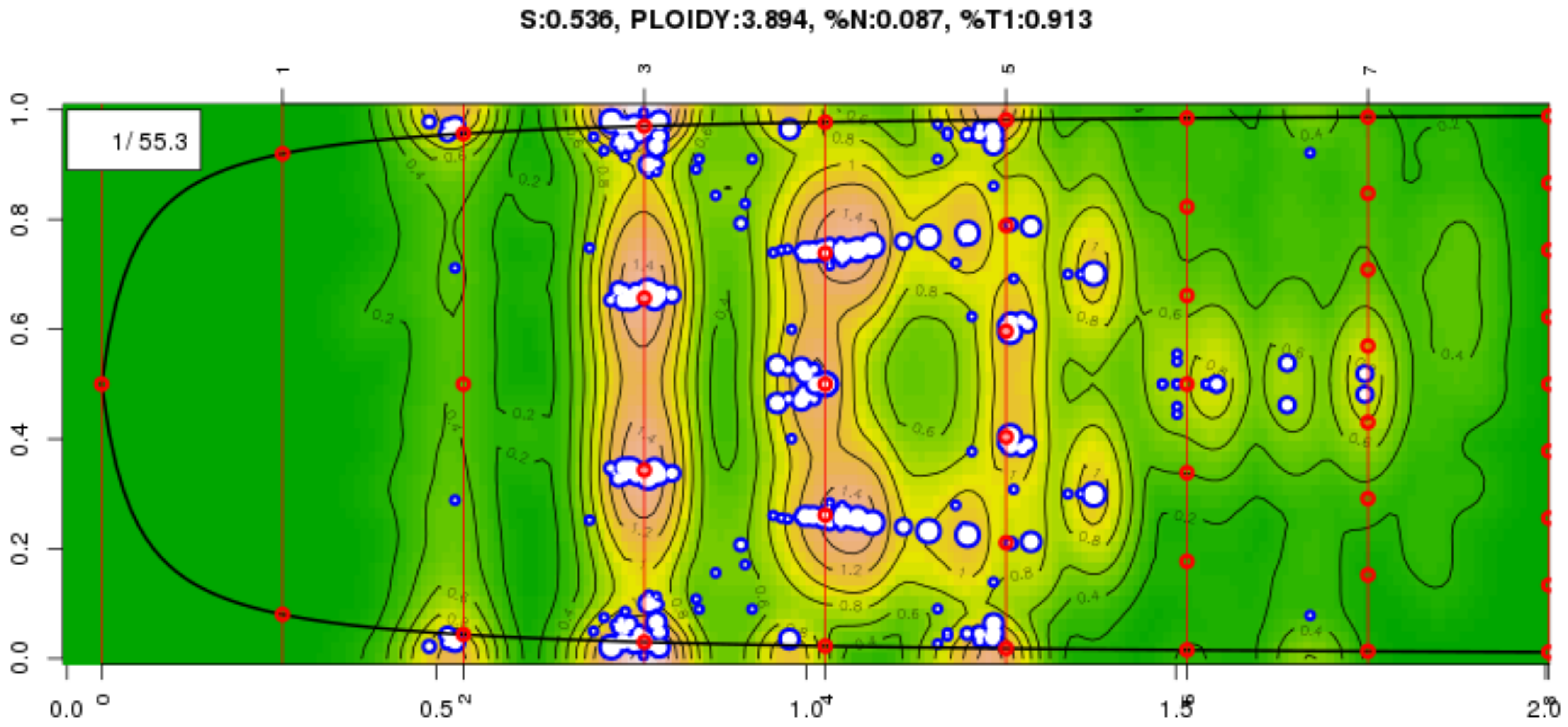

PCSI 0633- Tumour

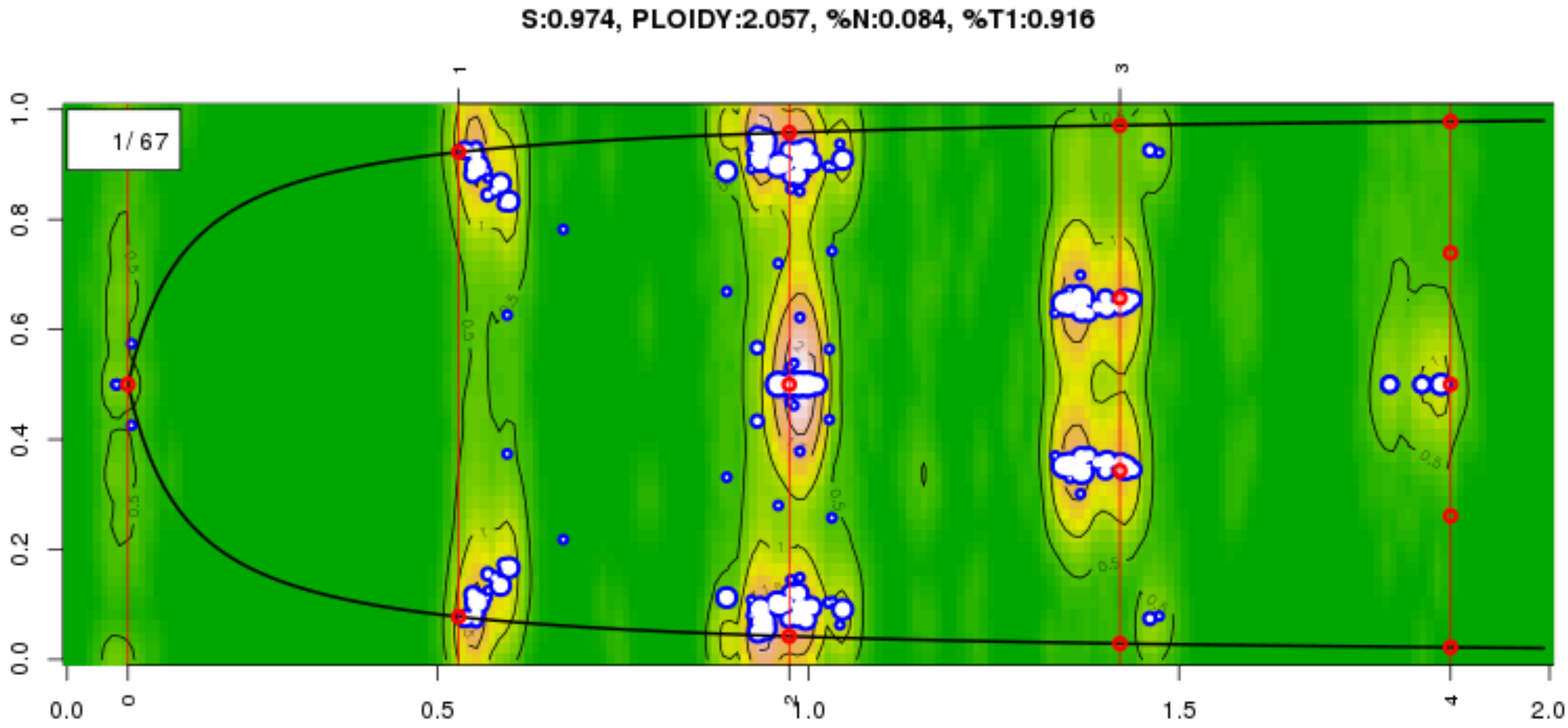

PCSI 0633 - Xenograft

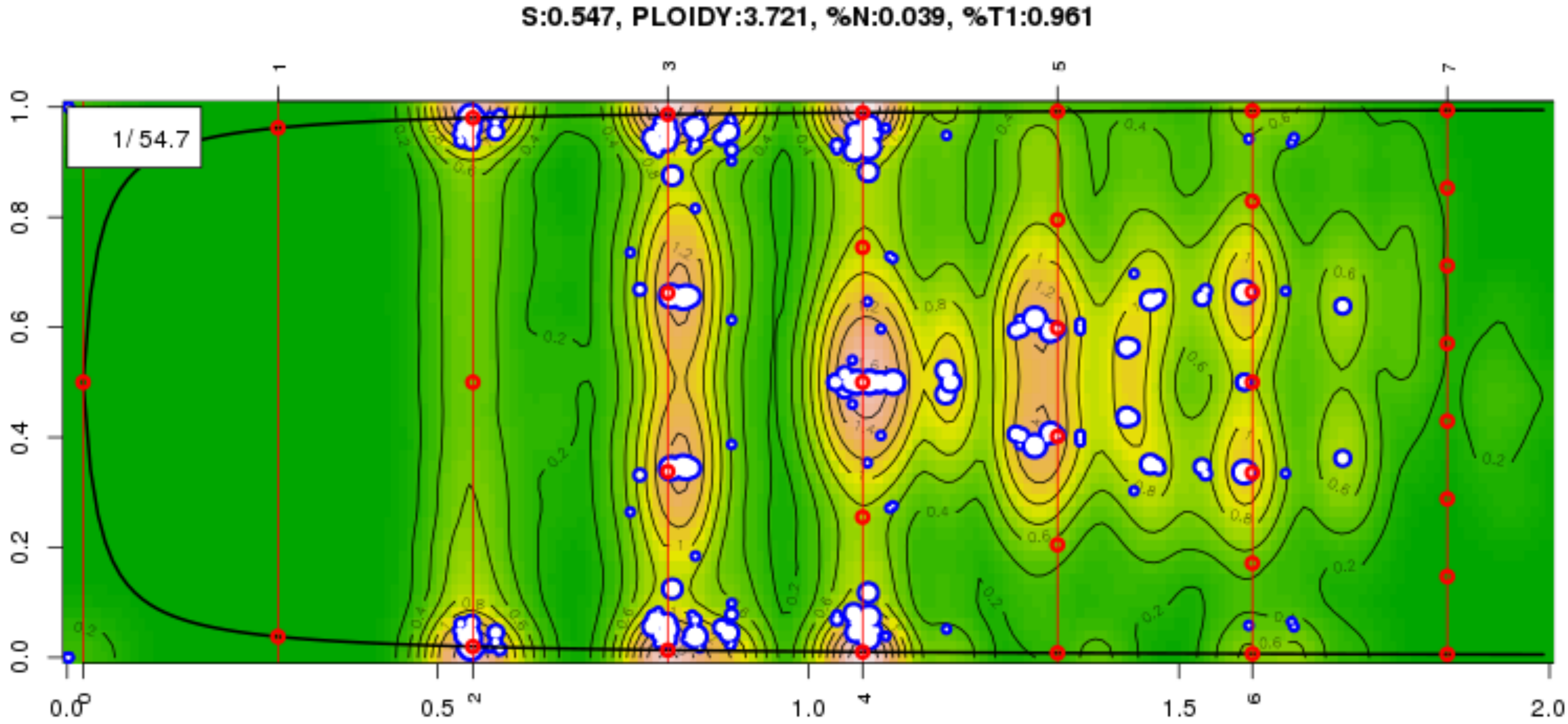

PCSI 0642- Tumour

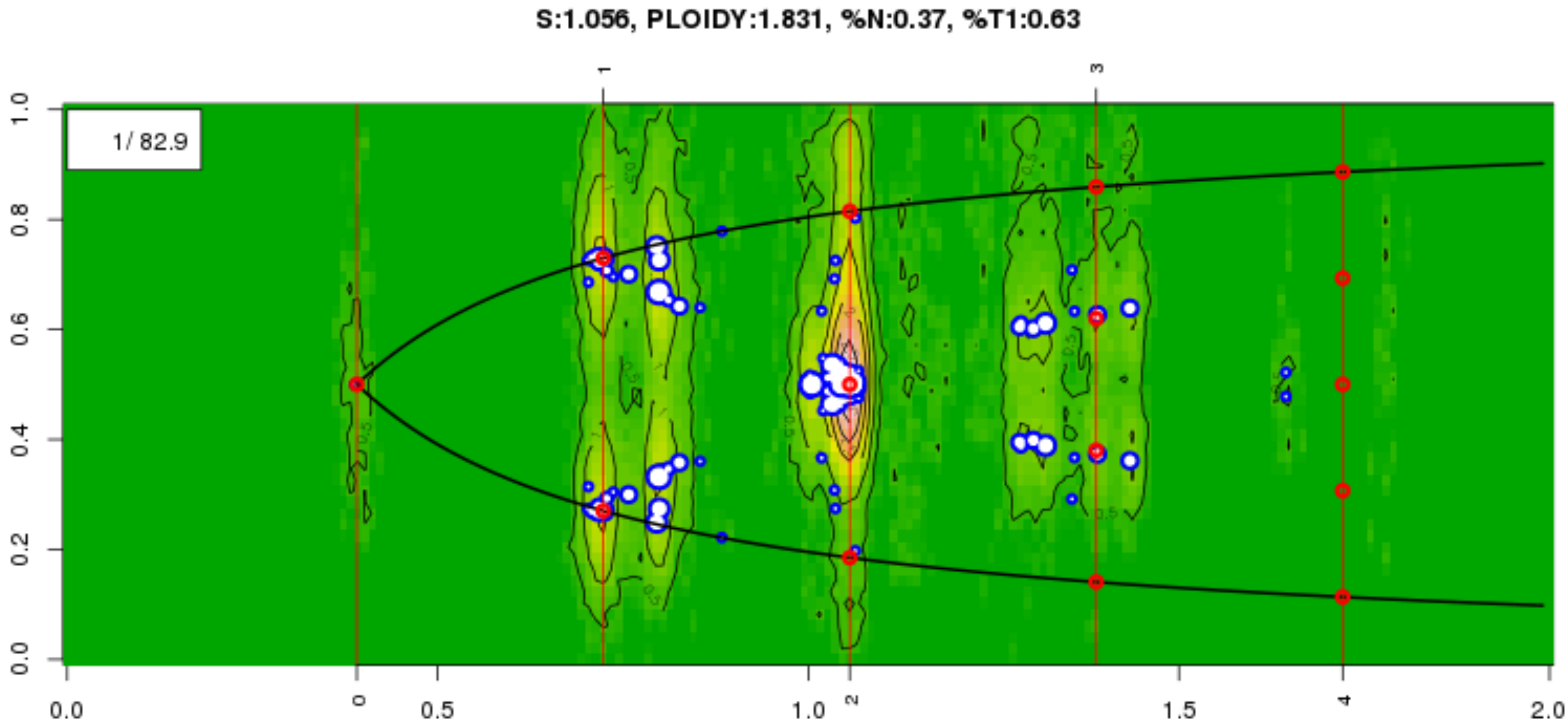

PCSI 0642 - Xenograft

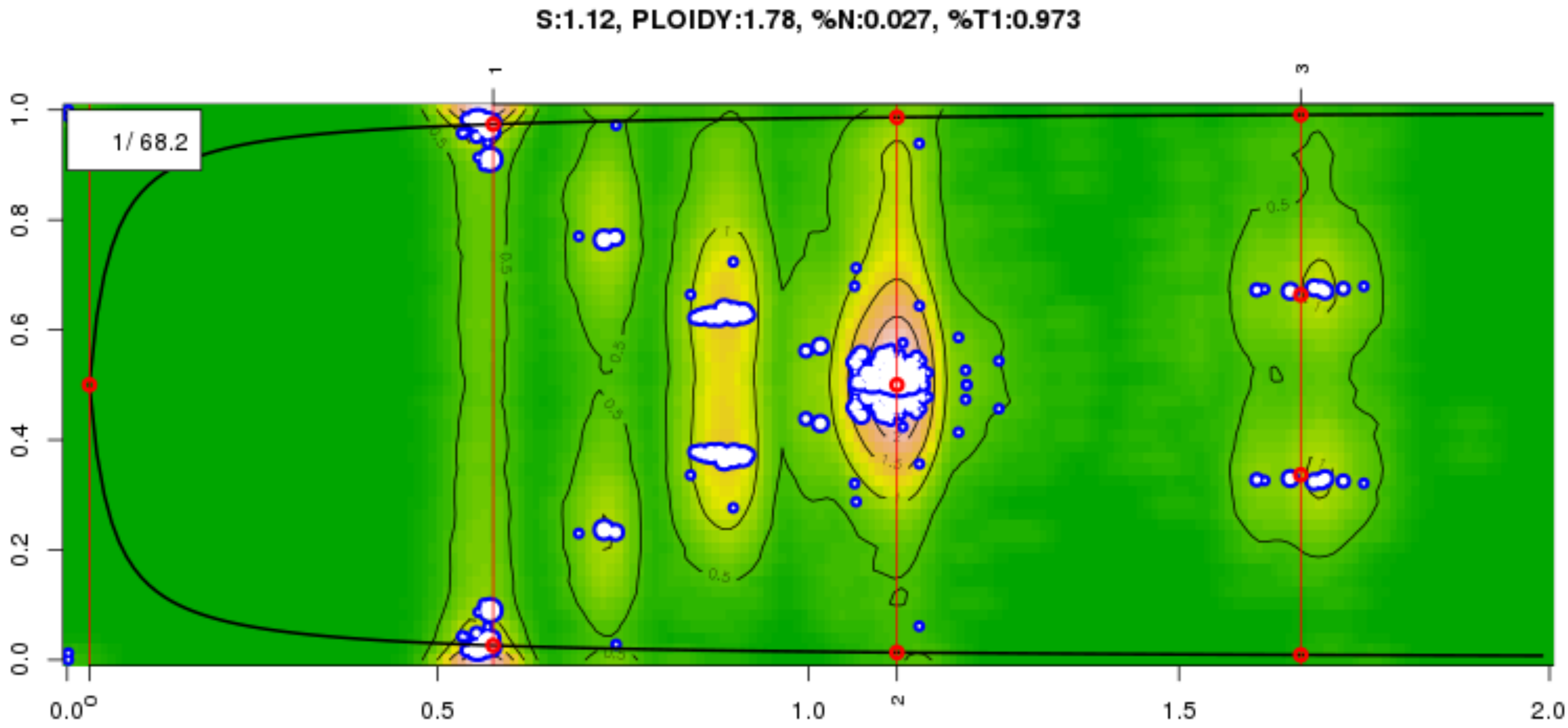

PCSI 0489

Liver Metastasis

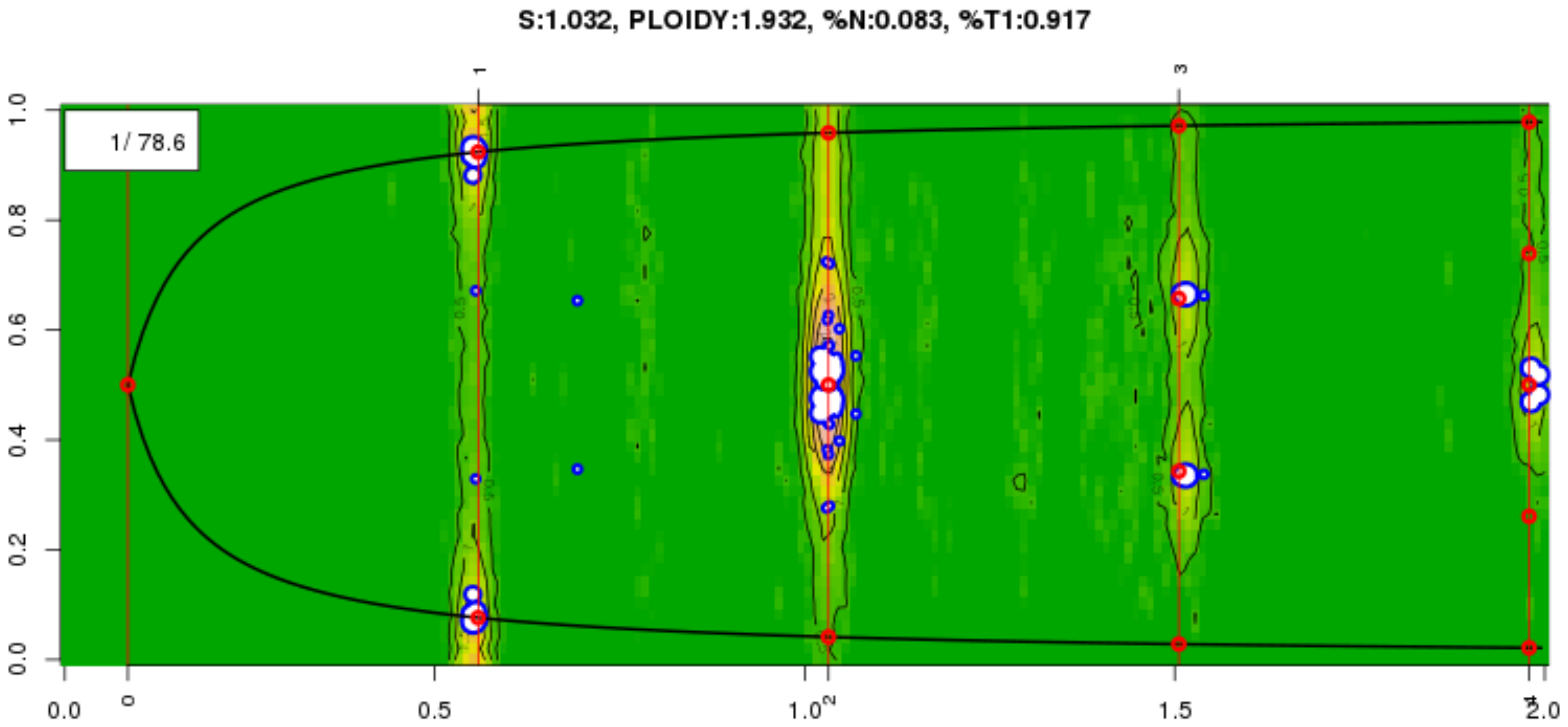

Xenograft

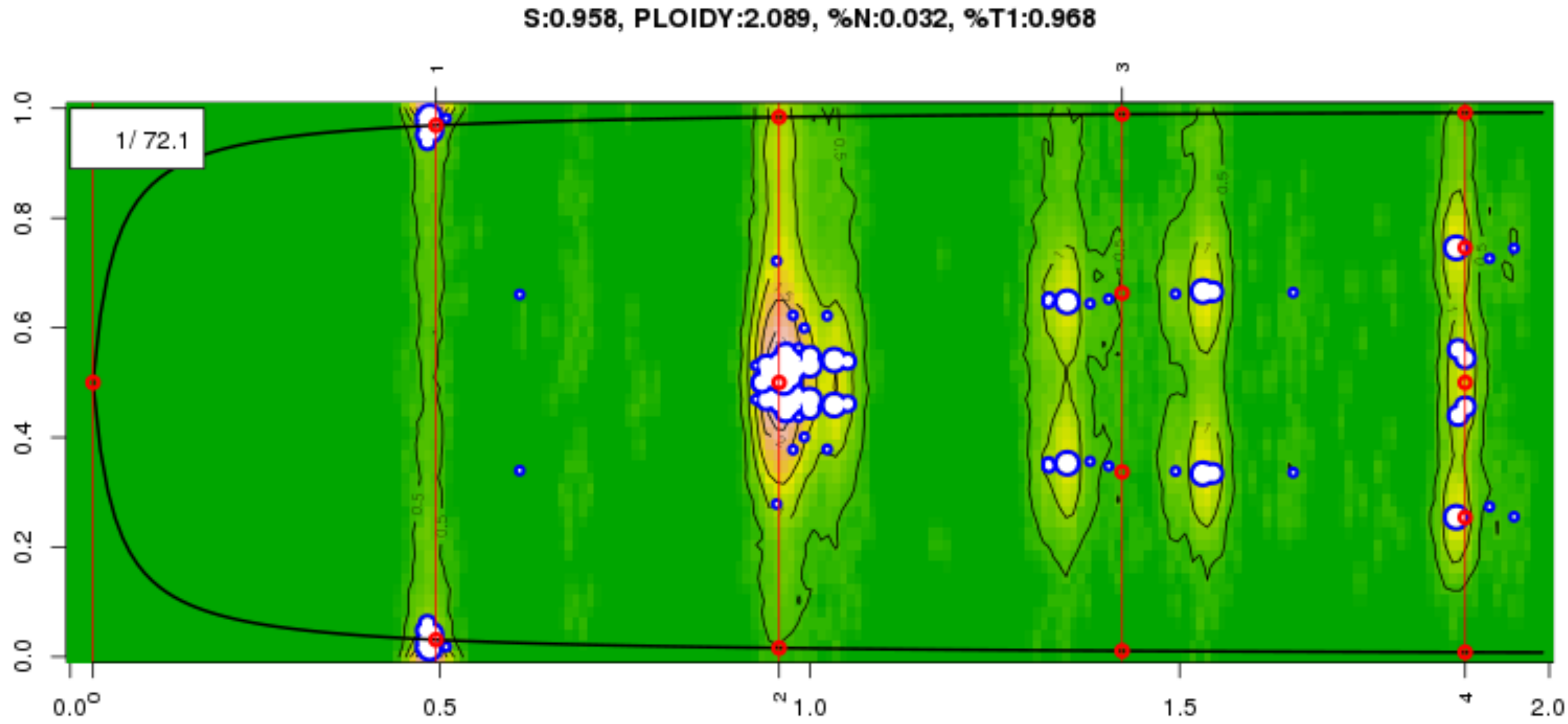

PCSI 0491

Liver Metastasis

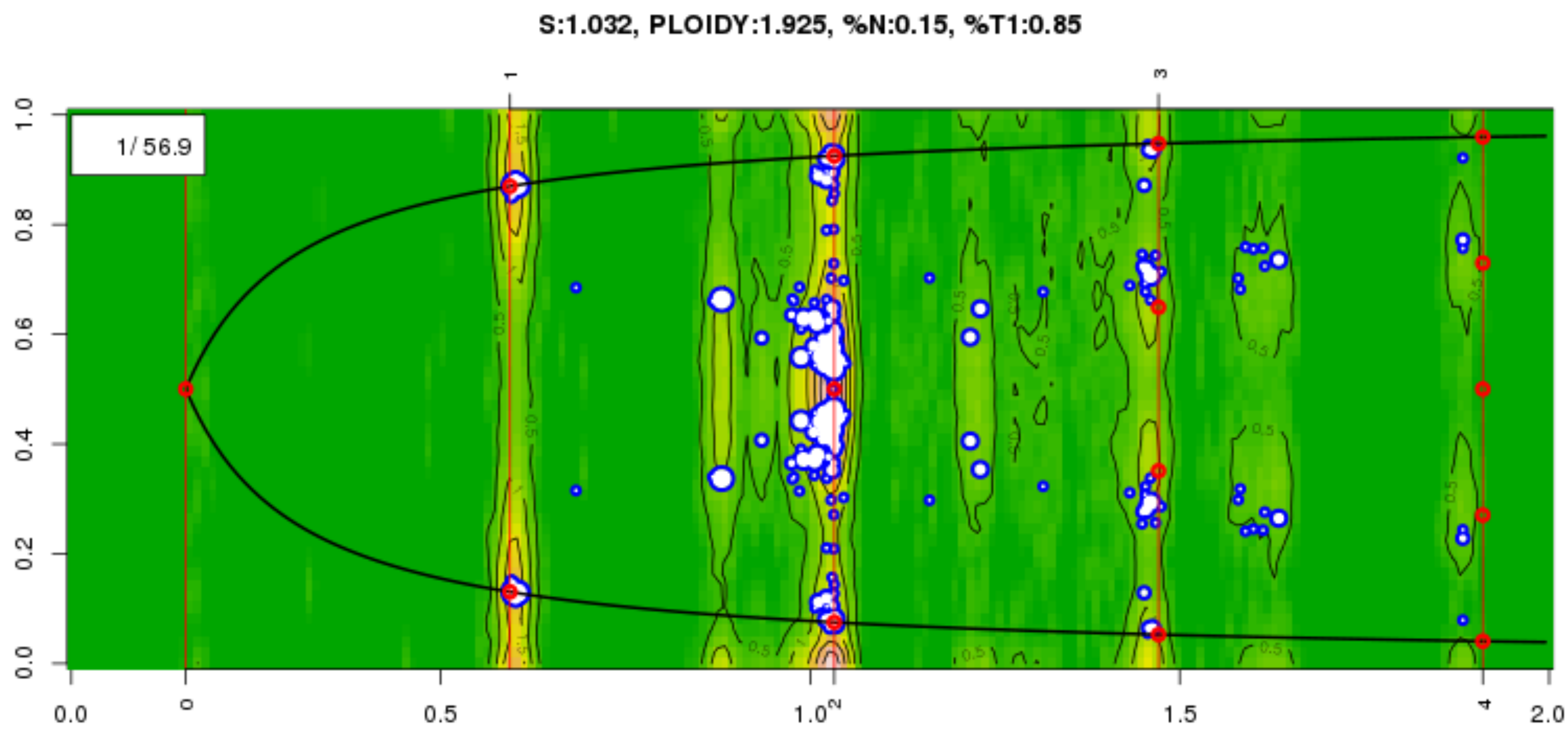

Xenograft

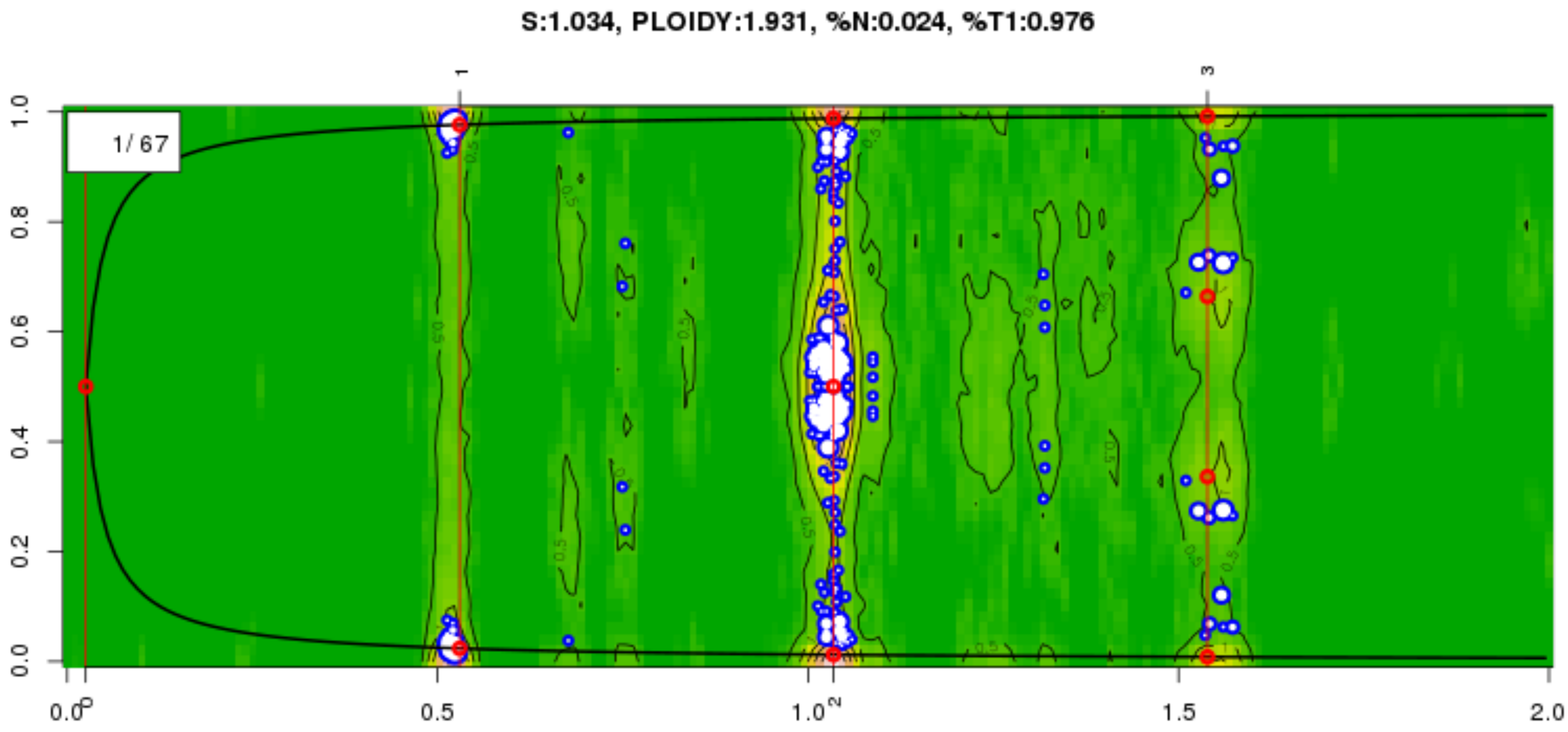

PCSI 0585

Liver Metastasis

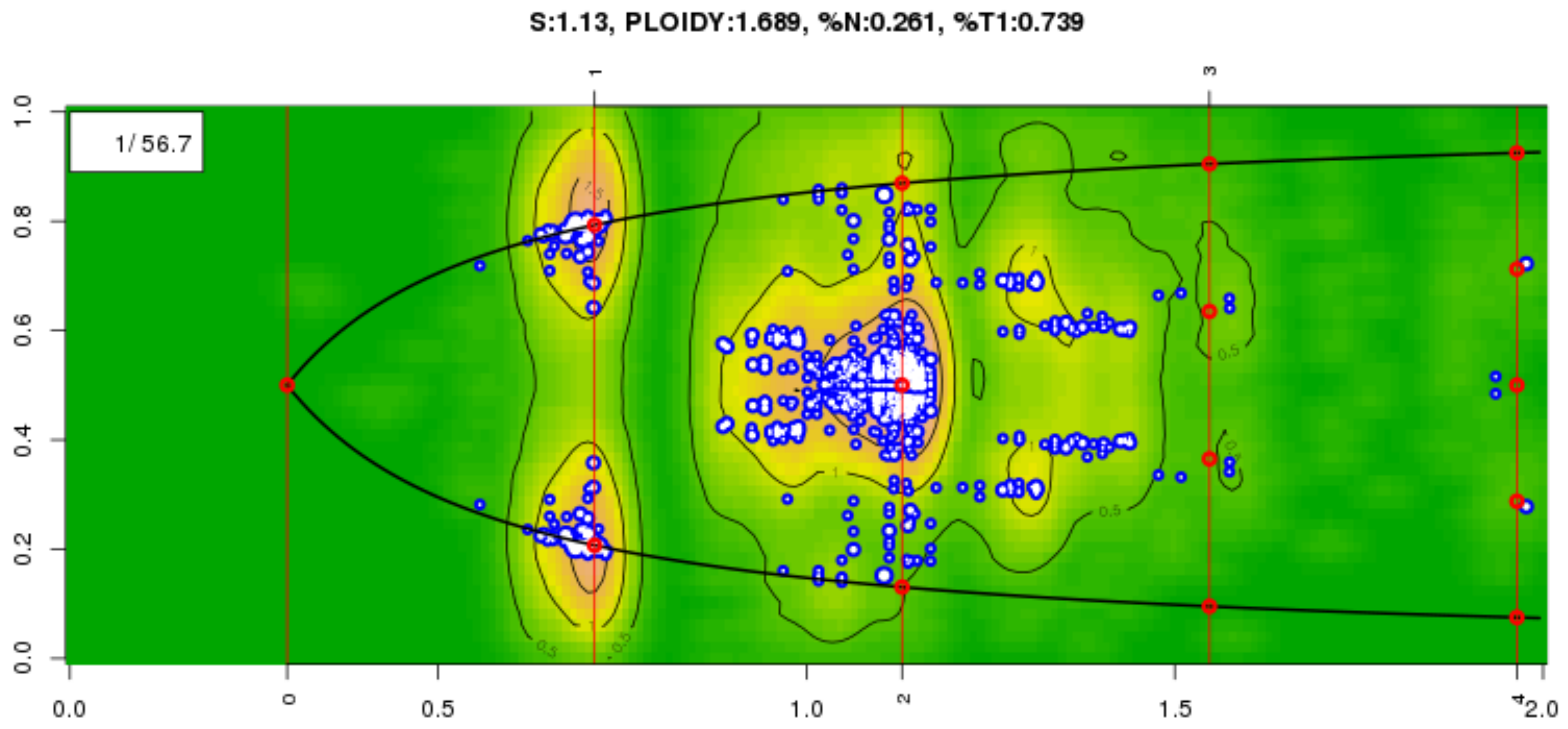

Xenograft

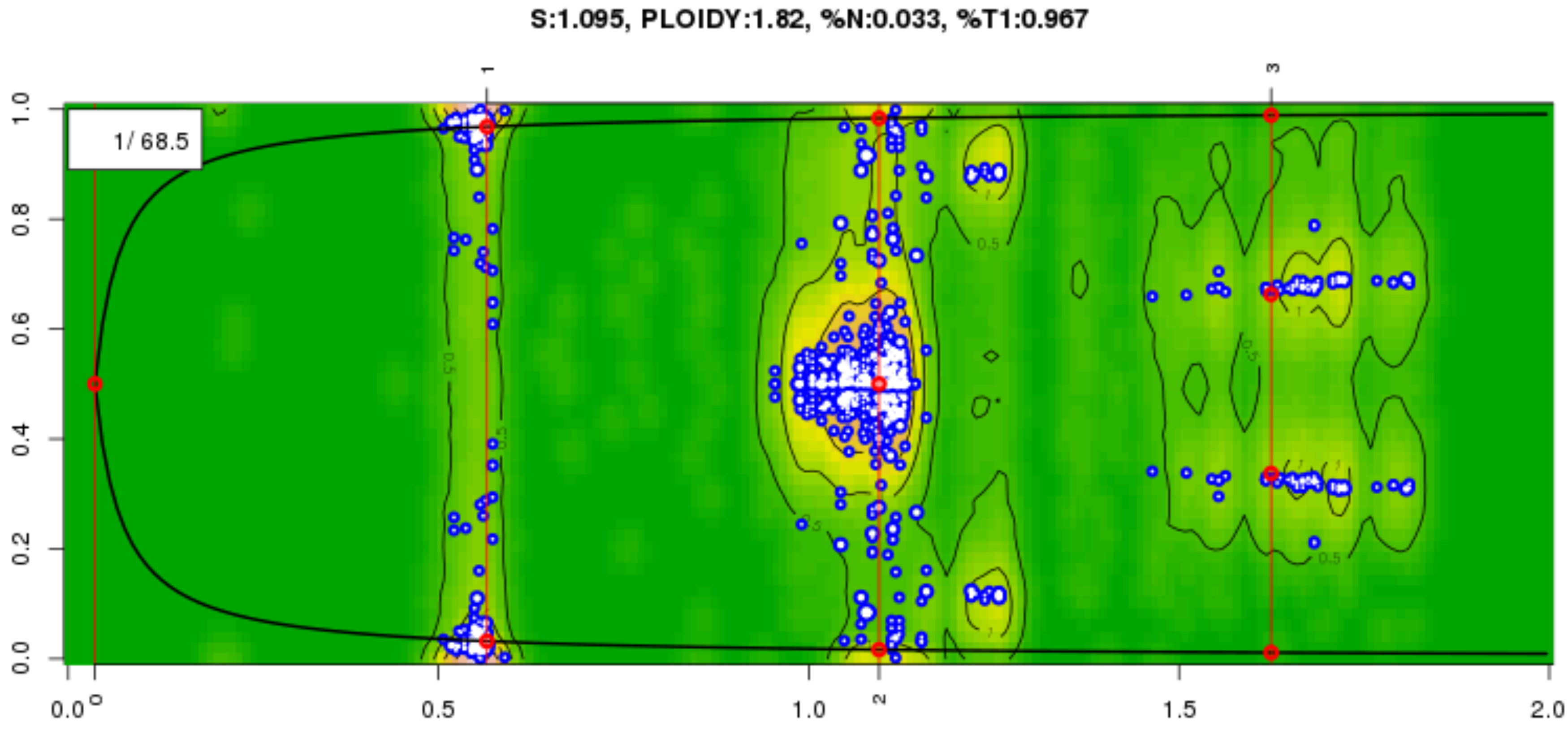

PCSI 0604

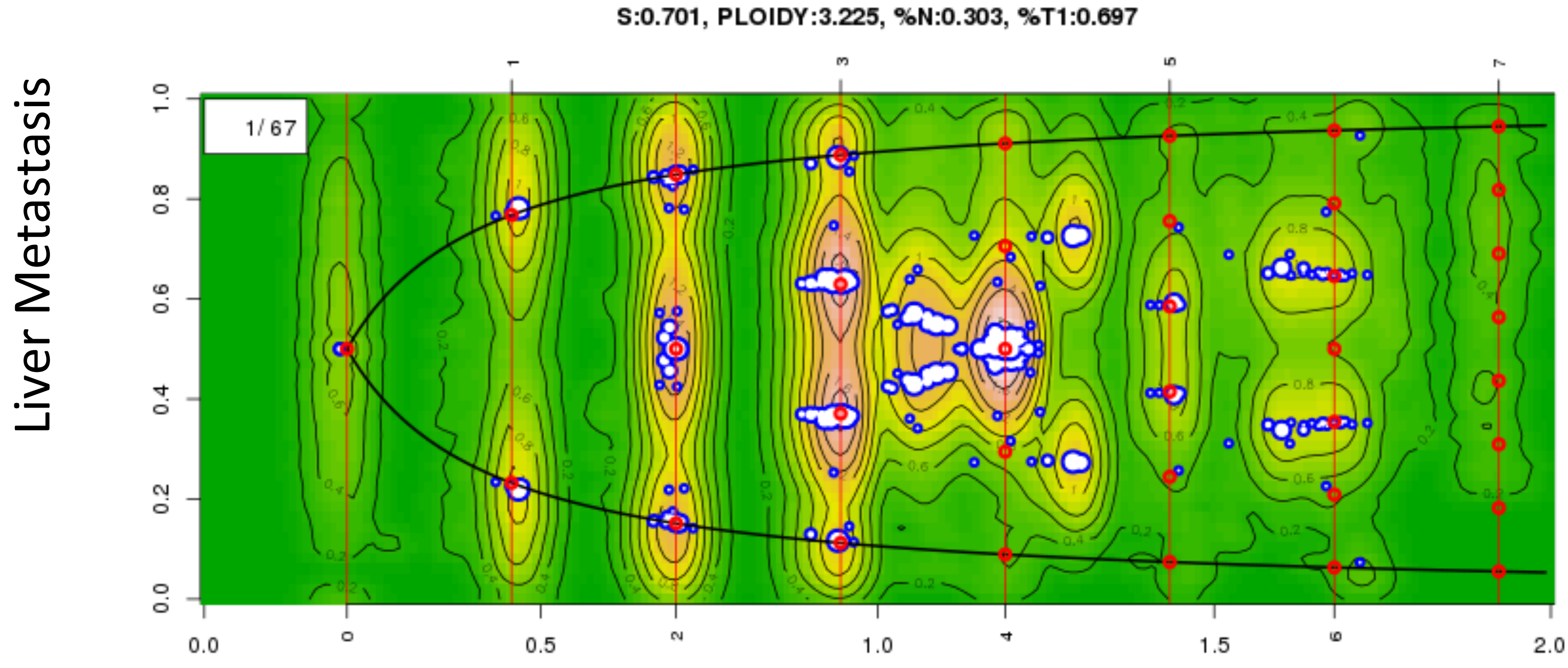
Liver Metastasis
S:0.701, PLOIDY:3.225, %N:0.303, %T1:0.697
1/ 67
0.0
0.5
1.0
1.5
2.0

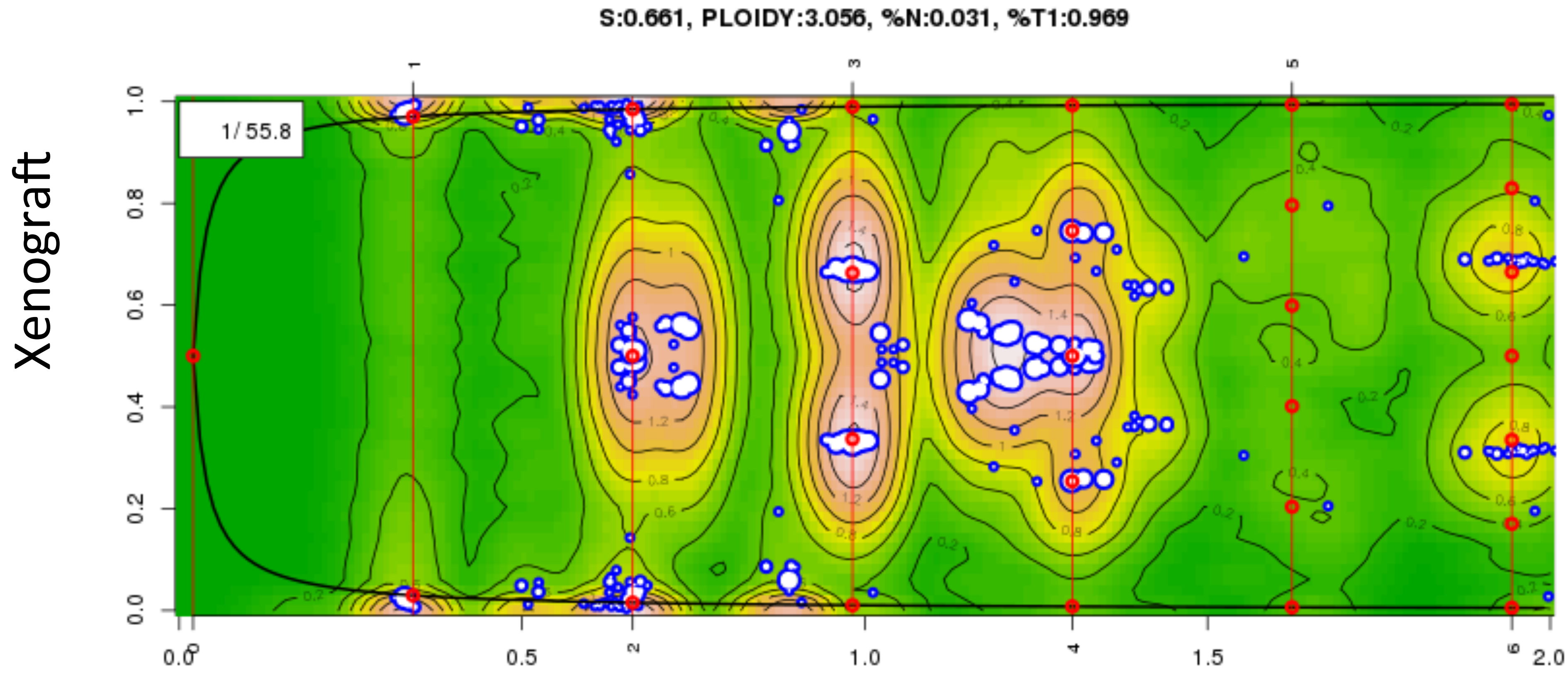
Xenograft
S:0.661, PLOIDY:3.056, %N:0.031, %T1:0.969
1/ 55.8
0.0
0.5
1.0
1.5
2.0

PCSI 0605

Liver Metastasis

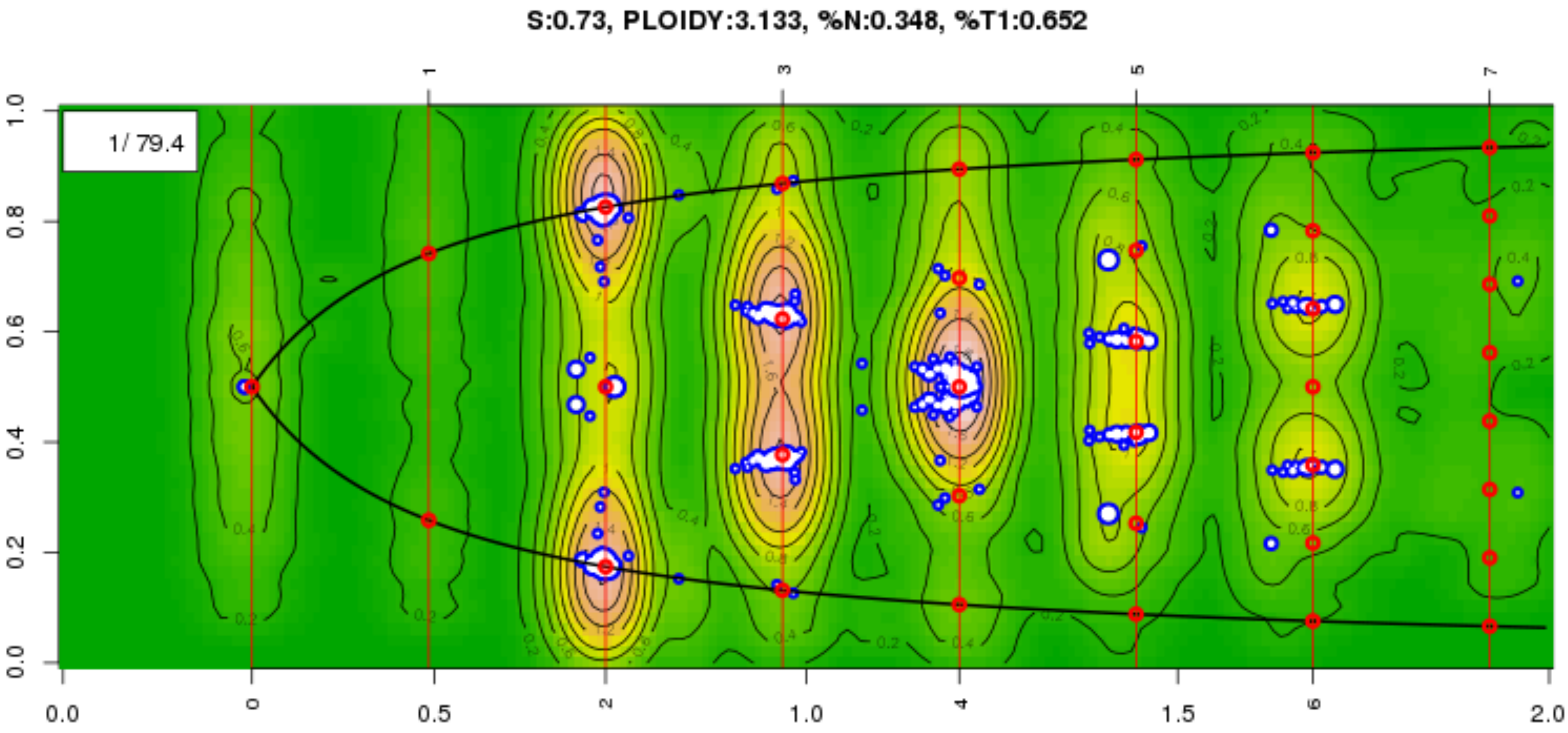

Xenograft

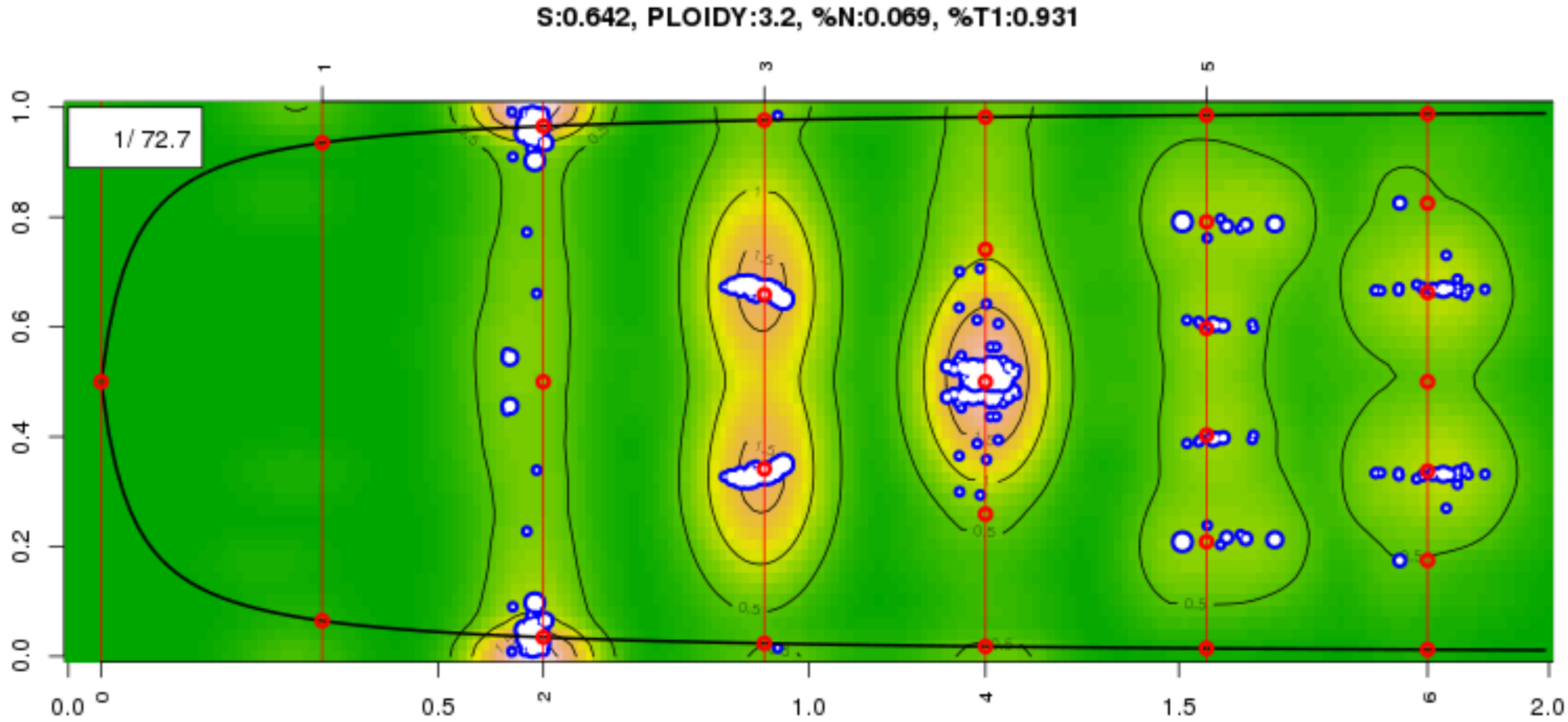

PCSI 0606

Liver Metastasis

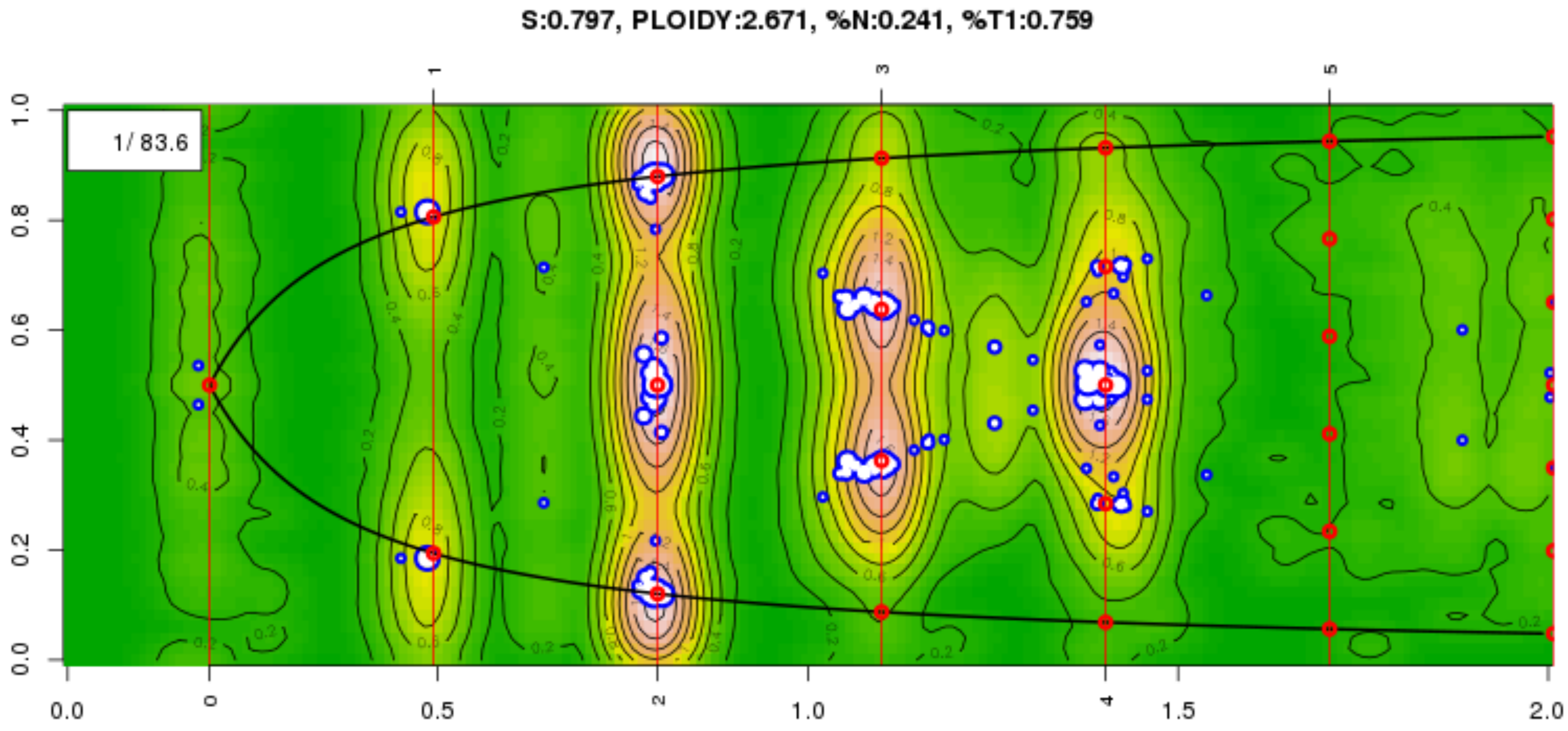

Xenograft

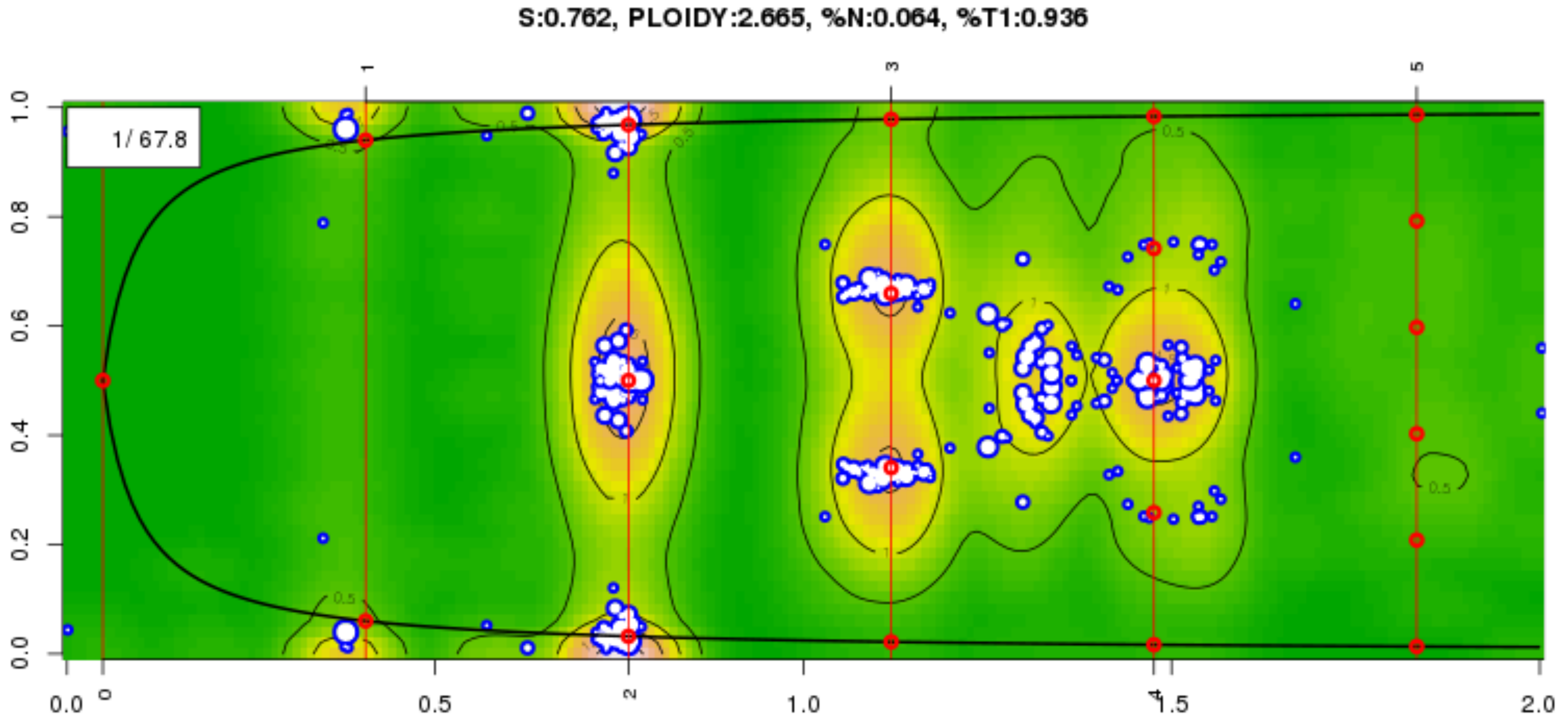

Supplement: S7 Fig — (PDF) [file pcbi.1006596.s007.pdf]
