# Supplementary material for: Whole genomes define concordance of matched primary, xenograft, and organoid models of pancreas cancer
Source: PLoS Comput Biol. 2019 Jan 10;15(1):e1006596. doi: 10.1371/journal.pcbi.1006596 (PMC6328084; doi:10.1371/journal.pcbi.1006596)
Supplement: S9 Fig — Complete CELLULOID solutions (solutions 1–5) are also provided for each of the samples which have been excluded from the analysis (PCSI_0592 and PCSI_0602). (PDF) [file pcbi.1006596.s009.pdf]

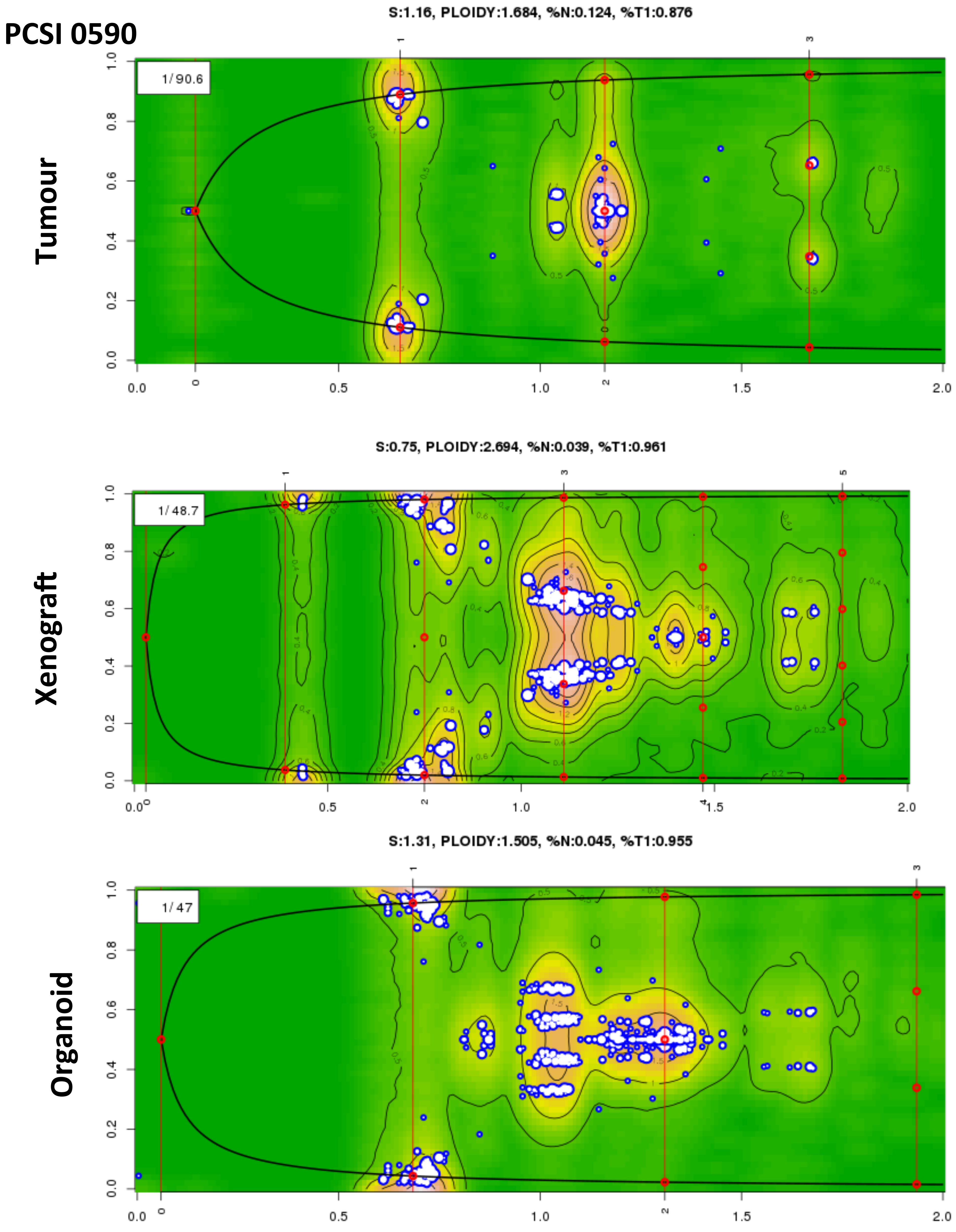
PCSI 0590
Tumour
S:1.16, PLOIDY:1.684, %N:0.124, %T1:0.876
1/90.6
Xenograft
S:0.75, PLOIDY:2.694, %N:0.039, %T1:0.961
1/48.7
Organoid
S:1.31, PLOIDY:1.505, %N:0.045, %T1:0.955
1/47

PCSI 0624

Tumour

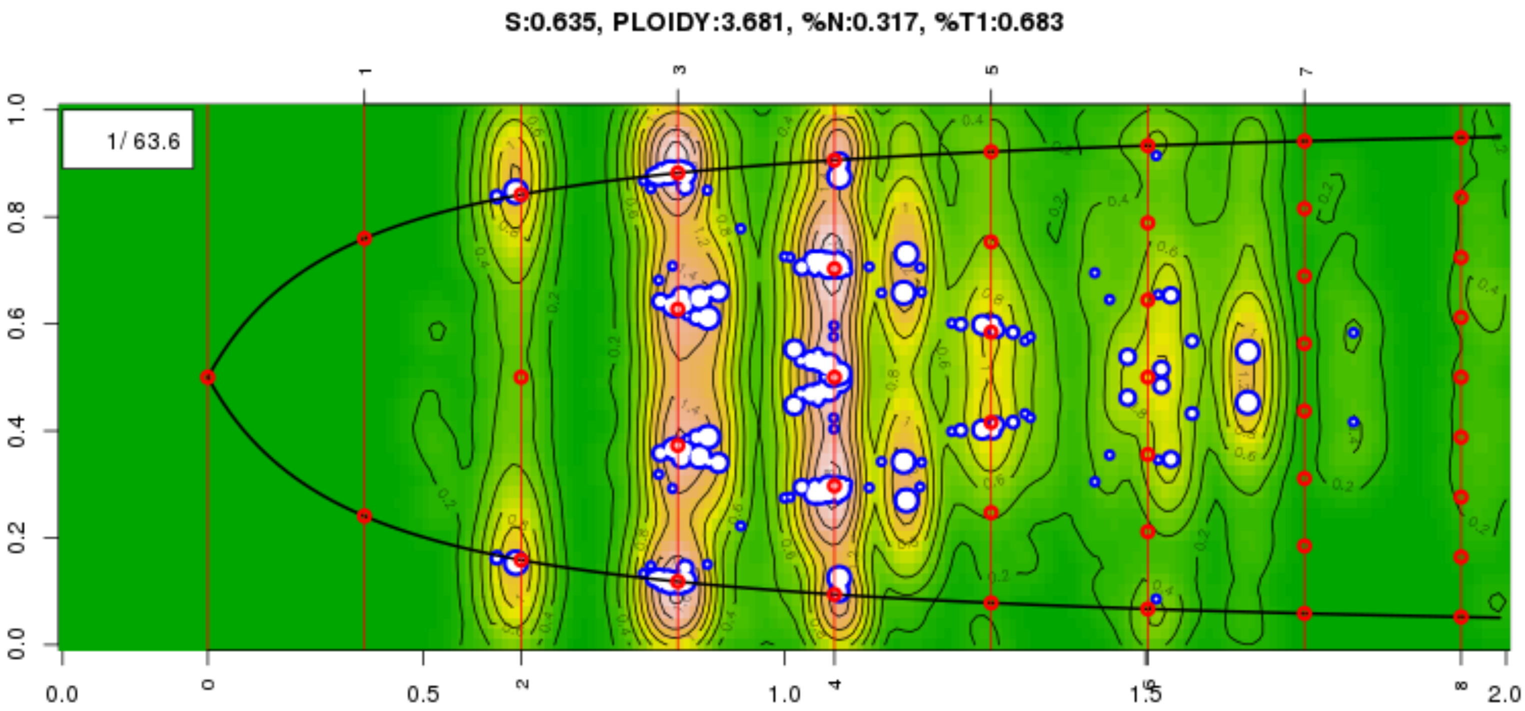

Xenograft

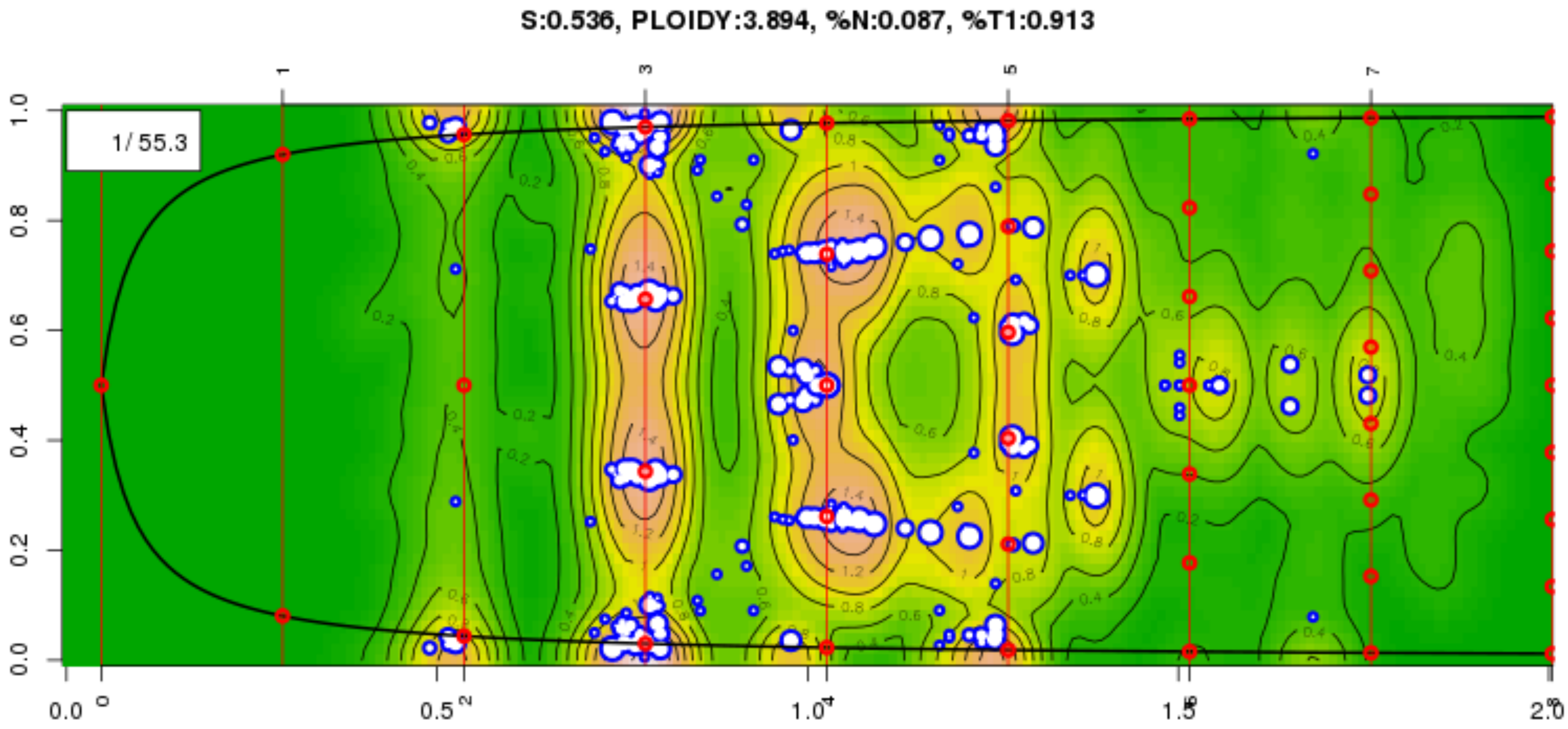

Organoid

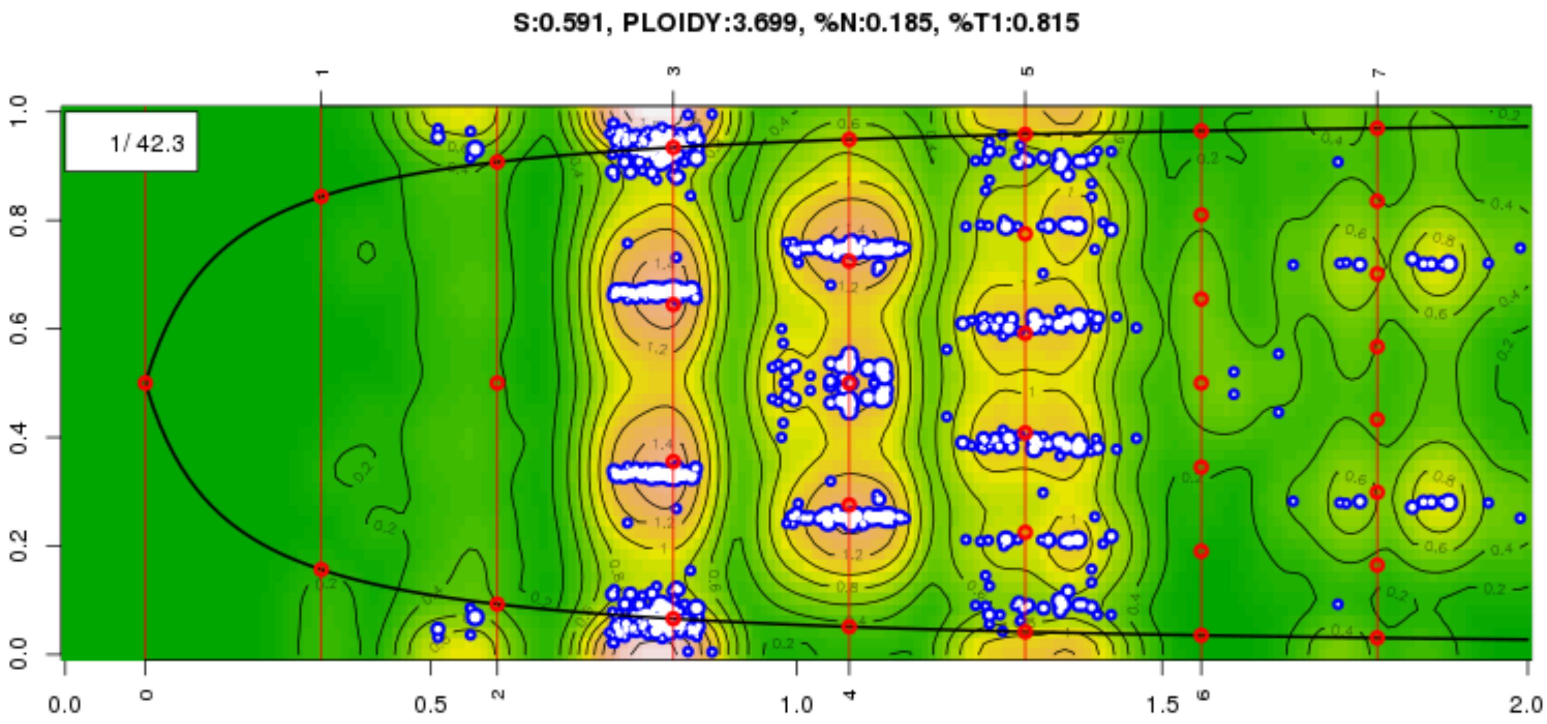

PCSI 0642

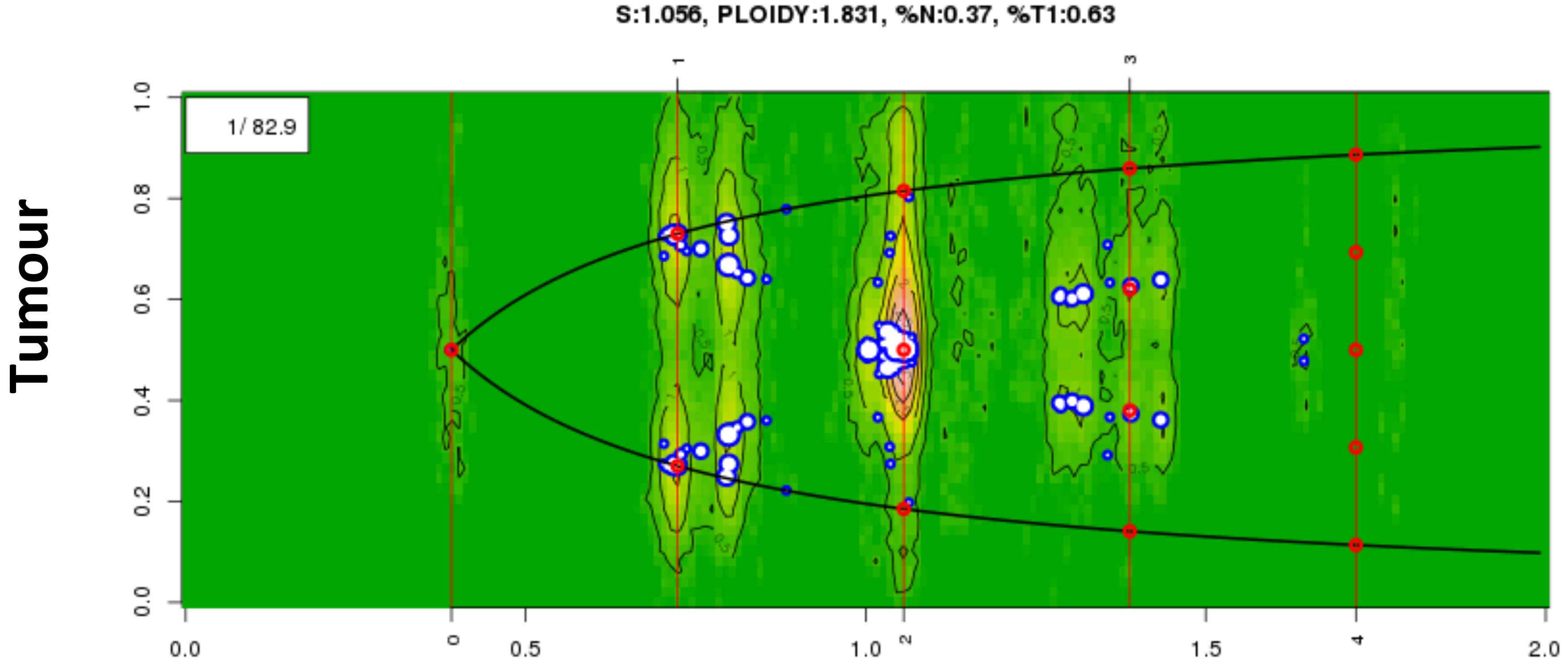
S:1.056, PLOIDY:1.831, %N:0.37, %T1:0.63
Tumour
1/ 82.9
0.0
0.2
0.4
0.6
0.8
1.0
0.0
0.5
1.0
1.5
2.0
0
1
2
3
4

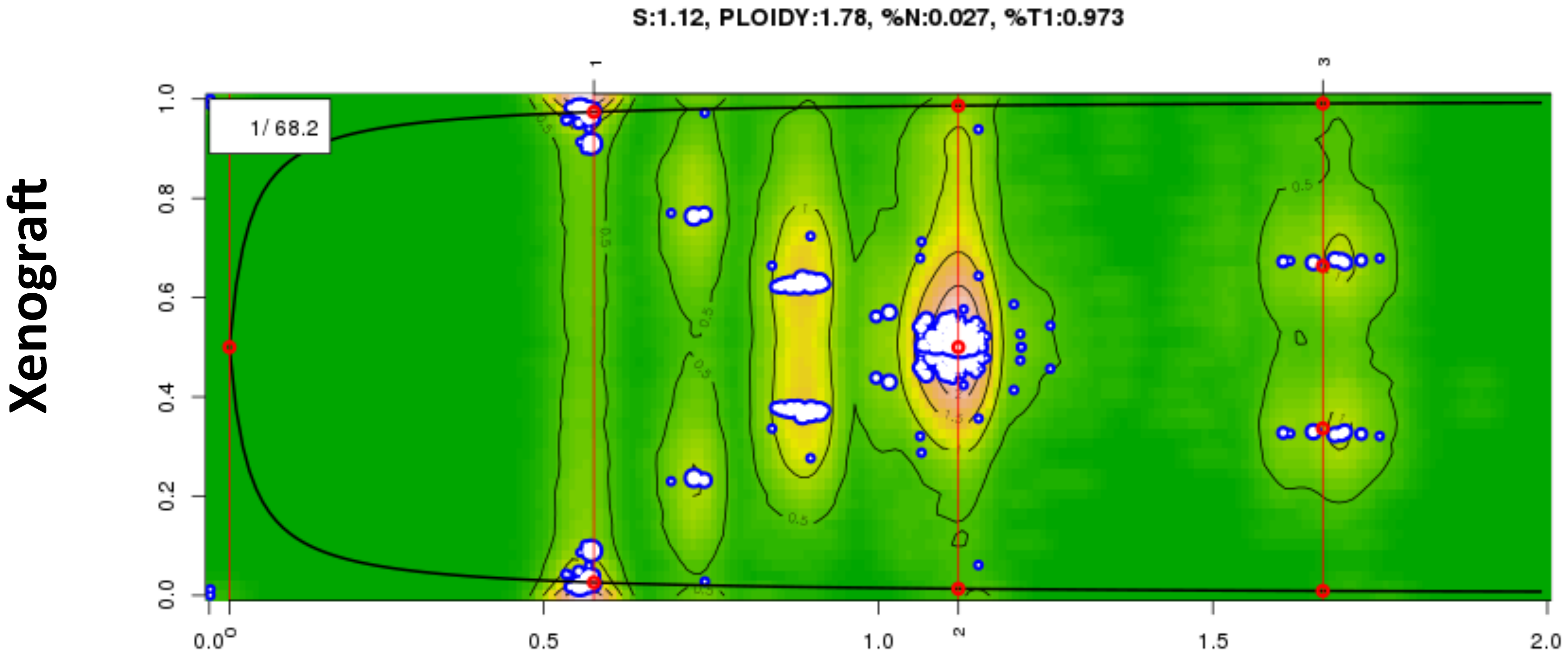
S:1.12, PLOIDY:1.78, %N:0.027, %T1:0.973
Xenograft
1/ 68.2
0.0
0.2
0.4
0.6
0.8
1.0
0.0
0.5
1.0
1.5
2.0
0
1
2
3

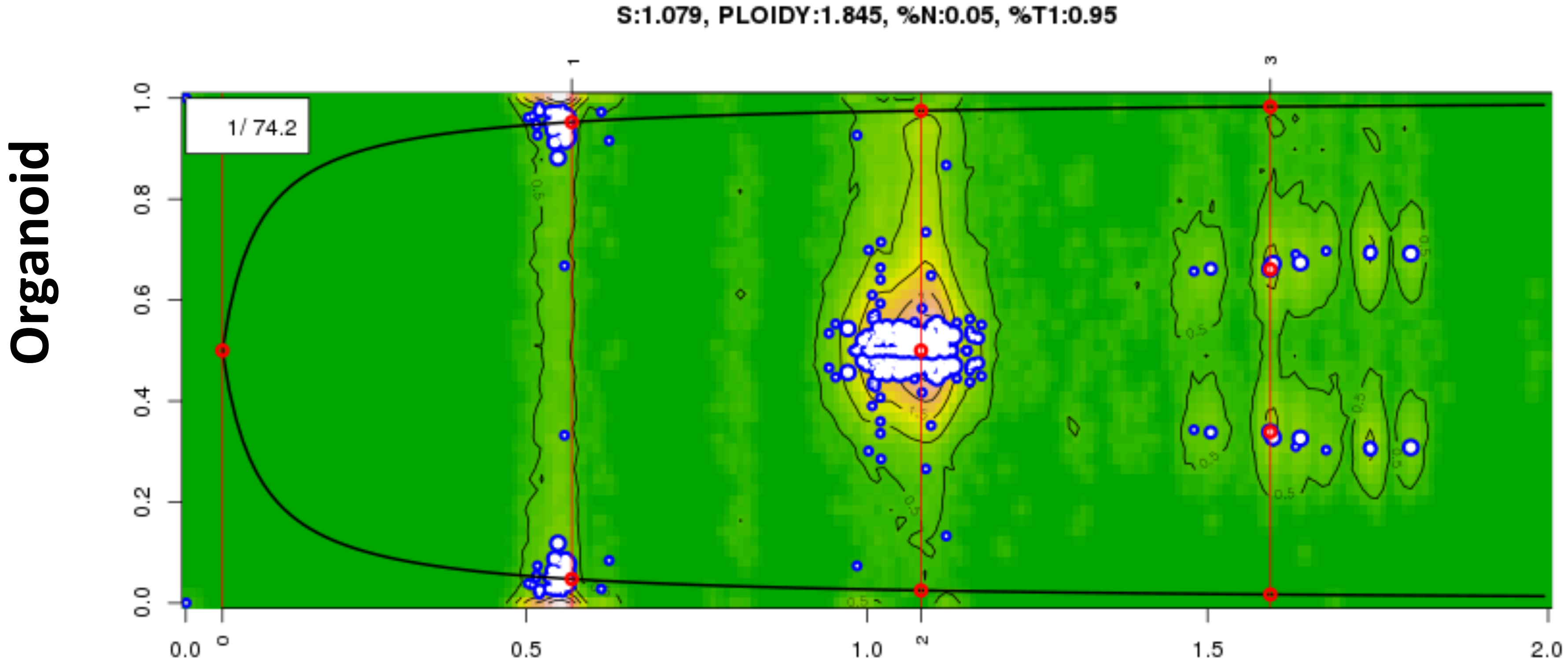
S:1.079, PLOIDY:1.845, %N:0.05, %T1:0.95
Organoid
1/ 74.2
0.0
0.2
0.4
0.6
0.8
1.0
0.0
0.5
1.0
1.5
2.0
0
1
2
3

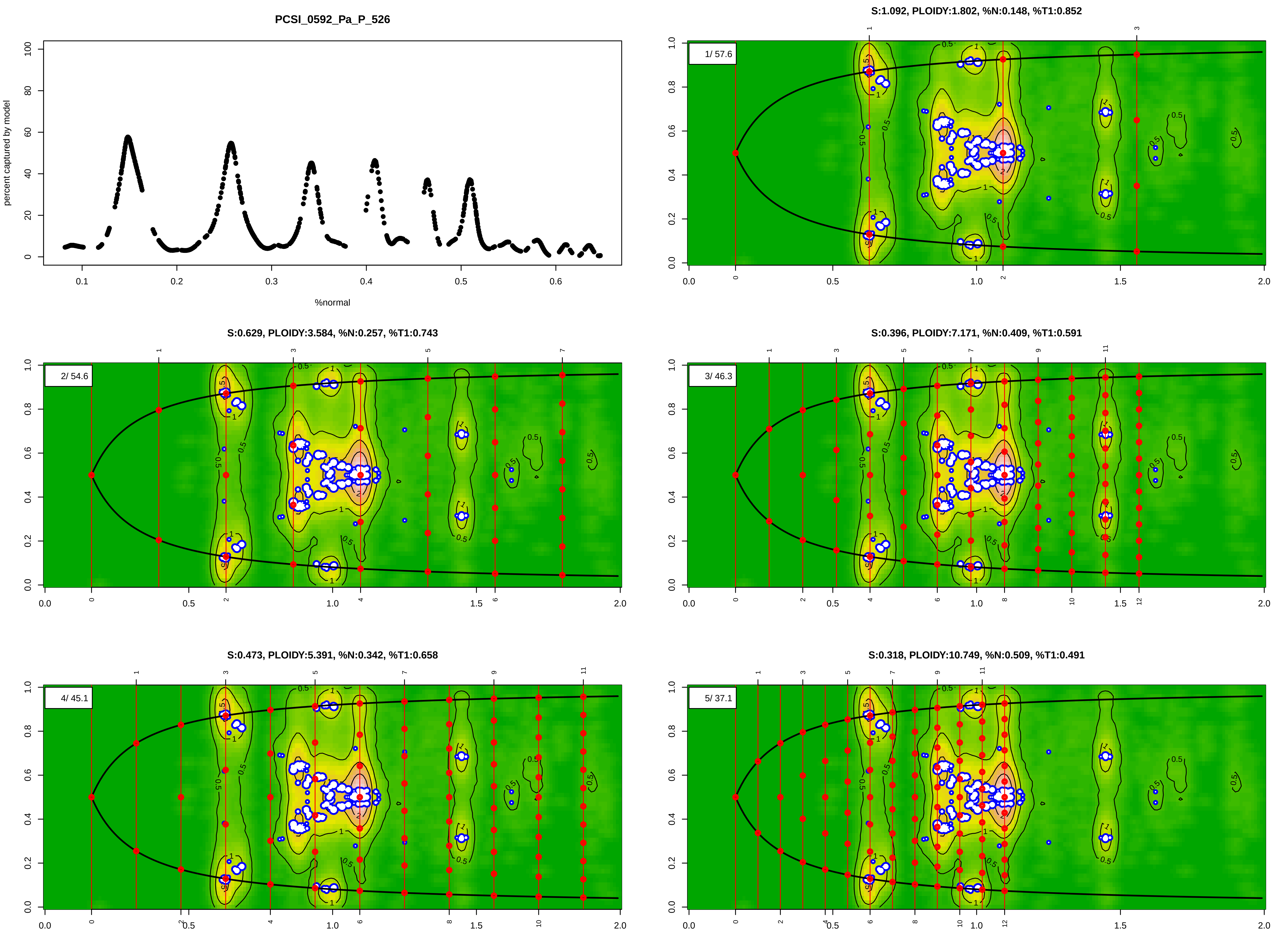
PCSI_0592_Pa_P_526
percent captured by model
%normal
S:1.092, PLOIDY:1.802, %N:0.148, %T1:0.852
1/ 57.6
S:0.629, PLOIDY:3.584, %N:0.257, %T1:0.743
2/ 54.6
S:0.396, PLOIDY:7.171, %N:0.409, %T1:0.591
3/ 46.3
S:0.473, PLOIDY:5.391, %N:0.342, %T1:0.658
4/ 45.1
S:0.318, PLOIDY:10.749, %N:0.509, %T1:0.491
5/ 37.1

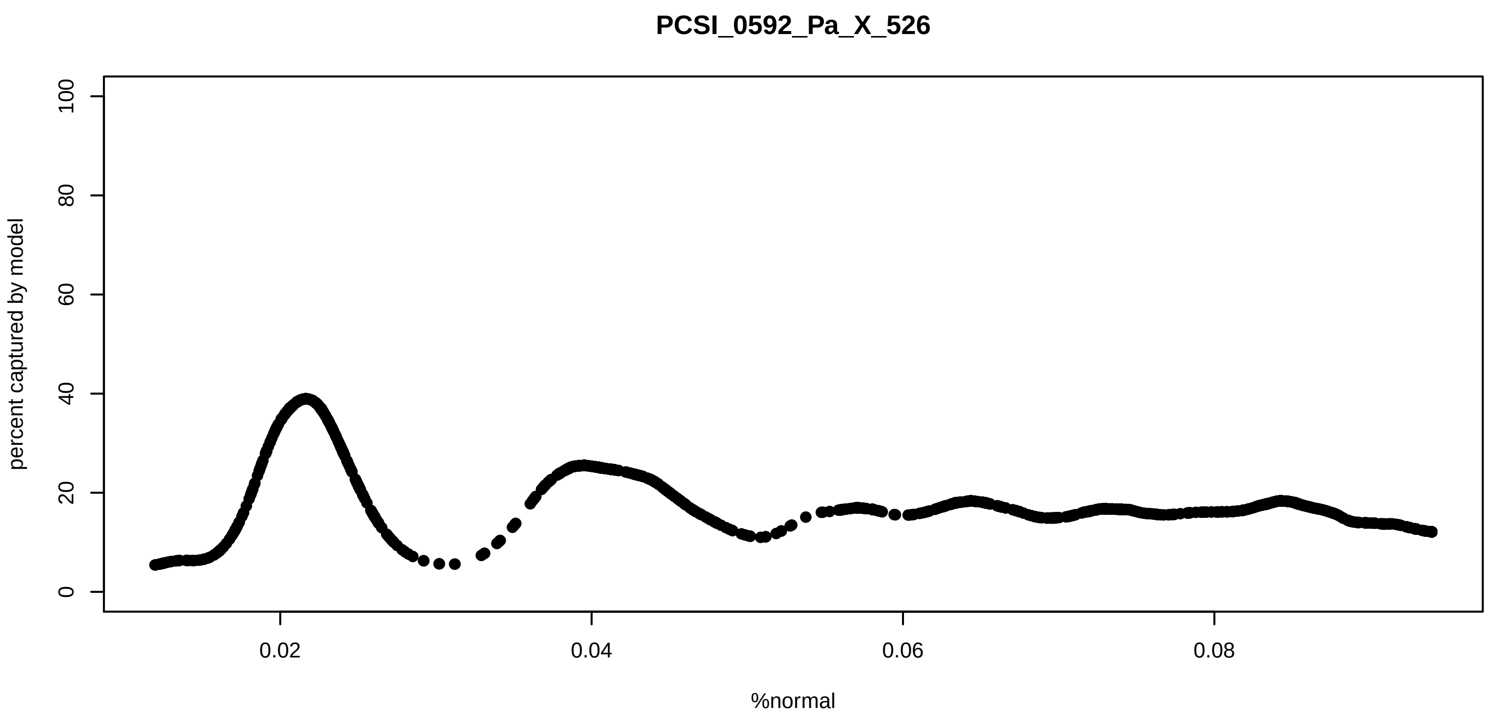
PCSI_0592_Pa_X_526
percent captured by model
%normal

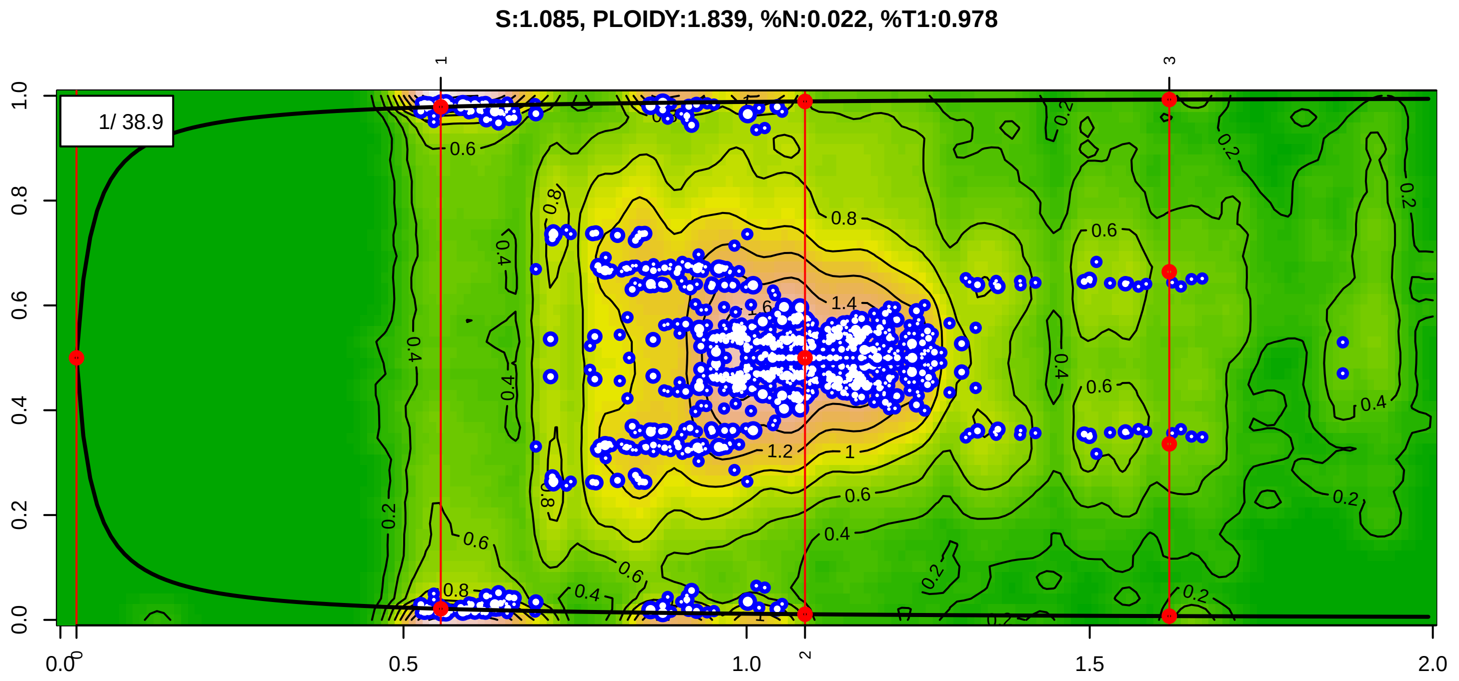
S:1.085, PLOIDY:1.839, %N:0.022, %T1:0.978
1/ 38.9

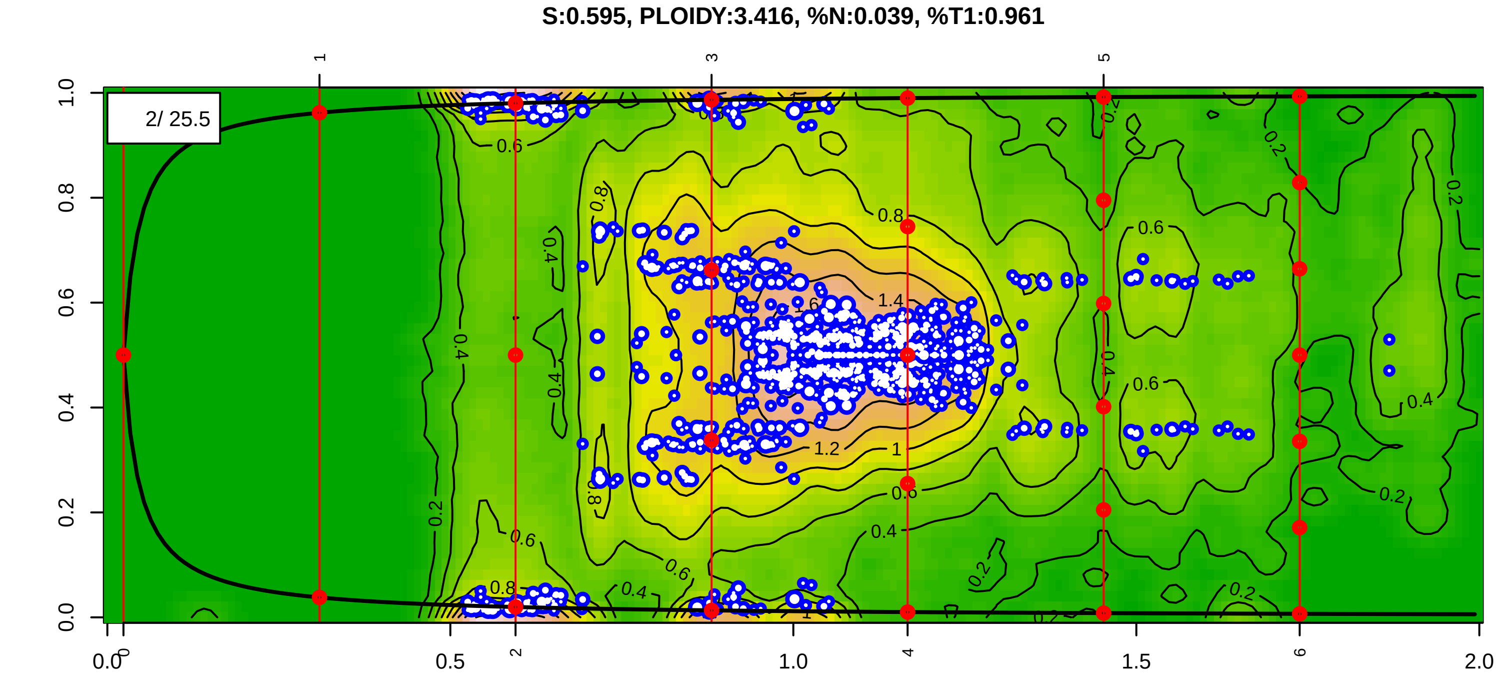
S:0.595, PLOIDY:3.416, %N:0.039, %T1:0.961
2/ 25.5

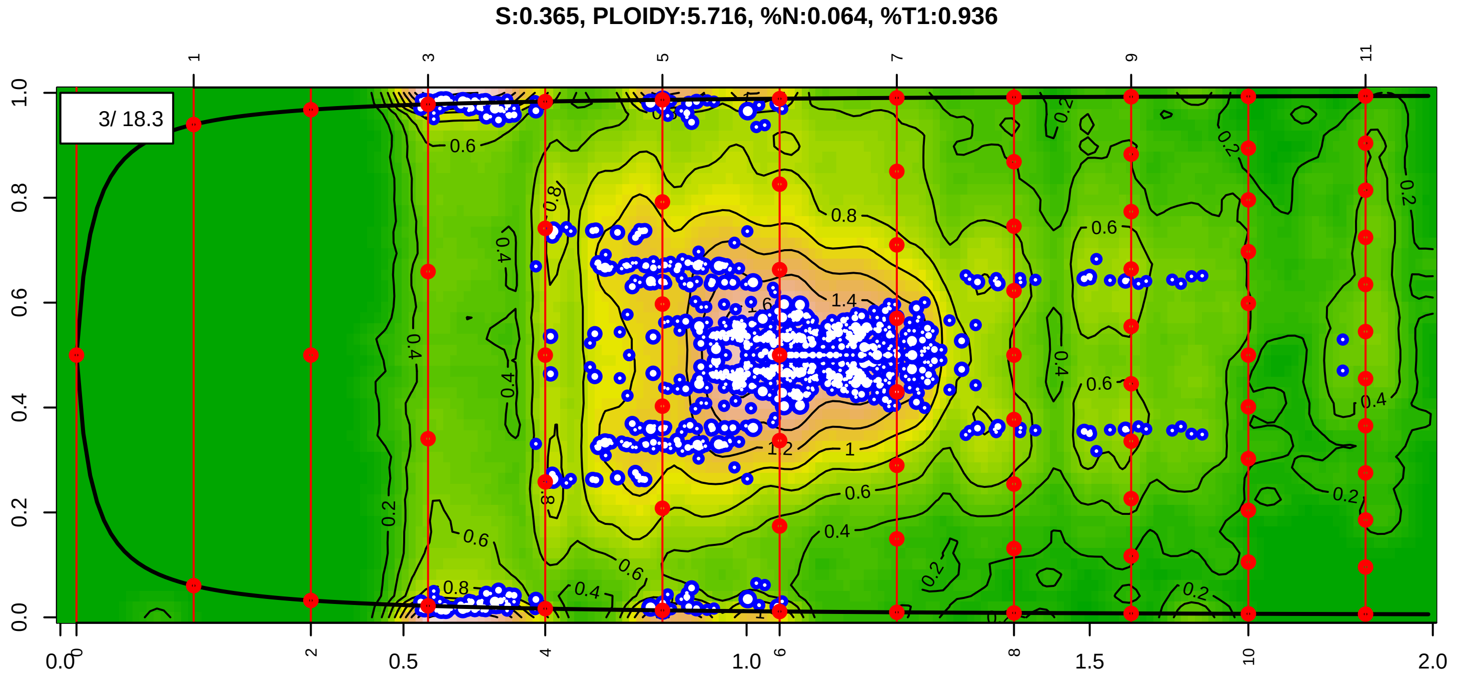
S:0.365, PLOIDY:5.716, %N:0.064, %T1:0.936
3/ 18.3

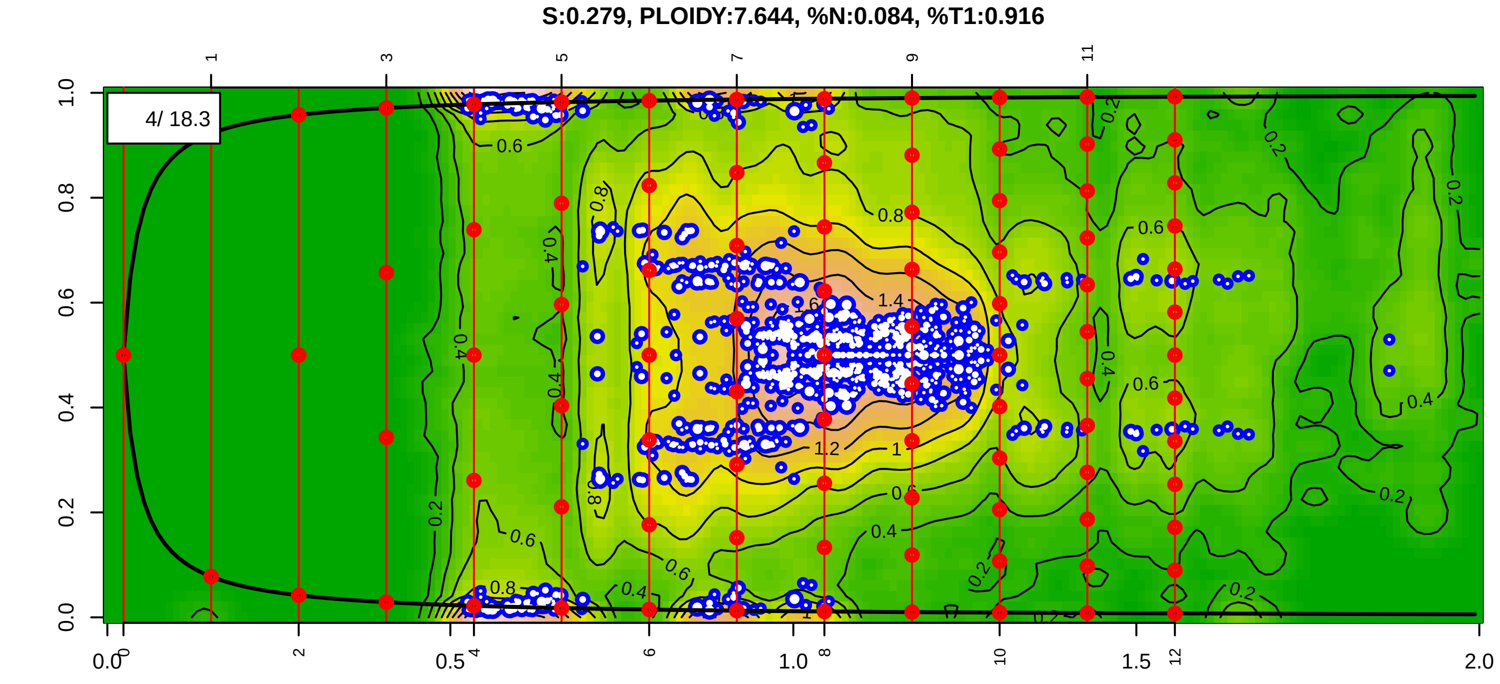
S:0.279, PLOIDY:7.644, %N:0.084, %T1:0.916
4/ 18.3

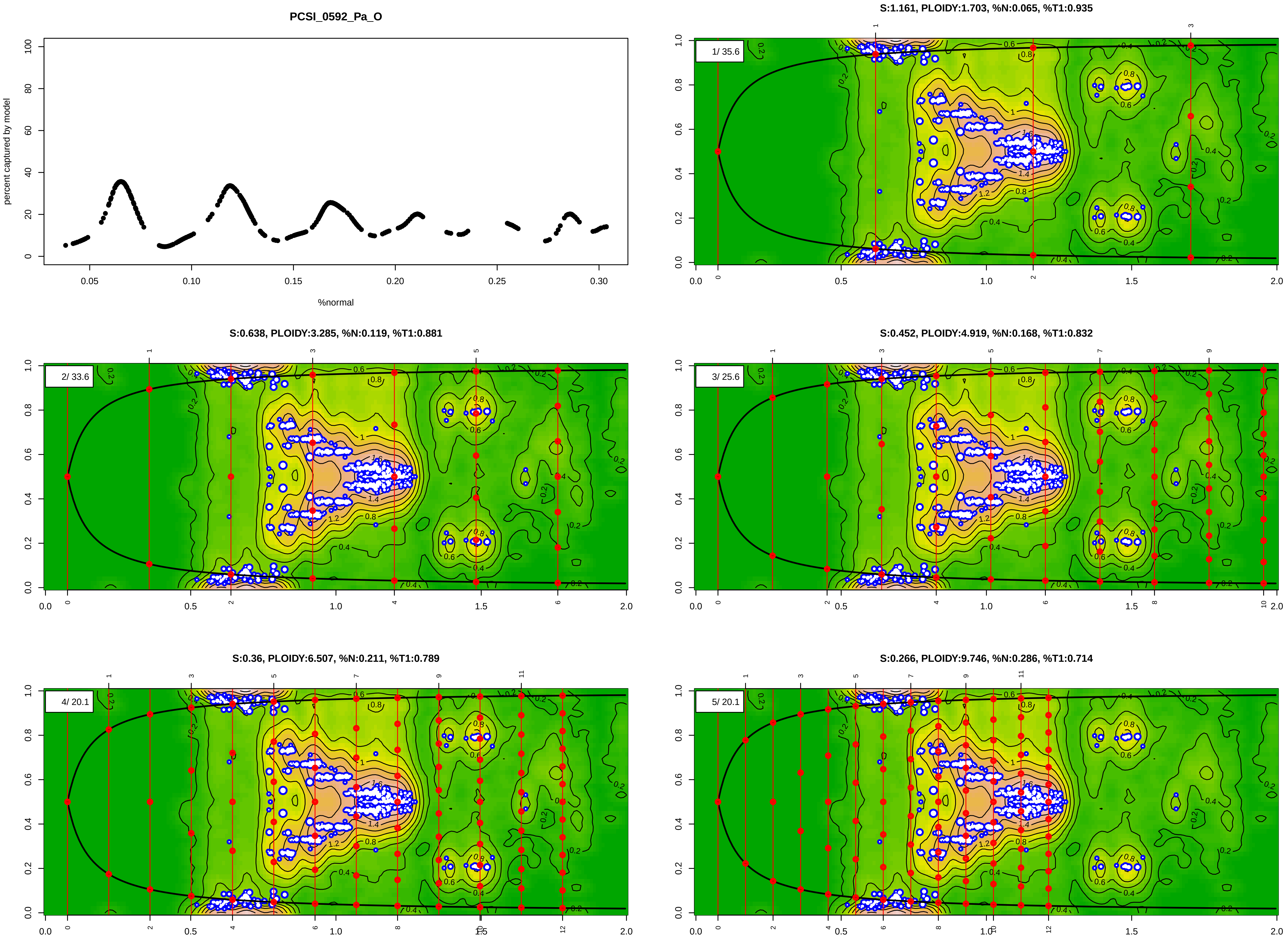

PCSI_0592_Pa_O
percent captured by model
%normal
S:1.161, PLOIDY:1.703, %N:0.065, %T1:0.935
1/ 35.6
S:0.638, PLOIDY:3.285, %N:0.119, %T1:0.881
2/ 33.6
S:0.452, PLOIDY:4.919, %N:0.168, %T1:0.832
3/ 25.6
S:0.36, PLOIDY:6.507, %N:0.211, %T1:0.789
4/ 20.1
S:0.266, PLOIDY:9.746, %N:0.286, %T1:0.714
5/ 20.1

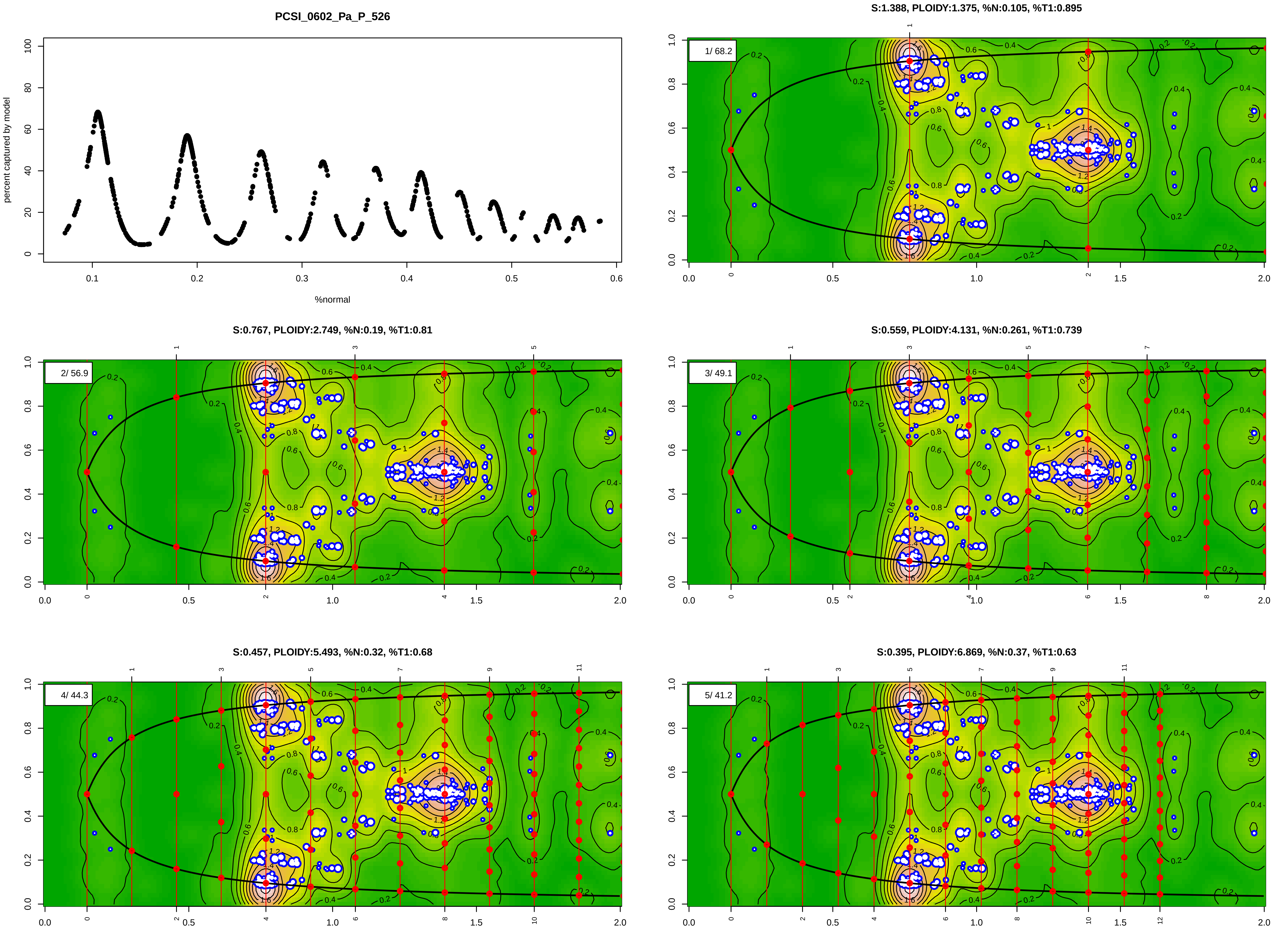

PCSI_0602_Pa_P_526
percent captured by model
%normal
S:1.388, PLOIDY:1.375, %N:0.105, %T1:0.895
1/ 68.2
S:0.767, PLOIDY:2.749, %N:0.19, %T1:0.81
2/ 56.9
S:0.559, PLOIDY:4.131, %N:0.261, %T1:0.739
3/ 49.1
S:0.457, PLOIDY:5.493, %N:0.32, %T1:0.68
4/ 44.3
S:0.395, PLOIDY:6.869, %N:0.37, %T1:0.63
5/ 41.2

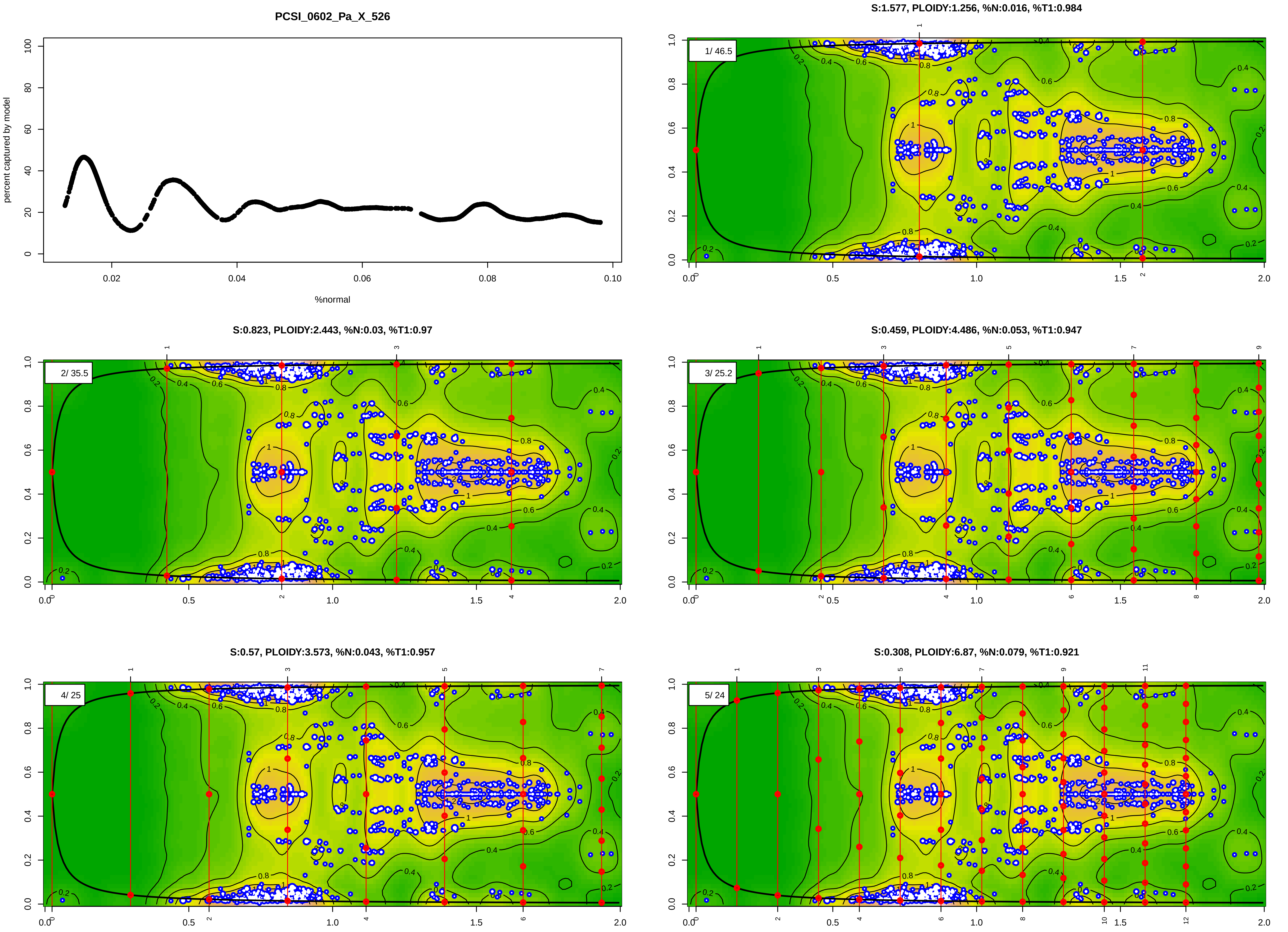

PCSI_0602_Pa_X_526
percent captured by model
%normal
S:1.577, PLOIDY:1.256, %N:0.016, %T1:0.984
1/ 46.5
S:0.823, PLOIDY:2.443, %N:0.03, %T1:0.97
2/ 35.5
S:0.459, PLOIDY:4.486, %N:0.053, %T1:0.947
3/ 25.2
S:0.57, PLOIDY:3.573, %N:0.043, %T1:0.957
4/ 25
S:0.308, PLOIDY:6.87, %N:0.079, %T1:0.921
5/ 24

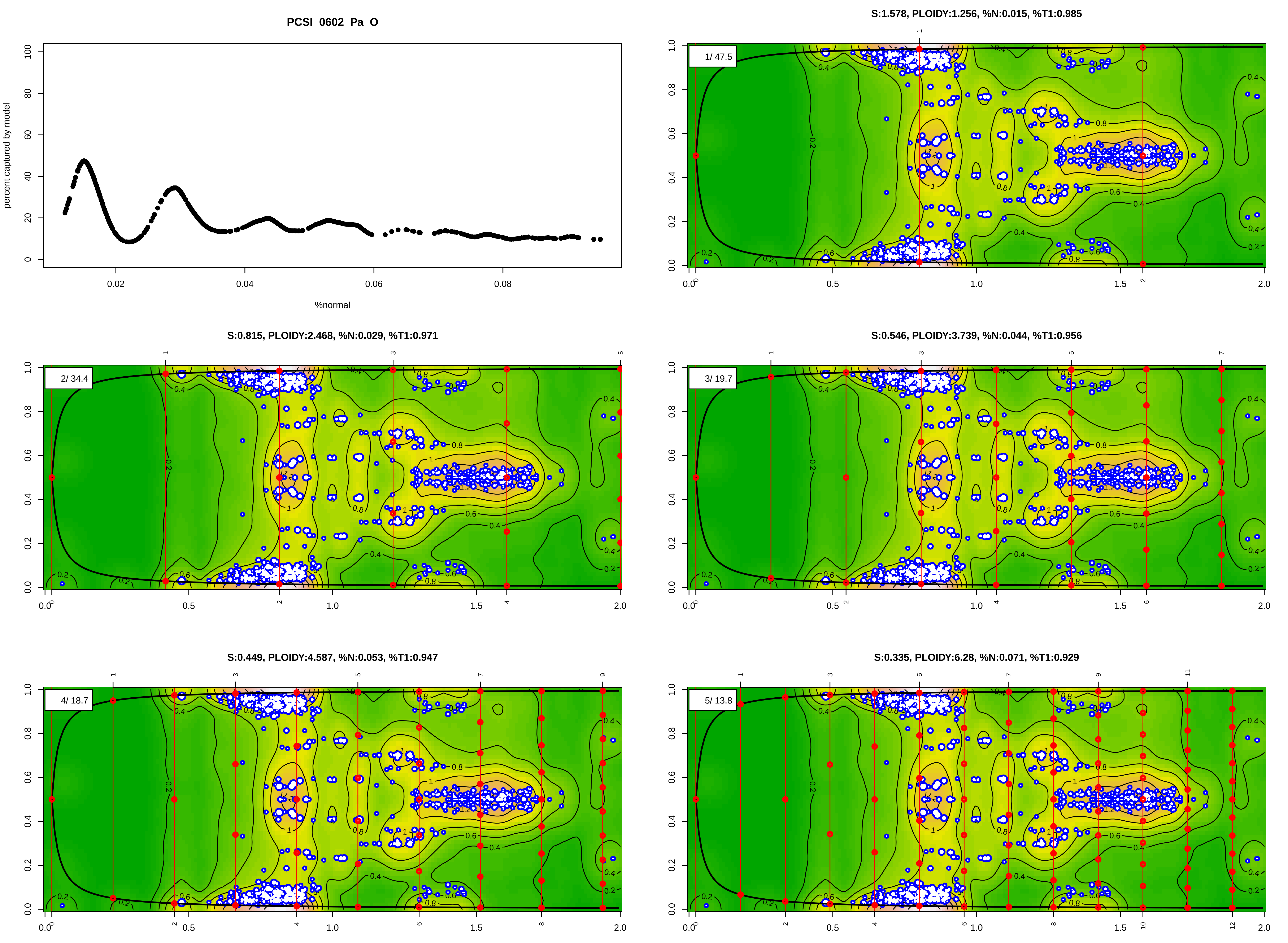
PCSI_0602_Pa_O
percent captured by model
%normal
S:1.578, PLOIDY:1.256, %N:0.015, %T1:0.985
1/ 47.5
S:0.815, PLOIDY:2.468, %N:0.029, %T1:0.971
2/ 34.4
S:0.546, PLOIDY:3.739, %N:0.044, %T1:0.956
3/ 19.7
S:0.449, PLOIDY:4.587, %N:0.053, %T1:0.947
4/ 18.7
S:0.335, PLOIDY:6.28, %N:0.071, %T1:0.929
5/ 13.8
